# Cohort profile: The Growing Up Healthy Study (GUHS)—A prospective and observational cohort study investigating the long-term health outcomes of offspring conceived after assisted reproductive technologies

**DOI:** 10.1371/journal.pone.0272064

**Published:** 2022-07-22

**Authors:** Blagica Penova-Veselinovic, Laura A. Wijs, John L. Yovich, Peter Burton, Roger J. Hart

**Affiliations:** 1 Division of Obstetrics and Gynaecology, University of Western Australia, Perth, WA, Australia; 2 School of Pharmacy and Biomedical Science, Curtin University, Perth, WA, Australia; 3 Faculty of Health Science, School of Health and Medical Sciences, Edith Cowan University, Perth, WA, Australia; Fondazione IRCCS Ca’ Granda Ospedale Maggiore Policlinico, ITALY

## Abstract

Worldwide, over 8 million children and adults are conceived following assisted reproductive technologies (ART), and their long-term health is of consequential public health interest. The objective of this paper is to describe the Growing up Healthy Study (GUHS) cohort in detail, publicise it and invite collaboration. Combining the data collected in the GUHS with other cohorts or databases will improve the much-needed knowledge about the effects of ART, and allow for better understanding of the long-term health outcomes of offspring conceived after ART. The GUHS cohort is a prospective observational study of adolescents and young adults conceived after assisted reproductive technologies (ART). It was established to determine if the long-term health of offspring conceived by ART differs from that of the general population. This was investigated by comparing a substantial number of health parameters to those of a representative population of offspring conceived without ART. The n = 303 GUHS participants were born between 1991–2001 in the two fertility clinics operating at the time in Perth, Western Australia, and undertook assessments at ages 14, 17 and 20, replicating the pre-defined study protocols from the reference cohort—the Raine Study. Participants were comprehensively phenotyped through detailed questionnaires, anthropometry, biochemical analyses, as well as age-specific assessments (asthma, atopy, cardiometabolic health, body composition, mental health, thyroid function, epigenetics and vision). To date the GUHS cohort has been used to study the methylation, cardiometabolic, and thyroid profiles, as well as respiratory and mental health. To summarise, the GUHS cohort provides a valuable addition to the limited knowledge of the long-term health outcomes of ART-conceived offspring.

## Introduction

The long-term health of children conceived following assisted reproductive technologies (ART), such as in vitro fertilisation (IVF) and intracytoplasmic sperm injection (ICSI) is of substantial public health interest. Over 8 million children and adults have been conceived by ART worldwide [1], and currently 1 in 20 children born in Australia were conceived following such techniques [2].

Considerable data is available from short-term follow-ups, reporting an increased risk of adverse health outcomes such as: congenital malformations, intra-uterine growth restriction [3] and neonatal complications [4]. From the limited number of longer-term follow-up studies, differences in respiratory [5], cardiovascular [6] and cardiometabolic health [7], and thyroid function [8] have also been reported in the ART conceived children [9–12]. Epigenetic alterations have been proposed as underlying mechanisms for the observed adverse health outcomes in the ART-conceived offspring [13,14]. The ART manipulations occurring during the very early developmental period, which is characterized by extensive epigenetic reprogramming, may indirectly alter normal development and long-term health outcomes of these offspring [15,16]. However, to date there is no common agreement on whether the potential differences observed in ART-born offspring are driven by the laboratory techniques used in ART, or perhaps by the intrinsic features of the infertile couple [17].

Long-term prospective studies investigating the health of adolescent and adults conceived through ART are therefore of great importance and consequential public health interest. To this end we established the Growing Up Healthy Study (GUHS), a prospective observational cohort study, to investigate an extensive number of long-term health parameters in adolescence and young adulthood. The outcomes of these assessments were compared to those of offspring of similar age and sex, conceived without ART, from the Raine Study [18].

The objective of this paper is to describe the GUHS in detail, as well as publicise the cohort and invite collaboration. Combining the data collected in the GUHS with other cohorts or databases will improve the much-needed knowledge about the effects of these widely used fertility treatments, and better understand the long-term health outcomes of offspring conceived after ART.

## Cohort description

### Study design and recruitment process

Parents who underwent ART treatment in the only two operating fertility clinics in Perth in the 1990’s (Concept fertility and PIVET Medical Centre), were approached via their treating fertility clinic, to seek formal consent for the family to participate in the GUHS. If the letter seeking permission was returned to sender or no response was received, updated addresses were received through data linkage using the Western Australian electoral roll. Following the receipt of the consent, information about the study was sent to the families, followed by an invitation for their child to attend the age-appropriate assessment. Inclusion criteria were as follows: only families that were successfully treated with IVF or ICSI (excluding gamete intrafallopian transfer (GIFT), intra-uterine insemination (IUI) and ovarian stimulation) between 1991–2001 were invited to enrol. The recruitment process ran concurrently for all three age groups to maximise the number of participants per follow-up and minimise burden to the participating families. Depending on the age at enrolment, a number of participants were eligible to complete two follow-ups (N = 63 participated in both the 14- and 17- year follow-up, and N = 14 participated in both the 17- and 20- year follow up). The remaining N = 226 participants completed one follow-up only.

**Fig 1:**
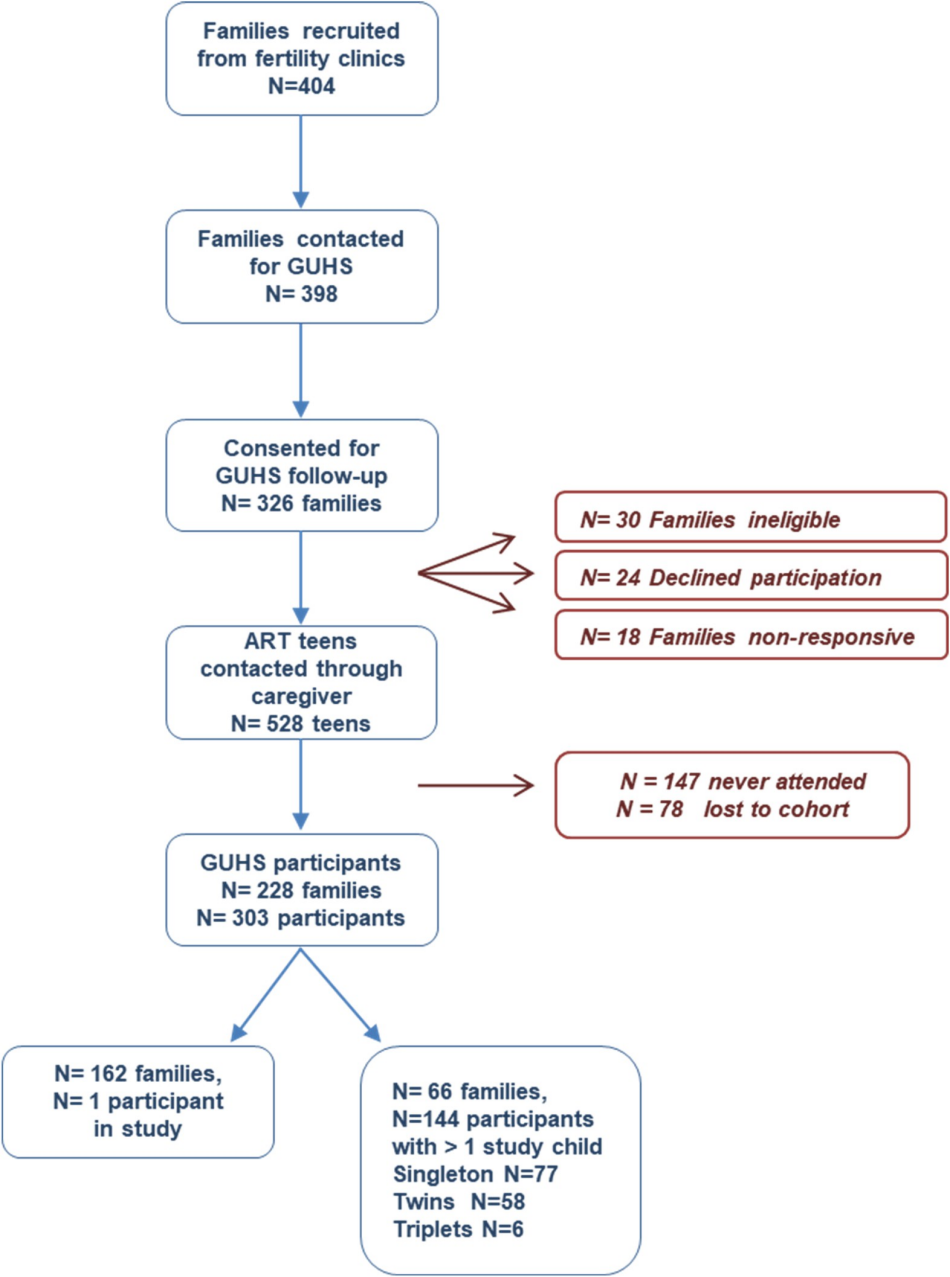
Flowchart of study recruitment process. ART: Assisted reproductive technologies; GUHS: Growing Up Healthy Study.

Of the 528 eligible adolescents and young adults, a final number of n = 303 took part in the study. Of these, 266 had confirmed conceptions via IVF or ICSI, from both fresh (ET) and frozen (FET) embryo transfers. For sixteen participants who were initially confirmed as IVF or ICSI, further investigation of fertility records revealed that 15 were conceived through GIFT and one through IUI. As these participants had already consented to and participated in assessments, they were retained in the study. Twenty-one participants had a confirmed ART status, but unconfirmed which type (i.e. IVF or ICSI). The full recruitment process can be viewed in **Fig 1**.

The included participants underwent comprehensive phenotype assessments through questionnaires, physical assessments, as well as blood and urine analyses. Age-specific assessments were conducted at three different follow-ups, at ages 14, 17 and 20, with targeted interest for specific health outcomes (**Table 1**). The GUHS offspring replicated the exact same predefined assessments previously completed by their counterparts from the Raine Study, conceived without ART. The Raine Study is a former pregnancy cohort study, established (1989–1991) to investigate the effect of perinatal health on subsequent childhood and adult health [18]. The Raine Study offspring have also been recognised to be representative of the Western Australian adolescent population at the time of assessments [19]. The outcomes of these assessments were then compared between the cohorts. The assessments for GUHS took place between 2013 and 2017, about 1 and 14 years after the Raine Study assessments. Both cohorts were assessed in the Raine Study House and the Centre for Sleep Science located on the campus of the University of Western Australia, Crawley, Western Australia.

**Table 1:** Overview of conducted assessments per follow-up.

Assessment	14 years (N)	17 years (N)	20 years (N)
Anthropometric assessments	152	165	61
**Questionnaires**			
Participant questionnaire	152	165	61
Participant confidential questionnaire	-	165	-
Participant medical questionnaire	-	151	61
Participant thinking questionnaire	-	-	60
Primary caregiver questionnaire	152	165	-
Food frequency questionnaire	153	163	58
DASS-21	-	-	61
CBCL	152	165	-
YSR	152	165	-
Cowen self-efficacy assessment	152	165	-
Beck’s depressive inventory	152	165	-
**Cognitive function**			
CogState	-	126	-
**Biochemistry**			
Full blood picture	132	154	52
Glucose homeostasis	134	154	61
Thyroid function	134	-	61
Lipids and cardiac measures	134	154	61
Liver function and kidney function	134	154	61
Iron studies	-	154	-
Vitamin D	134	154	61
DNA for epigenetics	273 total		
**Urine analyses**			
Kidney function	-	148	-
**Asthma and allergy**			
Spirometry	147	-	-
Methacholine challenge	141	-	-
Skin prick testing	142	-	-
**Cardiometabolic health**			
Sphygmocor	-	161	-
Abdominal ultrasonography	-	138	-
**Body composition**			
DEXA-scan	-	-	63
**Vision**			
Auto-refraction	-	-	60

DASS-21: Depression, Anxiety and Stress Scale; CBCL: Child Behaviour Checklist; YSR: Youth Self-Report; DEXA: Dual energy X-ray absorptiometry scan.

Due to the relatively static population and remote geolocation of Perth, Western Australian women receive fertility treatments within the State, and the cohort is not contaminated by couples who come to Western Australia solely for fertility treatment. This makes the ART cohort representative of the Western Australian ART population. Comparing replicated assessments between these two representative cohorts provides a unique opportunity that would be difficult to replicate elsewhere in the world.

### Ethics approval

The University of Western Australia, Human Research Ethics Committee approved the overall study (RA/4/1/5860), as well as the approach of the parents via the fertility clinics (RA/4/1/2417). Further approval was granted by the Western Australian Department of Health, Human Research Ethics Committee (2013/25) for data linkage using the electoral roll and data from the Midwives Notification System.

Informed and written consent, including genetic assessment consent, was obtained from all adult members of the participating families (>18 years of age) at each follow-up. For minors (<18 years of age), parents signed a ‘Parent/Participant Consent Form’ and the minors themselves signed an assent for their involvement. (**S1 File**).

### Data collection

#### Clinical information

Comprehensive clinical information at the time of conception, including all aspects of the successful ART cycle, type of ART (IVF/ICSI, fresh/frozen), causes of a couples’ subfertility, as well as pregnancy outcomes were collected retrospectively from the two fertility clinics. Detailed embryological data of the index pregnancy, number and type of embryos transferred, along with details on media and duration of embryo incubation, type of ovarian stimulation used, and maximum level of maternal oestradiol were also collected.

Additional information regarding pregnancy (outcomes) was collected from the Midwives Notification System records, via data linkage through the Western Australia Department of Health. The obtained data was primarily used in case of missing information regarding pregnancy outcomes from the fertility clinics, as well as to cross check and validate variables, as fertility clinics do not always hold information regarding pregnancy outcomes.

#### Routine assessments of the GUHS adolescents and young adults

*Questionnaires*. At all three follow-ups, the study participants answered their own questionnaires with questions appropriate for their age and with increasing complexity and detail. The questionnaire included general information regarding their physical health, self-reported pubertal development (Tanner staging), mental health, physical and social activity, relationships including family members’ support, eating, and drinking habits, education and/or occupation. At the 14-year follow-up this questionnaire included questions regarding smoking, alcohol and drug use. At the 17- and 20-year follow-up, these questions, as well as questions regarding relationships and sexual experience, were included in a separate confidential questionnaire. Information on a range of medical conditions was provided in a separate medical history questionnaire.

At the 14- and 17-year follow-up, the primary caregiver provided information on social determinants and quality of life of the family (housing, environment, primary caregiver health and wellbeing). A second primary caregiver questionnaire collected information on the participant’s physical activity, social behaviour, mental health, learning and education. Detailed medical questions were also included with emphasis on a wide range of doctor-diagnosed conditions, hospitalisation, injuries and the use of prescription and non-prescription medications, alongside a brief medical history of all family members.

At each follow-up, age-specific standardised questionnaires were completed. At all three follow-ups a standardised food-frequency questionnaire was completed measuring the dietary intake. This Dietary Questionnaire for Epidemiological Studies (DQES v.2.0) [20] was a modified version from the questionnaire developed by the Cancer Council Victoria [21]. At the 17-year follow-up participants completed the International Physical Activity Questionnaire (IPAQ) [22]. At the 14- and 17-year follow-up the Youth Self-Report [23], Cowen self-efficacy assessment [24] and Beck’s youth inventory [25] were administered. At the 20-year follow-up, the Depression, Anxiety and Stress Scale (DASS-21) [26], as well as the Autism-spectrum Quotient (AQ) [27] were administered. At the 14- and 17-year follow-up the primary caregiver completed the Child Behaviour Checklist (CBCL) [23].

All questionnaires are available as **S2–S4 Files** for the 14-, 17- and 20-year follow-up respectively.

*Blood sample and urine analysis*. Following informed consent, fasting venous blood samples were taken by a trained phlebotomist, in three different collection tubes containing the appropriate preserving agent for the specific measured analytes. Samples were delivered to the PathWest Laboratory in Perth, Western Australia, Australia, within two hours of phlebotomy for immediate analysis. The remaining aliquots of serum, plasma and urine samples have been appropriately archived at -80°C for long term storage.

The following analyses were performed: glucose homeostasis, including a calculation of the homeostatic model assessment for insulin resistance (HOMA-IR) [Fasting insulin (mU/L) X (fasting glucose (mmol/L)/22.5)] [28]; thyroid, kidney and liver function tests; iron studies; lipids and cardiac measurements, Vitamin D, fatty acids, as well as plasma and urine spot analyses for kidney function. The detailed methodology and a full list of analyses is presented in **S1 Table.**

### Genomic DNA extraction, DNA methylation profiling and quality control

Genomic DNA was extracted from whole blood using the Promega Reliaprep Large volume HT g DNA Isolation System and quantitated on the Qubit 4.0 System in the Western Australian DNA Bank at the Centre for Genetic Origins of Health and Disease, Australia.

*DNA methylation profiling*. Genomic DNA was used for epigenomic profiling, on the Infinium MethylationEpic BeadChip (EPIC Illumina inc, San Diego, CA) platform through PathWest Laboratory Medicine (Perth, Western Australia). EPIC profiles over 850,000 CpG sites, roughly 3% of the human epigenome [29], at a single nucleotide resolution allowing for epigenome wide association to be completed. Independent repeated measurements of the quantified epigenetic marks from 15 participants were used as technical replicates for a total of 288 DNA methylation profiles.

*Quality control*. The pre-processing of the raw EPIC array files was conducted using the RnBeads package in R [30]. Single nucleotide probes—enriched sites, probes with a high likelihood of cross-hybridization and probes with the highest fraction of unreliable measurement were removed. In addition, one DNA methylation profile was removed due to a high number of probes with unreliable measurements. A total of 287 profiles (including 15 replicates where one replicate was used to replace the profile with unreliable measurement) and 793,224 probes were then normalized using the Beta-Mixture Quantile dilation model [31]. Following normalization using this model, 1120 probes were removed due to missing values resulting in 287 profiles (corresponding to n = 273 participants and 14 technical replicates) and a total of 792,104 probes for downstream analysis. To account for potential cell count heterogeneity, six cell types were estimated (CD8T, CD4T, NK, B cell, monocytes and granulocytes) using the minfi package in R [32].

#### Anthropometric assessments

Quantitative anthropometric measurements were collected routinely at all three follow-ups to assess body composition.

Standing height was measured using a stadiometer (to the nearest 0.1 cm) (Wall mounted Holtain stadiometer, United Kingdom) and weight was measured seated, with participants lightly clothed, using an electronic scale (to the nearest 0.1 kg) (Wedderburn Chair Scales, Australia). Body mass index (BMI) was calculated (kg/m^2^).

To improve accuracy of the measurements, the following parameters: waist, hip, and wrist circumference, biacromial and anterior superior iliac spine breadth were measured twice (to the nearest 0.1cm), using a Birch plastic measuring tape, and the average of both measurements was recorded (Birch plastic measuring tape, Australia). Waist to hip ratio was calculated (average waist circumference/average hip circumference, to the nearest 0.1 cm). Similarly, skinfold thickness was measured twice using a calliper, and the average of both measurements was recorded (triceps, subscapular, biceps, mid-abdominal and suprailiac skinfolds, to the nearest 0.1 mm) (Holtain skin fold Callipers, United Kingdom).

Systolic and diastolic blood pressure measurements were taken in a supine position, on the right arm, following 5 minutes rest, using DinaMap ProCare 100 oscillometric sphygmomanometer with appropriate cuff size. Furthermore, all age groups underwent a cardiovascular endurance test to estimate physical work capacity (PWC170) using a Monark cycle ergometer and the Australian Education Fitness Award test (AFEA) encompassing four physical activities: curl ups, shoulder stretches, sit and reach and basketball throws. Weekly pedometer logs were completed. A photocopy of both hands was taken, and finger length for the second and fourth digit, as well as 2^nd^ to 4^th^ digit ratio was recorded.

At the 14 and 17-year follow-up, the psychomotor skills of the participants were assessed by administering the standardised quantitative McCarron Assessment of Neuromuscular Development tool (MAND) [33].

### Age-specific assessments

#### 14-year follow-up (adolescents aged 13–15.9)

At the 14-year follow-up, the assessments of particular interest were: lung function, bronchial responsiveness and allergy profiles.

*Lung function and bronchial hyperresponsiveness test*. Lung function was assessed by base spirometry [34], using an electronic spirometer (KoKo Spirometer, nSpire Health Ltd. USA), to asses airway function by calculating standard spirometry variables, and to define the suitability of the participants to perform the bronchial hyperresponsiveness test. Bronchial hyperresponsiveness was assessed by administering increasing doses of Methacholine, with the forced expiratory volume (FEV1) measured within five minutes of administration, following the American Thoracic Society guidelines for Methacholine and Exercise Challenge Testing (KoKo Dosimeter, nSpire Health Ltd USA) [35]. Testing was continued until either a drop in FEV1 ≥20% from baseline assessment occurred, or maximum cumulative dose was reached (16 mg/ml). If a drop in FEV1 ≥20% occurred, 400 mcg of salbutamol was given via spacer device under medical supervision. The provocative concentration causing the 20% fall in FEV1 was calculated according to the following formula:

$$PC20=\frac{antilog\;[log\;C1+(log\;C2‐log\;C1)(20‐R1)]}{\left(R2‐R1\right)}$$

Where:

C1 = second last concentration (<20% FEV1 fall)

C2 = last concentration (>20% FEV1 fall)

R1 = % fall FEV1 after C1

R2 = % fall FEV1 after C2

*Skin prick testing for allergies*. Standard skin prick testing was completed using the following panel of allergens: egg white, 7 grass mix and grass pollen (perennial ryegrass), cat and dog hair, cow’s milk (whole), cockroach, fungus (Aspergillus fumigatus), mould (Alternaria alternata), and dust mite (Dermatophagoides farina and pteronyssinus), positive controls (histamine hydrochloride 10 mg/ml) and negative controls (glycerine/saline). In case of a reaction the size of the wheal and flare (mm) were recorded. Positive control size was recorded 10 minutes after administration, and size of allergens and negative control 15 minutes after administration. Participants were instructed to cease the use of antihistamines 24h prior to the administered test and were questioned to verify medication withholding on the day of testing. Exclusion criteria were as follows: severe reactions to respiratory irritants (smoke, fumes, perfumes etc.), diffuse dermatological conditions (the test was only performed on healthy skin) and inability to cease antihistamines.

In addition to the physical measurements, questions with emphasis on participants and familial asthma and allergy medical history, as well as a modified version of the standardised ‘International Studies of Asthma and Allergies in Childhood’ (ISAAC) questionnaire were completed by the primary caregiver.

*17-year follow-up (adolescents aged 16–18*.*9)*. At the 17-year follow-up cardiometabolic health and mental health were assessed.

*Abdominal ultrasonography*. Abdominal ultrasonography was conducted for investigation of the presence and severity of hepatic steatosis and measurement of abdominal adipose tissue (mm) (subcutaneous, visceral and pre-peritoneal), using a Siemens Antares ultrasound machine with a CH 6–2 curved array probe (Sequoia, Siemens Medical Solutions, USA). Trained sonographers performed focused liver ultrasonography in accordance with the protocol described by Hamaguchi et al., which provides 92% sensitivity and 100% specificity for the histological diagnosis of hepatic steatosis [36].

A single specialist radiologist, who was blinded to the clinical and laboratory characteristics of the subjects, interpreted the ultrasound images. Scores of 0 to 3, 0 to 2 and 0 to 1 were determined from captured images for liver echotexture (bright liver and hepatorenal echo contrast), deep attenuation (diaphragm visibility) and vessel blurring (intrahepatic vessel visibility). The diagnosis of hepatic steatosis required a total score of ≥ 2, inclusive of echotexture score of ≥1. The severity of hepatic steatosis was classified by the total fatty liver score as 0–1 (no fatty liver), 2–3 (mild fatty liver), or 4–6 (moderate to severe fatty liver). Adolescents with sonographic fatty liver, the absence of self-reported long-term use of medication affecting the liver, and a self-reported weekly alcohol intake of < 140 g for males and < 70 g for females over the previous year were classified as having non-alcoholic fatty liver disease.

Abdominal (subcutaneous, visceral and pre-peritoneal) adipose thickness measurements were performed with previously described criteria that correlate closely with compartmental adipose areas and cardiovascular and metabolic risk factors [37–39].

*Measures of blood pressure and arterial stiffness*. Pulse wave velocity (m/s) and heart rate corrected augmentation index (%), as measures of arterial stiffness, as well as systolic and diastolic blood pressure (mmHg) and resting heart rate (bpm) were measured using SphygmoCor assessment (AtCor Medical Pulse Wave Analysis System SCOR-Px, Australia) [40]. Systolic and diastolic blood pressure were measured by oscillometric sphygmomanometer (DINAMAP vital signs monitor 8100, DINAMAP XL vital signs monitor or DINAMAP ProCare 100), after resting 5 minutes and using the appropriate cuff size. Six readings were taken in a supine position, every 2 minutes for 10 minutes. The average value was calculated after excluding the first reading. After 5 minutes rest, the six blood pressure measurements were recorded, and three electrocardiogram leads attached to left leg, right arm and left arm. Tonometers were applied to two sites (the carotid artery and the distal dorsalis pedis). Distance measurement was recorded in millimetres between the manubrium sternum and the two sites. The pulse wave analysis was recorded from the supported radial artery with the wrist facing up. Data were entered after the waveform was maintained for 10 seconds and the test was repeated until at least two captures were recorded with a quality index of >80. Pulse wave velocity was calculated by dividing the distance between tonometers by the transit time of the arterial pulse wave. Heart rate corrected augmentation index was defined as the difference in the second and first systolic pressure peaks as a percentage of pulse pressure and was corrected for heart rate.

In addition to the physical measurements, the participant questionnaire included a set of questions with emphasis on familial cardiometabolic and cardiovascular medical history.

*Cognitive function*. Measures on a range of elements of cognitive performance were collected from a sensitive and reliable computerized battery of tests, using the digital cognitive testing system via the downloaded Cogstate Research™ software (CogState Limited, USA) (www.cogstate.com) [41,42]. The following tests were administered to each participant following adequate familiarization through practice sessions: The Groton Maze Learning Test, assessing the total number of errors made in attempting to learn the same hidden pathway on five consecutive trials at a single session; The Groton Maze Learning Test Recall, assessing the total number of errors made in remembering the maze pathway after a delay; Card Detection and Card Identification Tests, both measuring speed performance; One Card Learning Test, assessing accuracy of performance; Continuous Paired Associate Learning Test, assessing accurate memory patterns; total number of errors across the five rounds.

#### 20-year follow-up (young adults aged 20–22.9)

The 20-year follow-up investigated vision and body composition.

*Vision*. An accurate measurement of the refractive status of the participants’ eyes, as well as a measurement of the corneal curvature was attained using the NIDEK ARK-1 Auto ref/keratometer (NIDEK CO., LTD. Tokyo, Japan). On the day of the assessment, the participants were asked not to wear contact lenses or for them to be removed >20 minutes prior to the autorefraction test. If the participants wore glasses, they were asked to remove them right before testing. Trained research assistants operated the autorefractor according to manufacturer’s recommendation, and the following measurements were taken from both eyes: spherical refractive error, cylindrical refractive error, cylinder axis, pupillary distance, corneal curvature radius and axis angle in the steepest and flattest meridian direction.

In addition to the physical measurements, the participant questionnaire included a set of questions with emphasis on participants and familial eye-health medical history.

*Body composition*. The bone mineral content and body composition including body fat percentage and skeletal integrity was assessed from a dual energy X-ray absorptiometry scan (DEXA). DEXA is considered the preferred method for measuring bone mineral density and soft tissue composition [43]. Each participant completed one total body scan and standard measurements were recorded. The Norland XR-36 Quick scan machine (Norland Medical Systems, Inc., Fort Atkinson, WI, USA), that utilizes a very small dose (<10 microSieverts) ionising radiation was operated by a trained Research Assistants. Due to the use of ionising radiation, approval was obtained from the Radiation Safety Officer at the University of Western Australia.

## Findings to date

The clinical characteristics for the index pregnancy of the 303 included GUHS participants can be viewed in **Table 2** and participant characteristics per follow-up are presented in **Table 3**. The findings to date are summarised below according to investigated outcomes.

**Table 2:** Clinical characteristics of the GUHS cohort.

		Total N = 303
**Sex N (%)**		
	Male	146 (48.2%)
	Female	157 (51.8%)
**Type of ART N (%)**		
	IVF	128 (42.2%)
	ICSI	36 (11.9%)
	Frozen IVF	76 (25.1%)
	Frozen ICSI	26 (8.6%)
	GIFT	15 (5.0%)
	IUI	1 (0.3%)
	Unconfirmed type	21 (6.9%)
**Plurality N (%)**		
	Singletons	234 (77.2%)
	Twins	63 (20.8%)
	Triplets	6 (2.0%)
**Birthweight in grams**		
	Median (Q1-Q3)	3150 (2785–3535)
**Gestational age in weeks and days as fraction**		
	Median (Q1-Q3)	
		38.43 (36.86–39.57)
**Type of Frozen Embryo transfer N (%)**		
	Natural cycle	37 (36.3%)
	Ovulation Induction	23 (22.5%)
	Hormone replacement therapy	17 (16.7%)
	Unknown	25 (24.5%)
**Day of Embryo Transfer N (%)**		
	Day 0	1 (0.3%)
	Day 1	38 (12.5%)
	Day 2	186 (61.4%)
	Day 3	34 (11.2%)
	Missing	44 (14.5%)
**Donor Used N (%)**		
	None	296 (97.7%)
	Egg	4 (1.3%)
	Sperm	2 (0.7%)
	Embryo	1 (0.3%)
**Maternal ethnicity N (%)**		
	Caucasian	292 (96.4%)
	Asian	7 (2.3%)
	Indian	2 (0.7%)
	Missing	2 (0.7%)
**Maternal age at conception (in years)**		
	Mean ± SD	34.0 **±** 3.9
**Cause of Infertility N (%)** *		
	Female	126 (41.6%)
	Male	55 (18.2%)
	Both female and male	34 (11.2%)
	Unexplained	68 (22.4%)
	Missing	20 (6.6%)

IVF: In vitro fertilisation; ICSI: Intracytoplasmic sperm injection; GIFT: Gamete intrafallopian transfer; IUI: Intra uterine insemination.

*Female causes included tubal, endometrial, ovarian and sterility. Male causes included suboptimal sperm parameters and sterility.

**Table 3:** Demographic characteristics per follow-up.

	14 years N = 152	17 years N = 165	20 years N = 61
**Age at assessment** Mean ± SD	14.7 ± 0.9	17.1 ± 0.9	20.8 ± 0.9
**BMI** kg/m^2^ Median (Q1-Q3)	20.2 (18.5–22.7)	21.2 (19.7–23.2)	21.7 (19.7–25.2)
**Cigarette smokers** Yes N (%)	N/A	7 (2.3%)	5 (8.3%)
**SES** N (%) 1^st^ quintile 2^nd^ quintile 3^rd^ quintile 4^th^ quintile 5^th^ quintile	6 (4.0%) 22 (14.8%) 23 (15.4%) 25 (16.8%) 73 (49.0%)	3 (1.8%) 27 (16.5%) 27 (16.5%) 23 (14.0%) 84 (51.2%)	0 (0.0%) 11 (18.3%) 4 (6.7%) 8 (13.3%) 37 (61.7%)

BMI: Body Mass Index, N/A: Not applicable, SES: Socio-economic status [based on postcodes and is reported as advantage-disadvantage deciles based on the Socio-Economic Indexes for Areas (SEIFA) scores for Western Australia, from the Australian Bureau of Statistics (from lowest score of 1 to the highest score of 10) (Australian Bureau of Statistics 2006, 2011, 2016). For presentation, we transformed deciles into quintiles (from lowest score of 1 to highest score of 5). Categorical outcomes do not add up to the presented total due to the missing values.

### ART and potential epigenetic differences

The potential epigenetic differences between the cohorts were investigated using an epigenome-wide association study approach, and demonstrated no significant differences in the overall DNA methylation signatures between the cohorts (GUHS n = 231, the Raine Study n = 1188) [44]. Furthermore, no differentially methylated regions reached statistical significance, and no significant biological pathways were identified in the comparative gene set enrichment analysis.

However, differences in the DNA methylation signatures were identified when comparing IVF and ICSI conceived offspring, with a general trend towards hypomethylation in the ICSI offspring. A moderate to high correlation was observed between the chronological age of the GUHS participants and 4 estimates (Horvath, Hannum, PhenoAge and Levine) of DNA methylation age in whole blood.

Overall, despite minor perturbations in the DNA methylation signatures of ART newborns reported in the literature, in adolescence, we observed no significant differences in the DNA methylation profiles between the cohorts.

### Cardiometabolic profiles of adolescent conceived after ART

Cardiometabolic profiles were compared at the 17-year follow-up (GUHS n = 163, the Raine Study n = 1690) [45]. Analyses were carried out for the total sample, and separately for males (GUHS n = 81 vs. the Raine Study n = 842) and females (GUHS n = 82 vs. the Raine Study n = 848). It is important to note that all cardiometabolic outcomes fell within the normal range in both cohorts.

Overall, we report a trend toward lower body size in the GUHS cohort, with a lower BMI, thinner waist circumference and skinfolds. Serum fasting parameters were mostly comparable between the cohorts, apart from the following significant differences: lower triglycerides in females, increased high density lipoprotein (HDL-C), decreased cholesterol/HDL-C ratio and alanine aminotransaminase in males. No differences in the prevalence of non-alcoholic fatty liver disease were observed between the cohorts. Overall, the GUHS adolescents showed a trend towards less subcutaneous adipose tissue measured on ultrasound, and an increase in visceral and preperitoneal adipose tissue compared to the Raine Study adolescents. Measures of arterial stiffness were lower in the GUHS adolescents, and no differences in blood pressure and heart rate were reported.

In this study we did not detect an adverse effect of ART on most cardiometabolic parameters at adolescence, in contrast to some previous studies, focusing mainly on childhood. The detected increase in visceral adipose tissue requires further investigation.

### Thyroid function of offspring conceived after ART

Thyroid function was compared at the 14- and 20-year follow-up (GUHS n = 134, the Raine Study n = 1359, and GUHS n = 47, the Raine Study n = 914 respectively) [46]. At both follow-ups mean thyroid function fell within the normal range. Mean thyroid stimulation hormone did not significantly differ between the cohorts. At the 14-year follow-up mean free triiodothyronine (fT3) was significantly lower in the GUHS adolescents and mean free thyroxine (fT4) significantly increased. At the 20-year follow-up fT3 and fT4 were significantly increased in GUHS adolescents. The percentage of offspring with thyroid peroxidase antibodies >6 kU/L did not differ between the cohorts. No differences in the percentage of offspring classifying as having subclinical or overt hypo- and hyperthyroidism, or autoimmune thyroid disorder, were demonstrated. Thyroid function did not differ between fresh ET and FET and comparing ET and FET to the Raine Study separately, demonstrated similar results as when the entire GUHS cohort was compared to the Raine Study. No correlation between peri-ovulatory peak maternal estrogen levels and fT4 was demonstrated.

Our study does not support findings to date of altered thyroid function in offspring conceived after ART, and no differences between ET and FET were demonstrated. The statistically significant differences in fT3 and fT4 at both ages warrant reinvestigation in adulthood, although not clinically relevant when measured in this study in adolescence.

### Asthma and allergies

At age 14, n = 152 GUHS and n = 1845 Gen2 adolescents completed asthma and allergy assessments (provisionally accepted). No differences were detected in the prevalence of current asthma, while spirometry measured lung volumes were larger, and bronchial hyperresponsiveness was less prevalent in the ART cohort. Current allergic rhinoconjunctivitis, food allergies and having a positive SPT were more prevalent in the ART cohort. Current atopic dermatitis did not differ between the cohorts.

In this study of 14-year-old adolescents, we report no differences in the prevalence of asthma, slightly altered lung function, and a higher prevalence of all allergies assessed in the ART vs. non-ART cohort. This is in contrast with some previous studies that report an increase in asthma in offspring conceived with ART, but not in allergies. As those studies were mainly conducted in childhood, it is possible that ART offspring grow out of asthma with time. If ART conceived offspring are indeed at an increased risk of allergies, it is important for families and healthcare providers alike, to be aware of this. Further studies are required to confirm our findings.

## Strengths and limitations

The GUHS has various strengths and limitations. The major strength is that it is the first large-scale long-term follow up of adolescents and young adults conceived after ART. It is the first study, to collect data on such a wide scale of health-outcomes. In this field of research, the number of included offspring is quite substantial, particularly for a cohort assessing the health of offspring past childhood. This number was reached, by the attempt to include all offspring conceived after ART in the only two fertility clinics operating at the time, born over a 10-year period. By approaching everyone via fertility clinics and electoral roll, instead of approaching offspring who are already seeking healthcare, selection bias is reduced, which further improves representativeness of the cohort. A further major strength is the unique study design, where an ART conceived cohort replicated the exact same assessments previously completed by their counterparts conceived without ART. The replication of assessments in combination with the relatively static population of Western Australia, makes this design unique and difficult to replicate elsewhere. Lastly, one of the potential explanations for a higher risk of adverse health outcomes in ART-conceived offspring, is the higher prevalence of adverse obstetric outcomes following ART pregnancies. A strength of the GUHS is the collection of a large amount of obstetric data, which allows for the ability to adjust for such obstetric factors.

The study also has various limitations. Despite substantial overall cohort size, the sample size often does not allow for comparison of IVF and ICSI, as well as ET and FET, due to the medical practice at this time. Given the higher prevalence of congenital malformations in offspring conceived after ICSI, investigating the long-term health of these offspring, is of particular interest. In the present cohort, one third of offspring were conceived from a frozen cycle, which limited the ability of subgroup analyses of these offspring. Combining our cohort with others will allow for comparison of outcomes between FET and ET, as well as ICSI and IVF with greater power. Furthermore, information regarding couples’ causes of infertility was limited, and again numbers did not allow to compare outcomes for different causes of infertility. Additionally, some of our variables, particularly those regarding pregnancy, were collected retrospectively from fertility clinics and a state registry, which meant the available information was limited and contained missing data. A further limitation is the time between assessments of the GUHS and the Raine Study. To replicate assessments at the same age, GUHS assessments took place between 1 and 14 years after the Raine Study assessments. Although protocols and methodologies were identical, external factors may have changed which could have affected study results. Furthermore, since the participants of this study were conceived in the 1990’s IVF laboratory protocols have undergone considerable changes, particularly changes in embryo culturing, which may affect health outcomes. Lastly, 96.4% of mothers were of Caucasian decent, which reduces the applicability of our findings to other ethnicities. Given the observational nature of the study, causation cannot be proven in the GUHS. The role of various epigenetic mechanisms, potentially occurring in the early developmental period in ART pregnancies, needs to be further explored. The question whether potential differences in health outcomes are due the ART treatment itself, the underlying infertility and genetic make-up of a couple, or other factors such as perinatal and family factors, remains to be definitively addressed.

## Conclusion

In conclusion, the GUHS cohort provides a valuable addition to the limited knowledge of the long-term health outcomes of ART-conceived offspring. Although most of the findings to date are reassuring, replication in independent cohorts is of great importance. Additionally, if feasible, reinvestigation of the GUHS cohort as they transition into later adulthood would give valuable information about longer-term health outcomes. Lastly, the envisioned in-depth study of markers of reproductive function of the cohort would give some much-needed information on their ability to reproduce, which is of particular relevance.

## Collaboration

We have established a cohort with a wealth of data on long-term health outcomes in ART conceived offspring. The Growing Up Healthy Study welcomes opportunities for collaboration. Combining our data with other cohorts or databases will improve the much-needed knowledge about the long-term effects of these widely used techniques. In doing so, investigation of specific subgroup analyses such as ICSI vs IVF, FET vs ET, and the role of different causes of infertility and other underlying parental factors will be possible. Researchers are encouraged to contact the principal investigator Professor Roger Hart, to discuss collaboration (roger.hart@uwa.edu.au).

## Supporting information

S1 Table General blood biochemistry and age specific targeted panels. Measurements from full blood (full blood picture, differential white cell count and HbA1c), plasma (glucose, lipids, liver function test and iron studies), serum (remaining analytes) and urine.
(PDF)

S1 File Study consents. (PDF)

S2 File Questionnaires 14-year follow-up. (PDF)

S3 File Questionnaires 17-year follow-up. (PDF)

S4 File Questionnaires 20-year follow-up. (PDF)
