# Supplementary material for: Cohort profile: The Growing Up Healthy Study (GUHS)—A prospective and observational cohort study investigating the long-term health outcomes of offspring conceived after assisted reproductive technologies
Source: PLoS One. 2022 Jul 22;17(7):e0272064. doi: 10.1371/journal.pone.0272064 (PMC9307151; doi:10.1371/journal.pone.0272064)
Supplement: S1 Table — Measurements from full blood (full blood picture, differential white cell count and HbA1c), plasma (glucose, lipids, liver function test and iron studies), serum (remaining analytes) and urine. (PDF) [file pone.0272064.s001.pdf]

S5 Supplemental Table General blood biochemistry and age specific targeted panels

BLOOD BIOCHEMISTRY	13- year F/U	16-year F/U	20-year F/U
**HAEMATOLOGY – FULL BLOOD PICTURE**			
Haemoglobin (Hgb) [g/L]	+	+	+
Red cell count (RCC) [x10^12/L]	+	+	+
Haematocrit (Hct) [%]	+	+	+
Mean Corpuscular Volume (MCV) [fL]	+	+	+
Mean Corpuscular Haemoglobin (MCH) [pg]	+	+	+
Mean Corpuscular Haemoglobin Concentration (MCHC) [g/L]	+	+	+
Red cell distribution width (RDW)	+	+	+
White cell count (WCC) [x10^9/L]	+	+	+
**DIFFERENTIAL WHITE CELL COUNT (ABSOLUTE AND %)**			
Neutrophils [x10^9/L]	+	+	+
Lymphocytes [x10^9/L]	+	+	+
Monocytes [x10^9/L]	+	+	+
Eosinophils [x10^9/L]	+	+	+
Basophils [x10^9/L]	+	+	+
Thrombocyte count [x10^9/L]	+	+	+
Mean Platelet Volume [fL]	+	+	+
**GLUCOSE HOMEOSTASIS**			
Insulin [mU/L]	+	+	+
Glucose [mmol/L]	+	+	+
HbA1c [IFCC mmol/mol and NGSP %]	+	+	+
**LIPIDS AND CARDIAC PARAMETERS**			
Total cholesterol [mmol/L]	+	+	+
Low Density Level – cholesterol [mmol/L]	+	+	+
High Density Level – cholesterol [mmol/L]	+	+	+
Cholesterol/HDL ratio	+	+	+
Triglycerides [mmol/L]	+	+	+
High-sensitivity C-Reactive Protein (hsCRP) [mg/L]	+	+	+
**IRON STUDIES**			
Iron [umol/L]		+	+
Transferrin [umol/L]		+	+
% Transferrin saturation		+	+
Ferritin [ug/L]		+	+
**LIVER FUNCTION TEST**			
Alkaline phosphatase (ALP) [U/L]		+	+
Alkaline aminotransferase (ALT) [U/L]	+	+	+
Aspartate aminotransferase (AST) [U/L]		+	+
Gamma-glutamyl transferase (GGT) [U/L]	+	+	+
Total Bilirubin [umol/L]		+	+
Icterus index	+	+	+
Uric acid [mmol/L]	+		+
Total protein [g/L]		+	+
Albumin [g/L]	+		+
Globulins [g/L]			+
**THYROID FUNCTION TEST**			
Thyroid stimulating hormone (TSH) [mU/L]	+		+
Free thyroxin (fT4) [pmol/L]	+		+
Free triiodothyronine (fT3) [pmol/L]	+		+
Thyroid peroxidase antibodies (TPO) [kU/L]	+		+
**KIDNEY FUNCTION TEST**			
Estimated glomerular filtration rate (eGFR)	+		+
Sodium (Na) [mmol/L]	+		+
Potassium (K) [mmol/L]	+		+
Creatinine [umol/L]	+		+
**VITAMINS**			
25-hydroxyvitamin D (nmol/L)	+		+
Red blood cell fatty acids (%)	Erythrocyte pellets	Erythrocyte pellets	Erythrocyte pellets
**URINE ANALYSIS (AT 16 YR FOLLOW-UP)**	Na [mmol/L], K [mmol/L], Calcium (Ca) [mmol/L], Creatinine [mmol/L], microalbumin [mg/mmol], albumin [mg/L], albumin/creatinine and calcium/creatinine ratio		

Measurements from full blood (full blood picture, differential white cell count and HbA1c), plasma (glucose, lipids, liver function test and iron studies), serum (remaining analytes) and urine.

Blood sample and urine analysis

Following informed consent, fasting venous blood samples were taken by a trained phlebotomist, in three different collection tubes containing the appropriate preserving agent for the specific measured analytes.

Blood for general biochemistry (full blood picture including differential white cell count) and glycated haemoglobin (HbA1c) was collected in a 4ml Ethylenediaminetetraacetic acid (EDTA) spray coated tube*, and samples were delivered to the PathWest Laboratory in Perth, Western Australia, Australia, within two hours of phlebotomy for immediate analysis. Whole blood for DNA extraction was collected from the participants at a single time point, commonly at their first assessment. The whole blood was archived in -80^{0}C freezers until genomic DNA was extracted.

Plasma and serum samples were obtained from bloods collected in the appropriate collection tubes, K2EDTA / Fluoride** and serum separating tubes*** respectively, following a standardised laboratory protocol. Six aliquots of both plasma and serum samples, were frozen within the designated 2 hours from blood collection. The following analyses were performed: glucose homeostasis, including a calculation of the homeostatic model assessment for insulin resistance (HOMA-IR) [Fasting insulin (mU/L) X (fasting glucose (mmol/L)/22.5)][28]; thyroid, kidney and liver function tests; iron studies; lipids and cardiac measurements, Vitamin D, as well as plasma and urine spot analyses for kidney function.

Erythrocyte fatty acids were analysed by gas-liquid chromatography (Agilent Technologies Model 7980A Gas Chromatograph, Agilent Technologies, Santa Clara, CA, USA), using an SPTH-2560 column (100m x 0.25mm, 0.2um film thickness; Supelco, Bellefonte, PA, USA), with the temperature programmed from 140°C to 240°C at 4°C/min, and using hydrogen as carrier gas (1ml/min) at a split ratio of 30:1. Individual fatty acids were identified by their retention time relative to a standard mixture of fatty acids, and calculated as a relative percentage of the peak total area (set at 100%).

The remaining aliquots of serum, plasma and urine samples have been appropriately archived at -80^{0}C for long term storage.

Collection tubes used: *(BD Vacutainer™ K2 EDTA Tube, Lavander BD Hemogard Closure);

**Fluoride - BD Vacutainer™ Plastic Blood Collection Tubes with Fluoride; Gray Hemogard Closure, 4ml;

***BD Vacutainer™ Venous Blood Collection Tubes: SST™ Serum Separation Tubes: Hemogard, 8.5ml.
