# Supplementary material for: Cohort profile: The Growing Up Healthy Study (GUHS)—A prospective and observational cohort study investigating the long-term health outcomes of offspring conceived after assisted reproductive technologies
Source: PLoS One. 2022 Jul 22;17(7):e0272064. doi: 10.1371/journal.pone.0272064 (PMC9307151; doi:10.1371/journal.pone.0272064)
Supplement: S1 File — (PDF) [file pone.0272064.s002.pdf]

GROWING UP HEALTHY STUDY (RA/4/1/5860) - CONSENT FORM

**Please note that we are looking to involve children born from <u>IVF treatment only</u>. Please confirm the method by which your child was conceived and return this form to us in the reply paid envelope provided:*

☐ IVF ☐ IUI ☐ ovulation induction ☐ tracking with timed intercourse

I, we ....

Date of birth (mother) ....

Together with my child/children ....

Date of Birth/Gender (child/children) ....

- Am/Are interested in participating in the *Growing Up Healthy Study*.
- I/we agree to being contacted by a research co-ordinator in this regard.
- I/we agree to the release of information from my/our medical records held at the IVF clinic.
- I/we understand I can withdraw my consent at any time.

Signatures

Mother ....

Father (partner) ....

Date ....

Contact information

Mobile phone ....

Daytime (work) phone ....

Evening (home) phone ....

Email address ....

Growing Up Healthy Study
University of Western Australia School of Women's and Infants' Health (M550)
35 Stirling Highway CRAWLEY WA 6009
T: +61 8 9340 1443 M: 0439 266 434 Email: guhstudy-swih@uwa.edu.au

---

The Human Research Ethics Committee at the University of Western Australia requires that all participants are informed that, if they have any complaint regarding the manner, in which a research project is conducted, it may be given to the researcher or, alternatively to the Secretary, Human Research Ethics Committee, Registrar's Office, University of Western Australia, 35 Stirling Highway, Crawley, WA 6009 (telephone number 6488-3703). Please refer to the Information Sheet for a list of contact numbers of Chief Investigators should you wish to discuss any aspects of this study.

THE UNIVERSITY OF WESTERN AUSTRALIA

women & infants research foundation Western Australia

GROWING UP HEALTHY STUDY 13-15

ID ☐☐☐☐☐

Parent/Participant Consent Form

(For participants <18 years of age)

I,……………………………………………………………………………… have read the information about my child participating in the age 13-15 follow-up, genetic studies and data linkage in relation to the *Growing Up Healthy Study* and all of my questions have been answered.

I agree to my child …………………………………………………………….participating in the following parts of the *Growing Up Healthy Study*.

	YES	NO
Participant Questionnaires	☐	☐
Physical Measurements (i.e. height, weight, blood pressure, hand photocopy)	☐	☐
Bronchial Responsiveness Assessment	☐	☐
Skin Prick Allergy Test	☐	☐
Motor Control Assessment	☐	☐
Cardiovascular Endurance Test	☐	☐
Australian Fitness Education Award	☐	☐
Pedometer Physical Activity Assessment (completed at home)	☐	☐

I understand/agree that:

- My child does not have to participate in all parts of this study if they choose not to and they can withdraw from any part of the study at any time, without explanation.

- All information provided will be de-identified (all names, addresses etc. removed), treated as strictly confidential and will not be released by the investigators without my permission. The only exception to this principle of confidentiality is if documents are required by law.

- I have the right to change my mind and withdraw permission to the use and storage of my child's blood sample and the permission to use information at any time, without explanation.

- Neither I nor my child will receive any personal financial benefit from any commercial tests or products that may be developed from the study.

- Research data gathered for the study may be sent overseas for research purposes.

- Research data gathered for the study may be published and we understand that no identifying information (e.g. individual results, names, dates of birth, addresses) will be used.

- I agree that publically held information accessible through the data linkage unit at the Department of Health, Western Australia, can be sourced on my child.

- I will be given a copy of this consent form and information sheet for my personal records.

Parent/Participant Consent for Biological Samples

I agree:

	YES	NO
My child can provide a blood sample (via venepuncture)	☐	☐
My child can provide a urine sample	☐	☐
My child's blood and urine samples can be stored	☐	☐

I agree:

	YES	NO
My child's DNA can be extracted from the blood sample	☐	☐
My child's DNA sample can be stored	☐	☐

I understand/agree that my child's DNA sample can be used for genetic studies as below:

- DNA can be used for genetic analysis of risk factors associated with the development of childhood and adult health and disease.
- DNA will not be used to diagnose disease.
- DNA can be stored for future research and analysis.
- DNA sample and data will be discarded at any time upon my written request.
- My child's de-identified DNA sample may be analysed outside of Australia as part of international collaborations studying the genetic/epigenetic basis of health and disease.
- My child's DNA and any results of the DNA analysis will not be revealed or made available to any other person or organisation except in circumstances where disclosure is required by law.

……………………………………………… ………………………………

Signature ***Date***

Approval to conduct this research has been provided by the University of Western Australia, in accordance with its ethics review and approval procedures. Any person considering participation in this research project, or agreeing to participate, may raise any questions or issues with the researchers at any time. In addition, any person not satisfied with the response of researchers may raise ethics issues or concerns, and may make any complaints about this research project by contacting the Human Research Ethics Office at the University of Western Australia on (08) 6488 3703 or by emailing to hreo-research@uwa.edu.au. All research participants are entitles to retain a copy of any Participant Information For and/or Participant Consent Form relating to this research project. Please refer to the Information Sheet for a list of contact numbers of Chief Investigators should you wish to discuss any aspects of this study.

THE UNIVERSITY OF WESTERN AUSTRALIA

women & infants research foundation Western Australia

GROWING UP HEALTHY STUDY 13-15

ID ☐☐☐☐☐

Participant Consent Form

I,………………………………………………………………………… have read the information about participating in the age 13-15 follow-up, genetic studies and data linkage in relation to the *Growing Up Healthy Study* and all of my questions have been answered.

I agree to participate in the following parts of the *Growing Up Healthy Study*.

	YES	NO
Participant Questionnaires	☐	☐
Physical Measurements (i.e. height, weight, blood pressure, hand photocopy)	☐	☐
Bronchial Responsiveness Assessment	☐	☐
Skin Prick Allergy Test	☐	☐
Motor Control Assessment	☐	☐
Cardiovascular Endurance Test	☐	☐
Australian Fitness Education Award	☐	☐
Pedometer Physical Activity Assessment (completed at home)	☐	☐

I understand/agree that:

- I don't have to participate in all parts of this study if I choose not to and I can withdraw from any part of the study at any time, without explanation.
- All information provided will be de-identified (all names, addresses etc. removed), treated as strictly confidential and will not be released by the investigators without my permission. The only exception to this principle of confidentiality is if documents are required by law.
- I have the right to change my mind and withdraw my permission to the use and storage of my blood sample and the permission to use information at any time, without explanation.
- I will not receive any personal financial benefit from any commercial tests or products that may be developed from the study.
- Research data gathered for the study may be sent overseas for research purposes.
- Research data gathered for the study may be published and I understand that no identifying information (e.g. individual results, names, dates of birth, addresses) will be used.
- I agree that publically held information accessible through the data linkage unit at the Department of Health, Western Australia, can be sourced on me.
- I will be given a copy of this consent form and information sheet for my personal records.

Participant Consent for Biological Samples

I agree:

	YES	NO
To provide a blood sample (via venepuncture)	☐	☐
To provide a urine sample	☐	☐
My blood and urine samples can be stored	☐	☐

I agree:

	YES	NO
DNA can be extracted from the blood sample	☐	☐
My DNA sample can be stored	☐	☐

I understand/agree that my DNA sample can be used for genetic studies as below:

- DNA can be used for genetic analysis of risk factors associated with the development of childhood and adult health and disease.
- DNA will not be used to diagnose disease.
- DNA can be stored for future research and analysis.
- DNA sample and data will be discarded at any time upon my written request.
- My de-identified DNA sample may be analysed outside of Australia as part of international collaborations studying the genetic/epigenetic basis of health and disease.
- My DNA and any results of the DNA analysis will not be revealed or made available to any other person or organisation except in circumstances where disclosure is required by law.

....

Signature ***Date***

Approval to conduct this research has been provided by the University of Western Australia, in accordance with its ethics review and approval procedures. Any person considering participation in this research project, or agreeing to participate, may raise any questions or issues with the researchers at any time. In addition, any person not satisfied with the response of researchers may raise ethics issues or concerns, and may make any complaints about this research project by contacting the Human Research Ethics Office at the University of Western Australia on (08) 6488 3703 or by emailing to hreo-research@uwa.edu.au. All research participants are entitles to retain a copy of any Participant Information For and/or Participant Consent Form relating to this research project. Please refer to the Information Sheet for a list of contact numbers of Chief Investigators should you wish to discuss any aspects of this study.

THE UNIVERSITY OF WESTERN AUSTRALIA

women & infants research foundation Western Australia

GROWING UP HEALTHY STUDY 16-18

ID □□□□□

Parent/Participant Consent Form

(For participants <18 years of age)

I,…………………………………………………………………………… have read the information about my child participating in the age 16-18 follow-up, genetic studies and data linkage in relation to the *Growing Up Healthy Study* and all of my questions have been answered.

I agree to my child ………………………………………………………….participating in the following parts of the *Growing Up Healthy Study*.

	YES	NO
Participant Questionnaires	☐	☐
Physical Measurements (i.e. height, weight, blood pressure, hand photocopy)	☐	☐
SphygmoCor Pulse Wave Examination	☐	☐
Cognitive Function Test	☐	☐
Motor Control Assessment	☐	☐
Cardiovascular Endurance Test	☐	☐
Australian Fitness Education Award	☐	☐
Pedometer Physical Activity Assessment (completed at home)	☐	☐
Liver Ultrasound Examination (completed at King Edward Memorial Hospital)	☐	☐

I understand/agree that:

- My child does not have to participate in all parts of this study if they choose not to and they can withdraw from any part of the study at any time, without explanation.
- All information provided will be de-identified (all names, addresses etc. removed), treated as strictly confidential and will not be released by the investigators without my permission. The only exception to this principle of confidentiality is if documents are required by law.
- I have the right to change my mind and withdraw permission to the use and storage of my child’s blood sample and the permission to use information at any time, without explanation.
- Neither I nor my child will receive any personal financial benefit from any commercial tests or products that may be developed from the study.
- Research data gathered for the study may be sent overseas for research purposes.
- Research data gathered for the study may be published and we understand that no identifying information (e.g. individual results, names, dates of birth, addresses) will be used.
- I agree that publically held information accessible through the data linkage unit at the Department of Health, WA, can be sourced on my child.
- I will be given a copy of this consent form and information sheet for my personal records.

Parent/Participant Consent for Biological Samples

I agree:

	YES	NO
My child can provide a blood sample (via venepuncture)	☐	☐
My child can provide a urine sample	☐	☐
My child's blood and urine samples can be stored	☐	☐

☐ This section is not applicable (informed consent for DNA previously obtained).

I agree:

	YES	NO
My child's DNA can be extracted from the blood sample	☐	☐
My child's DNA sample can be stored	☐	☐

I understand/agree that my child's DNA sample can be used for genetic studies as below:

- DNA can be used for genetic analysis of risk factors associated with the development of childhood and adult health and disease.
- DNA will not be used to diagnose disease.
- DNA can be stored for future research and analysis.
- DNA sample and data will be discarded at any time upon my written request.
- My child's de-identified DNA sample may be analysed outside of Australia as part of international collaborations studying the genetic/epigenetic basis of health and disease.
- My child's DNA and any results of the DNA analysis will not be revealed or made available to any other person or organisation except in circumstances where disclosure is required by law.

……………………………………………… ……………………………………

Signature *Date*

Approval to conduct this research has been provided by the University of Western Australia, in accordance with its ethics review and approval procedures. Any person considering participation in this research project, or agreeing to participate, may raise any questions or issues with the researchers at any time. In addition, any person not satisfied with the response of researchers may raise ethics issues or concerns, and may make any complaints about this research project by contacting the Human Research Ethics Office at the University of Western Australia on (08) 6488 3703 or by emailing to hreo-research@uwa.edu.au. All research participants are entitles to retain a copy of any Participant Information For and/or Participant Consent Form relating to this research project. Please refer to the Information Sheet for a list of contact numbers of Chief Investigators should you wish to discuss any aspects of this study.

GROWING UP HEALTHY STUDY 16-18

ID ☐☐☐☐☐

Participant Consent Form

I,……………………………………………………………………………… have read the information about participating in the age 16-18 follow-up, genetic studies and data linkage in relation to the *Growing Up Healthy Study* and all of my questions have been answered.

I agree to participate in the following parts of the *Growing Up Healthy Study*.

	YES	NO
Participant Questionnaires	☐	☐
Physical Measurements (i.e. height, weight, blood pressure, hand photocopy)	☐	☐
SphygmoCor Pulse Wave Examination	☐	☐
Cognitive Function Test	☐	☐
Motor Control Assessment	☐	☐
Cardiovascular Endurance Test	☐	☐
Australian Fitness Education Award	☐	☐
Pedometer Physical Activity Assessment (completed at home)	☐	☐
Liver Ultrasound Examination (completed at King Edward Memorial Hospital)	☐	☐

I understand/agree that:

- I don't have to participate in all parts of this study if I choose not to and I can withdraw from any part of the study at any time, without explanation.
- All information provided will be de-identified (all names, addresses etc. removed), treated as strictly confidential and will not be released by the investigators without my permission. The only exception to this principle of confidentiality is if documents are required by law.
- I have the right to change my mind and withdraw my permission to the use and storage of my blood sample and the permission to use information at any time, without explanation.
- I will not receive any personal financial benefit from any commercial tests or products that may be developed from the study.
- Research data gathered for the study may be sent overseas for research purposes.
- Research data gathered for the study may be published and I understand that no identifying information (e.g. individual results, names, dates of birth, addresses) will be used.
- I agree that publically held information accessible through the data linkage unit at the Department of Health, WA, can be sourced on me.
- I will be given a copy of this consent form and information sheet for my personal records.

Participant Consent for Biological Samples

I agree:

	YES	NO
To provide a blood sample (via venepuncture)	☐	☐
To provide a urine sample	☐	☐
My blood and urine samples can be stored	☐	☐

☐ This section is not applicable (informed consent for DNA previously obtained).

I agree:

	YES	NO
DNA can be extracted from the blood sample	☐	☐
My DNA sample can be stored	☐	☐

I understand/agree that my DNA sample can be used for genetic studies as below:

- DNA can be used for genetic analysis of risk factors associated with the development of childhood and adult health and disease.
- DNA will not be used to diagnose disease.
- DNA can be stored for future research and analysis.
- DNA sample and data will be discarded at any time upon my written request.
- My de-identified DNA sample may be analysed outside of Australia as part of international collaborations studying the genetic/epigenetic basis of health and disease.
- My DNA and any results of the DNA analysis will not be revealed or made available to any other person or organisation except in circumstances where disclosure is required by law.

………………………………………………… ……………………………………
Signature ***Date***

Approval to conduct this research has been provided by the University of Western Australia, in accordance with its ethics review and approval procedures. Any person considering participation in this research project, or agreeing to participate, may raise any questions or issues with the researchers at any time. In addition, any person not satisfied with the response of researchers may raise ethics issues or concerns, and may make any complaints about this research project by contacting the Human Research Ethics Office at the University of Western Australia on (08) 6488 3703 or by emailing to hreo-research@uwa.edu.au. All research participants are entitles to retain a copy of any Participant Information For and/or Participant Consent Form relating to this research project. Please refer to the Information Sheet for a list of contact numbers of Chief Investigators should you wish to discuss any aspects of this study.

GROWING UP HEALTHY STUDY 20-22

ID ☐☐☐☐☐

Participant Consent Form

I,………………………………………………………………………………… have read the information about participating in the age 20-22 follow-up, genetic studies and data linkage in relation to the Growing Up Healthy Study and all of my questions have been answered.

I agree to participate in the following parts of the *Growing Up Healthy Study*.

	YES	NO
Participant Questionnaires	☐	☐
Physical Measurements (i.e. height, weight, blood pressure, hand photocopy)	☐	☐
Clinical Eye Examination	☐	☐
Dual Energy X-Ray Absorptiometry (DEXA) Body Scan	☐	☐

I understand/agree that:

- I don't have to participate in all parts of this study if I choose not to and I can withdraw from any part of the study at any time, without explanation.
- All information provided will be de-identified (all names, addresses etc. removed), treated as strictly confidential and will not be released by the investigators without my permission. The only exception to this principle of confidentiality is if documents are required by law.
- I have the right to change my mind and withdraw my permission to the use and storage of my blood sample and the permission to use information at any time, without explanation.
- I will not receive any personal financial benefit from any commercial tests or products that may be developed from the study.
- Research data gathered for the study may be sent overseas for research purposes.
- Research data gathered for the study may be published and I understand that no identifying information (e.g. individual results, names, dates of birth, addresses) will be used.
- I agree that publically held information accessible through the data linkage unit at the Department of Health, Western Australia, can be sourced on me.
- I will be given a copy of this consent form and information sheet for my personal records.

Participant Consent for Biological Samples

I agree:

	YES	NO
To provide a blood sample (via venepuncture)	☐	☐
To provide a urine sample	☐	☐
My blood and urine samples can be stored	☐	☐

☐ This section is not applicable (informed consent for DNA previously obtained).

I agree:

	YES	NO
DNA can be extracted from the blood sample	☐	☐
My DNA sample can be stored	☐	☐

I understand/agree that my DNA sample can be used for genetic studies as below:

- DNA can be used for genetic analysis of risk factors associated with the development of childhood and adult health and disease.
- DNA will not be used to diagnose disease.
- DNA can be stored for future research and analysis.
- DNA sample and data will be discarded at any time upon my written request.
- My de-identified DNA sample may be analysed outside of Australia as part of international collaborations studying the genetic/epigenetic basis of health and disease.
- My DNA and any results of the DNA analysis will not be revealed or made available to any other person or organisation except in circumstances where disclosure is required by law.

……………………………………………… ………………………………

Signature ***Date***

Approval to conduct this research has been provided by the University of Western Australia, in accordance with its ethics review and approval procedures. Any person considering participation in this research project, or agreeing to participate, may raise any questions or issues with the researchers at any time. In addition, any person not satisfied with the response of researchers may raise ethics issues or concerns, and may make any complaints about this research project by contacting the Human Research Ethics Office at the University of Western Australia on (08) 6488 3703 or by emailing to hreo-research@uwa.edu.au. All research participants are entitles to retain a copy of any Participant Information For and/or Participant Consent Form relating to this research project. Please refer to the Information Sheet for a list of contact numbers of Chief Investigators should you wish to discuss any aspects of this study.
