# Supplementary material for: Cohort profile: The Growing Up Healthy Study (GUHS)—A prospective and observational cohort study investigating the long-term health outcomes of offspring conceived after assisted reproductive technologies
Source: PLoS One. 2022 Jul 22;17(7):e0272064. doi: 10.1371/journal.pone.0272064 (PMC9307151; doi:10.1371/journal.pone.0272064)
Supplement: S2 File — (PDF) [file pone.0272064.s003.pdf]

OFFICE USE ONLY

RA-CH ☐☐ RA-CO ☐☐ RA1-E ☐☐ RA2-E ☐☐

ID ☐☐☐☐

GROWING UP HEALTHY STUDY

Primary Caregiver Questionnaire

Part A

13-15

Thank you for giving your time to fill in this questionnaire

Please read each question carefully and answer all questions from ***Part A*** and ***Part B***.
Write your answers clearly in the space provided or mark the most appropriate response

☒

All information will be strictly confidential

Please take your time in answering all of the questions

If you require assistance to answer any of the questions please contact the Study Coordinator:

Blagica & Tina
T: +61 6458 1443
M: 0439 266 434
Email: guhstudy-swih@uwa.edu.au

The purpose of this questionnaire is to obtain information about your teenager's home life, leisure activities, schooling, behaviour and general health.

In answering this questionnaire the **Primary Caregiver** is the person who provides the majority of pastoral care for the study teenager(s).

The primary caregiver answering this questionnaire is the teenagers'…

- ☐ Biological mother
- ☐ Biological father
- ☐ Non-biological mother
- ☐ Non-biological father
- ☐ Other person *Please specify:* ....

If you are able to answer questions about your teenagers' other biological parent please do so.

Please complete this questionnaire independently
(without discussing it with your teenagers)

If you are coming in for an appointment, please bring your completed questionnaire with you on the day.

If you are unable to attend an appointment, please use the Reply Paid envelope enclosed to return your completed questionnaire.

If possible, could you please return your completed questionnaire to us by:

☐☐ / ☐☐ / ☐☐☐☐

SECTION 1 Housing and Family - Strictly Confidential

The following questions regarding your residence, past education and current job provide us with useful information about your teenager's home environment at the time of birth and now:

Q1 What is your current residential postcode? ☐☐☐☐

Q2 How many adults and children live in your home?
Please include your study teenager(s) and yourself. Children less than one year of age: Age = 0

First name	Age (years)	Sex (M/F)	Relationship to study teenager
e.g. Elizabeth<br>David<br>Jessica<br>Hannah	42<br>35<br>13<br>2	F<br>M<br>F<br>F	Mother<br>Stepfather<br>Study teenager<br>Stepsister
1			
2			
3			
4			
5			
6			
7			
8			
9			
10			

Q3 Does your study teenager have any other brothers or sisters not living at home?
Please include your study teenager(s) here if they **do not** *live at home*

☐ No → **Go to Q4**
☐ Yes

↓

First name	Age (years)	Sex (M/F)	Relationship to study teenager

Q4 How old were you when you left school? ☐☐

Q5 What was the last year of school that you completed? ☐☐
(e.g. year 10) or equivalent

Q6 Since leaving school have you completed any further education?

- ☐ None
- ☐ Trade certificate, or apprenticeship
- ☐ Professional registration (non-degree e.g. Police)
- ☐ College Diploma or Degree (e.g. TAFE, WAIT, WACAE)
- ☐ University Degree
- ☐ Other *Please specify:*……………………………………………………….

Q7 Do you currently have a full-time or part-time job of any kind (excluding home duties)?
Please mark only **one** *response- the main job*

- ☐ No, do not have a job - not seeking work → **Go to Q9**
- ☐ No, do not have a job - actively seeking work → **Go to Q9**
- ☐ Yes, do work for payment or profit
- ☐ Yes, do unpaid work in a family business
- ☐ Yes, do other unpaid work

Q8 Describe your current main job.
Please give details of job and description of work

Job title:
Job Description:

Q9 What do you currently spend most of your time doing?
Please mark only **one** *response, unless two or more responses apply equally*

- ☐ Full-time or part-time job (salary or own business)
- ☐ Voluntary work
- ☐ Looking for work
- ☐ Home duties / caring for children
- ☐ Studying
- ☐ Voluntarily out of the workforce
- ☐ Recovering from injury / illness
- ☐ Caring for an aged / disabled / ill person (friend or relative)
- ☐ Other *Please specify:*…………………………………………………

Q10 Does your partner currently have a full-time or part-time job of any kind (excluding home duties)? *Please mark only* **one** *response - the main job*

- ☐ No Partner → **Go to Section 2**
- ☐ No, does not have a job - not seeking work → **Go to Section 2**
- ☐ No, does not have a job - actively seeking work → **Go to Section 2**
- ☐ Yes, works for payment or profit
- ☐ Yes, does unpaid work in a family business
- ☐ Yes, does other unpaid work

Q11 Describe your partner's current main job.
Please give details of job and description of work

Job title:
Job Description:

SECTION 2 Your Health and Wellbeing - Strictly Confidential

The following questions are about the health and wellbeing of the study teenager's mother and father. We are also interested to know about the health and wellbeing of your partner if the mother/father of your teenager(s) is no longer living with you. We have tried to keep these to a minimum but some things that affect parents may also affect their children:

Q12 Do you currently smoke cigarettes?

- ☐ No → **Go to Q15**
- ☐ Yes

Q13 How many cigarettes do you smoke per day?
Please mark only **one** *response*

- ☐ Less than one
- ☐ 1 - 5
- ☐ 6 - 10
- ☐ 11 - 15
- ☐ 16 - 20
- ☐ More than 20

Q14 Do you smoke inside your house?

- ☐ No
- ☐ Yes

OFFICE USE ONLY

Q8 ☐☐ Q11 ☐☐

Q15 Does anyone else in your house currently smoke cigarettes?

- ☐ No → **Go to Q18**
- ☐ Yes

Q16 How many do they smoke per day **now**?
If more than one person at home smokes, please mark the **total** *number of cigarettes smoked*

- ☐ Less than one
- ☐ 1 - 5
- ☐ 6 - 10
- ☐ 11 - 15
- ☐ 16 - 20
- ☐ More than 20

Q17 Do they smoke inside your house?

- ☐ No
- ☐ Yes

Q18 Does anyone in your home smoke/use any other substances?
Please include pipe, cigars, marijuana, other drugs, etc.

- ☐ No → **Go to Q19**
- ☐ Yes

Which other substances do they smoke/use?

Please mark **one** *response for each item*	**No**	**Yes, once a week or less**	**Yes, more than once weekly but not everyday**	**Yes, everyday**
Pipe	☐	☐	☐	☐
Cigars	☐	☐	☐	☐
Marijuana	☐	☐	☐	☐
Other<br>*Please specify:*……………………………………	☐	☐	☐	☐

SECTION 3 Your Physical Activity and Health - Strictly Confidential

The following questions relate to your usual leisure-time physical activity habits:

Q19 During a typical 7 day period (a week), how many times on average do you do the following ***kinds of exercise*** for more than 15 minutes during your free time.

Please write in each box the appropriate number Times per week

a. Strenuous exercise (heart beats rapidly) ☐☐

(e.g., running, jogging, hockey, football, soccer, squash, basketball, judo, roller skating, vigorous swimming, vigorous long distance bicycling)

b. Moderate exercise (not exhausting) ☐☐

(e.g., fast walking, baseball, tennis, easy bicycling, volleyball, badminton, easy swimming, dancing)

c. Mild exercise (minimal effort) ☐☐

(e.g., yoga, archery, fishing from land, bowling, golf, easy walking)

Q20 During a typical 7 day period (a week), in your leisure time, how often do you engage in ***any regular activity*** long enough to work up a sweat (heart beats rapidly)?

- ☐ Often
- ☐ Sometimes
- ☐ Never/Rarely

Q21 Are you worried about your weight?

- ☐ Not at all
- ☐ A little
- ☐ Moderately
- ☐ Very

Q22 Do you consider yourself to be…

- ☐ Underweight?
- ☐ Normal weight?
- ☐ A bit overweight?
- ☐ Very overweight?

The following series of questions relate to your levels of depression, anxiety and stress and provide us with valuable information about your emotional wellbeing:

Q23 Please read each statement and mark the response which indicates how much the statement applied to you over the past week. There are no right or wrong answers. Do not spend too much time on any one statement:

The rating scale is as follows:

0 = Did not apply to me at all - Never
1 = Applied to me to some degree, or some of the time - Sometimes
2 = Applied to me a considerable degree, or a good part of the time - Often
3 = Applied to me very much, or most of the time – Almost always

Please mark **one** *response for each item*	**0**	**1**	**2**	**3**
**a.** I found it hard to wind down	☐	☐	☐	☐
**b.** I was aware of dryness of my mouth	☐	☐	☐	☐
**c.** I couldn't seem to experience any positive feeling at all	☐	☐	☐	☐
**d.** I experienced breathing difficulty (e.g., excessively rapid breathing, breathlessness in the absence of physical exertion)	☐	☐	☐	☐
**e.** I found it difficult to work up the initiative to do things	☐	☐	☐	☐
**f.** I tended to over-react to situations	☐	☐	☐	☐
**g.** I experienced trembling (e.g., in the hands)	☐	☐	☐	☐
**h.** I felt that I was using a lot of nervous energy	☐	☐	☐	☐
**i.** I was worried about situations in which I might panic and make a fool of myself	☐	☐	☐	☐
**j.** I felt that I had nothing to look forward to	☐	☐	☐	☐
**k.** I found myself getting agitated	☐	☐	☐	☐
**l.** I found it difficult to relax	☐	☐	☐	☐
**m.** I felt down-hearted and blue	☐	☐	☐	☐
**n.** I was intolerant of anything that kept me from getting on with what I was doing	☐	☐	☐	☐
**o.** I felt I was close to panic	☐	☐	☐	☐
**p.** I was unable to become enthusiastic about anything	☐	☐	☐	☐
**q.** I felt I wasn't worth much as a person	☐	☐	☐	☐
**r.** I felt that I was rather touchy	☐	☐	☐	☐
**s.** I was aware of the action of my heart in the absence of physical exertion (e.g., sense of heart rate increase, heart missing a beat)	☐	☐	☐	☐
**t.** I felt scared without any good reason	☐	☐	☐	☐
**u.** I felt that life was meaningless	☐	☐	☐	☐

Q24 Does this reflect a typical a week for you?

☐ Yes → **Go to Q25**
☐ No

Please describe the reason(s) for this not being a typical a week for you:

Q25 Have any of the following happened to you in the past year?

Please mark **Yes** *or* **No** *for each item*	**Yes**	**No**
Pregnancy problems	☐	☐
Death of a close relative *Which relative?...*	☐	☐
Death of a close friend	☐	☐
Separation or divorce	☐	☐
Marital problems	☐	☐
Problems with your children	☐	☐
Your own job loss (not voluntary)	☐	☐
Your partner's job loss (not voluntary)	☐	☐
Money problems	☐	☐
Residential move	☐	☐
Other *Please describe: ....*	☐	☐

This is called the Family Assessment Device. It was developed to give an idea of how families work together.

Q26 Below are statements about families and family relationships. Mark the statement which best describes your family (the people living in your house):

Please mark **one** *response for each item*	**Strongly Agree**	**Agree**	**Disagree**	**Strongly Disagree**
**a.** Planning family activities is difficult because we misunderstand each other	☐	☐	☐	☐
**b.** In times of crisis we can turn to each other for support	☐	☐	☐	☐
**c.** We cannot talk to each other about sadness we feel	☐	☐	☐	☐
**d.** Individuals (in the family) are accepted for what they are	☐	☐	☐	☐
**e.** We avoid discussing our fears and concerns	☐	☐	☐	☐
**f.** We express feelings to each other	☐	☐	☐	☐
**g.** There are lots of bad feelings in our family	☐	☐	☐	☐
**h.** We feel accepted for what we are	☐	☐	☐	☐
**i.** Making decisions is a problem in our family	☐	☐	☐	☐
**j.** We are able to make decisions about how to solve problems	☐	☐	☐	☐
**k.** We don't get on well together	☐	☐	☐	☐
**l.** We confide in each other	☐	☐	☐	☐
**m.** Drinking is a source of tension or disagreement in our family	☐	☐	☐	☐

SECTION 4 Housing Environment

Q27 Is your home air-conditioned?

☐ No → **Go to Q30**
☐ Yes

Q28 How many rooms are air-conditioned?

☐ One room
☐ Two rooms
☐ Three rooms
☐ More than three rooms
☐ Portable air-conditioner

Q29 Is it evaporative air-conditioning?

☐ No
☐ Yes

Q30 Is your home heated?

☐ No → **Go to Q31**
☐ Yes

How is your home heated?

*Please mark **all** applicable responses*	**Yes**	**Is there are chimney (flue) attached?** **Yes**	**No**
Gas heater	☐	☐	☐
Kerosene heater	☐	☐	☐
Electric bar radiator, fan or column heater	☐		
Reverse cycle air-conditioning	☐		
Fully ducted heating	☐		
Wood fire/slow combustion heater	☐		
Other *Please specify*……………………………………	☐		

Q31 Do you have gas cooking in your home?

☐ No
☐ Yes

Q32 Are there any pets at home?

☐ No → **Go to Q33**
☐ Yes

↓

How many pets are there?

If you have no pets at home then please leave this section blank. If you have either no cats, no dogs or birds or no other pets, then print the number 0 in the spaces.

Please print the number in the space provided	**Inside**	**Outside**	**Total**
Cats	☐☐	☐☐	☐☐
Dogs	☐☐	☐☐	☐☐
Birds (include ducks, geese, chickens)	☐☐	☐☐	☐☐

Other pets?

How many other pets inside? ☐☐ What type? ………………………………………………

How many other pets outside? ☐☐ What type? ………………………………………………

Q33 Date questionnaire completed: ☐☐ / ☐☐ / ☐☐☐☐

End of Part A

Please continue to Part B – Your Teenager

If you have more than one child participating in the study please complete a separate Part B for each of your study teenagers

THANK YOU

ID ☐☐☐☐

OFFICE USE ONLY

RA-CH RA-CO RA1-E RA2-E

ID

GROWING UP HEALTHY STUDY

Primary Caregiver Questionnaire

Part B

13-15

YOUR TEENAGER

Please answer the following sections in relation to your study teenager:

....

Q1 On average, how much time is spent with your teenager each day from Monday to Friday (only count the time you spend interacting with each other, helping with homework, talking and just "being together" - excluding sleeping)?

Please mark **one** *response for each person (if applicable)*

	None	Less than 1 hour	About 1 hour	About 1 to 2 hours	About 3 to 5 hours	More than 5 hours
Teen's Mother	☐	☐	☐	☐	☐	☐
Teen's Father	☐	☐	☐	☐	☐	☐
Your Partner/Defacto	☐	☐	☐	☐	☐	☐

Q2 On average, how much time is spent with your teenager each day on the weekend (only count the time you spend interacting with each other, helping with homework, talking and just "being together" - excluding sleeping)?

Please mark **one** *response for each person (if applicable)*

	None	Less than 1 hour	1 to 5 hours	6 to 10 hours	11 to 20 hours
Teen's Mother	☐	☐	☐	☐	☐
Teen's Father	☐	☐	☐	☐	☐
Your Partner/Defacto	☐	☐	☐	☐	☐

Q3 Compared with other children how easy or difficult is your teenager to manage?
Please mark the **one** *response which best represents your feelings*

☐ Much more difficult than average
☐ A little more difficult than average
☐ Average
☐ A little easier than average
☐ Much easier than average

Q4 How much time does your teenager usually spend watching TV or videos?

☐ None
☐ Up to 1 hour a day (3 to 6 hrs a week)
☐ Between 1 and 2 hours a day (7 to 13 hrs a week)
☐ Between 2 and 3 hours a day (14 to 21 hrs a week)
☐ 4 hours or more a day (21 hrs or more a week)

Q5 How much time does your teenager usually spend using a computer (including Internet and chat use)?

☐ None
☐ Up to 1 hour a day (3 to 6 hrs a week)
☐ Between 1 and 2 hours a day (7 to 13 hrs a week)
☐ Between 2 and 3 hours a day (14 to 21 hrs a week)
☐ 4 hours or more a day (21 hrs or more a week)

Q6 How would you compare the physical activity level of your teenager with that of other teenagers of the same age?

- ☐ I am unable to make the comparison
- ☐ My teenager is less active than other children
- ☐ My teenager is as active as other children
- ☐ My teenager is more active than other children

Q7 How does your teenager's level of activity now compare to 12 months ago?

- ☐ Less active than 12 months ago
- ☐ About the same as 12 months ago
- ☐ More active than 12 months ago

Q8 How would you rate the ability level of your teenager for each of the following skills?

Please mark **one** *response for each item*	**Poor**	**Below Average**	**Average**	**Above Average**	**Excellent**
**a.** Running	☐	☐	☐	☐	☐
**b.** Jumping	☐	☐	☐	☐	☐
**c.** Hopping	☐	☐	☐	☐	☐
**d.** Skipping	☐	☐	☐	☐	☐
**e.** Throwing	☐	☐	☐	☐	☐
**f.** Catching	☐	☐	☐	☐	☐
**g.** Kicking	☐	☐	☐	☐	☐
**h.** Striking/hitting	☐	☐	☐	☐	☐
**i.** Dodging	☐	☐	☐	☐	☐
**j.** Biking	☐	☐	☐	☐	☐
**k.** Balancing	☐	☐	☐	☐	☐

Q9 What is the name and suburb of the school your teenager is currently attending?

School: …………………………………………………… Suburb: ……………………..………………….

Q10 What year/grade is your teenager in at school now? ☐☐ year/grade

Q11 Has your teenager ever repeated a year/grade at school?

- ☐ No
- ☐ Yes *Which year(s)/grade(s)?*..

OFFICE USE ONLY

Q9 ☐☐☐☐

Q12 How satisfied are you with the standard of education offered at your teenager's current school?

- ☐ Very dissatisfied
- ☐ Dissatisfied
- ☐ Neither satisfied or dissatisfied
- ☐ Satisfied
- ☐ Very satisfied

Q13 How would you describe your teenager's academic performance in school during the past 6 months?

- ☐ Poor
- ☐ Below average
- ☐ Average
- ☐ Very good
- ☐ Excellent

Q14 How satisfied are you with your teenager's progress at school in the following areas:

Please mark **one** *response for each item*	**Very Satisfied**	**Satisfied**	**Neither**	**Dissatisfied**	**Very Dissatisfied**
**a.** Learning skills?	☐	☐	☐	☐	☐
**b.** Physical development, coordination?	☐	☐	☐	☐	☐
**c.** Getting on with other children?	☐	☐	☐	☐	☐
**d.** General behaviour?	☐	☐	☐	☐	☐

Q15 Is your teenager limited in the kind or amount of school work he/she does because of physical problems?

- ☐ No → **Go to Q17**
- ☐ Yes

↓

Q16 How long has your teenager been limited in this way?

- ☐ < 6 months
- ☐ 6 months to 2 years
- ☐ More than 2 years

Q17 Is your teenager limited in the kind or amount of school work he/she does because of emotional problems?

- ☐ No → **Go to Q19**
- ☐ Yes

↓

Q18 How long has your teenager been limited in this way?

- ☐ < 6 months
- ☐ 6 months to 2 years
- ☐ More than 2 years

Q19 Is your teenager limited in the kind or amount of school work he/she does because of learning problems?

- ☐ No → **Go to Q21**
- ☐ Yes

Q20 How long has your teenager been limited in this way?

- ☐ < 6 months
- ☐ 6 months to 2 years
- ☐ More than 2 years

Q21 Is your teenager limited in the kind or amount of school work he/she does because of speech and/or language problems?

- ☐ No → **Go to Q23**
- ☐ Yes

Q22 How long has your teenager been limited in this way?

- ☐ < 6 months
- ☐ 6 months to 2 years
- ☐ More than 2 years

Q23 Has your teenager ever received any of the following types of special education or special teaching?

Please mark **one** *response for each item*	**No**	**Yes, full-time**	**Yes, part-time**
**a.** For children with visual or hearing difficulties?	☐	☐	☐
**b.** For children with speech and/or language problems?	☐	☐	☐
**c.** For children who are intellectually handicapped?	☐	☐	☐
**d.** For children with emotional or behavioural problems?	☐	☐	☐
**e.** For children who are intellectually gifted?	☐	☐	☐
**f.** For children with remedial education needs?	☐	☐	☐

Q24 During the past six months has your teenager (or have you on your teenager's behalf) had contact with a school counsellor or guidance officer?

- ☐ No
- ☐ Yes *How many times?...........................*

Q25 During the past six months has your teenager (or have you on your teenager's behalf) had contact with a teacher for a behavioural problem or a learning problem?

- ☐ No
- ☐ Yes *How many times?...........................*

Q26 Does your teenager take part in any of the following activities outside of school hours:

Please mark **one** *response for each item*	**No**	**Yes**
**a.** Organised groups such as scouts, guides, church groups?	☐	☐
**b.** Organised sport like football, netball, little athletics?	☐	☐
**c.** Informal sporting activities like swimming, rollerblading?	☐	☐
**d.** Music, art, drama, dance outside of school?	☐	☐
**e.** Informal recreation like going to the movies or swimming pool	☐	☐
**f.** Going to friend's houses (any friends, not necessarily school friends)?	☐	☐

Q27 How satisfied are you with the opportunities that your teenager has to take part in activities outside school?

☐ Very dissatisfied
☐ Dissatisfied
☐ Neither satisfied or dissatisfied
☐ Satisfied
☐ Very satisfied

Q28 How would you rate the overall health of your teenager?

☐ Poor (seldom well)
☐ So-so (he/she is ill as often as he/she is well)
☐ OK, could be better (mostly well)
☐ Excellent (nearly always well)

Q29 Is your teenager limited in any physical activities (e.g. running, biking, climbing stairs, lifting, dressing) because of health problems?

☐ No → **Go to Q31**
☐ Yes

Q30 How long has your teenager been limited in this way?

☐ < 6 months
☐ 6 months to 2 years
☐ More than 2 years

Q31 Does your teenager have now, or had in the past, any of the following <u>health professional diagnosed</u> medical conditions or health problems?

*Please mark **one** response for each item*	**No**	**Yes, in the past**	**Yes, now**	**Yes, now and in the past**
Anxiety problems	☐	☐	☐	☐
Arthritis or joint problems	☐	☐	☐	☐
Asthma	☐	☐	☐	☐
Attentional problems	☐	☐	☐	☐
Back pain	☐	☐	☐	☐
Behavioural problems	☐	☐	☐	☐
Chronic respiratory or breathing problems (other than asthma)	☐	☐	☐	☐
Co-ordination or clumsiness difficulties	☐	☐	☐	☐
Depression	☐	☐	☐	☐
Developmental disorder (e.g. attention deficit disorder, autism, intellection disability)	☐	☐	☐	☐
Hayfever or some other allergy	☐	☐	☐	☐
Hearing impairment or deafness	☐	☐	☐	☐
Heart condition	☐	☐	☐	☐
Intellectual disability	☐	☐	☐	☐
Learning problems	☐	☐	☐	☐
Migraine or severe headache	☐	☐	☐	☐
Neck pain	☐	☐	☐	☐
Sleep disturbance	☐	☐	☐	☐
Speech and/or language problems	☐	☐	☐	☐
Vision problems	☐	☐	☐	☐
Any other medical condition or health problem not mentioned above	☐	☐	☐	☐

OFFICE USE ONLY

Q32

1 ☐☐☐☐☐☐☐☐☐☐ ☐☐☐☐☐☐☐☐☐☐ ☐☐☐☐☐☐☐☐☐☐

5 ☐☐☐☐☐☐☐☐☐☐ ☐☐☐☐☐☐☐☐☐☐ ☐☐☐☐☐☐☐☐☐☐

9 ☐☐☐☐☐☐☐☐☐☐ ☐☐☐☐☐☐☐☐☐☐ ☐☐☐☐☐☐☐☐☐☐

13 ☐☐☐☐☐☐☐☐☐☐ ☐☐☐☐☐☐☐☐☐☐ ☐☐☐☐☐☐☐☐☐☐

Q32 If you have answered "Yes" to any of the above, or your teenager has any other <u>health professional diagnosed</u> problem or condition please describe the condition or problem below in more detail:

Please list every medical condition/health problem separately - otherwise leave this blank

What condition/problem?	Who diagnosed it?	When was it diagnosed?	Treatment
e.g. Impacted wisdom teeth	*Dentist*	*6 months ago*	*Referral to dental surgeon, antibiotics*

Q33 Has your teenager had any accidents or injuries which required you to take him/her to a <u>doctor (GP), hospital or clinic</u>?

☐ No → **Go to Q34**
☐ Yes

Please describe the accident, the injury and any treatment (e.g. fell off bike, cut arm, 3 stitches), and list every accident/injury separately, giving as much detail as possible

Injury	How did it happen?	When did it happen?	Treatment
e.g. Sprained wrist	*Fell down stairs*	*3 months ago*	*Physiotherapy/bandage*

OFFICE USE ONLY

Q33 1
2
3
4
5

Q34 1
2
3
4
5

Q34 Has your teenager ever been admitted to hospital/day surgery?

- [ ] No → **Go to Q35**
- [ ] Yes

Please list each admission separately, giving as much detail as possible

**Date**	**Which hospital?**	**Reason for admission**
e.g. October 2005	*McCourt St Day Surgery*	*Removal of impacted wisdom teeth*

Q35 Has your teenager attended any of the following in the past 12 months?

- [ ] No → **Go to Q36**
- [ ] Yes

*Please mark **all** appropriate answers*	**Yes** Now completed	**Yes** Still attending regularly/occasionally
GP or family doctor	☐	☐
Accident and emergency	☐	☐
Hospital outpatient (department or clinic)	☐	☐
Private medical specialist	☐	☐
Dentist/Dental therapist/Orthodontist	☐	☐
School nurse	☐	☐
Optician/Optometrist	☐	☐
Dietician/Nutritionist	☐	☐
Physiotherapist	☐	☐
Occupational therapist (OT)	☐	☐
Speech therapist	☐	☐
Psychologist/Psychiatrist	☐	☐
Podiatrist	☐	☐
Chiropractor	☐	☐
Alternative therapist (e.g. iridologist)	☐	☐

Q36 In the past 6 months has your teenager taken/used any prescription medication(s)?

☐ No → **Go to Q37**
☐ Yes

Which medication(s)?

**Name**	**Reason for medication**	**Is he/she still taking the medication?**
e.g. Antibiotics<br>Ventolin<br>Cortisone cream	*For acne<br>For asthma<br>For eczema*	*Yes<br>Yes<br>No*

Q37 In the past 6 months has your teenager taken/used any 'over the counter' medication(s) (including vitamins, minerals and health food products)?

☐ No → **Go to Q38**
☐ Yes

Which medication(s)?

**Name**	**Reason for medication**	**Is he/she still taking the medication?**
e.g. Neurofen<br>Antihistamine	*For period pain<br>For hayfever*	

Q38 How many colds has your teenager had in the past 12 months? ☐☐

Q39 Does your teenager usually cough when he/she gets a cold these days?

☐ No
☐ Yes

Q40 Does your teenager seem congested or bring up phlegm (spit) from his/her chest with colds?

☐ No
☐ Yes

OFFICE USE ONLY

Q36 (1 … 10 … 20) ☐☐☐☐☐☐☐☐☐☐☐☐☐☐☐☐☐☐☐☐ / ☐☐☐☐☐☐☐☐☐☐☐☐☐☐☐☐☐☐☐☐

Q37 (1 … 10 … 20) ☐☐☐☐☐☐☐☐☐☐☐☐☐☐☐☐☐☐☐☐ / ☐☐☐☐☐☐☐☐☐☐

WHEEZE

Q41 Has your teenager wheezed at any time in his/her life? (wheeze is a whistling or rattling noise in the chest, best heard when breathing out)

- ☐ No → **Go to Q47**
- ☐ Yes

Q42 Has your teenager ever wheezed in the past 12 months?

- ☐ No → **Go to Q47**
- ☐ Yes

Q43 How many episodes of wheezing has your teenager had in the past 12 months?

- ☐ 1 to 2
- ☐ 3 to 12
- ☐ More than 12

In the past 12 months..

Q44 How often, on average has your teenager's sleep been disturbed due to wheezing?

- ☐ Never woken with wheezing
- ☐ Less than one night per week
- ☐ One or more nights per week

Q45 Has wheezing ever been severe enough to limit your teenager's speech to only one or two words at a time between breaths?

- ☐ No
- ☐ Yes

Q46 Has your teenager's chest sounded wheezy during or after exercise?

- ☐ No
- ☐ Yes

Q47 Has your teenager had a dry cough at night, apart from a cough associated with a cold or chest infection?

- ☐ No
- ☐ Yes

Q48 Has your teenager ever had an episode of coughing, congestion or bringing up phlegm lasting for more than 1 week?

- ☐ No → **Go to Q49**
- ☐ Yes

How many times ***without*** wheezing? ☐☐

How many times ***with*** wheezing? ☐☐

ASTHMA

Q49 Do you think your teenager has ever had asthma?

- ☐ No
- ☐ Yes
- ☐ Don’t know

Q50 Has a doctor (GP, pediatrician, respiratory specialist) ever told you that your teenager has asthma?

- ☐ No
- ☐ Yes

Q51 Does your teenager still have asthma?

- ☐ Not applicable (never had asthma)
- ☐ No
- ☐ Yes
- ☐ Don’t know

Q52 Has your teenager taken/used any of the following asthma medications in the past 12 months?

- ☐ No → **Go to Q53**
- ☐ Yes

		**Ordered by a Doctor**	
Please mark **all** *appropriate answers*	**Yes**	**Yes**	**No**
Ventolin (Asmol, Airomir, etc.)	☐	☐	☐
Respolin	☐	☐	☐
Nuelin	☐	☐	☐
Theo-dur	☐	☐	☐
Bricanyl	☐	☐	☐
Alupent	☐	☐	☐
Atrovent	☐	☐	☐
Intal or Intal Forte	☐	☐	☐
Qvar	☐	☐	☐
Becotide	☐	☐	☐
Flixotide	☐	☐	☐
Pulmicort	☐	☐	☐
Berotec	☐	☐	☐
Becloforte	☐	☐	☐
Tilade	☐	☐	☐
Oxis	☐	☐	☐
Serevent	☐	☐	☐
Singulair	☐	☐	☐
Accolate	☐	☐	☐
Seretide	☐	☐	☐
Symbicort	☐	☐	☐
Prednisolone	☐	☐	☐
Other *Please specify:*……………………………………	☐	☐	☐

The following questions are about problems which occurred when your teenager DID NOT have a cold or the flu:

RHINITIS (runny or blocked nose - including hayfever)

Q53 Has your teenager ever had a problem with sneezing or a runny or blocked nose (including hayfever) when he/she DID NOT have a cold or the flu?

- ☐ No → **Go to Q61**
- ☐ Yes

Q54 In the past 12 months, has your teenager had a problem with sneezing or a runny or blocked nose (including hayfever) when he/she DID NOT have a cold or the flu?

- ☐ No → **Go to Q56**
- ☐ Yes

Q55 In the past 12 months, was this nose problem accompanied by itchy-watery eyes?

- ☐ No
- ☐ Yes

Q56 Has a doctor (GP, pediatrician, respiratory specialist) ever told you that your teenager has an allergic nose problem (including hayfever)?

- ☐ No
- ☐ Yes

Q57 In the past 12 months, how many episodes of allergic nose problem (including hayfever) has your teenager had?

- ☐ None → **Go to Q61**
- ☐ 1 to 2
- ☐ 3 to 12
- ☐ More than 12

Q58 In which of the past 12 months did this problem occur?
Please mark **all** *months which apply*

☐ January	☐ July
☐ February	☐ August
☐ March	☐ September
☐ April	☐ October
☐ May	☐ November
☐ June	☐ December

Q59 What was the trigger/cause of these episodes?
*Please mark **all** responses that apply*

- [ ] Grass
- [ ] Pollen
- [ ] Animal
- [ ] Dust
- [ ] Other *Please specify:*…………………………………………………
- [ ] Don't know

Q60 Has your teenager taken/used any medication for an allergic nose problem (including hayfever)?

- [ ] No → **Go to Q61**
- [ ] Yes

Please list the medication and indicate if it was prescription or non-prescription.

Type of medication	Not Prescribed by doctor	Prescribed by doctor
Steroid nasal spray *(please specify):*	☐	☐
Non-steroid nasal spray *(please specify):*	☐	☐
Antihistamine drops/tablets *(please specify):*	☐	☐
Other *non-prescription* medication *(please specify):*	☐	
Other *prescription* medication *(please specify):*		☐

ALLERGIC CONJUNCTIVITIS (itchy water eyes - including hayfever)

Q61 Has your teenager ever had a problem with red/watery or itchy eyes?

- [ ] No → **Go to Q69**
- [ ] Yes

Q62 Do you think your teenager has ever had allergic reaction in the eyes (including hayfever)?

- [ ] No
- [ ] Yes
- [ ] Don't know

Q63 Has a doctor (GP, pediatrician, respiratory specialist) ever told you that your teenager had an allergic reaction in the eyes (including hayfever)?

- [ ] No
- [ ] Yes

Q64 In the past 12 months, has your teenager suffered from an allergic reaction in the eyes?

- ☐ No → **Go to Q69**
- ☐ Yes

Q65 How many episodes has your teenager had in the past 12 months?

- ☐ 1 to 2
- ☐ 3 to 12
- ☐ More than 12

Q66 In which of the past 12 months did this problem occur?
*Please mark **all** months which apply*

- ☐ January
- ☐ February
- ☐ March
- ☐ April
- ☐ May
- ☐ June
- ☐ July
- ☐ August
- ☐ September
- ☐ October
- ☐ November
- ☐ December

Q67 What was the trigger/cause of these episodes?
*Please mark **all** responses that apply*

- ☐ Grass
- ☐ Pollen
- ☐ Animal
- ☐ Dust
- ☐ Other *Please specify:*……………………………………………………
- ☐ Don't know

Q68 Has your teenager taken/used any medication for an allergic reaction in the eyes?

- ☐ No → **Go to Q69**
- ☐ Yes

Please list the medication and indicate if it was prescription or non-prescription.

**Type of medication**	**Not Prescribed by doctor**	**Prescribed by doctor**
Eye drops *(please specify):*	☐	☐
Steroid tablets *(please specify):*	☐	☐
Antihistamine drops/tablets *(please specify):*	☐	☐
Other *non-prescription* medication *(please specify):*	☐	
Other *prescription* medication *(please specify):*		☐

ECZEMA (itchy rash)

Q69 Has your teenager ever had eczema or an itchy rash, which was coming and going for at least 6 months?

- [ ] No → **Go to Q80**
- [ ] Yes

Q70 Has this eczema / itchy rash at any time affected any one of the following places; the folds of the elbows, behind the knees, in front of the ankles, under the buttocks, or around the neck, ears, or eyes?

- [ ] No
- [ ] Yes

Q71 Has your teenager had this eczema/itchy rash any time in the past 12 months?

- [ ] No → **Go to Q74**
- [ ] Yes

Q72 Has this rash cleared completely at any time during the past 12 months?

- [ ] No
- [ ] Yes

Q73 In the past 12 months, how often, on average, has your teenager been kept awake at night by this itchy rash?

- [ ] Never in the past 12 months
- [ ] Less than one night per week
- [ ] One or more nights per week

Q74 Do you think your teenager has ever had eczema?

- [ ] No
- [ ] Yes
- [ ] Don't know

Q75 Has a doctor (GP, pediatrician) ever told you that your teenager has eczema?

- [ ] No
- [ ] Yes

Q76 Has your teenager suffered from eczema in the past 12 months?

- [ ] No → **Go to Q80**
- [ ] Yes

Q77 How many episodes of eczema has your teenager had in the past 12 months?

- [ ] 1 to 2
- [ ] 3 to 12
- [ ] More than 12

Q78 In which of the <u>past 12 months</u> did this problem occur?
*Please mark **all** months which apply*

☐ January ☐ July
☐ February ☐ August
☐ March ☐ September
☐ April ☐ October
☐ May ☐ November
☐ June ☐ December

Q79 Has your teenager taken/used any medication for eczema?

☐ No → **Go to Q80**
☐ Yes

Please list the medication and indicate if it was prescription or non-prescription.

**Type of medication**	**Not Prescribed by doctor**	**Prescribed by doctor**
Moisturiser *(please specify):*	☐	☐
Steroid cream *(please specify):*	☐	☐
Oral steroids *(please specify):*	☐	☐
Tacrolimus ointment *(please specify):*	☐	☐
Other *non-prescription* medication *(please specify):*	☐	
Other *prescription* medication *(please specify):*		☐

Q80 Does your teenager have any regular contact with pets outside your home? (e.g. relatives, neighbours, friends)

☐ No → **Go to Q81**
☐ Yes

What types of animals?

*Please mark **all** applicable responses*	**Yes**
Cats	☐
Dogs	☐
Birds	☐
Fish	☐
Rodents (e.g. guinea pigs, rabbits, rats/mice)	☐
Reptiles/Amphibia	☐
Other (e.g. farm animals - sheep, horses cattle)<br>*Please specify:*………………………………………………………………………………..	☐

Q81 Has your teenager ever had any food allergies?

☐ No → **Go to Q82**
☐ Yes

Example Only

Food Type	***What starts it***		***What reaction(s)***	***Severity of the reaction***		
	Eating	***Contact***		***Mild***	***Moderate***	***Severe***
Peanut Products	☒	☒	***Difficulty breathing***	☐	☒	☐

Please mark **all** *appropriate responses and write in the spaces provided*

**Food Type**	**What starts it**		**What reaction(s)**	**Severity of the reaction**		
	Eating	***Contact***		***Mild***	***Moderate***	***Severe***
Peanut Products	☐	☐		☐	☐	☐
Wheat/Yeast	☐	☐		☐	☐	☐
Dairy	☐	☐		☐	☐	☐
Fruit	☐	☐		☐	☐	☐
Eggs	☐	☐		☐	☐	☐
Seafood	☐	☐		☐	☐	☐
Preservatives/Coloring	☐	☐		☐	☐	☐
Other<br>*(Please specify below):*	☐	☐		☐	☐	☐

OFFICE USE ONLY

Q81 R	1	2	3	4	5	6	7	8	9	10	11	12
Pe	☐	☐	☐	☐	☐	☐	☐	☐	☐	☐	☐	☐
Wh	☐	☐	☐	☐	☐	☐	☐	☐	☐	☐	☐	☐
Da	☐	☐	☐	☐	☐	☐	☐	☐	☐	☐	☐	☐
Fr	☐	☐	☐	☐	☐	☐	☐	☐	☐	☐	☐	☐
Eg	☐	☐	☐	☐	☐	☐	☐	☐	☐	☐	☐	☐
Se	☐	☐	☐	☐	☐	☐	☐	☐	☐	☐	☐	☐
Pr	☐	☐	☐	☐	☐	☐	☐	☐	☐	☐	☐	☐
O	☐	☐	☐	☐	☐	☐	☐	☐	☐	☐	☐	☐

Q82 Has your teenager ever had any other allergies (excluding foods)?

☐ No → **Go to Q83**
☐ Yes

Example Only

Allergies	***What starts it***				***What reaction(s)***	***Severity of the reaction***		
	Ingestion	***Contact***	***Inhalation***	***Injection***		***Mild***	***Moderate***	***Severe***
House dust mites	☐	☒	☐	☐	***Difficulty breathing***	☒	☐	☐

Please mark **all** *appropriate responses and write in the spaces provided*

**Allergies**	**What starts it**				**What reaction(s)**	**Severity of the reaction**		
	Ingestion	***Contact***	***Inhalation***	***Injection***		***Mild***	***Moderate***	***Severe***
House dust mites	☐	☐	☐	☐		☐	☐	☐
Pollen	☐	☐	☐	☐		☐	☐	☐
Moulds	☐	☐	☐	☐		☐	☐	☐
Cat fur	☐	☐	☐	☐		☐	☐	☐
Animal fur other than cat	☐	☐	☐	☐		☐	☐	☐
Soaps/ detergents	☐	☐	☐	☐		☐	☐	☐
Insect bites	☐	☐	☐	☐		☐	☐	☐
Antibiotics	☐	☐	☐	☐		☐	☐	☐
Other medicines	☐	☐	☐	☐		☐	☐	☐
Other *(Please specify below):*	☐	☐	☐	☐		☐	☐	☐

OFFICE USE ONLY

Q82 R	1	2	3	4	5	6	7	8	9	10	11	12
Dm	☐	☐	☐	☐	☐	☐	☐	☐	☐	☐	☐	☐
Po	☐	☐	☐	☐	☐	☐	☐	☐	☐	☐	☐	☐
Mo	☐	☐	☐	☐	☐	☐	☐	☐	☐	☐	☐	☐
Ca	☐	☐	☐	☐	☐	☐	☐	☐	☐	☐	☐	☐
An	☐	☐	☐	☐	☐	☐	☐	☐	☐	☐	☐	☐
So	☐	☐	☐	☐	☐	☐	☐	☐	☐	☐	☐	☐
In	☐	☐	☐	☐	☐	☐	☐	☐	☐	☐	☐	☐
An	☐	☐	☐	☐	☐	☐	☐	☐	☐	☐	☐	☐
Om	☐	☐	☐	☐	☐	☐	☐	☐	☐	☐	☐	☐
O	☐	☐	☐	☐	☐	☐	☐	☐	☐	☐	☐	☐

The following questions (Q83-91) apply to your study teenager's family.

Q83 On average, over the past 6 months, about how many drinks of beer, wine, spirits or other alcoholic beverage have the people listed below taken?

*Please mark **one** response for each person (if applicable)*

	Don't drink alcohol	Less than 3 drinks a week	3 - 6 drinks a week	1 or 2 drinks a day	3 - 6 drinks a day	More than 6 drinks a day
Teen's Mother	☐	☐	☐	☐	☐	☐
Teen's Father	☐	☐	☐	☐	☐	☐
Your Partner/Defacto	☐	☐	☐	☐	☐	☐

Q84 In general how would you describe the health of the people listed below?

*Please mark **one** response for each person (if applicable)*

	Excellent	Very Good	Good	Fair	Poor
Teen's Mother	☐	☐	☐	☐	☐
Teen's Father	☐	☐	☐	☐	☐
Your Partner/Defacto	☐	☐	☐	☐	☐

Q85 Are the people listed below limited in any way in carrying out normal daily activities at home, at a job or in studying, because of a medical condition or health problem?

*Please mark **one** response for each person (if applicable)*

	No	Yes
Teen's Mother	☐	☐
Teen's Father	☐	☐
Your Partner/Defacto	☐	☐

Q86 This question asks about your family's history of asthma, allergic nose and eye reactions and eczema ***and*** whether or not it was diagnosed by a doctor:

Please mark **all** *applicable responses*

	**No**	**Yes In the past**	**Yes Now**	**Yes Now and in the past**	**Diagnosed by a Doctor No**	**Diagnosed by a Doctor Yes**
Has the teenagers' mother had…						
Asthma	☐	☐	☐	☐	☐	☐
Allergic nose reaction (inc. hayfever)	☐	☐	☐	☐	☐	☐
Allergic eye reaction	☐	☐	☐	☐	☐	☐
Eczema	☐	☐	☐	☐	☐	☐
Has the teenager's father had...						
Asthma	☐	☐	☐	☐	☐	☐
Allergic nose reaction (inc. hayfever)	☐	☐	☐	☐	☐	☐
Allergic eye reaction	☐	☐	☐	☐	☐	☐
Eczema	☐	☐	☐	☐	☐	☐
Have any of the teenager's brothers or sisters (siblings) had...						
Asthma	☐	☐	☐	☐	☐	☐
Allergic nose reaction (inc. hayfever)	☐	☐	☐	☐	☐	☐
Allergic eye reaction	☐	☐	☐	☐	☐	☐
Eczema	☐	☐	☐	☐	☐	☐

Q87 This question asks about your family's history of diabetes, kidney and heart conditions and arthritis ***and*** whether or not it was diagnosed by a doctor:

Please mark **all** *applicable responses*

	**No**	**Yes In the past**	**Yes Now**	**Yes Now and in the past**	**Diagnosed by a Doctor No**	**Diagnosed by a Doctor Yes**
Has the teenagers' mother had…						
Diabetes	☐	☐	☐	☐	☐	☐
Renal conditions (Kidney)	☐	☐	☐	☐	☐	☐
Arthritis	☐	☐	☐	☐	☐	☐
Cardiac conditions (Heart)	☐	☐	☐	☐	☐	☐
Has the teenager's father had...						
Diabetes	☐	☐	☐	☐	☐	☐
Renal conditions (Kidney)	☐	☐	☐	☐	☐	☐
Arthritis	☐	☐	☐	☐	☐	☐
Cardiac conditions (Heart)	☐	☐	☐	☐	☐	☐
Have any of the teenager's brothers or sisters (siblings) had...						
Diabetes	☐	☐	☐	☐	☐	☐
Renal conditions (Kidney)	☐	☐	☐	☐	☐	☐
Arthritis	☐	☐	☐	☐	☐	☐
Cardiac conditions (Heart)	☐	☐	☐	☐	☐	☐

Q88 Has the study teenager's mother ever had post-natal depression?
*Please mark **all** appropriate answers*

- ☐ No
- ☐ Yes, with a child(ren) born before the study teenager
- ☐ Yes, with a child(ren) born after the study teenager
- ☐ Yes, associated with the birth of the study teenager
- ☐ Don't know, unsure

Q89 Have any of the people listed below ever been treated for an emotional or mental health problem (other than post-natal depression)?

*Please mark **one** response for each person (if applicable)*

	**No**	**Yes**
Teen's Mother	☐	☐
Teen's Father	☐	☐
Your Partner/Defacto	☐	☐

Q90 Have any of the people listed below been treated for an emotional or mental health problem within the past 6 months?

*Please mark **one** response for each person (if applicable)*

	**Yes**	**No**	**N/A (Never had a treated emotional problem)**
Teen's Mother	☐	☐	☐
Teen's Father	☐	☐	☐
Your Partner/Defacto	☐	☐	☐

Q91 Have any of the people listed below ever been hospitalised for an emotional or mental health problem?

*Please mark **one** response for each person (if applicable)*

	**Yes**	**No**	**N/A (Never had a treated emotional problem)**
Teen's Mother	☐	☐	☐
Teen's Father	☐	☐	☐
Your Partner/Defacto	☐	☐	☐

CHILD BEHAVIOUR CHECKLIST (FOR AGES 4-18 YEARS)

Q92 This question asks for information on the health, behaviour and social activities of your teenager to help us to describe different patterns of behaviour and to understand how these affect the health, education and wellbeing of children.

Please answer all items as well as you can, even if some do not seem to apply to your teenager.

For each item that describes your teenager now, or within the ***past 6 months****, please mark only one response as:*

0=Not True (as far as you know) **1=Somewhat or Sometimes True** **2=Very true or Often true**

	0	1	2		0	1	2
1. Acts too young for his/her age	☐	☐	☐	28. Eats or drinks things that are not food (don't include sweets (describe):………………………………………………………………………………	☐	☐	☐
2. Allergy (describe):………………………………………………………………………………………	☐	☐	☐	29. Fears certain animals, situations or places, other than school (describe): ………………………………………………………………………………	☐	☐	☐
3. Argues a lot	☐	☐	☐	30. Fears going to school	☐	☐	☐
4. Asthma	☐	☐	☐	31. Fears he/she might think or do something bad	☐	☐	☐
5. Behave like opposite sex	☐	☐	☐	32. Feels he/she has to be perfect	☐	☐	☐
6. Bowel movements outside toilet	☐	☐	☐	33. Feels or complains that no one loves him/her	☐	☐	☐
7. Bragging, boasting	☐	☐	☐	34. Feels others are out to get him/her	☐	☐	☐
8. Can't concentrate, can't pay attention for long	☐	☐	☐	35. Feels worthless or inferior	☐	☐	☐
9. Can't get his/her mind off certain thoughts; obsessions (describe):………………………………………………………………………………	☐	☐	☐	36. Gets hurt a lot or accident-prone	☐	☐	☐
10. Can't sit still, restless, hyperactive	☐	☐	☐	37. Gets in many fights	☐	☐	☐
11. Clings to adults or too dependent	☐	☐	☐	38. Gets teased a lot	☐	☐	☐
12. Complains of loneliness	☐	☐	☐	39. Hangs around with others who get in trouble	☐	☐	☐
13. Confused or seems to be in a fog	☐	☐	☐	40. Hears sounds or voices that aren't there (describe):……………………………………………	☐	☐	☐
14. Cries a lot	☐	☐	☐	41. Impulsive or acts without thinking	☐	☐	☐
15. Cruel to animals	☐	☐	☐	42. Would rather be alone than with others	☐	☐	☐
16. Cruelty, bullying or meanness to others	☐	☐	☐	43. Lying or cheating	☐	☐	☐
17. Day-dreams or gets lost in his/her thoughts	☐	☐	☐	44. Bites fingernails	☐	☐	☐
18. Deliberately harms self or attempts suicide	☐	☐	☐	45. Nervous, high strung or tense	☐	☐	☐
19. Demands a lot of attention	☐	☐	☐	46. Nervous movements or twitching (describe): ………………………………………………………	☐	☐	☐
20. Destroys his/her own things	☐	☐	☐	47. Nightmares	☐	☐	☐
21. Destroys things belonging to his/her family or others	☐	☐	☐	48. Not liked by other kids	☐	☐	☐
22. Disobedient at home	☐	☐	☐	49. Constipated, doesn't move bowels	☐	☐	☐
23. Disobedient at school	☐	☐	☐	50. Too fearful or anxious	☐	☐	☐
24. Doesn't eat well	☐	☐	☐	51. Feels dizzy	☐	☐	☐
25. Doesn't get along with other kids	☐	☐	☐	52. Feels too guilty	☐	☐	☐
26. Doesn't seem to feel guilty after misbehaving	☐	☐	☐	53. Overeating	☐	☐	☐

27. Easily jealous	☐	☐	☐	54. Overtired	☐	☐	☐

0=Not True (as far as you know) **1=Somewhat or Sometimes True** **2=Very true or Often true**

	0	1	2
55. Overweight	☐	☐	☐
56. Physical problems without medical cause:			
a. Aches or pains (not headaches)	☐	☐	☐
b. Headaches	☐	☐	☐
c. Nausea, feels sick	☐	☐	☐
d. Problems with eyes (describe):	☐	☐	☐
e. Rashes or other skin problems	☐	☐	☐
f. Stomach aches or cramps	☐	☐	☐
g. Vomiting, throwing up	☐	☐	☐
h. Other (describe):	☐	☐	☐
57. Physically attacks people	☐	☐	☐
58. Picks nose, skin or other parts of body (describe):………………………………………	☐	☐	☐
59. Plays with own sex parts in public	☐	☐	☐
60. Plays with own sex parts too much	☐	☐	☐
61. Poor school work	☐	☐	☐
62. Poorly coordinated or clumsy	☐	☐	☐
63. Prefers being with older kids	☐	☐	☐
64. Prefers being with younger kids	☐	☐	☐
65. Refuses to talk	☐	☐	☐
66. Repeats certain acts over and over; compulsions (describe):………………………………………	☐	☐	☐
67. Runs away from home	☐	☐	☐
68. Screams a lot	☐	☐	☐
69. Secretive, keeps things to self	☐	☐	☐
70. Sees things that aren't there (describe):……………………………………… ………………………………………………………	☐	☐	☐
71. Self-conscious or easily embarrassed	☐	☐	☐
72. Sets fires	☐	☐	☐
73. Sexual problems (describe):……………………………………… ………………………………………………………	☐	☐	☐
74. Showing off or clowning	☐	☐	☐
75. Shy or timid	☐	☐	☐
76. Sleeps less than most kids	☐	☐	☐
77. Sleeps more than most kids during the day and/or at night (describe):	☐	☐	☐
78. Smears or plays with bowel movements	☐	☐	☐
79. Speech problem (describe):……………………………………… ………………………………………………………	☐	☐	☐
80. Stares blankly	☐	☐	☐

	0	1	2
81. Steals at home	☐	☐	☐
82. Steals outside the home	☐	☐	☐
83. Stores up things he/she doesn't need (describe):………………………………………	☐	☐	☐
84. Strange behaviour (describe):………………………………………	☐	☐	☐
85. Strange ideas (describe):………………………………………	☐	☐	☐
86. Stubborn, sullen or irritable	☐	☐	☐
87. Sudden changes in mood or feelings	☐	☐	☐
88. Sulks a lot	☐	☐	☐
89. Suspicious	☐	☐	☐
90. Swearing or obscene language	☐	☐	☐
91. Talks about killing self	☐	☐	☐
92. Talks or walks in sleep (describe):	☐	☐	☐
93. Talks too much	☐	☐	☐
94. Teases a lot	☐	☐	☐
95. Temper tantrums or hot temper	☐	☐	☐
96. Thinks about sex too much	☐	☐	☐
97. Threatens people	☐	☐	☐
98. Thumb sucking	☐	☐	☐
99. Too concerned about neatness or cleanliness	☐	☐	☐
100. Trouble sleeping (describe):	☐	☐	☐
101. Truancy, skips school	☐	☐	☐
102. Under active, slow moving or lacks energy	☐	☐	☐
103. Unhappy, sad or depressed	☐	☐	☐
104. Unusually loud	☐	☐	☐
105. Uses alcohol or drugs for non-medical purposes (describe):………….. ………………………………………………………	☐	☐	☐
106. Vandalism	☐	☐	☐
107. Wets self during the day	☐	☐	☐
108. Wets the bed	☐	☐	☐
109. Whining	☐	☐	☐
110. Wishes to be of opposite sex	☐	☐	☐
111. Withdrawn, doesn't get involved with others	☐	☐	☐
112. Worries	☐	☐	☐
113. Please write in any problems your teenager has that were not listed above: ……………………………………………………… ……………………………………………………… ………………………………………………………	☐	☐	☐

Q93 Are you worried about your teenager’s weight?

- ☐ Not at all
- ☐ A little
- ☐ Moderately
- ☐ Very

Q94 Do you consider your teenager to be…

- ☐ Underweight?
- ☐ Normal weight?
- ☐ A bit overweight?
- ☐ Very overweight?

Q95 Do you have any concerns or worries about your teenager's health or development (e.g. speech/language development, physical development, emotional development)?

- ☐ No → **Go to Q96**
- ☐ Yes

Please tell us about these concerns if you wish to do so in the space provided:

Q96 Date questionnaire completed: ☐☐ / ☐☐ / ☐☐☐☐

Q97 Please write below any comments concerning this questionnaire, the research or anything else you would like to tell us about:

THANK YOU

WE APPRECIATE THE TIME THAT YOU HAVE SPENT COMPLETING THIS QUESTIONNAIRE

ID ☐☐☐☐☐

OFFICE USE ONLY

RA-CH RA-CO RA1-E RA2-E

ID

GROWING UP HEALTHY STUDY

Participant Questionnaire

FEMALE

13-15

The purpose of this questionnaire is to obtain information about a range of topics including your household and neighbourhood, your study, job seeking and work experiences, and your health and wellbeing.

Please read each question carefully and answer all of the questions.
Write your answers clearly in the space provided or mark the most appropriate response

☒

Please take your time in answering all of the questions

If you are uncomfortable about a question or unsure of an answer, please leave it blank or ask one of the Growing Up Healthy Study team for assistance.

Please complete this questionnaire independently (without discussing it with anyone)

Remember all answers are STRICTLY confidential

Questionnaire

What is your date of birth?

☐☐ / ☐☐ / ☐☐☐☐

Are you **FEMALE**?

☐ Yes
☐ No (Please ask one of the Growing Up Healthy Study team for assistance)

SECTION 1 Physical Education and Physical Activity

Please mark only ***one*** *response for each question:*

Q1 How do you usually get to and from school?

Car	Bus	Bicycle	Walk	Home schooled
☐	☐	☐	☐	☐

Q2 How many physical education periods do you usually attend at school each week?

None	One per week	Two per week	Three or more per week
☐	☐	☐	☐

Q3 During physical education periods, how much time do you spend exercising that makes you out of breath or sweat?

Not much time or none at all	About a quarter of the time	About half of the time	More than half of the time	Almost all of the time
☐	☐	☐	☐	☐

Q4 What do you feel about your physical education periods?

I like them very much	I like them	I neither like nor dislike them	I dislike them	I dislike them very much	I do not attend them
☐	☐	☐	☐	☐	☐

Q5 <u>Outside school hours</u>: How often do you usually exercise in your free time, so much that you get out of breath or sweat?

Once per month or less	Once per week	2-3 times per week	4-6 times per week	Every day
☐	☐	☐	☐	☐

Q6 <u>Outside school hours:</u> How many hours do you usually exercise in your free time, so much that you get out of breath or sweat?

None	About ½ hour per week	About 1 hour per week	About 2-3 hours per week	About 4-6 hours per week	7 or more hours per week
☐	☐	☐	☐	☐	☐

Q7 On average, how many <u>hours per day</u> do you usually watch TV or videos (including school days and weekends)?

Not at all	Up to one hour per day	1-2 hours per day	2-3 hours per day	4 hours or more per day
☐	☐	☐	☐	☐

Q8 On average, how many hours per week do you usually watch TV or videos (including school days and weekends)?

Not at all	Up to seven hour per week	7-14 hours per week	14-21 hours per week	21 hours or more per week
☐	☐	☐	☐	☐

Q9 On average, how many hours per day do you usually use a computer, e.g. for homework, the Internet or chat online (including school days and weekends)?

Not at all	Up to one hour per day	1-2 hours per day	2-3 hours per day	4 hours or more per day
☐	☐	☐	☐	☐

Q10 On average, how many hours per week do you usually use a computer, e.g. for homework, the Internet or chat online (including school days and weekends)?

Not at all	Up to seven hour per week	7-14 hours per week	14-21 hours per week	21 hours or more per week
☐	☐	☐	☐	☐

Q11 How often do each of the following people play some sort of sport or exercise (e.g., golf, tennis, football) or other activity like walking for exercise, cycling or swimming?

Please mark **one** *response for each item*	I don't have/I don't know	Never or rarely	Sometimes	About once per week	2-3 times each week	More than 3 times per week
Father/male carer	☐	☐	☐	☐	☐	☐
Mother/female carer	☐	☐	☐	☐	☐	☐
Your best friend	☐	☐	☐	☐	☐	☐

Q12 How often do each of the following people praise you or encourage you to play some sort of sport or to participate in other physical activity (e.g., watch you participate, say positive things to you, seem happy to do it)?

Please mark **one** *response for each item*	I don't have/I don't know	Never or rarely	Sometimes	Often	Very often
Father/male carer	☐	☐	☐	☐	☐
Mother/female carer	☐	☐	☐	☐	☐
Your best friend	☐	☐	☐	☐	☐
A teacher at your school	☐	☐	☐	☐	☐

Q13 How often do each of the following people help you to play some sort of sport or to participate in other physical activity (e.g., take you to training, give sport money)?

Please mark **one** *response for each item*	I don't have/I don't know	Never or rarely	Sometimes	Often	Very often
Father/male carer	☐	☐	☐	☐	☐
Mother/female carer	☐	☐	☐	☐	☐
Your best friend	☐	☐	☐	☐	☐
A teacher at your school	☐	☐	☐	☐	☐

Q14 Below are some reasons for not doing more exercise or activities than you do. Please show how strongly each statement applies to you:

Please mark **one** *response for each item*	Does not apply at all	Applies a little	Applies a fair amount	Applies strongly	Applies very strongly
**a.** I already do a lot of exercise	☐	☐	☐	☐	☐
**b.** I am self-conscious about my looks when I exercise or play sport	☐	☐	☐	☐	☐
**c.** I don't have enough time	☐	☐	☐	☐	☐
**d.** I don't have enough energy	☐	☐	☐	☐	☐
**e.** There are other things I like doing more	☐	☐	☐	☐	☐
**f.** I don't have anyone to exercise or play with	☐	☐	☐	☐	☐
**g.** I just don't enjoy exercise or sport	☐	☐	☐	☐	☐
**h.** My parents don't encourage or help me	☐	☐	☐	☐	☐
**i.** The right facilities are not available	☐	☐	☐	☐	☐
**j.** I don't have the skills	☐	☐	☐	☐	☐
**k.** I am just not very good at any sports or activities	☐	☐	☐	☐	☐
**l.** Others laugh/make fun of me when I try to play	☐	☐	☐	☐	☐
**m.** My health is not good enough	☐	☐	☐	☐	☐
**n.** I have an injury which prevents me	☐	☐	☐	☐	☐
**o.** Another reason *(please describe)*	☐	☐	☐	☐	☐

SECTION 2 Eating Habits, Weight, Physical Health and Development

Q15 How often do you eat the following foods?

Please mark **one** *response for each item*	6 + times a week	3-5 times a week	1-2 times a week	1-2 times a month	Rarely or never
**a.** Fried food with a batter or breadcrumb coating	☐	☐	☐	☐	☐
**b.** Gravy, creamy sauces or cheese sauces	☐	☐	☐	☐	☐
**c.** Vegetables, rice or pasta <u>with added</u> butter, margarine, oil or sour cream	☐	☐	☐	☐	☐
**d.** Vegetables that are fried or roasted with fat or oil (don't count oil sprays)	☐	☐	☐	☐	☐
**e.** Sausages, polony, salami, meat pies, pasties, hamburger or bacon	☐	☐	☐	☐	☐
**f.** Hot potato chips or French fries	☐	☐	☐	☐	☐
**g.** Pastries, cakes, sweet biscuits or croissants	☐	☐	☐	☐	☐
**h.** Chocolate, chocolate biscuits or sweet snack bars	☐	☐	☐	☐	☐
**i.** Potato crisps, corn chips, cheezels, twisties or nuts	☐	☐	☐	☐	☐
**j.** Ice cream (any variety)	☐	☐	☐	☐	☐
**k.** Cream or sour cream	☐	☐	☐	☐	☐
**l.** Cheddar, edam or other hard cheese, cream cheese or soft cheeses such as camembert or brie (but excluding ricotta or cottage cheese)	☐	☐	☐	☐	☐

Q16 How much of the following do you usually eat? (*Please mark* **one** *response for each item)*

a Fat on meat?

- ☐ Most or all
- ☐ Some
- ☐ None
- ☐ Don't eat meat

b Skin on chicken?

- ☐ Most or all
- ☐ Some
- ☐ None
- ☐ Don't eat chicken

Q17 How often do you eat the following foods?

Please mark **one** *response for each item*	6 + times a week	3-5 times a week	1-2 times a week	1-2 times a month	Rarely or never
**Fruit**, including fresh and canned fruit (do not include dried fruit, fruit juices, fruit drinks, fruit bars or frozen fruit desserts)	☐	☐	☐	☐	☐
**Vegetables**, including all forms of vegetables, e.g. fresh, frozen, canned and salads	☐	☐	☐	☐	☐

Q18 Here we are asking for some additional information on how often and how much of the following drinks you usually consume.

When answering these questions, please mark how often you have the drink and write the total number of glasses, cans, or cups you would usually drink (see example). To assist you, below each type of drink is the type of measurement.

	Never	less than once/ month	**1** day/ month	**2** days/ month	**3** days/ month	**1** day/ week	**2** days/ week	**3** days/ week	**4** days/ week	**5** days/ week	**6** days/ week	every day	**Total number of glasses/ cups/cans you usually drink**
i.e. **Water (250 ml glass)**	☐	☐	☐	☐	☐	☐	☐	☐	☐	☐	☒	☐	**8**
**1.** Water (250 ml glass)	☐	☐	☐	☐	☐	☐	☐	☐	☐	☐	☐	☐	
**2.** Fizzy drink (e.g. cola, lemonade) (can, glass)	☐	☐	☐	☐	☐	☐	☐	☐	☐	☐	☐	☐	
**3.**Diet fizzy drink (e.g. diet cola, diet lemonade) (can, glass)	☐	☐	☐	☐	☐	☐	☐	☐	☐	☐	☐	☐	
**4.** Energy drink (e.g. Redbull, V, Monster) (can)	☐	☐	☐	☐	☐	☐	☐	☐	☐	☐	☐	☐	
**5.** Diet energy drink (can)	☐	☐	☐	☐	☐	☐	☐	☐	☐	☐	☐	☐	
**6.** Tea (cup)	☐	☐	☐	☐	☐	☐	☐	☐	☐	☐	☐	☐	
**7.** Herbal tea (cup)	☐	☐	☐	☐	☐	☐	☐	☐	☐	☐	☐	☐	
**8.** Green tea (cup)	☐	☐	☐	☐	☐	☐	☐	☐	☐	☐	☐	☐	
**9.** Instant coffee (cup)	☐	☐	☐	☐	☐	☐	☐	☐	☐	☐	☐	☐	
**10.** Ground coffee (e.g. filter coffee, cappuccino, flat white) (cup, mug)	☐	☐	☐	☐	☐	☐	☐	☐	☐	☐	☐	☐	

Q19 Do you know how much you weigh?

☐ No → **Go to Q21**
☐ Yes

Q20 What is your current weight? ☐☐☐ . ☐ kg

OFFICE USE ONLY
Q18 1☐☐ 2☐☐ 3☐☐ 4☐☐ 5☐☐ 6☐☐ 7☐☐ 8☐☐ 9☐☐ 10☐☐

Please mark only **one** *response for each question below*

Q21 Are you worried about your weight?

**No, not at all**	**A little**	**Moderately**	**Very**
☐	☐	☐	☐

Q22 Do you consider yourself to be?

**Underweight**	**Normal weight**	**A bit overweight**	**Very overweight**
☐	☐	☐	☐

Q23 How often do you weigh yourself?

**Never**	**Once in a while**	**Often**	**Nearly every day**
☐	☐	☐	☐

Please look at the figures and select from the list of numbers and letters A and B provided:

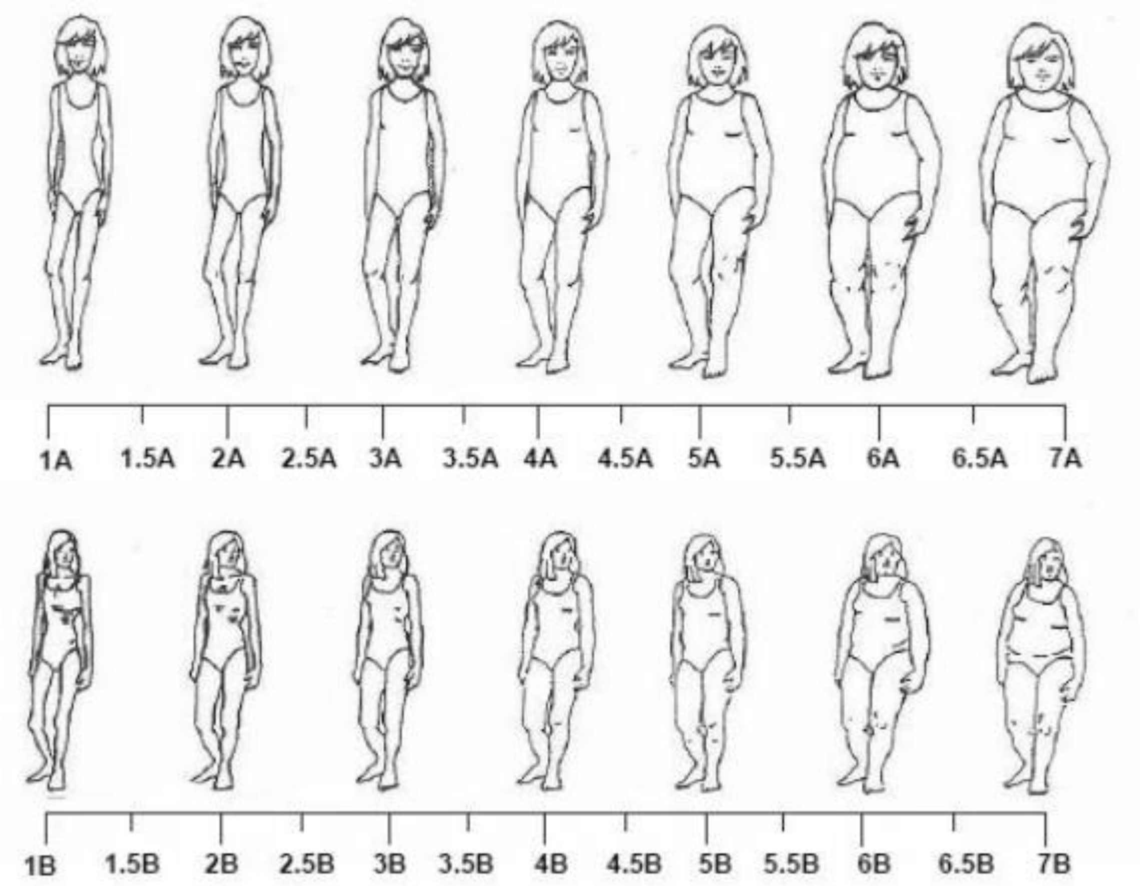

	Number	***Letter***
Q24 Which figure best represents what you currently look like?	☐	☐
Q25 Which figure would you most like to look like?	☐	☐

The drawings below show different amounts of female pubic hair. A girl passes through each of the four stages shown by these drawings.

Q26*a* Please look at each drawing then choose the one closest to your stage of development by placing an X in the corresponding box.

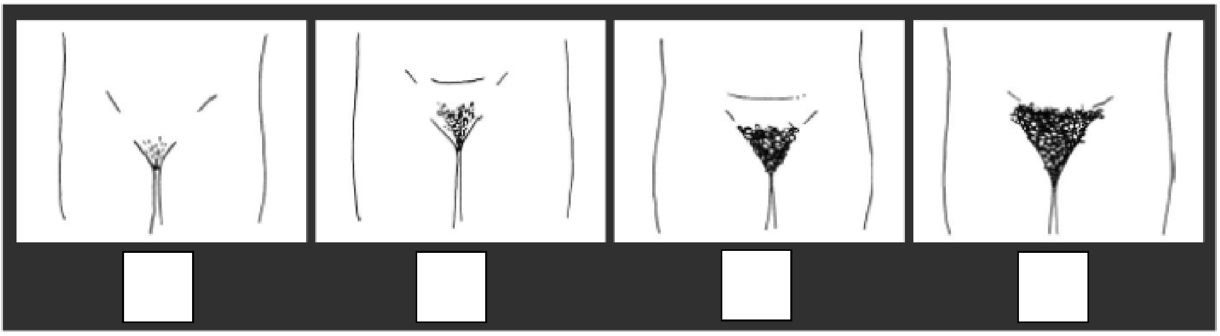

The drawings below show different stages of development of the breasts. A female passes through each of the five stages shown by these drawings.

Q26*b* Please look at each drawing then choose the one closest to your stage of development by placing an X in the corresponding box.

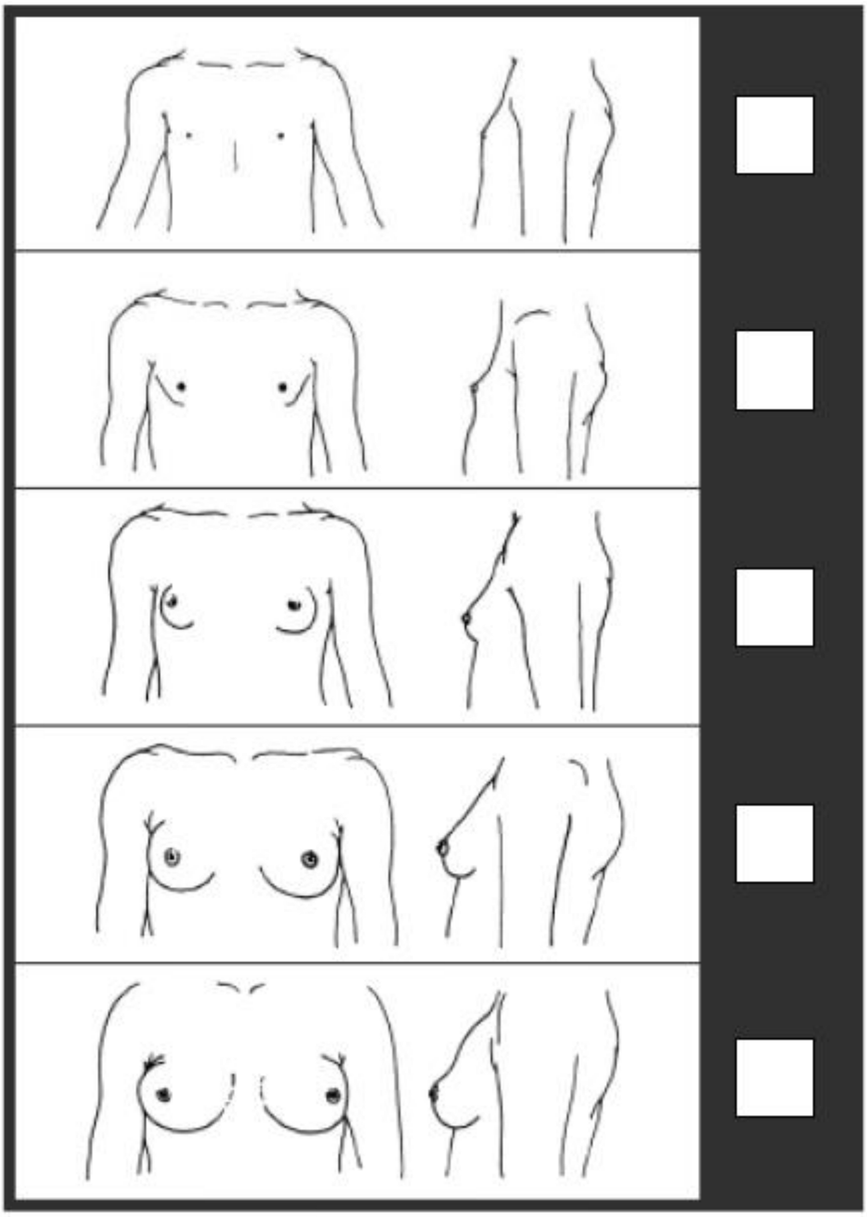

Q27 **Over the last two weeks…**

a. Have you been trying hard to eat less to change your shape or weight (even if you haven't managed to do so)?

Not at all	Some of the time	A lot of the time	Most of the time
☐	☐	☐	☐

b. Have you gone for long periods of time (8 hours or more) without eating anything to try and change your shape or weight?

Not at all	Some of the time	A lot of the time	Most of the time
☐	☐	☐	☐

c. Have you tried not to eat certain foods (like chocolate or chips to try to change your shape or weight (even if you haven't been able to do so)?

Not at all	Some of the time	A lot of the time	Most of the time
☐	☐	☐	☐

d. Have you tried to stick to any definite rules about diet or eating (e.g., sticking to a calorie limit, a set amount of food or rules about what or when you should eat (even if you haven't been able to do so)?

Not at all	Some of the time	A lot of the time	Most of the time
☐	☐	☐	☐

e. Have you been thinking about food or calories so much that you've found it hard to concentrate on things you are interested in (e.g., reading, watching TV or following a conversation)?

Not at all	Some of the time	A lot of the time	Most of the time
☐	☐	☐	☐

f. Have there been times when you feel that you have eaten an unusually large amount of food (more than what most people would eat in the same situation)?

Not at all	Some of the time	A lot of the time	Most of the time
☐	☐	☐	☐

g. Have you been afraid of losing control over your eating?

Not at all	Some of the time	A lot of the time	Most of the time
☐	☐	☐	☐

h. Have you felt that you couldn't control what or how much you were eating?

Not at all	Some of the time	A lot of the time	Most of the time
☐	☐	☐	☐

i. Have you felt that you couldn't stop eating once you had started?

Not at all	Some of the time	A lot of the time	Most of the time
☐	☐	☐	☐

j. Have you felt guilty after eating?

Not at all	Some of the time	A lot of the time	Most of the time
☐	☐	☐	☐

k. Have you eaten in secret because you are embarrassed by how much you eat?

Not at all	Some of the time	A lot of the time	Most of the time
☐	☐	☐	☐

l. Have you been afraid that you might gain weight or become fat?

Not at all	Some of the time	A lot of the time	Most of the time
☐	☐	☐	☐

m. Have you felt fat?

Not at all	Some of the time	A lot of the time	Most of the time
☐	☐	☐	☐

n. Have you had a strong desire to lose weight?

Not at all	Some of the time	A lot of the time	Most of the time
☐	☐	☐	☐

o. Have you made yourself sick (vomit) after eating to control your weight?

Not at all	Some of the time	A lot of the time	Most of the time
☐	☐	☐	☐

p. Have you taken any pills (like laxatives, water pills, diet tubes) to try to control your weight?

Not at all	Some of the time	A lot of the time	Most of the time
☐	☐	☐	☐

q. Have you exercised hard to control your weight?

Not at all	Some of the time	A lot of the time	Most of the time
☐	☐	☐	☐

People have different ideas about what sort of things are important to them in how they think about themselves. For some people doing well at school is very important to them, for others, how they are getting on with friends is very important. We're now going to ask you to think about how important weight and shape is to you:

r. Has your <u>weight</u> been important in how you think of yourself as a person?

Not at all	Some of the time	A lot of the time	Most of the time
☐	☐	☐	☐

s. Has your <u>shape</u> been important in how you think of yourself as a person?

Not at all	Some of the time	A lot of the time	Most of the time
☐	☐	☐	☐

SECTION 3 Adolescents and School

Q28 Below is a list of items that describes adolescents. For each item please mark the box that best represents whether the statement is true, somewhat/sometimes true or very true/often true for you now or within the past 6 months. Please answer all items as well as you can, even if some do not seem to apply to you:

Please mark **one** *response for each item*	**Not true**	**Somewhat/ sometimes true**	**Very true/ often true**
**1.** I act too young for my age	☐	☐	☐
**2.** I have an allergy	☐	☐	☐
**3.** I argue a lot	☐	☐	☐
**4.** I have asthma	☐	☐	☐
**5.** I like the opposite sex	☐	☐	☐
**6.** I like animals	☐	☐	☐
**7.** I brag	☐	☐	☐
**8.** I have trouble concentrating or paying attention	☐	☐	☐
**9.** I can't get my mind off certain thoughts	☐	☐	☐
**10.** I have trouble sitting still	☐	☐	☐
**11.** I am too dependent on adults	☐	☐	☐
**12.** I feel lonely	☐	☐	☐
**13.** I feel confused or in a fog	☐	☐	☐
**14.** I cry a lot	☐	☐	☐
**15.** I am pretty honest	☐	☐	☐
**16.** I am mean to others	☐	☐	☐
**17.** I day dream a lot	☐	☐	☐
**18.** I deliberately try to hurt or kill myself	☐	☐	☐
**19.** I try to get a lot of attention	☐	☐	☐
**20.** I destroy my own things	☐	☐	☐
**21.** I destroy things belonging to others	☐	☐	☐
**22.** I disobey my parents	☐	☐	☐
**23.** I disobey at school	☐	☐	☐
**24.** I don't eat as well as I should	☐	☐	☐
**25.** I don't get along with other kids	☐	☐	☐
**26.** I don't feel guilty after doing something I shouldn't	☐	☐	☐
**27.** I am jealous of others	☐	☐	☐
**28.** I am willing to help others when they need help	☐	☐	☐
**29.** I am afraid of certain animals, situations, or places other than school	☐	☐	☐
**30.** I am afraid of going to school	☐	☐	☐

Please mark **one** *response for each item*	**Not true**	**Somewhat/ sometimes true**	**Very true/ often true**
**31.** I am afraid I might think or do something bad	☐	☐	☐
**32.** I feel that I have to be perfect	☐	☐	☐
**33.** I feel that no one loves me	☐	☐	☐
**34.** I feel that others are out to get me	☐	☐	☐
**35.** I feel worthless or inferior	☐	☐	☐
**36.** I accidentally get hurt a lot	☐	☐	☐
**37.** I get in many fights	☐	☐	☐
**38.** I get teased a lot	☐	☐	☐
**39.** I hang around with kids who get in trouble	☐	☐	☐
**40.** I hear sounds or voices that other people think aren't there	☐	☐	☐
**41.** I act without stopping to think	☐	☐	☐
**42.** I would rather be alone than with others	☐	☐	☐
**43.** I lie or cheat	☐	☐	☐
**44.** I bite my fingernails	☐	☐	☐
**45.** I am nervous or tense	☐	☐	☐
**46.** Parts of my body twitch or make nervous movements	☐	☐	☐
**47.** I have nightmares	☐	☐	☐
**48.** I am not liked by other kids	☐	☐	☐
**49.** I can do certain things better than most kids	☐	☐	☐
**50.** I am too fearful or anxious	☐	☐	☐
**51.** I feel dizzy	☐	☐	☐
**52.** I feel too guilty	☐	☐	☐
**53.** I eat too much	☐	☐	☐
**54.** I feel overtired	☐	☐	☐
**55.** I am overweight	☐	☐	☐
**56.** Physical problems without known medical cause: **a.** Aches or pains (not headaches)	☐	☐	☐
**b.** Headaches	☐	☐	☐
**c.** Nausea, feel sick	☐	☐	☐
**d.** Problems with eyes	☐	☐	☐
**e.** Rashes or other skin problems	☐	☐	☐
**f.** Stomach-aches or cramps	☐	☐	☐
**g.** Vomiting, throwing up	☐	☐	☐
**h.** Other *(please describe)*…………………………..	☐	☐	☐

Please mark **one** *response for each item*	**Not true**	**Somewhat/ sometimes true**	**Very true/ often true**
**57.** I physically attack people	☐	☐	☐
**58.** I pick my skin or other parts of my body	☐	☐	☐
**59.** I can be pretty friendly	☐	☐	☐
**60.** I like to try new things	☐	☐	☐
**61.** My school work is poor	☐	☐	☐
**62.** I am poorly coordinated or clumsy	☐	☐	☐
**63.** I would rather be with older kids than kids my own age	☐	☐	☐
**64.** I would rather be with younger kids than kids my own age	☐	☐	☐
**65.** I refuse to talk	☐	☐	☐
**66.** I repeat certain actions over and over	☐	☐	☐
**67.** I run away from home	☐	☐	☐
**68.** I scream a lot	☐	☐	☐
**69.** I am secretive or keep things to myself	☐	☐	☐
**70.** I see things that other people think aren’t there	☐	☐	☐
**71.** I am self-conscious or easily embarrassed	☐	☐	☐
**72.** I set fires	☐	☐	☐
**73.** I can work well with my hands	☐	☐	☐
**74.** I show off or clown	☐	☐	☐
**75.** I am shy	☐	☐	☐
**76.** I sleep less than most kids	☐	☐	☐
**77.** I sleep more than most kids during the day and/or night	☐	☐	☐
**78.** I have a good imagination	☐	☐	☐
**79.** I have a speech problem	☐	☐	☐
**80.** I stand up for my rights	☐	☐	☐
**81.** I steal at home	☐	☐	☐
**82.** I steal from places other than home	☐	☐	☐
**83.** I store things up I don’t need	☐	☐	☐
**84.** I do things other people think are strange	☐	☐	☐
**85.** I have thoughts that other people would think are strange	☐	☐	☐
**86.** I am stubborn	☐	☐	☐
**87.** My moods or feelings change suddenly	☐	☐	☐
**88.** I enjoy being with other people	☐	☐	☐
**89.** I am suspicious	☐	☐	☐
**90.** I swear or use dirty language	☐	☐	☐

*Please mark **one** response for each item*	Not true	Somewhat/ sometimes true	Very true/ often true
**91.** I think about killing myself	☐	☐	☐
**92.** I like to make others laugh	☐	☐	☐
**93.** I talk too much	☐	☐	☐
**94.** I tease others a lot	☐	☐	☐
**95.** I have a hot temper	☐	☐	☐
**96.** I think about sex too much	☐	☐	☐
**97.** I threaten to hurt people	☐	☐	☐
**98.** I like to help others	☐	☐	☐
**99.** I am too concerned about being neat or clean	☐	☐	☐
**100.** I have trouble sleeping	☐	☐	☐
**101.** I skip classes or wag school	☐	☐	☐
**102.** I don't have much energy	☐	☐	☐
**103.** I am unhappy, sad or depressed	☐	☐	☐
**104.** I am louder than other kids	☐	☐	☐
**105.** I use alcohol or drugs for nonmedical purposes	☐	☐	☐
**106.** I try to be fair to others	☐	☐	☐
**107.** I enjoy a good joke	☐	☐	☐
**108.** I like to take life easy	☐	☐	☐
**109.** I try to help other people when I can	☐	☐	☐
**110.** I wish I were of the opposite sex	☐	☐	☐
**111.** I keep from getting involved with others	☐	☐	☐
**112.** I worry a lot	☐	☐	☐

Q29 How much do these things go on at your school?

*Please mark **one** response for each item*	Not at all	A Little	Some	Quite a bit	Very much
**a.** Students using drugs before and after school	☐	☐	☐	☐	☐
**b.** Students destroying things (vandalism)	☐	☐	☐	☐	☐
**c.** Students drinking beer/wine/spirits	☐	☐	☐	☐	☐
**d.** Students getting into fights	☐	☐	☐	☐	☐
**e.** Students stealing things	☐	☐	☐	☐	☐
**f.** Students threatening or bullying	☐	☐	☐	☐	☐

Q30 For each of the statements, which is most true for you?

Please mark **one** *response for each item*	**Strongly disagree**	**Disagree**	**Agree**	**Strongly agree**
**a.** I enjoy the work I do at school	☐	☐	☐	☐
**b.** I have lots of friends at my school	☐	☐	☐	☐
**c.** I am motivated to want to learn at my school	☐	☐	☐	☐
**d.** At school I learn things that will be useful to me when I leave	☐	☐	☐	☐
**e.** Learning is fun at my school	☐	☐	☐	☐
**f.** People at my school think a lot of me	☐	☐	☐	☐
**g.** I am keen to do well at school	☐	☐	☐	☐
**h.** What I learn at school will help me get a job when I leave	☐	☐	☐	☐
**i.** I get excited about the work I do at school	☐	☐	☐	☐
**j.** I get on well with others at school	☐	☐	☐	☐
**k.** I think it is worth trying hard at my school work	☐	☐	☐	☐
**l.** What I learn at school is useful to me	☐	☐	☐	☐
**m.** I enjoy being at my school	☐	☐	☐	☐
**n.** I am popular with others at my school	☐	☐	☐	☐
**o.** I want to get good results	☐	☐	☐	☐
**p.** What I learn at school will be useful to me in the future	☐	☐	☐	☐

Q31 How well are these goals being met in your life?

Please mark **one** *response for each item*	***Very poorly*** **0**	**1**	**2**	***Average*** **3**	**4**	**5**	**6**	***Very well*** **7**
**a.** Having a high grade average	☐	☐	☐	☐	☐	☐	☐	☐
**b.** Attending classes regularly	☐	☐	☐	☐	☐	☐	☐	☐
**c.** Doing well even in hard subjects	☐	☐	☐	☐	☐	☐	☐	☐
**d.** Having others think of me as a good student	☐	☐	☐	☐	☐	☐	☐	☐
**e.** Deciding on a future career/education	☐	☐	☐	☐	☐	☐	☐	☐

The next questions are about bullying at school: Bullying is when someone is picked on by another person, or a group of people say nasty and unpleasant things to him or her. It is also when someone is hit, kicked, threatened, sent nasty notes, when no one talks to them and things like that.

Please mark only **one** *response for each question:*

Q32 Have you ever been bullied at school?

- ☐ No → **Go to Q36**
- ☐ Yes

↓

Q33 Has this happened at the school you go to now?

- ☐ No → **Go to Q35**
- ☐ Yes

↓

Q34 Was this in the past three months?

- ☐ No
- ☐ Yes

Q35 How did you feel about being bullied?

- ☐ Made you sad
- ☐ Made you angry
- ☐ Doesn't bother you
- ☐ Stressed you out
- ☐ Other feelings (*Please specify*) ……………………………………

Q36 Have you ever bullied other kids?

- ☐ No
- ☐ Yes

Q37 Are you satisfied with the way the school handles bullying?

- ☐ Very satisfied
- ☐ Fairly satisfied
- ☐ Unsatisfied (they could do a lot more)
- ☐ Very unsatisfactory (nothing is done about it)

SECTION 4 Social Issues Often Faced By Young People

Q38 In some situations we feel sure that we can manage well and make things turn out the way we want; in other situations we feel less sure of managing well and less able to make things turn out the way we want. Please select the response that shows how sure you feel in managing each of the following situations. There are no right or wrong answers - just say what you think would be true for you:

Please mark **one** *response for each item*	**Not at all sure**	**A little sure**	**Some-what sure**	**Quite sure**	**Very sure**
**a.** You meet a person for the first time	☐	☐	☐	☐	☐
**b.** You are in a place you don't know anything about	☐	☐	☐	☐	☐
**c.** You have new work to do at school	☐	☐	☐	☐	☐
**d.** You have to get something done and there is a lot of pressure	☐	☐	☐	☐	☐
**e.** You have to work out a problem with a teacher	☐	☐	☐	☐	☐
**f.** You have to work out a problem with your mother	☐	☐	☐	☐	☐
**g.** You have to give a talk in front of people	☐	☐	☐	☐	☐
**h.** You have to do something for the first time	☐	☐	☐	☐	☐
**i.** You have to travel to a new place by yourself	☐	☐	☐	☐	☐
**j.** You have to work out a problem with a friend	☐	☐	☐	☐	☐
**k.** You have trouble solving a problem in school	☐	☐	☐	☐	☐
**l.** You feel very unhappy	☐	☐	☐	☐	☐
**m.** You lose something important	☐	☐	☐	☐	☐
**n.** You have to do things people expect you to do	☐	☐	☐	☐	☐
**o.** You have to figure out something by yourself	☐	☐	☐	☐	☐
**p.** You have to make an important decision	☐	☐	☐	☐	☐
**q.** Someone counts on you to do something important	☐	☐	☐	☐	☐
**r.** You are bored and want to find something interesting to do	☐	☐	☐	☐	☐
**s.** Things are going wrong	☐	☐	☐	☐	☐
**t.** You become older	☐	☐	☐	☐	☐
**u.** You have to work out a problem with your father	☐	☐	☐	☐	☐
**v.** You have done something wrong	☐	☐	☐	☐	☐

We have much to learn about the actual attitudes, knowledge and experiences of young people. So, your honest responses to the questions in this section will provide valuable information on this important topic. If there is a question you'd prefer not to answer, please skip it, rather than give a false answer. All your answers are CONFIDENTIAL:

Q39 Have you ever smoked even part of a cigarette?

- ☐ No → **Go to Q42**
- ☐ Yes, just a few puffs
- ☐ Yes, I have smoked fewer than 10 cigarettes in my life
- ☐ Yes, I have smoked more than 10 cigarettes in my life

Q40 Have you smoked cigarettes in the <u>past 12 months</u>?

- ☐ No → **Go to Q42**
- ☐ Yes

Q41 Have you smoked cigarettes in the <u>past 4 weeks</u>?

- ☐ No
- ☐ Yes

Q42 Have you ever had even part of an alcoholic drink?

- ☐ No → **Go to Q46**
- ☐ Yes, just a few sips
- ☐ Yes, I have had fewer than 10 alcoholic drinks in my life
- ☐ Yes, I have had more than 10 alcoholic drinks in my life

Q43 Have you ever drunk 6 or more alcoholic drinks at one time or drunk so much alcohol that you threw up (vomited)?

- ☐ Never
- ☐ Yes, once only
- ☐ Yes, more than once

Q44 Have you had an alcoholic drink in the <u>past 12 months</u>?

- ☐ No → **Go to Q46**
- ☐ Yes

Q45 Have you been drunk at any time in the <u>past 6 months</u>?

- ☐ No
- ☐ Yes

Q46 How often do you use any of the following drugs for _non-medical_ purposes?

Please mark **one** *response for each item*	**Never**	**Over 1 year ago**	**Less than monthly**	**About monthly**	**About weekly**	**Daily**	**Don't know**
**a.** Marijuana (mull, grass, pot)	☐	☐	☐	☐	☐	☐	☐
**b.** Inhalants (glue, petrol)	☐	☐	☐	☐	☐	☐	☐
**c.** Pain killers (panadol etc.)	☐	☐	☐	☐	☐	☐	☐
**d.** Amphetamines (Speed, ice, dexis etc.)	☐	☐	☐	☐	☐	☐	☐
**e.** Party drugs (Ecstasy, acid, nangs/nitrous oxide)	☐	☐	☐	☐	☐	☐	☐
**f.** Something else *(Please specify)* …………………………….	☐	☐	☐	☐	☐	☐	☐

SECTION 5 Support and Care

Q47 How much do you feel that...

Please mark **one** *response for each item*	**Not at all**	**Little**	**Some**	**Quite a bit**	**Very much**	**Don't know**
**a.** Adults care about you?	☐	☐	☐	☐	☐	☐
**b.** School / work people care about you?	☐	☐	☐	☐	☐	☐
**c.** Your parents care about you?	☐	☐	☐	☐	☐	☐
**d.** Your friends care about you?	☐	☐	☐	☐	☐	☐
**e.** Church leaders care about you?	☐	☐	☐	☐	☐	☐
**f.** You get upset at home?	☐	☐	☐	☐	☐	☐
**g.** Your family cares about your feelings?	☐	☐	☐	☐	☐	☐
**h.** People in your family understand you?	☐	☐	☐	☐	☐	☐
**i.** You want to leave home?	☐	☐	☐	☐	☐	☐
**j.** You and your family have lots of fun together?	☐	☐	☐	☐	☐	☐
**k.** Your family pays a lot of attention to you?	☐	☐	☐	☐	☐	☐

Q48 Please read the following statements and choose the answer that best describes the way your parents (or step-parents or foster parents) in general acted towards you during the past 6 months: My parents (or step-parents or foster parents) …….

Please mark **one** *response for each item*	**Never**	**Sometimes**	**Often**	**Very often**
**a.** Smile at me	☐	☐	☐	☐
**b.** Soon forget a rule they have made	☐	☐	☐	☐
**c.** Praise me	☐	☐	☐	☐
**d.** Nag me about little things	☐	☐	☐	☐
**e.** Only keep rules when it suits them	☐	☐	☐	☐
**f.** Make sure I know I am appreciated	☐	☐	☐	☐
**g.** Threaten punishment more often than they use it	☐	☐	☐	☐
**h.** Speak of the good things I do	☐	☐	☐	☐
**i.** Enforce a rule or do not enforce a rule depending upon their mood	☐	☐	☐	☐
**j.** Hit me or threaten to do so	☐	☐	☐	☐
**k.** Seem proud of the things I do	☐	☐	☐	☐

Q49 Here is a list of things that happen to people and that people think or feel. Read each sentence carefully, and mark the one word (**Never, Sometimes, Often, or Always**) that describes you best, especially over the past two weeks. There are no right or wrong answers:

Please mark **one** *response for each item*	**Never**	**Sometimes**	**Often**	**Always**
**a.** I think that my life is bad	☐	☐	☐	☐
**b.** I have trouble doing things	☐	☐	☐	☐
**c.** I feel that I am a bad person	☐	☐	☐	☐
**d.** I wish I were dead	☐	☐	☐	☐
**e.** I have trouble sleeping	☐	☐	☐	☐
**f.** I feel no one loves me	☐	☐	☐	☐
**g.** I think bad things happen because of me	☐	☐	☐	☐
**h.** I feel lonely	☐	☐	☐	☐
**i.** My Stomach hurts	☐	☐	☐	☐
**j.** I feel like bad things happen to me	☐	☐	☐	☐

Please mark **one** *response for each item*	Never	Sometimes	Often	Always
**k.** I feel like I am stupid	☐	☐	☐	☐
**l.** I feel sorry for myself	☐	☐	☐	☐
**m.** I think I do things badly	☐	☐	☐	☐
**n.** I feel bad about what I do	☐	☐	☐	☐
**o.** I hate myself	☐	☐	☐	☐
**p.** I want to be alone	☐	☐	☐	☐
**q.** I feel like crying	☐	☐	☐	☐
**r.** I feel sad	☐	☐	☐	☐
**s.** I feel empty inside	☐	☐	☐	☐
**t.** I think my life will be bad	☐	☐	☐	☐

Q50 About how many close friends would you say you have?

None	1 – 2	3 – 4	5 or more
☐	☐	☐	☐

Q51 How important are your friends to you in your life? (Mark the response that fits for you)

Not at all important **Important** **Very Important**

0 ☐ **1** ☐ **2** ☐ **3** ☐ **4** ☐ **5** ☐ **6** ☐

Q52 Date questionnaire completed: ☐☐ / ☐☐ / ☐☐☐☐

THANK YOU

WE APPRECIATE THE TIME THAT YOU HAVE SPENT COMPLETING THIS QUESTIONNAIRE

ID ☐☐☐☐☐

OFFICE USE ONLY

RA-CH RA-CO RA1-E RA2-E

ID

GROWING UP HEALTHY STUDY

Participant Questionnaire

MALE

13-15

The purpose of this questionnaire is to obtain information about a range of topics including your household and neighbourhood, your study, job seeking and work experiences, and your health and wellbeing.

Please read each question carefully and answer all of the questions.
Write your answers clearly in the space provided or mark the most appropriate response

☒

Please take your time in answering all of the questions

If you are uncomfortable about a question or unsure of an answer, please leave it blank or ask one of the Growing Up Healthy Study team for assistance.

Please complete this questionnaire independently (without discussing it with anyone)

Remember all answers are STRICTLY confidential

Questionnaire

What is your date of birth?

☐☐ / ☐☐ / ☐☐☐☐

Are you **MALE**?

☐ Yes
☐ No (Please ask one of the Growing Up Healthy Study team for assistance)

SECTION 1 Physical Education and Physical Activity

Please mark only **one** *response for each question:*

Q1 How do you usually get to and from school?

Car	Bus	Bicycle	Walk	Home schooled
☐	☐	☐	☐	☐

Q2 How many physical education periods do you usually attend at school each week?

None	One per week	Two per week	Three or more per week
☐	☐	☐	☐

Q3 During physical education periods, how much time do you spend exercising that makes you out of breath or sweat?

Not much time or none at all	About a quarter of the time	About half of the time	More than half of the time	Almost all of the time
☐	☐	☐	☐	☐

Q4 What do you feel about your physical education periods?

I like them very much	I like them	I neither like nor dislike them	I dislike them	I dislike them very much	I do not attend them
☐	☐	☐	☐	☐	☐

Q5 Outside school hours: How often do you usually exercise in your free time, so much that you get out of breath or sweat?

Once per month or less	Once per week	2-3 times per week	4-6 times per week	Every day
☐	☐	☐	☐	☐

Q6 Outside school hours: How many hours do you usually exercise in your free time, so much that you get out of breath or sweat?

None	About ½ hour per week	About 1 hour per week	About 2-3 hours per week	About 4-6 hours per week	7 or more hours per week
☐	☐	☐	☐	☐	☐

Q7 On average, how many hours per day do you usually watch TV or videos (including school days and weekends)?

Not at all	Up to one hour per day	1-2 hours per day	2-3 hours per day	4 hours or more per day
☐	☐	☐	☐	☐

Q8 On average, how many hours per week do you usually watch TV or videos (including school days and weekends)?

Not at all	Up to seven hour per week	7-14 hours per week	14-21 hours per week	21 hours or more per week
☐	☐	☐	☐	☐

Q9 On average, how many hours per day do you usually use a computer, e.g. for homework, the Internet or chat online (including school days and weekends)?

Not at all	Up to one hour per day	1-2 hours per day	2-3 hours per day	4 hours or more per day
☐	☐	☐	☐	☐

Q10 On average, how many hours per week do you usually use a computer, e.g. for homework, the Internet or chat online (including school days and weekends)?

Not at all	Up to seven hour per week	7-14 hours per week	14-21 hours per week	21 hours or more per week
☐	☐	☐	☐	☐

Q11 How often do each of the following people play some sort of sport or exercise (e.g., golf, tennis, football) or other activity like walking for exercise, cycling or swimming?

Please mark **one** *response for each item*	I don't have/I don't know	Never or rarely	Sometimes	About once per week	2-3 times each week	More than 3 times per week
Father/male carer	☐	☐	☐	☐	☐	☐
Mother/female carer	☐	☐	☐	☐	☐	☐
Your best friend	☐	☐	☐	☐	☐	☐

Q12 How often do each of the following people praise you or encourage you to play some sort of sport or to participate in other physical activity (e.g., watch you participate, say positive things to you, seem happy to do it)?

Please mark **one** *response for each item*	I don't have/I don't know	Never or rarely	Sometimes	Often	Very often
Father/male carer	☐	☐	☐	☐	☐
Mother/female carer	☐	☐	☐	☐	☐
Your best friend	☐	☐	☐	☐	☐
A teacher at your school	☐	☐	☐	☐	☐

Q13 How often do each of the following people help you to play some sort of sport or to participate in other physical activity (e.g., take you to training, give sport money)?

Please mark **one** *response for each item*	**I don't have/I don't know**	**Never or rarely**	**Sometimes**	**Often**	**Very often**
Father/male carer	☐	☐	☐	☐	☐
Mother/female carer	☐	☐	☐	☐	☐
Your best friend	☐	☐	☐	☐	☐
A teacher at your school	☐	☐	☐	☐	☐

Q14 Below are some reasons for not doing more exercise or activities than you do. Please show how strongly each statement applies to you:

Please mark **one** *response for each item*	**Does not apply at all**	**Applies a little**	**Applies a fair amount**	**Applies strongly**	**Applies very strongly**
**a.** I already do a lot of exercise	☐	☐	☐	☐	☐
**b.** I am self-conscious about my looks when I exercise or play sport	☐	☐	☐	☐	☐
**c.** I don't have enough time	☐	☐	☐	☐	☐
**d.** I don't have enough energy	☐	☐	☐	☐	☐
**e.** There are other things I like doing more	☐	☐	☐	☐	☐
**f.** I don't have anyone to exercise or play with	☐	☐	☐	☐	☐
**g.** I just don't enjoy exercise or sport	☐	☐	☐	☐	☐
**h.** My parents don't encourage or help me	☐	☐	☐	☐	☐
**i.** The right facilities are not available	☐	☐	☐	☐	☐
**j.** I don't have the skills	☐	☐	☐	☐	☐
**k.** I am just not very good at any sports or activities	☐	☐	☐	☐	☐
**l.** Others laugh/make fun of me when I try to play	☐	☐	☐	☐	☐
**m.** My health is not good enough	☐	☐	☐	☐	☐
**n.** I have an injury which prevents me	☐	☐	☐	☐	☐
**o.** Another reason *(please describe)* ……………………………………………………	☐	☐	☐	☐	☐

SECTION 2 Eating Habits, Weight, Physical Health and Development

Q15 How often do you eat the following foods?

Please mark **one** *response for each item*	6 + times a week	3-5 times a week	1-2 times a week	1-2 times a month	Rarely or never
**a.** Fried food with a batter or breadcrumb coating	☐	☐	☐	☐	☐
**b.** Gravy, creamy sauces or cheese sauces	☐	☐	☐	☐	☐
**c.** Vegetables, rice or pasta with added butter, margarine, oil or sour cream	☐	☐	☐	☐	☐
**d.** Vegetables that are fried or roasted with fat or oil (don't count oil sprays)	☐	☐	☐	☐	☐
**e.** Sausages, polony, salami, meat pies, pasties, hamburger or bacon	☐	☐	☐	☐	☐
**f.** Hot potato chips or French fries	☐	☐	☐	☐	☐
**g.** Pastries, cakes, sweet biscuits or croissants	☐	☐	☐	☐	☐
**h.** Chocolate, chocolate biscuits or sweet snack bars	☐	☐	☐	☐	☐
**i.** Potato crisps, corn chips, cheezels, twisties or nuts	☐	☐	☐	☐	☐
**j.** Ice cream (any variety)	☐	☐	☐	☐	☐
**k.** Cream or sour cream	☐	☐	☐	☐	☐
**l.** Cheddar, edam or other hard cheese, cream cheese or soft cheeses such as camembert or brie (but excluding ricotta or cottage cheese)	☐	☐	☐	☐	☐

Q16 How much of the following do you usually eat? (*Please mark* **one** *response for each item)*

a Fat on meat?

☐ Most or all
☐ Some
☐ None
☐ Don't eat meat

b Skin on chicken?

☐ Most or all
☐ Some
☐ None
☐ Don't eat chicken

Q17 How often do you eat the following foods?

Please mark **one** *response for each item*	6 + times a week	3-5 times a week	1-2 times a week	1-2 times a month	Rarely or never
**Fruit**, including fresh and canned fruit (do not include dried fruit, fruit juices, fruit drinks, fruit bars or frozen fruit desserts)	☐	☐	☐	☐	☐
**Vegetables**, including all forms of vegetables, e.g. fresh, frozen, canned and salads	☐	☐	☐	☐	☐

Q18 Here we are asking for some additional information on <u>how often</u> and <u>how much</u> of the following drinks you usually consume.

When answering these questions, please mark how often you have the drink and write the total number of glasses, cans, or cups you would usually drink (see example). To assist you, below each type of drink is the type of measurement.

	Never	less than once/ month	**1** day/ month	**2** days/ month	**3** days/ month	**1** day/ week	**2** days/ week	**3** days/ week	**4** days/ week	**5** days/ week	**6** days/ week	every day	**Total number of glasses/ cups/cans you usually drink**
***i.e.* Water (250 ml glass)**	☐	☐	☐	☐	☐	☐	☐	☐	☐	☐	☒	☐	**8**
**1.** Water (250 ml glass)	☐	☐	☐	☐	☐	☐	☐	☐	☐	☐	☐	☐	
**2.** Fizzy drink (e.g. cola, lemonade) (can, glass)	☐	☐	☐	☐	☐	☐	☐	☐	☐	☐	☐	☐	
**3.**Diet fizzy drink (e.g. diet cola, diet lemonade) (can, glass)	☐	☐	☐	☐	☐	☐	☐	☐	☐	☐	☐	☐	
**4.** Energy drink (e.g. Redbull, V, Monster) (can)	☐	☐	☐	☐	☐	☐	☐	☐	☐	☐	☐	☐	
**5.** Diet energy drink (can)	☐	☐	☐	☐	☐	☐	☐	☐	☐	☐	☐	☐	
**6.** Tea (cup)	☐	☐	☐	☐	☐	☐	☐	☐	☐	☐	☐	☐	
**7.** Herbal tea (cup)	☐	☐	☐	☐	☐	☐	☐	☐	☐	☐	☐	☐	
**8.** Green tea (cup)	☐	☐	☐	☐	☐	☐	☐	☐	☐	☐	☐	☐	
**9.** Instant coffee (cup)	☐	☐	☐	☐	☐	☐	☐	☐	☐	☐	☐	☐	
**10.** Ground coffee (e.g. filter coffee, cappuccino, flat white) (cup, mug)	☐	☐	☐	☐	☐	☐	☐	☐	☐	☐	☐	☐	

Q19 Do you know how much you weigh?

☐ No → **Go to Q21**
☐ Yes

Q20 What is your current weight? ☐☐☐ . ☐ kg

OFFICE USE ONLY
Q18 1☐☐ 2☐☐ 3☐☐ 4☐☐ 5☐☐ 6☐☐ 7☐☐ 8☐☐ 9☐☐ 10☐☐

Please mark only **one** *response for each question below*

Q21 Are you worried about your weight?

No, not at all	A little	Moderately	Very
☐	☐	☐	☐

Q22 Do you consider yourself to be?

Underweight	Normal weight	A bit overweight	Very overweight
☐	☐	☐	☐

Q23 How often do you weigh yourself?

Never	Once in a while	Often	Nearly every day
☐	☐	☐	☐

Please look at the figures and select from the list of numbers and letters A and B provided:

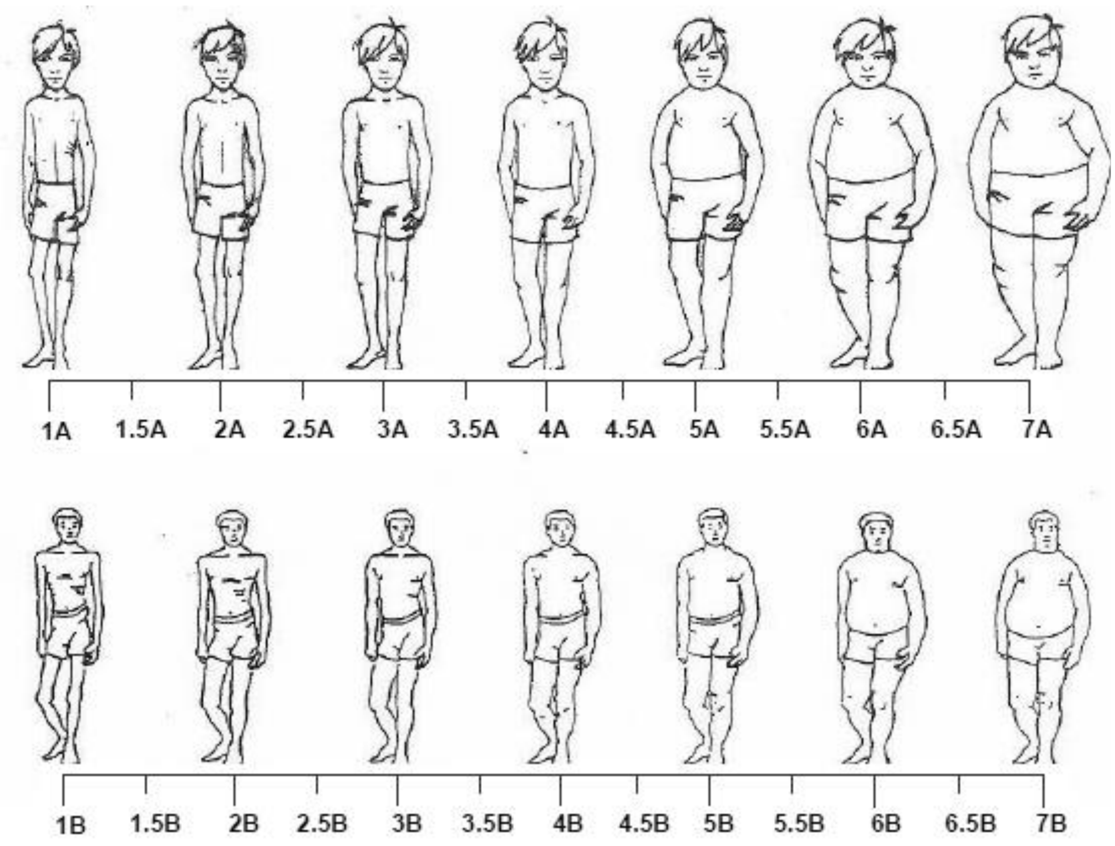

	Number	***Letter***
Q24 Which figure best represents what you currently look like?	☐	☐
Q25 Which figure would you most like to look like?	☐	☐

The drawings on this page show different amounts of male pubic hair and stages of development of the testes, scrotum and penis. A boy passes through each of the four stages shown by these drawings.

Q26 Please look at each drawing then choose the one closest to your stage of development by placing an X in the corresponding box.

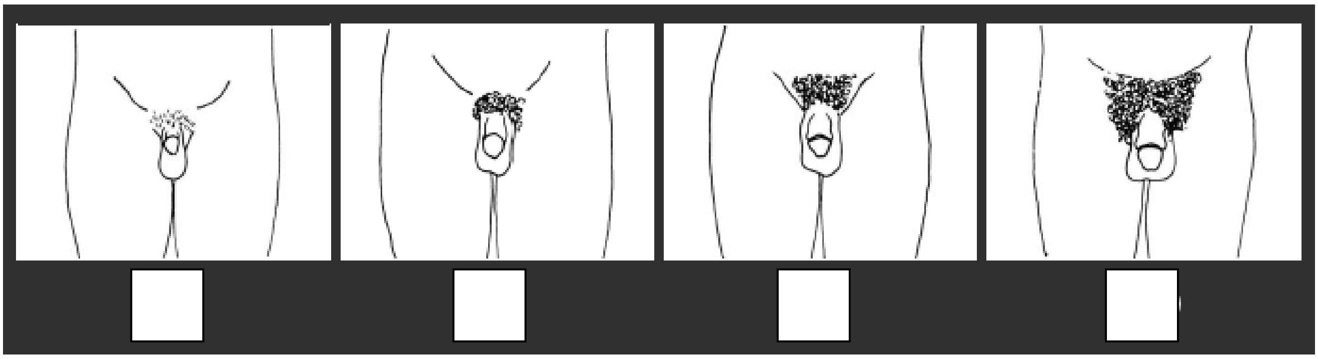

Q27 **Over the last two weeks…**

a. Have you been trying hard to eat less to change your shape or weight (even if you haven't managed to do so)?

**Not at all**	**Some of the time**	**A lot of the time**	**Most of the time**
☐	☐	☐	☐

b. Have you gone for long periods of time (8 hours or more) without eating anything to try and change your shape or weight?

**Not at all**	**Some of the time**	**A lot of the time**	**Most of the time**
☐	☐	☐	☐

c. Have you tried not to eat certain foods (like chocolate or chips to try to change your shape or weight (even if you haven't been able to do so)?

**Not at all**	**Some of the time**	**A lot of the time**	**Most of the time**
☐	☐	☐	☐

d. Have you tried to stick to any definite rules about diet or eating (e.g., sticking to a calorie limit, a set amount of food or rules about what or when you should eat (even if you haven't been able to do so)?

**Not at all**	**Some of the time**	**A lot of the time**	**Most of the time**
☐	☐	☐	☐

e. Have you been thinking about food or calories so much that you've found it hard to concentrate on things you are interested in (e.g., reading, watching TV or following a conversation)?

Not at all	Some of the time	A lot of the time	Most of the time
☐	☐	☐	☐

f. Have there been times when you feel that you have eaten an unusually large amount of food (more than what most people would eat in the same situation)?

Not at all	Some of the time	A lot of the time	Most of the time
☐	☐	☐	☐

g. Have you been afraid of losing control over your eating?

Not at all	Some of the time	A lot of the time	Most of the time
☐	☐	☐	☐

h. Have you felt that you couldn't control what or how much you were eating?

Not at all	Some of the time	A lot of the time	Most of the time
☐	☐	☐	☐

i. Have you felt that you couldn't stop eating once you had started?

Not at all	Some of the time	A lot of the time	Most of the time
☐	☐	☐	☐

j. Have you felt guilty after eating?

Not at all	Some of the time	A lot of the time	Most of the time
☐	☐	☐	☐

k. Have you eaten in secret because you are embarrassed by how much you eat?

Not at all	Some of the time	A lot of the time	Most of the time
☐	☐	☐	☐

l. Have you been afraid that you might gain weight or become fat?

Not at all	Some of the time	A lot of the time	Most of the time
☐	☐	☐	☐

m. Have you felt fat?

Not at all	Some of the time	A lot of the time	Most of the time
☐	☐	☐	☐

n. Have you had a strong desire to lose weight?

Not at all	Some of the time	A lot of the time	Most of the time
☐	☐	☐	☐

o. Have you made yourself sick (vomit) after eating to control your weight?

Not at all	Some of the time	A lot of the time	Most of the time
☐	☐	☐	☐

p. Have you taken any pills (like laxatives, water pills, diet tubes) to try to control your weight?

Not at all	Some of the time	A lot of the time	Most of the time
☐	☐	☐	☐

q. Have you exercised hard to control your weight?

Not at all	Some of the time	A lot of the time	Most of the time
☐	☐	☐	☐

People have different ideas about what sort of things are important to them in how they think about themselves. For some people doing well at school is very important to them, for others, how they are getting on with friends is very important. We're now going to ask you to think about how important weight and shape is to you:

r. Has your <u>weight</u> been important in how you think of yourself as a person?

Not at all	Some of the time	A lot of the time	Most of the time
☐	☐	☐	☐

s. Has your <u>shape</u> been important in how you think of yourself as a person?

Not at all	Some of the time	A lot of the time	Most of the time
☐	☐	☐	☐

SECTION 3 Adolescents and School

Q28 Below is a list of items that describes adolescents. For each item please mark the box that best represents whether the statement is true, somewhat/sometimes true or very true/often true for you now or within the past 6 months. Please answer all items as well as you can, even if some do not seem to apply to you:

Please mark **one** *response for each item*	**Not true**	**Somewhat/ sometimes true**	**Very true/ often true**
**1.** I act too young for my age	☐	☐	☐
**2.** I have an allergy	☐	☐	☐
**3.** I argue a lot	☐	☐	☐
**4.** I have asthma	☐	☐	☐
**5.** I like the opposite sex	☐	☐	☐
**6.** I like animals	☐	☐	☐
**7.** I brag	☐	☐	☐
**8.** I have trouble concentrating or paying attention	☐	☐	☐
**9.** I can't get my mind off certain thoughts	☐	☐	☐
**10.** I have trouble sitting still	☐	☐	☐
**11.** I am too dependent on adults	☐	☐	☐
**12.** I feel lonely	☐	☐	☐
**13.** I feel confused or in a fog	☐	☐	☐
**14.** I cry a lot	☐	☐	☐
**15.** I am pretty honest	☐	☐	☐
**16.** I am mean to others	☐	☐	☐
**17.** I day dream a lot	☐	☐	☐
**18.** I deliberately try to hurt or kill myself	☐	☐	☐
**19.** I try to get a lot of attention	☐	☐	☐
**20.** I destroy my own things	☐	☐	☐
**21.** I destroy things belonging to others	☐	☐	☐
**22.** I disobey my parents	☐	☐	☐
**23.** I disobey at school	☐	☐	☐
**24.** I don't eat as well as I should	☐	☐	☐
**25.** I don't get along with other kids	☐	☐	☐
**26.** I don't feel guilty after doing something I shouldn't	☐	☐	☐
**27.** I am jealous of others	☐	☐	☐
**28.** I am willing to help others when they need help	☐	☐	☐
**29.** I am afraid of certain animals, situations, or places other than school	☐	☐	☐
**30.** I am afraid of going to school	☐	☐	☐

Please mark **one** *response for each item*	**Not true**	**Somewhat/ sometimes true**	**Very true/ often true**
**31.** I am afraid I might think or do something bad	☐	☐	☐
**32.** I feel that I have to be perfect	☐	☐	☐
**33.** I feel that no one loves me	☐	☐	☐
**34.** I feel that others are out to get me	☐	☐	☐
**35.** I feel worthless or inferior	☐	☐	☐
**36.** I accidentally get hurt a lot	☐	☐	☐
**37.** I get in many fights	☐	☐	☐
**38.** I get teased a lot	☐	☐	☐
**39.** I hang around with kids who get in trouble	☐	☐	☐
**40.** I hear sounds or voices that other people think aren't there	☐	☐	☐
**41.** I act without stopping to think	☐	☐	☐
**42.** I would rather be alone than with others	☐	☐	☐
**43.** I lie or cheat	☐	☐	☐
**44.** I bite my fingernails	☐	☐	☐
**45.** I am nervous or tense	☐	☐	☐
**46.** Parts of my body twitch or make nervous movements	☐	☐	☐
**47.** I have nightmares	☐	☐	☐
**48.** I am not liked by other kids	☐	☐	☐
**49.** I can do certain things better than most kids	☐	☐	☐
**50.** I am too fearful or anxious	☐	☐	☐
**51.** I feel dizzy	☐	☐	☐
**52.** I feel too guilty	☐	☐	☐
**53.** I eat too much	☐	☐	☐
**54.** I feel overtired	☐	☐	☐
**55.** I am overweight	☐	☐	☐
**56.** Physical problems without known medical cause: **a.** Aches or pains (not headaches)	☐	☐	☐
**b.** Headaches	☐	☐	☐
**c.** Nausea, feel sick	☐	☐	☐
**d.** Problems with eyes	☐	☐	☐
**e.** Rashes or other skin problems	☐	☐	☐
**f.** Stomach-aches or cramps	☐	☐	☐
**g.** Vomiting, throwing up	☐	☐	☐
**h.** Other *(please describe)*…………………………..	☐	☐	☐

Please mark **one** *response for each item*	Not true	Somewhat/ sometimes true	Very true/ often true
**57.** I physically attack people	☐	☐	☐
**58.** I pick my skin or other parts of my body	☐	☐	☐
**59.** I can be pretty friendly	☐	☐	☐
**60.** I like to try new things	☐	☐	☐
**61.** My school work is poor	☐	☐	☐
**62.** I am poorly coordinated or clumsy	☐	☐	☐
**63.** I would rather be with older kids than kids my own age	☐	☐	☐
**64.** I would rather be with younger kids than kids my own age	☐	☐	☐
**65.** I refuse to talk	☐	☐	☐
**66.** I repeat certain actions over and over	☐	☐	☐
**67.** I run away from home	☐	☐	☐
**68.** I scream a lot	☐	☐	☐
**69.** I am secretive or keep things to myself	☐	☐	☐
**70.** I see things that other people think aren’t there	☐	☐	☐
**71.** I am self-conscious or easily embarrassed	☐	☐	☐
**72.** I set fires	☐	☐	☐
**73.** I can work well with my hands	☐	☐	☐
**74.** I show off or clown	☐	☐	☐
**75.** I am shy	☐	☐	☐
**76.** I sleep less than most kids	☐	☐	☐
**77.** I sleep more than most kids during the day and/or night	☐	☐	☐
**78.** I have a good imagination	☐	☐	☐
**79.** I have a speech problem	☐	☐	☐
**80.** I stand up for my rights	☐	☐	☐
**81.** I steal at home	☐	☐	☐
**82.** I steal from places other than home	☐	☐	☐
**83.** I store things up I don’t need	☐	☐	☐
**84.** I do things other people think are strange	☐	☐	☐
**85.** I have thoughts that other people would think are strange	☐	☐	☐
**86.** I am stubborn	☐	☐	☐
**87.** My moods or feelings change suddenly	☐	☐	☐
**88.** I enjoy being with other people	☐	☐	☐
**89.** I am suspicious	☐	☐	☐
**90.** I swear or use dirty language	☐	☐	☐

*Please mark **one** response for each item*	Not true	Somewhat/ sometimes true	Very true/ often true
**91.** I think about killing myself	☐	☐	☐
**92.** I like to make others laugh	☐	☐	☐
**93.** I talk too much	☐	☐	☐
**94.** I tease others a lot	☐	☐	☐
**95.** I have a hot temper	☐	☐	☐
**96.** I think about sex too much	☐	☐	☐
**97.** I threaten to hurt people	☐	☐	☐
**98.** I like to help others	☐	☐	☐
**99.** I am too concerned about being neat or clean	☐	☐	☐
**100.** I have trouble sleeping	☐	☐	☐
**101.** I skip classes or wag school	☐	☐	☐
**102.** I don't have much energy	☐	☐	☐
**103.** I am unhappy, sad or depressed	☐	☐	☐
**104.** I am louder than other kids	☐	☐	☐
**105.** I use alcohol or drugs for nonmedical purposes	☐	☐	☐
**106.** I try to be fair to others	☐	☐	☐
**107.** I enjoy a good joke	☐	☐	☐
**108.** I like to take life easy	☐	☐	☐
**109.** I try to help other people when I can	☐	☐	☐
**110.** I wish I were of the opposite sex	☐	☐	☐
**111.** I keep from getting involved with others	☐	☐	☐
**112.** I worry a lot	☐	☐	☐

Q29 How much do these things go on at your school?

*Please mark **one** response for each item*	Not at all	A Little	Some	Quite a bit	Very much
**a.** Students using drugs before and after school	☐	☐	☐	☐	☐
**b.** Students destroying things (vandalism)	☐	☐	☐	☐	☐
**c.** Students drinking beer/wine/spirits	☐	☐	☐	☐	☐
**d.** Students getting into fights	☐	☐	☐	☐	☐
**e.** Students stealing things	☐	☐	☐	☐	☐
**f.** Students threatening or bullying	☐	☐	☐	☐	☐

Q30 For each of the statements, which is most true for you?

Please mark **one** *response for each item*	**Strongly disagree**	**Disagree**	**Agree**	**Strongly agree**
**a.** I enjoy the work I do at school	☐	☐	☐	☐
**b.** I have lots of friends at my school	☐	☐	☐	☐
**c.** I am motivated to want to learn at my school	☐	☐	☐	☐
**d.** At school I learn things that will be useful to me when I leave	☐	☐	☐	☐
**e.** Learning is fun at my school	☐	☐	☐	☐
**f.** People at my school think a lot of me	☐	☐	☐	☐
**g.** I am keen to do well at school	☐	☐	☐	☐
**h.** What I learn at school will help me get a job when I leave	☐	☐	☐	☐
**i.** I get excited about the work I do at school	☐	☐	☐	☐
**j.** I get on well with others at school	☐	☐	☐	☐
**k.** I think it is worth trying hard at my school work	☐	☐	☐	☐
**l.** What I learn at school is useful to me	☐	☐	☐	☐
**m.** I enjoy being at my school	☐	☐	☐	☐
**n.** I am popular with others at my school	☐	☐	☐	☐
**o.** I want to get good results	☐	☐	☐	☐
**p.** What I learn at school will be useful to me in the future	☐	☐	☐	☐

Q31 How well are these goals being met in your life?

Please mark **one** *response for each item*	***Very poorly*** **0**	**1**	**2**	***Average*** **3**	**4**	**5**	**6**	***Very well*** **7**
**a.** Having a high grade average	☐	☐	☐	☐	☐	☐	☐	☐
**b.** Attending classes regularly	☐	☐	☐	☐	☐	☐	☐	☐
**c.** Doing well even in hard subjects	☐	☐	☐	☐	☐	☐	☐	☐
**d.** Having others think of me as a good student	☐	☐	☐	☐	☐	☐	☐	☐
**e.** Deciding on a future career/education	☐	☐	☐	☐	☐	☐	☐	☐

The next questions are about bullying at school: Bullying is when someone is picked on by another person, or a group of people say nasty and unpleasant things to him or her. It is also when someone is hit, kicked, threatened, sent nasty notes, when no one talks to them and things like that.

Please mark only **one** *response for each question:*

Q32 Have you ever been bullied at school?

- ☐ No → **Go to Q36**
- ☐ Yes

↓

Q33 Has this happened at the school you go to now?

- ☐ No → **Go to Q35**
- ☐ Yes

↓

Q34 Was this in the past three months?

- ☐ No
- ☐ Yes

Q35 How did you feel about being bullied?

- ☐ Made you sad
- ☐ Made you angry
- ☐ Doesn’t bother you
- ☐ Stressed you out
- ☐ Other feelings (*Please specify*) ……………………………………

Q36 Have you ever bullied other kids?

- ☐ No
- ☐ Yes

Q37 Are you satisfied with the way the school handles bullying?

- ☐ Very satisfied
- ☐ Fairly satisfied
- ☐ Unsatisfied (they could do a lot more)
- ☐ Very unsatisfactory (nothing is done about it)

SECTION 4 Social Issues Often Faced By Young People

Q38 In some situations we feel sure that we can manage well and make things turn out the way we want; in other situations we feel less sure of managing well and less able to make things turn out the way we want. Please select the response that shows how sure you feel in managing each of the following situations. There are no right or wrong answers - just say what you think would be true for you:

Please mark **one** *response for each item*	**Not at all sure**	**A little sure**	**Some-what sure**	**Quite sure**	**Very sure**
**a.** You meet a person for the first time	☐	☐	☐	☐	☐
**b.** You are in a place you don't know anything about	☐	☐	☐	☐	☐
**c.** You have new work to do at school	☐	☐	☐	☐	☐
**d.** You have to get something done and there is a lot of pressure	☐	☐	☐	☐	☐
**e.** You have to work out a problem with a teacher	☐	☐	☐	☐	☐
**f.** You have to work out a problem with your mother	☐	☐	☐	☐	☐
**g.** You have to give a talk in front of people	☐	☐	☐	☐	☐
**h.** You have to do something for the first time	☐	☐	☐	☐	☐
**i.** You have to travel to a new place by yourself	☐	☐	☐	☐	☐
**j.** You have to work out a problem with a friend	☐	☐	☐	☐	☐
**k.** You have trouble solving a problem in school	☐	☐	☐	☐	☐
**l.** You feel very unhappy	☐	☐	☐	☐	☐
**m.** You lose something important	☐	☐	☐	☐	☐
**n.** You have to do things people expect you to do	☐	☐	☐	☐	☐
**o.** You have to figure out something by yourself	☐	☐	☐	☐	☐
**p.** You have to make an important decision	☐	☐	☐	☐	☐
**q.** Someone counts on you to do something important	☐	☐	☐	☐	☐
**r.** You are bored and want to find something interesting to do	☐	☐	☐	☐	☐
**s.** Things are going wrong	☐	☐	☐	☐	☐
**t.** You become older	☐	☐	☐	☐	☐
**u.** You have to work out a problem with your father	☐	☐	☐	☐	☐
**v.** You have done something wrong	☐	☐	☐	☐	☐

We have much to learn about the actual attitudes, knowledge and experiences of young people. So, your honest responses to the questions in this section will provide valuable information on this important topic. If there is a question you'd prefer not to answer, please skip it, rather than give a false answer. All your answers are CONFIDENTIAL:

Q39 Have you ever smoked even part of a cigarette?

- ☐ No → **Go to Q42**
- ☐ Yes, just a few puffs
- ☐ Yes, I have smoked fewer than 10 cigarettes in my life
- ☐ Yes, I have smoked more than 10 cigarettes in my life

Q40 Have you smoked cigarettes in the past 12 months?

- ☐ No → **Go to Q42**
- ☐ Yes

Q41 Have you smoked cigarettes in the past 4 weeks?

- ☐ No
- ☐ Yes

Q42 Have you ever had even part of an alcoholic drink?

- ☐ No → **Go to Q46**
- ☐ Yes, just a few sips
- ☐ Yes, I have had fewer than 10 alcoholic drinks in my life
- ☐ Yes, I have had more than 10 alcoholic drinks in my life

Q43 Have you ever drunk 6 or more alcoholic drinks at one time or drunk so much alcohol that you threw up (vomited)?

- ☐ Never
- ☐ Yes, once only
- ☐ Yes, more than once

Q44 Have you had an alcoholic drink in the past 12 months?

- ☐ No → **Go to Q46**
- ☐ Yes

Q45 Have you been drunk at any time in the past 6 months?

- ☐ No
- ☐ Yes

Q46 How often do you use any of the following drugs for *non-medical* purposes?

Please mark **one** *response for each item*	**Never**	**Over 1 year ago**	**Less than monthly**	**About monthly**	**About weekly**	**Daily**	**Don't know**
**a.** Marijuana (mull, grass, pot)	☐	☐	☐	☐	☐	☐	☐
**b.** Inhalants (glue, petrol)	☐	☐	☐	☐	☐	☐	☐
**c.** Pain killers (panadol etc.)	☐	☐	☐	☐	☐	☐	☐
**d.** Amphetamines (Speed, ice, dexis etc.)	☐	☐	☐	☐	☐	☐	☐
**e.** Party drugs (Ecstasy, acid, nangs/nitrous oxide)	☐	☐	☐	☐	☐	☐	☐
**f.** Something else *(Please specify)* ……………………………	☐	☐	☐	☐	☐	☐	☐

SECTION 5 Support and Care

Q47 How much do you feel that...

Please mark **one** *response for each item*	**Not at all**	**Little**	**Some**	**Quite a bit**	**Very much**	**Don't know**
**a.** Adults care about you?	☐	☐	☐	☐	☐	☐
**b.** School / work people care about you?	☐	☐	☐	☐	☐	☐
**c.** Your parents care about you?	☐	☐	☐	☐	☐	☐
**d.** Your friends care about you?	☐	☐	☐	☐	☐	☐
**e.** Church leaders care about you?	☐	☐	☐	☐	☐	☐
**f.** You get upset at home?	☐	☐	☐	☐	☐	☐
**g.** Your family cares about your feelings?	☐	☐	☐	☐	☐	☐
**h.** People in your family understand you?	☐	☐	☐	☐	☐	☐
**i.** You want to leave home?	☐	☐	☐	☐	☐	☐
**j.** You and your family have lots of fun together?	☐	☐	☐	☐	☐	☐
**k.** Your family pays a lot of attention to you?	☐	☐	☐	☐	☐	☐

Q48 Please read the following statements and choose the answer that best describes the way your parents (or step-parents or foster parents) in general acted towards you during the past 6 months: My parents (or step-parents or foster parents) …….

Please mark **one** *response for each item*	Never	Sometimes	Often	Very often
**a.** Smile at me	☐	☐	☐	☐
**b.** Soon forget a rule they have made	☐	☐	☐	☐
**c.** Praise me	☐	☐	☐	☐
**d.** Nag me about little things	☐	☐	☐	☐
**e.** Only keep rules when it suits them	☐	☐	☐	☐
**f.** Make sure I know I am appreciated	☐	☐	☐	☐
**g.** Threaten punishment more often than they use it	☐	☐	☐	☐
**h.** Speak of the good things I do	☐	☐	☐	☐
**i.** Enforce a rule or do not enforce a rule depending upon their mood	☐	☐	☐	☐
**j.** Hit me or threaten to do so	☐	☐	☐	☐
**k.** Seem proud of the things I do	☐	☐	☐	☐

Q49 Here is a list of things that happen to people and that people think or feel. Read each sentence carefully, and mark the one word (**Never, Sometimes, Often, or Always**) that describes you best, especially over the past two weeks. There are no right or wrong answers:

Please mark **one** *response for each item*	Never	Sometimes	Often	Always
**a.** I think that my life is bad	☐	☐	☐	☐
**b.** I have trouble doing things	☐	☐	☐	☐
**c.** I feel that I am a bad person	☐	☐	☐	☐
**d.** I wish I were dead	☐	☐	☐	☐
**e.** I have trouble sleeping	☐	☐	☐	☐
**f.** I feel no one loves me	☐	☐	☐	☐
**g.** I think bad things happen because of me	☐	☐	☐	☐
**h.** I feel lonely	☐	☐	☐	☐
**i.** My Stomach hurts	☐	☐	☐	☐
**j.** I feel like bad things happen to me	☐	☐	☐	☐

Please mark **one** *response for each item*	Never	Sometimes	Often	Always
**k.** I feel like I am stupid	☐	☐	☐	☐
**l.** I feel sorry for myself	☐	☐	☐	☐
**m.** I think I do things badly	☐	☐	☐	☐
**n.** I feel bad about what I do	☐	☐	☐	☐
**o.** I hate myself	☐	☐	☐	☐
**p.** I want to be alone	☐	☐	☐	☐
**q.** I feel like crying	☐	☐	☐	☐
**r.** I feel sad	☐	☐	☐	☐
**s.** I feel empty inside	☐	☐	☐	☐
**t.** I think my life will be bad	☐	☐	☐	☐

Q50 About how many close friends would you say you have?

None	1 – 2	3 – 4	5 or more
☐	☐	☐	☐

Q51 How important are your friends to you in your life? (Mark the response that fits for you)

Not at all important **Important** **Very Important**

0☐ **1**☐ **2**☐ **3**☐ **4**☐ **5**☐ **6**☐

Q52 Date questionnaire completed: ☐☐ / ☐☐ / ☐☐☐☐

THANK YOU

WE APPRECIATE THE TIME THAT YOU HAVE SPENT COMPLETING THIS QUESTIONNAIRE

ID | | | | | |
