# Supplementary material for: Cohort profile: The Growing Up Healthy Study (GUHS)—A prospective and observational cohort study investigating the long-term health outcomes of offspring conceived after assisted reproductive technologies
Source: PLoS One. 2022 Jul 22;17(7):e0272064. doi: 10.1371/journal.pone.0272064 (PMC9307151; doi:10.1371/journal.pone.0272064)
Supplement: S3 File — (PDF) [file pone.0272064.s004.pdf]

OFFICE USE ONLY

RA-CH ☐☐ RA-CO ☐☐ RA1-E ☐☐ RA2-E ☐☐

ID ☐☐☐☐

GROWING UP HEALTHY STUDY

Primary Caregiver Questionnaire

Part A

16-18

Thank you for giving your time to fill in this questionnaire

Please read each question carefully and answer all questions from ***Part A*** and ***Part B***.
Write your answers clearly in the space provided or mark the most appropriate response

☒

All information will be strictly confidential

Please take your time in answering all of the questions

If you require assistance to answer any of the questions please contact the Study Coordinators:

Blagica & Tina
T: +61 6458 1443
M: 0439 266 434
Email: **guhstudy-swih@uwa.edu.au**

The purpose of this questionnaire is to obtain information about your teenager's home life, leisure activities, schooling, behaviour and general health.

In answering this questionnaire the **Primary Caregiver** is the person who provides the majority of pastoral care for the study teenager(s).

The primary caregiver answering this questionnaire is the teenagers'…

- ☐ Biological mother
- ☐ Biological father
- ☐ Non-biological mother
- ☐ Non-biological father
- ☐ Other person *Please specify:* ....

If you are able to answer questions about your teenagers' other biological parent please do so.

Please complete this questionnaire independently
(without discussing it with your teenagers)

If you are coming in for an appointment, please bring your completed questionnaire with you on the day.

If you are unable to attend an appointment, please use the Reply Paid envelope enclosed to return your completed questionnaire.

If possible, could you please return your completed questionnaire to us by:

☐☐ / ☐☐ / ☐☐☐☐

SECTION 1 Housing and Family - Strictly Confidential

The following questions regarding your residence, past education and current job provide us with useful information about your teenager's home environment at the time of birth and now:

Q1 What is your current residential postcode? ☐☐☐☐

Q2 How many adults and children live in your home?
Please include your study teenager(s) and yourself. Children less than one year of age: Age = 0

First name	Age (years)	Sex (M/F)	Relationship to study teenager
e.g. Elizabeth<br>David<br>Jessica<br>Hannah	42<br>35<br>13<br>2	F<br>M<br>F<br>F	Mother<br>Stepfather<br>Study teenager<br>Stepsister
1			
2			
3			
4			
5			
6			
7			
8			
9			
10			

Q3 Does your study teenager have any other brothers or sisters not living at home?
Please include your study teenager(s) here if they **do not** *live at home*

☐ No → **Go to Q4**
☐ Yes

↓

First name	Age (years)	Sex (M/F)	Relationship to study teenager

Q4 How old were you when you left school? ☐☐

Q5 What was the last year of school that you completed? ☐☐
(e.g. year 10) or equivalent

Q6 Since leaving school have you completed any further education?

- ☐ None
- ☐ Trade certificate, or apprenticeship
- ☐ Professional registration (non-degree e.g. Police)
- ☐ College Diploma or Degree (e.g. TAFE, WAIT, WACAE)
- ☐ University Degree
- ☐ Other *Please specify:*…………………………………………………………..

Q7 Do you currently have a full-time or part-time job of any kind (excluding home duties)?
Please mark only **one** *response- the main job*

- ☐ No, do not have a job - not seeking work → **Go to Q9**
- ☐ No, do not have a job - actively seeking work → **Go to Q9**
- ☐ Yes, do work for payment or profit
- ☐ Yes, do unpaid work in a family business
- ☐ Yes, do other unpaid work

Q8 Describe your current main job.
Please give details of job and description of work

Job title:
Job Description:

Q9 What do you currently spend most of your time doing?
Please mark only **one** *response, unless two or more responses apply equally*

- ☐ Full-time or part-time job (salary or own business)
- ☐ Voluntary work
- ☐ Looking for work
- ☐ Home duties / caring for children
- ☐ Studying
- ☐ Voluntarily out of the workforce
- ☐ Recovering from injury / illness
- ☐ Caring for an aged / disabled / ill person (friend or relative)
- ☐ Other *Please specify:*…………………………………………………….

Q10 Does your partner currently have a full-time or part-time job of any kind (excluding home duties)? *Please mark only* **one** *response - the main job*

- ☐ No Partner → **Go to Section 2**
- ☐ No, does not have a job - not seeking work → **Go to Section 2**
- ☐ No, does not have a job - actively seeking work → **Go to Section 2**
- ☐ Yes, works for payment or profit
- ☐ Yes, does unpaid work in a family business
- ☐ Yes, does other unpaid work

Q11 Describe your partner's current main job.
Please give details of job and description of work

Job title:
Job Description:

SECTION 2 Your Health and Wellbeing - Strictly Confidential

The following questions are about the health and wellbeing of the study teenager's mother and father. We are also interested to know about the health and wellbeing of your partner if the mother/father of your teenager(s) is no longer living with you. We have tried to keep these to a minimum but some things that affect parents may also affect their children:

Q12 Do you currently smoke cigarettes?

- ☐ No → **Go to Q15**
- ☐ Yes

Q13 How many cigarettes do you smoke per day?
Please mark only **one** *response*

- ☐ Less than one
- ☐ 1 - 5
- ☐ 6 - 10
- ☐ 11 - 15
- ☐ 16 - 20
- ☐ More than 20

Q14 Do you smoke inside your house?

- ☐ No
- ☐ Yes

OFFICE USE ONLY

Q8 ☐☐☐☐☐☐ Q11 ☐☐☐☐☐☐

Q15 Does anyone else in your house currently smoke cigarettes?

☐ No → **Go to Q18**
☐ Yes

Q16 How many do they smoke per day **now**?
If more than one person at home smokes, please mark the **total** *number of cigarettes smoked*

☐ Less than one
☐ 1 - 5
☐ 6 - 10
☐ 11 - 15
☐ 16 - 20
☐ More than 20

Q17 Do they smoke inside your house?

☐ No
☐ Yes

Q18 Does anyone in your home smoke/use any other substances?
Please include pipe, cigars, marijuana, other drugs, etc.

☐ No → **Go to Q19**
☐ Yes

Which other substances do they smoke/use?

Please mark **one** *response for each item*	**No**	**Once a week or less**	**More than once weekly but not everyday**	**Everyday**
Pipe	☐	☐	☐	☐
Cigars	☐	☐	☐	☐
Marijuana	☐	☐	☐	☐
Other<br>*Please specify:*……………………………………	☐	☐	☐	☐

SECTION 3 Your Physical Activity and Health - Strictly Confidential

The following questions relate to your usual leisure-time physical activity habits:

Q19 During a typical 7 day period (a week), how many times on average do you do the following ***kinds of exercise*** for more than 15 minutes during your free time.

Please write in each box the appropriate number — Times per week

a. Strenuous exercise (heart beats rapidly) ☐☐

(e.g., running, jogging, hockey, football, soccer, squash, basketball, judo, roller skating, vigorous swimming, vigorous long distance bicycling)

b. Moderate exercise (not exhausting) ☐☐

(e.g., fast walking, baseball, tennis, easy bicycling, volleyball, badminton, easy swimming, dancing)

c. Mild exercise (minimal effort) ☐☐

(e.g., yoga, archery, fishing from land, bowling, golf, easy walking)

Q20 During a typical 7 day period (a week), in your leisure time, how often do you engage in ***any regular activity*** long enough to work up a sweat (heart beats rapidly)?

- ☐ Often
- ☐ Sometimes
- ☐ Never/Rarely

Q21 Are you worried about your weight?

- ☐ Not at all
- ☐ A little
- ☐ Moderately
- ☐ Very

Q22 Do you consider yourself to be…

- ☐ Underweight?
- ☐ Normal weight?
- ☐ A bit overweight?
- ☐ Very overweight?

The following series of questions relate to your levels of depression, anxiety and stress and provide us with valuable information about your emotional wellbeing:

Q23 Please read each statement and mark the response which indicates how much the statement applied to you over the past week. There are no right or wrong answers. Do not spend too much time on any one statement:

The rating scale is as follows:

0 = Did not apply to me at all - Never
1 = Applied to me to some degree, or some of the time - Sometimes
2 = Applied to me a considerable degree, or a good part of the time - Often
3 = Applied to me very much, or most of the time – Almost always

Please mark **one** *response for each item*	**0**	**1**	**2**	**3**
**a.** I found it hard to wind down	☐	☐	☐	☐
**b.** I was aware of dryness of my mouth	☐	☐	☐	☐
**c.** I couldn't seem to experience any positive feeling at all	☐	☐	☐	☐
**d.** I experienced breathing difficulty (e.g., excessively rapid breathing, breathlessness in the absence of physical exertion)	☐	☐	☐	☐
**e.** I found it difficult to work up the initiative to do things	☐	☐	☐	☐
**f.** I tended to over-react to situations	☐	☐	☐	☐
**g.** I experienced trembling (e.g., in the hands)	☐	☐	☐	☐
**h.** I felt that I was using a lot of nervous energy	☐	☐	☐	☐
**i.** I was worried about situations in which I might panic and make a fool of myself	☐	☐	☐	☐
**j.** I felt that I had nothing to look forward to	☐	☐	☐	☐
**k.** I found myself getting agitated	☐	☐	☐	☐
**l.** I found it difficult to relax	☐	☐	☐	☐
**m.** I felt down-hearted and blue	☐	☐	☐	☐
**n.** I was intolerant of anything that kept me from getting on with what I was doing	☐	☐	☐	☐
**o.** I felt I was close to panic	☐	☐	☐	☐
**p.** I was unable to become enthusiastic about anything	☐	☐	☐	☐
**q.** I felt I wasn't worth much as a person	☐	☐	☐	☐
**r.** I felt that I was rather touchy	☐	☐	☐	☐
**s.** I was aware of the action of my heart in the absence of physical exertion (e.g., sense of heart rate increase, heart missing a beat)	☐	☐	☐	☐
**t.** I felt scared without any good reason	☐	☐	☐	☐
**u.** I felt that life was meaningless	☐	☐	☐	☐

Q24 Does this reflect a typical a week for you?

☐ Yes → **Go to Q25**
☐ No

Please describe the reason(s) for this not being a typical a week for you:

Q25 Have any of the following happened to you in the past year?

Please mark **Yes** *or* **No** *for each item*	**Yes**	**No**
Pregnancy problems	☐	☐
Death of a close relative *Which relative?...*	☐	☐
Death of a close friend	☐	☐
Separation or divorce	☐	☐
Marital problems	☐	☐
Problems with your children	☐	☐
Your own job loss (not voluntary)	☐	☐
Your partner's job loss (not voluntary)	☐	☐
Money problems	☐	☐
Residential move	☐	☐
Other *Please describe: ....*	☐	☐

This is called the Family Assessment Device. It was developed to give an idea of how families work together.

Q26 Below are statements about families and family relationships. Mark the statement which best describes your family (the people living in your house):

Please mark **one** *response for each item*	**Strongly Agree**	**Agree**	**Disagree**	**Strongly Disagree**
**a.** Planning family activities is difficult because we misunderstand each other	☐	☐	☐	☐
**b.** In times of crisis we can turn to each other for support	☐	☐	☐	☐
**c.** We cannot talk to each other about sadness we feel	☐	☐	☐	☐
**d.** Individuals (in the family) are accepted for what they are	☐	☐	☐	☐
**e.** We avoid discussing our fears and concerns	☐	☐	☐	☐
**f.** We express feelings to each other	☐	☐	☐	☐
**g.** There are lots of bad feelings in our family	☐	☐	☐	☐
**h.** We feel accepted for what we are	☐	☐	☐	☐
**i.** Making decisions is a problem in our family	☐	☐	☐	☐
**j.** We are able to make decisions about how to solve problems	☐	☐	☐	☐
**k.** We don't get on well together	☐	☐	☐	☐
**l.** We confide in each other	☐	☐	☐	☐
**m.** Drinking is a source of tension or disagreement in our family	☐	☐	☐	☐

SECTION 4 Housing Environment

Q27 Is your home air-conditioned?

- ☐ No → **Go to Q30**
- ☐ Yes

Q28 How many rooms are air-conditioned?

- ☐ One room
- ☐ Two rooms
- ☐ Three rooms
- ☐ More than three rooms
- ☐ Portable air-conditioner

Q29 Is it evaporative air-conditioning? or Is it refrigerated air-conditioning?

Is it evaporative air-conditioning?

- ☐ No
- ☐ Yes

Is it refrigerated air-conditioning?

- ☐ No
- ☐ Yes

Q30 Is your home heated?

- ☐ No → **Go to Q31**
- ☐ Yes

How is your home heated?

Please mark **all** *applicable responses*	**Yes**	**Is there are chimney (flue) attached?** Yes	No
Gas heater	☐	☐	☐
Kerosene heater	☐	☐	☐
Electric bar radiator, fan or column heater	☐		
Reverse cycle air-conditioning	☐		
Fully ducted heating	☐		
Wood fire/slow combustion heater	☐		
Other *Please specify……………………………………*	☐		

Q31 Do you have gas cooking in your home?

- ☐ No
- ☐ Yes

Q32 Are there any pets at home?

☐ No → **Go to Q33**
☐ Yes

How many pets are there?

If you have no pets at home then please leave this section blank. If you have either no cats, no dogs or birds or no other pets, then print the number 0 in the spaces.

Please print the number in the space provided	**Inside**	**Outside**	**Total**
Cats	☐☐	☐☐	☐☐
Dogs	☐☐	☐☐	☐☐
Birds (include ducks, geese, chickens)	☐☐	☐☐	☐☐

Other pets?

How many other pets inside? ☐☐ What type? ……………………………………………………

How many other pets outside? ☐☐ What type? ……………………………………………………

Q33 Date questionnaire completed: ☐☐ / ☐☐ / ☐☐☐☐

End of Part A

Please continue to Part B – Your Teenager

If you have more than one child participating in the study please complete a separate Part B for each of your study teenagers

THANK YOU

WE APPRECIATE THE TIME THAT YOU HAVE SPENT COMPLETING THIS QUESTIONNAIRE

ID ☐☐☐☐

OFFICE USE ONLY
RA-CH RA-CO RA1-E RA2-E

ID

GROWING UP HEALTHY STUDY

Primary Caregiver Questionnaire

Part B

16-18

YOUR TEENAGER

Please answer the following sections in relation to your study teenager:

....

Q1 On average, how much time is spent with your teenager each day from Monday to Friday (only count the time you spend interacting with each other, helping with homework, talking and just "being together" - excluding sleeping)?

Please mark **one** *response for each item*

	None	Less than 1 hour	About 1 hour	About 1 to 3 hours	About 3 to 5 hours	More than 5 hours
Teen's Mother	☐	☐	☐	☐	☐	☐
Teen's Father	☐	☐	☐	☐	☐	☐
Your Partner/Defacto	☐	☐	☐	☐	☐	☐

Q2 On average, how much time is spent with your teenager each day on the weekend (only count the time you spend interacting with each other, helping with homework, talking and just "being together" - excluding sleeping)?

Please mark **one** *response for each item*

	None	Less than 1 hour	1 to 6 hours	6 to 10 hours	11 to 20 hours
Teen's Mother	☐	☐	☐	☐	☐
Teen's Father	☐	☐	☐	☐	☐
Your Partner/Defacto	☐	☐	☐	☐	☐

Q3 Compared with other children how easy or difficult is your teenager to manage?
Please mark the **one** *response that best represents your feelings*

- ☐ Much more difficult than average
- ☐ A little more difficult than average
- ☐ Average
- ☐ A little easier than average
- ☐ Much easier than average

Q4 How would you compare the physical activity level of your teenager with that of other teenagers of the same age?

- ☐ I am unable to make the comparison
- ☐ My teenager is less active than other teenagers
- ☐ My teenager is as active as other teenagers
- ☐ My teenager is more active than other teenagers

Q5 How does your teenager's level of activity now compare to 12 months ago?

- ☐ Less active than 12 months ago
- ☐ About the same as 12 months ago
- ☐ More active than 12 months ago

OFFICE USE ONLY

Q7 ☐☐☐☐

Q6 What does your teenager currently spend most of his/her time doing?
*Please only mark only **one** response, unless two or more answers apply equally*

- ☐ Studying – school → **Go to Q7**
- ☐ Studying - TAFE
- ☐ Studying – University
- ☐ Studying - Other

→ **Go to Q14** (Studying - TAFE / University / Other)

- ☐ Looking for work
- ☐ Full-time or part-time job (salary or own business)
- ☐ Voluntary work
- ☐ Home duties / caring for children
- ☐ Voluntarily out of the workforce
- ☐ Recovering from injury / illness
- ☐ Caring for an aged / disabled / ill person (relative / friend)
- ☐ Other *Please describe:*……………………………………….

→ **Go to Q17** (Looking for work through Other)

SCHOOL

Q7 What is the name and suburb of the school your teenager is currently attending?

School: …………………………………………………… Suburb: ……………………..………………….

Q8 What year/grade is your teenager in at school now? ☐☐ year/grade

Q9 Is your teenager boarding at school?

- ☐ No
- ☐ Yes

Q10 Has your teenager ever repeated a year/grade at school?

- ☐ No
- ☐ Yes *Which year(s)/grade(s)?*..

Q11 How satisfied are you with the standard of education offered at your teenager's current school?

- ☐ Very dissatisfied
- ☐ Dissatisfied
- ☐ Neither satisfied or dissatisfied
- ☐ Satisfied
- ☐ Very satisfied

Q12 How would you describe your teenager's academic performance in school during the past 6 months?

- ☐ Poor
- ☐ Below average
- ☐ Average
- ☐ Very good
- ☐ Excellent

Q13 How satisfied are you with your teenager's progress at school in the following areas?

*Please mark **one** response for each item*	**Very Satisfied**	**Satisfied**	**Neither**	**Dissatisfied**	**Very Dissatisfied**
Having a high grade average	☐	☐	☐	☐	☐
Attending classes regularly	☐	☐	☐	☐	☐
Doing well even in hard subjects	☐	☐	☐	☐	☐
Having others think of them as a good student	☐	☐	☐	☐	☐
Deciding on a future career/education	☐	☐	☐	☐	☐

→ Go to Q21

TAFE / UNI

Q14 How satisfied are you with the standard of education offered at your teenager's current TAFE or Uni?

- ☐ Very dissatisfied
- ☐ Dissatisfied
- ☐ Neither satisfied or dissatisfied
- ☐ Satisfied
- ☐ Very satisfied

Q15 How would you describe your teenager's academic performance in TAFE or Uni during the past 6 months?

- ☐ Poor
- ☐ Below average
- ☐ Average
- ☐ Very good
- ☐ Excellent

Q16 How satisfied are you with your teenager's progress at TAFE or Uni in the following areas?

Please mark **one** *response for each item*	**Very Satisfied**	**Satisfied**	**Neither**	**Dissatisfied**	**Very Dissatisfied**
Having a high grade average	☐	☐	☐	☐	☐
Attending classes regularly	☐	☐	☐	☐	☐
Doing well even in hard subjects	☐	☐	☐	☐	☐
Having others think of them as a good student	☐	☐	☐	☐	☐
Deciding on a future career/ education	☐	☐	☐	☐	☐
**→ Go to Q21**					

WORK

Q17 In which month and year did your teenager leave school?

Month ☐☐ Year ☐☐☐☐

Q18 What was the highest year of school your teenager completed?

- ☐ Year 12 (or equivalent)
- ☐ Year 11 (or equivalent)
- ☐ Year 10 (or equivalent)
- ☐ Other *(please specify):* ………………………………………………………

Q19 How would you describe your teenager's performance at work or job seeking?

- ☐ Poor
- ☐ Below average
- ☐ Average
- ☐ Very good
- ☐ Excellent

Q20 How satisfied are you with your teenager's...

Please mark **one** *response for each item*	**Very Satisfied**	**Satisfied**	**Neither**	**Dissatisfied**	**Very Dissatisfied**
Decision not to study at this time?	☐	☐	☐	☐	☐
Current work or job seeking or other?	☐	☐	☐	☐	☐

Q21 Is your teenager limited in the kind or amount of school work he/she does because of physical problems?

- ☐ No → **Go to Q23**
- ☐ Yes

Q22 How long has your teenager been limited in this way?

- ☐ Less than 6 months
- ☐ 6 months to 2 years
- ☐ More than 2 years

Q23 Is your teenager limited in the kind or amount of school work he/she does because of emotional problems?

- ☐ No → **Go to Q25**
- ☐ Yes

Q24 How long has your teenager been limited in this way?

- ☐ Less than 6 months
- ☐ 6 months to 2 years
- ☐ More than 2 years

Q25 Is your teenager limited in the kind or amount of school work he/she does because of learning problems?

- ☐ No → **Go to Q27**
- ☐ Yes

Q26 How long has your teenager been limited in this way?

- ☐ Less than 6 months
- ☐ 6 months to 2 years
- ☐ More than 2 years

Q27 Is your teenager limited in the kind or amount of school work he/she does because of speech and/or language problems?

- ☐ No → **Go to Q29**
- ☐ Yes

⇓

Q28 How long has your teenager been limited in this way?

- ☐ Less than 6 months
- ☐ 6 months to 2 years
- ☐ More than 2 years

Q29 Does your teenager take part in any of the following activities outside of school / TAFE / Uni / work hours?

Please mark **all** *applicable responses*	**Yes**
Organised groups such as scouts, guides, church groups	☐
Organised sport like football, netball, little athletics	☐
Informal sporting activities like swimming, rollerblading	☐
Music, art, drama, dance	☐
Informal recreation like going to the movies or swimming pool	☐
Going to friend's houses (any friends, not necessarily school friends)	☐
None of these	☐

Q30 How satisfied are you with the opportunities that your teenager has to take part in activities outside school / TAFE / Uni / work?

- ☐ Very dissatisfied
- ☐ Dissatisfied
- ☐ Neither satisfied or dissatisfied
- ☐ Satisfied
- ☐ Very satisfied

Q31 How would you rate the overall health of your teenager?

- ☐ Poor (seldom well)
- ☐ So-so (he/she is ill as often as he/she is well)
- ☐ OK, could be better (mostly well)
- ☐ Excellent (nearly always well)

Q32 Is your teenager limited in any physical activities (e.g. running, biking, climbing stairs, lifting, dressing) because of health problems?

- ☐ No → **Go to Q34**
- ☐ Yes

⇓

Q33 How long has your teenager been limited in this way?

- ☐ Less than 6 months
- ☐ 6 months to 2 years
- ☐ More than 2 years

Q34 Does your study teenager have now, or has he/she had in the past, any of the following health professional diagnosed medical conditions or health problems?

Please mark **one** *response for each item*	**No**	**Yes, in the past**	**Yes, now**	**Yes, now and in the past**
Acne	☐	☐	☐	☐
Anxiety problems	☐	☐	☐	☐
Arthritis or joint problems	☐	☐	☐	☐
Asthma	☐	☐	☐	☐
Attentional problems	☐	☐	☐	☐
Back pain	☐	☐	☐	☐
Behavioural problems	☐	☐	☐	☐
Bladder control problems	☐	☐	☐	☐
Chronic respiratory or breathing problems (other than asthma)	☐	☐	☐	☐
Coeliac disease	☐	☐	☐	☐
Co-ordination or clumsiness difficulties	☐	☐	☐	☐
Depression	☐	☐	☐	☐
Developmental disorder (e.g. attention deficit disorder, autism, intellectual disability)	☐	☐	☐	☐
Diabetes	☐	☐	☐	☐
Eating disorder/weight problems	☐	☐	☐	☐
Hayfever or some other allergy	☐	☐	☐	☐
Hearing impairment or deafness	☐	☐	☐	☐
Heart condition	☐	☐	☐	☐
Hemochromatosis (iron overload disease)	☐	☐	☐	☐
Intellectual disability	☐	☐	☐	☐
Learning problems	☐	☐	☐	☐
Menstrual problems	☐	☐	☐	☐
Migraine or severe headache	☐	☐	☐	☐
Neck pain	☐	☐	☐	☐
Sleep disturbance	☐	☐	☐	☐
Speech and/or language problems	☐	☐	☐	☐
Thyroid gland problems	☐	☐	☐	☐
Vision problems	☐	☐	☐	☐
Any other medical condition or health problem not mentioned here	☐	☐	☐	☐

Q35 If you have answered "Yes" to any of the above, or your teenager has any other health professional diagnosed problem or condition please describe the condition or problem below in more detail:

Please list every medical condition/health problem separately - otherwise leave this blank

What condition/problem?	Who diagnosed it?	When was it diagnosed?	Treatment
e.g. Impacted wisdom teeth	*Dentist*	*6 months ago*	*Referral to dental surgeon, antibiotics*

Q36 Has your teenager had any accidents or injuries which required you to take him/her to a doctor (GP), hospital or clinic?

- ☐ No → **Go to Q37**
- ☐ Yes

Please describe the accident, the injury and any treatment (e.g. fell off bike, cut arm, 3 stitches), and list every accident/injury separately, giving as much detail as possible

Injury	How did it happen?	When did it happen?	Treatment
e.g. Sprained wrist	*Fell down stairs*	*3 months ago*	*Physiotherapy/bandage*

OFFICE USE ONLY

Q35

1

5

9

13

Q36 1 4

2 5

3

Q37 1 / /

2 / /

3 / /

4 / /

5 / /

Q37 Has your teenager ever been admitted to hospital/day surgery?

- ☐ No → **Go to Q38**
- ☐ Yes

Please list each admission separately, giving as much detail as possible

Date	Which hospital?	Reason for admission
e.g. October 2005	*McCourt St Day Surgery*	*Removal of impacted wisdom teeth*

Q38 Has your teenager ever attended the School Dental Service in Western Australia (this includes dental vans visiting schools)?

- ☐ No
- ☐ Yes
- ☐ Don't know

Q39 Has your teenager attended any of the following in the past 12 months?

- ☐ No → **Go to Q40**
- ☐ Yes

Please mark **all** *appropriate responses applicable to your teenager*

	**No**	**Yes** Now completed	**Yes** Still attending regularly/occasionally
GP or family doctor	☐	☐	☐
Accident and emergency	☐	☐	☐
Hospital outpatient (department or clinic)	☐	☐	☐
Private medical specialist	☐	☐	☐
Dentist/Dental therapist/Orthodontist	☐	☐	☐
School nurse	☐	☐	☐
Optician/Optometrist	☐	☐	☐
Dietician/Nutritionist	☐	☐	☐
Physiotherapist	☐	☐	☐
Occupational therapist (OT)	☐	☐	☐
Speech therapist	☐	☐	☐
Psychologist/Psychiatrist	☐	☐	☐
Podiatrist	☐	☐	☐
Chiropractor	☐	☐	☐
Alternative therapist (e.g. iridologist)	☐	☐	☐

Q40 To your knowledge, has your teenager taken/used any prescription medication(s) in the past 6 months?

☐ No → **Go to Q41**
☐ Yes

Which medication(s)?

Name	Reason for medication	Is he/she still taking the medication?
e.g. Antibiotics<br>*Ventolin*<br>*Cortisone cream*	*For acne*<br>*For asthma*<br>*For eczema*	*Yes*<br>*Yes*<br>*No*

Q41 In the past 6 months has your teenager taken/used any 'over the counter' medication(s) (including vitamins, minerals and health food products)?

☐ No → **Go to Q42**
☐ Yes

Which medication(s)?

Name	Reason for medication	Is he/she still taking the medication?
e.g. Neurofen<br>*Antihistamine*<br>*Fish oil capsules*	*For period pain*<br>*For hayfever*<br>*For ADD*	*Yes*<br>*No*<br>*Yes*

Q42 Does your study teenager smoke?

☐ No, definitely not
☐ No, not as far as I know
☐ Yes
☐ Don't know

OFFICE USE ONLY

Q40 1 10 20

Q41 1 10 20

WHEEZE

Q43 Does your teenager usually cough when he/she gets a cold these days?

- ☐ No
- ☐ Yes
- ☐ Don't know

Q44 Does your teenager seem congested or bring up phlegm (spit) from his/her chest with colds?

- ☐ No
- ☐ Yes
- ☐ Don't know

Q45 Has your teenager wheezed at any time in his/her life? (wheeze is a whistling or rattling noise in the chest, best heard when breathing out)

- ☐ No → **Go to Q51**
- ☐ Yes

Q46 Has your teenager wheezed in the past 12 months?

- ☐ No → **Go to Q51**
- ☐ Yes

Q47 How many episodes of wheezing has your teenager had in the past 12 months?

- ☐ 1 to 2
- ☐ 3 to 12
- ☐ More than 12

In the past 12 months…

Q48 How often, on average has your teenager's sleep been disturbed due to wheezing?

- ☐ Never woken with wheezing
- ☐ Less than one night per week
- ☐ One or more nights per week
- ☐ Don't know

Q49 Has wheezing ever been severe enough to limit your teenager's speech to only one or two words at a time between breaths?

- ☐ No
- ☐ Yes
- ☐ Don't know

Q50 Has your teenager's chest sounded wheezy during or after exercise?

- ☐ No
- ☐ Yes
- ☐ Don't know

ASTHMA

Q51 Do you think your teenager has ever had asthma?

☐ No
☐ Yes
☐ Don't know

Q52 Has a doctor (GP, pediatrician, respiratory specialist) ever told you that your teenager has asthma?

☐ No
☐ Yes

Q53 Does your teenager still have asthma?

☐ Not applicable (never had asthma)
☐ No
☐ Yes
☐ Don't know

Q54 Has your teenager taken/used any of the following asthma medications in the <u>past 12 months</u>?

☐ No → **Go to Q55**
☐ Yes

		Ordered by a Doctor	
Please mark **all** *appropriate answers*	**Yes**	**Yes**	**No**
Ventolin (Asmol, Airomir, etc.)	☐	☐	☐
Respolin	☐	☐	☐
Nuelin	☐	☐	☐
Theo-dur	☐	☐	☐
Bricanyl	☐	☐	☐
Alupent	☐	☐	☐
Atrovent	☐	☐	☐
QVAR	☐	☐	☐
Flixotide	☐	☐	☐
Pulmacort	☐	☐	☐
Berotec	☐	☐	☐
OXIS	☐	☐	☐
Serevent	☐	☐	☐
Singlulaire	☐	☐	☐
Accolate	☐	☐	☐
Seretide	☐	☐	☐
Symbacort	☐	☐	☐
Prednisolone	☐	☐	☐
Other *(please specify):* ……………........………………………………………………	☐	☐	☐

The following questions are about problems which occurred when your teenager DID NOT have a cold or flu:

RHINITIS (runny or blocked nose - including hayfever)

Q55 Has your teenager ever had a problem with sneezing or a runny or blocked nose (including hayfever) when he/she DID NOT have a cold or the flu?

- ☐ No → **Go to Q63**
- ☐ Yes

Q56 In the past 12 months, has your teenager had a problem with sneezing or a runny or blocked nose (including hayfever) when he/she DID NOT have a cold or flu?

- ☐ No → **Go to Q60**
- ☐ Yes

Q57 In the past 12 months, was this nose problem accompanied by itchy-watery eyes?

- ☐ No
- ☐ Yes

Q58 In the past 12 months, how many episodes of allergic nose problem has your teenager had (including hayfever)?

- ☐ 1 to 2
- ☐ 3 to 12
- ☐ More than 12

Q59 In which of the past 12 months did this problem occur?
Please mark **all** *months which apply*

☐ January	☐ July
☐ February	☐ August
☐ March	☐ September
☐ April	☐ October
☐ May	☐ November
☐ June	☐ December

Q60 Has a doctor (GP, pediatrician, respiratory specialist) ever told you that your teenager has an allergic nose problem (including hayfever)?

- ☐ No
- ☐ Yes

Q61 What was the trigger/cause of these episodes?
Please mark **all** *responses that apply*

- ☐ Grass
- ☐ Pollen
- ☐ Animal
- ☐ Dust
- ☐ Other *(please specify):* …………………………………………………
- ☐ Don't know

Q62 Has your teenager taken/used any medication for an allergic nose problem (including hayfever) in the past 12 months?

- ☐ No → **Go to Q63**
- ☐ Yes

Please list the medication and indicate if it was prescription or non-prescription.

**Type of medication**	**Not Prescribed by doctor**	**Prescribed by doctor**
Steroid nasal spray *(please specify):*	☐	☐
Non-steroid nasal spray *(please specify):*	☐	☐
Antihistamine drops/tablets *(please specify):*	☐	☐
Other *non-prescription* medication *(please specify):*	☐	
Other *prescription* medication *(please specify):*		☐

ALLERGIC CONJUNCTIVITIS (itchy water eyes - including hayfever)

Q63 Has your teenager ever had a problem with red/watery or itchy eyes?

- ☐ No → **Go to Q71**
- ☐ Yes

Q64 Do you think your teenager has ever had allergic reaction in the eyes (including hayfever)?

- ☐ No
- ☐ Yes
- ☐ Don't know

Q65 Has a doctor (GP, pediatrician, respiratory specialist) ever told you that your teenager had an allergic reaction in the eyes (including hayfever)?

- ☐ No
- ☐ Yes
- ☐ Don't know

Q66 In the past 12 months, has your teenager suffered from an allergic reaction in the eyes (including hayfever)?

- ☐ No → **Go to Q71**
- ☐ Yes

Q67 In the past 12 months, how many episodes of allergic reaction in the eyes has your teenager had (including hayfever)?

- ☐ 1 to 2
- ☐ 3 to 12
- ☐ More than 12

Q68 In which of the past 12 months did this problem occur?
*Please mark **all** months which apply*

- ☐ January
- ☐ February
- ☐ March
- ☐ April
- ☐ May
- ☐ June
- ☐ July
- ☐ August
- ☐ September
- ☐ October
- ☐ November
- ☐ December

Q69 What was the trigger/cause of these episodes?
*Please mark **all** responses that apply*

- ☐ Grass
- ☐ Pollen
- ☐ Animal
- ☐ Dust
- ☐ Other *(please specify):* …………………..………………………………………
- ☐ Don't know

Q70 Has your teenager taken/used any medication for an allergic eye reaction (including hayfever) in the past 12 months?

- ☐ No → **Go to Q120**
- ☐ Yes

Please list the medication and indicate if it was prescription or non-prescription.

**Type of medication**	**Not Prescribed by doctor**	**Prescribed by doctor**
Eye drops *(please specify):*	☐	☐
Steroid tablets *(please specify):*	☐	☐
Antihistamine drops/tablets *(please specify):*	☐	☐
Other *non-prescription* medication *(please specify):*	☐	
Other *prescription* medication *(please specify):*		☐

ECZEMA (itchy rash)

Q71 Has your teenager <u>ever</u> had eczema or an itchy rash which was coming and going for at least 6 months?

- ☐ No → **Go to Q82**
- ☐ Yes

↓

Q72 Has this eczema / itchy rash at any time affected any one of the following places; the folds of the elbows, behind the knees, in front of the ankles, under the buttocks, or around the neck, ears, or eyes?

- ☐ No
- ☐ Yes

Q73 Has your teenager had this eczema/itchy rash in the <u>past 12 months</u>?

- ☐ No → **Go to Q76**
- ☐ Yes

↓

Q74 In the <u>past 12 months</u>, how often, on average, has your teenager been kept awake at night by this itchy rash?

- ☐ Never in the past 12 months
- ☐ Less than one night per week
- ☐ One or more nights per week

Q75 Has this rash cleared completely at any time during the <u>past 12 months</u>?

- ☐ No
- ☐ Yes

Q76 Do you think your teenager has <u>ever</u> had eczema?

- ☐ No
- ☐ Yes
- ☐ Don’t know

Q77 Has a doctor (GP, pediatrician) ever told you that your teenager has eczema?

- ☐ No
- ☐ Yes
- ☐ Don’t know

Q78 In the past 12 months, has your teenager suffered from eczema?

- [ ] No → **Go to Q82**
- [ ] Yes

Q79 In the past 12 months, how many episodes of eczema has your teenager had?

- [ ] 1 to 2
- [ ] 3 to 12
- [ ] More than 12

Q80 In which of the past 12 months did this problem occur?
*Please mark **all** months which apply*

[ ] January	[ ] July
[ ] February	[ ] August
[ ] March	[ ] September
[ ] April	[ ] October
[ ] May	[ ] November
[ ] June	[ ] December

Q81 Has your teenager taken/used any medication for eczema in the past 12 months?

- [ ] No → **Go to Q82**
- [ ] Yes

Please list the medication and indicate if it was prescription or non-prescription.

**Type of medication**	**Not Prescribed by doctor**	**Prescribed by doctor**
Moisturiser *(please specify):*	[ ]	[ ]
Steroid cream *(please specify):*	[ ]	[ ]
Oral steroids *(please specify):*	[ ]	[ ]
Tacrolimus ointment *(please specify):*	[ ]	[ ]
Other *non-prescription* medication *(please specify):*	[ ]	
Other *prescription* medication *(please specify):*		[ ]

OFFICE USE ONLY

Q83 R	1	2	3	4	5	6	7	8	9	10	11	12
Pe												
Wh												
Da												
Fr												
Eg												
Se												
Pr												
O												

Q82 Does your teenager have any regular contact with pets outside your home? (e.g. relatives, neighbours, friends)

☐ No → **Go to Q83**
☐ Yes

What types of animals?

Please mark **all** *applicable responses*	**Yes**
Cats	☐
Dogs	☐
Birds	☐
Fish	☐
Rodents (e.g. guinea pigs, rabbits, rats/mice)	☐
Reptiles/Amphibia	☐
Other (e.g. farm animals - sheep, horses cattle) *(please specify):* …………………………………………………………………………………………………..	☐

Q83 Has your teenager ever had any food allergies?

☐ No → **Go to Q84**
☐ Yes

Example Only

Food Type	***What starts it***		***What reaction(s)***	***Severity of the reaction***		
	Eating	***Contact***		***Mild***	***Moderate***	***Severe***
Peanut Products	☒	☒	***Difficulty breathing***	☐	☒	☐

Please mark **all** *appropriate responses and write in the spaces provided*

**Food Type**	**What starts it**		**What reaction(s)**	**Severity of the reaction**		
	Eating	***Contact***		***Mild***	***Moderate***	***Severe***
Peanut Products	☐	☐		☐	☐	☐
Wheat/Yeast	☐	☐		☐	☐	☐
Dairy	☐	☐		☐	☐	☐
Fruit	☐	☐		☐	☐	☐
Eggs	☐	☐		☐	☐	☐
Seafood	☐	☐		☐	☐	☐
Preservatives/Coloring	☐	☐		☐	☐	☐
Other *(please specify below):*	☐	☐		☐	☐	☐

The following questions (Q84-94) apply to your study teenager's family.

Q84 On average, over the past 6 months, about how many drinks of beer, wine, spirits or other alcoholic beverage have the people listed below taken?

Please mark **one** *response for each person (if applicable)*

	Don't drink alcohol	Less than 3 drinks a week	3 - 6 drinks a week	1 or 2 drinks a day	3 - 6 drinks a day	More than 6 drinks a day
Teen's Mother	☐	☐	☐	☐	☐	☐
Teen's Father	☐	☐	☐	☐	☐	☐
Your Partner/Defacto	☐	☐	☐	☐	☐	☐

Q85 In general how would you describe the health of the people listed below?

Please mark **one** *response for each person (if applicable)*

	Excellent	Very Good	Good	Fair	Poor
Teen's Mother	☐	☐	☐	☐	☐
Teen's Father	☐	☐	☐	☐	☐
Your Partner/Defacto	☐	☐	☐	☐	☐

Q86 Please answer ***both*** parts of the question below:

a. Do you have any medical conditions or health problems of a permanent or long term nature (that is, going to last for more than 6 months, e.g. diabetes, chronic back pain)?

b. Do these health problems or medical conditions limit you in any way in carrying out normal daily activities at home, at a job or in studying?

Please mark **one** *response for each person (if applicable)*

	a. Have health problems		b. Limited in daily activities		
	Yes	No	Yes	No	N/A (no health problems)
Teen's Mother	☐	☐	☐	☐	☐
Teen's Father	☐	☐	☐	☐	☐
Your Partner/Defacto	☐	☐	☐	☐	☐

Q87 This question asks about your family's history of asthma, allergic nose and eye reactions and eczema ***and*** whether or not it was diagnosed by a doctor:

Please mark **all** *applicable responses*

	No	Yes In the past	Yes Now	Yes Now and in the past	Diagnosed by a Doctor No	Diagnosed by a Doctor Yes
Has the teenagers' mother had…						
Asthma	☐	☐	☐	☐	☐	☐
Allergic nose reaction (inc. hayfever)	☐	☐	☐	☐	☐	☐
Allergic eye reaction	☐	☐	☐	☐	☐	☐
Eczema	☐	☐	☐	☐	☐	☐
Has the teenager's father had...						
Asthma	☐	☐	☐	☐	☐	☐
Allergic nose reaction (inc. hayfever)	☐	☐	☐	☐	☐	☐
Allergic eye reaction	☐	☐	☐	☐	☐	☐
Eczema	☐	☐	☐	☐	☐	☐
Have any of the teenager's brothers or sisters (siblings) had...						
Asthma	☐	☐	☐	☐	☐	☐
Allergic nose reaction (inc. hayfever)	☐	☐	☐	☐	☐	☐
Allergic eye reaction	☐	☐	☐	☐	☐	☐
Eczema	☐	☐	☐	☐	☐	☐

Q88 This question asks about your family's history of diabetes, kidney and heart conditions and arthritis ***and*** whether or not it was diagnosed by a doctor:

Please mark **all** *applicable responses*

	No	Yes In the past	Yes Now	Yes Now and in the past	Diagnosed by a Doctor No	Diagnosed by a Doctor Yes
Has the teenagers' mother had…						
Diabetes	☐	☐	☐	☐	☐	☐
Renal conditions (Kidney)	☐	☐	☐	☐	☐	☐
Arthritis	☐	☐	☐	☐	☐	☐
Cardiac conditions (Heart)	☐	☐	☐	☐	☐	☐
Has the teenager's father had...						
Diabetes	☐	☐	☐	☐	☐	☐
Renal conditions (Kidney)	☐	☐	☐	☐	☐	☐
Arthritis	☐	☐	☐	☐	☐	☐
Cardiac conditions (Heart)	☐	☐	☐	☐	☐	☐
Have any of the teenager's brothers or sisters (siblings) had...						
Diabetes	☐	☐	☐	☐	☐	☐
Renal conditions (Kidney)	☐	☐	☐	☐	☐	☐
Arthritis	☐	☐	☐	☐	☐	☐
Cardiac conditions (Heart)	☐	☐	☐	☐	☐	☐

Q89 This question asks about your family's history of depression, cholesterol problems and high blood pressure ***and*** whether or not it was diagnosed by a doctor.

Please mark **all** *applicable responses*

	No	Yes In the past	Yes Now	Yes Now and in the past	Diagnosed by a Doctor No	Diagnosed by a Doctor Yes
Has the teenagers' mother had…						
Depression needing treatment	☐	☐	☐	☐	☐	☐
Cholesterol problems	☐	☐	☐	☐	☐	☐
High blood pressure	☐	☐	☐	☐	☐	☐
Has the teenager's father had...						
Depression needing treatment	☐	☐	☐	☐	☐	☐
Cholesterol problems	☐	☐	☐	☐	☐	☐
High blood pressure	☐	☐	☐	☐	☐	☐
Have any of the teenager's brothers or sisters (siblings) had...						
Depression needing treatment	☐	☐	☐	☐	☐	☐
Cholesterol problems	☐	☐	☐	☐	☐	☐
High blood pressure	☐	☐	☐	☐	☐	☐

Q90 This question asks about your family's history of coeliac disease and hemochromatosis (iron overload disease) ***and*** whether or not it was diagnosed by a doctor (please include half-brothers and half-sisters but not step-brothers or step-sisters):

Please mark ***all*** *applicable responses*

	No	Yes In the past	Yes Now	Yes Now and in the past	Diagnosed by a Doctor No	Diagnosed by a Doctor Yes
Has the teenagers' mother had…						
Coeliac disease	☐	☐	☐	☐	☐	☐
Hemochromatosis (iron overload)	☐	☐	☐	☐	☐	☐
Has the teenager's father had...						
Coeliac disease	☐	☐	☐	☐	☐	☐
Hemochromatosis (iron overload)	☐	☐	☐	☐	☐	☐
Have any of the teenager's brothers or sisters (siblings) had...						
Coeliac disease	☐	☐	☐	☐	☐	☐
Hemochromatosis (iron overload)	☐	☐	☐	☐	☐	☐

Q91 Has the study teenager's mother ever had post-natal depression?

☐ No → **Go to Q92**
☐ Yes

When did you/she have post-natal depression?

Please mark **all** *responses that apply*	**No**	**Yes**	**Don't know/unsure**	**N/A**
With child(ren) born before the study teenager	☐	☐	☐	☐
With child(ren) born after the study teenager	☐	☐	☐	☐
Associated with the birth of the study teenager	☐	☐	☐	

Q92 Have any of the people listed below ever been treated for an emotional or mental health problem (other than post-natal depression)?

Please mark **one** *response for each person (if applicable)*

	**No**	**Yes**
Teen's Mother	☐	☐
Teen's Father	☐	☐
Your Partner/Defacto	☐	☐

Q93 Have any of the people listed below been treated for an emotional or mental health problem within the past 6 months?

Please mark **one** *response for each person (if applicable)*

	**Yes**	**No**	**N/A (Never had a treated emotional problem)**
Teen's Mother	☐	☐	☐
Teen's Father	☐	☐	☐
Your Partner/Defacto	☐	☐	☐

Q94 Have any of the people listed below ever been hospitalised for an emotional or mental health problem?

Please mark **one** *response for each person (if applicable)*

	**Yes**	**No**	**N/A (Never had a treated emotional problem)**
Teen's Mother	☐	☐	☐
Teen's Father	☐	☐	☐
Your Partner/Defacto	☐	☐	☐

CHILD BEHAVIOUR CHECKLIST (FOR AGES 4-18 YEARS)

Q95 This question asks for information on the health, behaviour and social activities of your teenager to help us to describe different patterns of behaviour and to understand how these affect the health, education and wellbeing of children.

Please answer all items as well as you can, even if some do not seem to apply to your teenager.

For each item that describes your teenager now, or within the past 6 months, please mark only one response as:

0=Not True (as far as you know) 1=Somewhat or Sometimes True 2=Very true or Often true

	0	1	2		0	1	2
1. Acts too young for his/her age	☐	☐	☐	28. Eats or drinks things that are not food (don't include sweets (describe):……………………… ………………………………………………………	☐	☐	☐
2. Allergy (describe):…………………………….. …………………………………………………..	☐	☐	☐	29. Fears certain animals, situations or places, other than school (describe): …………………… …………………………………………………..	☐	☐	☐
3. Argues a lot	☐	☐	☐	30. Fears going to school	☐	☐	☐
4. Asthma	☐	☐	☐	31. Fears he/she might think or do something bad	☐	☐	☐
5. Behave like opposite sex	☐	☐	☐	32. Feels he/she has to be perfect	☐	☐	☐
6. Bowel movements outside toilet	☐	☐	☐	33. Feels or complains that no one loves him/her	☐	☐	☐
7. Bragging, boasting	☐	☐	☐	34. Feels others are out to get him/her	☐	☐	☐
8. Can't concentrate, can't pay attention for long	☐	☐	☐	35. Feels worthless or inferior	☐	☐	☐
9. Can't get his/her mind off certain thoughts; obsessions (describe):……………………….. …………………………………………………..	☐	☐	☐	36. Gets hurt a lot or accident-prone	☐	☐	☐
10. Can't sit still, restless, hyperactive	☐	☐	☐	37. Gets in many fights	☐	☐	☐
11. Clings to adults or too dependent	☐	☐	☐	38. Gets teased a lot	☐	☐	☐
12. Complains of loneliness	☐	☐	☐	39. Hangs around with others who get in trouble	☐	☐	☐
13. Confused or seems to be in a fog	☐	☐	☐	40. Hears sounds or voices that aren't there (describe):………………………………………….	☐	☐	☐
14. Cries a lot	☐	☐	☐	41. Impulsive or acts without thinking	☐	☐	☐
15. Cruel to animals	☐	☐	☐	42. Would rather be alone than with others	☐	☐	☐
16. Cruelty, bullying or meanness to others	☐	☐	☐	43. Lying or cheating	☐	☐	☐
17. Day-dreams or gets lost in his/her thoughts	☐	☐	☐	44. Bites fingernails	☐	☐	☐
18. Deliberately harms self or attempts suicide	☐	☐	☐	45. Nervous, high strung or tense	☐	☐	☐
19. Demands a lot of attention	☐	☐	☐	46. Nervous movements or twitching (describe): ………………………………………………………	☐	☐	☐
20. Destroys his/her own things	☐	☐	☐	47. Nightmares	☐	☐	☐
21. Destroys things belonging to his/her family or others	☐	☐	☐	48. Not liked by other kids	☐	☐	☐
22. Disobedient at home	☐	☐	☐	49. Constipated, doesn't move bowels	☐	☐	☐
23. Disobedient at school	☐	☐	☐	50. Too fearful or anxious	☐	☐	☐
24. Doesn't eat well	☐	☐	☐	51. Feels dizzy	☐	☐	☐
25. Doesn't get along with other kids	☐	☐	☐	52. Feels too guilty	☐	☐	☐
26. Doesn't seem to feel guilty after misbehaving	☐	☐	☐	53. Overeating	☐	☐	☐
27. Easily jealous	☐	☐	☐	54. Overtired	☐	☐	☐

0=Not True (as far as you know) **1=Somewhat or Sometimes True** **2=Very true or Often true**

	0	1	2
55. Overweight	☐	☐	☐
56. <u>Physical problems without medical cause</u>:			
a. Aches or pains (not headaches)	☐	☐	☐
b. Headaches	☐	☐	☐
c. Nausea, feels sick	☐	☐	☐
d. Problems with eyes (describe):	☐	☐	☐
e. Rashes or other skin problems	☐	☐	☐
f. Stomach aches or cramps	☐	☐	☐
g. Vomiting, throwing up	☐	☐	☐
h. Other (describe):	☐	☐	☐
57. Physically attacks people	☐	☐	☐
58. Picks nose, skin or other parts of body (describe):………………………………………	☐	☐	☐
59. Plays with own sex parts in public	☐	☐	☐
60. Plays with own sex parts too much	☐	☐	☐
61. Poor school work	☐	☐	☐
62. Poorly coordinated or clumsy	☐	☐	☐
63. Prefers being with older kids	☐	☐	☐
64. Prefers being with younger kids	☐	☐	☐
65. Refuses to talk	☐	☐	☐
66. Repeats certain acts over and over; compulsions (describe):………………………………………	☐	☐	☐
67. Runs away from home	☐	☐	☐
68. Screams a lot	☐	☐	☐
69. Secretive, keeps things to self	☐	☐	☐
70. Sees things that aren't there (describe):……………………………………… ………………………………………………………	☐	☐	☐
71. Self-conscious or easily embarrassed	☐	☐	☐
72. Sets fires	☐	☐	☐
73. Sexual problems (describe):……………………………………… ………………………………………………………	☐	☐	☐
74. Showing off or clowning	☐	☐	☐
75. Shy or timid	☐	☐	☐
76. Sleeps less than most kids	☐	☐	☐
77. Sleeps more than most kids during the day and/or at night (describe):	☐	☐	☐
78. Smears or plays with bowel movements	☐	☐	☐
79. Speech problem (describe):……………………………………… ………………………………………………………	☐	☐	☐
80. Stares blankly	☐	☐	☐

	0	1	2
81. Steals at home	☐	☐	☐
82. Steals outside the home	☐	☐	☐
83. Stores up things he/she doesn't need (describe):………………………………………	☐	☐	☐
84. Strange behaviour (describe):………………………………………	☐	☐	☐
85. Strange ideas (describe):………………………………………	☐	☐	☐
86. Stubborn, sullen or irritable	☐	☐	☐
87. Sudden changes in mood or feelings	☐	☐	☐
88. Sulks a lot	☐	☐	☐
89. Suspicious	☐	☐	☐
90. Swearing or obscene language	☐	☐	☐
91. Talks about killing self	☐	☐	☐
92. Talks or walks in sleep (describe):	☐	☐	☐
93. Talks too much	☐	☐	☐
94. Teases a lot	☐	☐	☐
95. Temper tantrums or hot temper	☐	☐	☐
96. Thinks about sex too much	☐	☐	☐
97. Threatens people	☐	☐	☐
98. Thumb sucking	☐	☐	☐
99. Too concerned about neatness or cleanliness	☐	☐	☐
100. Trouble sleeping (describe):	☐	☐	☐
101. Truancy, skips school	☐	☐	☐
102. Under active, slow moving or lacks energy	☐	☐	☐
103. Unhappy, sad or depressed	☐	☐	☐
104. Unusually loud	☐	☐	☐
105. Uses alcohol or drugs for non-medical purposes (describe):………….. ………………………………………………………	☐	☐	☐
106. Vandalism	☐	☐	☐
107. Wets self during the day	☐	☐	☐
108. Wets the bed	☐	☐	☐
109. Whining	☐	☐	☐
110. Wishes to be of opposite sex	☐	☐	☐
111. Withdrawn, doesn't get involved with others	☐	☐	☐
112. Worries	☐	☐	☐
113. Please write in any problems your teenager has that were not listed above: ……………………………………………………… ……………………………………………………… ………………………………………………………	☐	☐	☐

Q96 Are you worried about your teenager's weight?

☐ Not at all
☐ A little
☐ Moderately
☐ Very

Q97 Do you consider your teenager to be…

☐ Underweight?
☐ Normal weight?
☐ A bit overweight?
☐ Very overweight?

Q98 Do you have any concerns or worries about your teenager's health or development (e.g. speech/language development, physical development, emotional development)?

☐ No → **Go to Q99**
☐ Yes

Please tell us about these concerns if you wish to do so in the space provided:

Q99 Date questionnaire completed: ☐☐ / ☐☐ / ☐☐☐☐

Q100 Please write below any comments concerning this questionnaire, the research or anything else you would like to tell us about:

THANK YOU

WE APPRECIATE THE TIME THAT YOU HAVE SPENT COMPLETING THIS QUESTIONNAIRE

ID ☐☐☐☐

OFFICE USE ONLY

RA-CH RA-CO RA1-E RA2-E

ID

GROWING UP HEALTHY STUDY

Participant Questionnaire

16-18

The purpose of this questionnaire is to obtain information about a range of topics including your household and neighbourhood, your study, job seeking and work experiences, and your health and wellbeing.

Please read each question carefully and answer all of the questions.
Write your answers clearly in the space provided or mark the most appropriate response

☒

All information will be strictly confidential

Please take your time in answering all of the questions

If you require assistance to answer any of the questions please contact the Study Coordinators:

Blagica & Tina
T: +61 6458 1443
M: 0439 266 434
Email: ***guhstudy-swih@uwa.edu.au***

Please complete this questionnaire independently
(without discussing it with anyone)

If you are coming in for an appointment, please bring your completed questionnaire with you on the day.

If you are unable to attend an appointment, please use the Reply Paid envelope enclosed to return your completed questionnaire.

If possible, could you please return your completed questionnaire to us by:

☐☐ / ☐☐ / ☐☐☐☐

Questionnaire

What is your date of birth?

☐☐ / ☐☐ / ☐☐☐☐

What is your gender?

☐ M ☐ F

SECTION 1 Housing and Family

Q1 What is your current residential postcode? ☐☐☐☐

Q2 What type of dwelling do you live in?
Please mark only **one** *response*

☐ Separate house
☐ Semi-detached house/row or terrace house/townhouse etc.
☐ Flat/unit/apartment
☐ Boarding school
☐ Boarding house, hostel
☐ Caravan/tent/cabin/houseboat
☐ Other private dwelling *(please specify):*…………………………………………

Q3 How many adults and children live in your home?
Please include yourself. Children less than one year of age: Age = 0. If there are more than 10 people living in your home please write their information on the very last page of this questionnaire.

First name	Age (years)	Sex (M/F)	Relationship to you
e.g. Karyn<br>Cameron<br>Diane<br>Brian	16<br>18<br>45<br>47	F<br>M<br>F<br>M	Me<br>Step brother<br>Mother<br>Step father
1			
2			
3			
4			
5			
6			
7			
8			
9			
10			

SECTION 2 Study, Work and Income

SCHOOL

Q4 Have you ever repeated a year/grade at school?

- ☐ No
- ☐ Yes *Which year(s)/grade(s)?*...

Q5 When you leave school/now that you have left school - what do you intend to do?
Please mark only **one** *response - the main one unless two or more responses apply equally*

- ☐ Study at TAFE
- ☐ Study at University
- ☐ Other study *(please specify)*:………………………………………….…..
- ☐ Work
- ☐ Take time off – travel etc.
- ☐ Care for own child(ren) or other family member
- ☐ Other *(please specify)*:…………………………………………………...

Q6 Are you currently enrolled as a student at a secondary school or in a secondary school level program of study?

- ☐ No → **Go to Q15**
- ☐ Yes

Q7 What is the name and suburb of the school you are currently attending?

School: …………………………………………………… Suburb: …………..…………………..

Q8 What year/grade are you in secondary school now? ☐☐ year/grade

Q9 Which of these categories best describes the school you currently attend?

- ☐ Government school
- ☐ Catholic non-government school
- ☐ Other non-government school
- ☐ Don't know
- ☐ Other, not included above *(please specify)*:……………………………………………

Q10 In general, how do you feel about school?

**Very satisfied**	**Quite satisfied**	**Neither satisfied nor dissatisfied**	**Somewhat dissatisfied**	**Vey dissatisfied**
☐	☐	☐	☐	☐

Q11 How would you describe your academic performance in school during the past 6 months?

**Poor**	**Below average**	**Average**	**Very good**	**Excellent**
☐	☐	☐	☐	☐

OFFICE USE ONLY

Q7 ☐☐☐☐

Q12 How much do these things go on among people from your school, during or outside of school hours?

*Please mark **one** response for each item*	Not at all	A Little	Some	Quite a bit	Very much
Students using drugs before and after school	☐	☐	☐	☐	☐
Students destroying things (vandalism)	☐	☐	☐	☐	☐
Students drinking beer/wine/spirits	☐	☐	☐	☐	☐
Students getting into fights	☐	☐	☐	☐	☐
Students stealing things	☐	☐	☐	☐	☐
Students threatening or bullying	☐	☐	☐	☐	☐

Q13 How well are these goals being met in your life?

For each goal please select the number between 1 and 7 that indicates how well these goals are being met for you. The better a goal is met the higher the number you should select. The less a goal is met the lower the number.

*Please mark **one** response for each item*	Very poorly<br>1	2	3	Average<br>4	5	6	Very well<br>7
Having a high grade average	☐	☐	☐	☐	☐	☐	☐
Attending classes regularly	☐	☐	☐	☐	☐	☐	☐
Doing well even in hard subjects	☐	☐	☐	☐	☐	☐	☐
Having others think of you as a good student	☐	☐	☐	☐	☐	☐	☐
Deciding on a future career/education	☐	☐	☐	☐	☐	☐	☐

Q14 For each of these statements, which is most true for you?

*Please mark **one** response for each item*	Strongly disagree	Disagree	Agree	Strongly agree
I enjoy the work I do at school	☐	☐	☐	☐
I have lots of friends at my school	☐	☐	☐	☐
I am motivated to want to learn at my school	☐	☐	☐	☐
At school I learn things that will be useful to me when I leave	☐	☐	☐	☐
Learning is fun at my school	☐	☐	☐	☐
People at my school think a lot of me	☐	☐	☐	☐
I am keen to do well at school	☐	☐	☐	☐
What I learn at school will help me get a job when I leave	☐	☐	☐	☐

Q14 *Continued…*

Please mark **one** *response for each item*	**Strongly disagree**	**Disagree**	**Agree**	**Strongly agree**
I get excited about the work I do at school	☐	☐	☐	☐
I get on well with others at school	☐	☐	☐	☐
I think it is worth trying hard at my school work	☐	☐	☐	☐
What I learn at school is useful to me	☐	☐	☐	☐
I enjoy being at my school	☐	☐	☐	☐
I am popular with others at my school	☐	☐	☐	☐
I want to get good results	☐	☐	☐	☐
What I learn at school will be useful to me in the future	☐	☐	☐	☐
**→ Go to Q22**				

STUDY

Q15 In which month and year did you leave school?

Month ☐☐ Year ☐☐☐☐

Q16 What was the highest year of school you have completed?

- ☐ Year 12 (or equivalent)
- ☐ Year 11 (or equivalent)
- ☐ Year 10 (or equivalent)
- ☐ Other *(please specify)*:……………………………………………………

Q17 Which of these categories best describes the school you attended in your last year?

- ☐ Government school
- ☐ Catholic non-government school
- ☐ Other non-government school
- ☐ Don't know
- ☐ Other, not included above *(please specify)*:……………………………………………

Q18 Have you spent any time enrolled in any other course of study for a trade certificate, diploma, degree or any other educational qualification?

- ☐ Certificate level 1
- ☐ Certificate level 2
- ☐ Certificate level 3
- ☐ Certificate level 4
- ☐ Certificate level 5
- ☐ Diploma (2 years full-time or equivalent)
- ☐ Associate Degree
- ☐ Advanced Diploma (3 years full-time or equivalent)
- ☐ Bachelor Degree but not honours
- ☐ Honours Bachelor Degree
- ☐ Graduate Certificate
- ☐ Graduate Diploma
- ☐ Masters Degree
- ☐ Doctorate
- ☐ Other *(please specify)*:…………...……………

If you have been enrolled in any other educational qualification (not listed above), please complete the details below:

Title of course:	
Description:	
Length of course (full-time):	

Q19 Are you still studying?

☐ No → **Go to Q22**
☐ Yes

Q20 Are you studying this full-time or part-time?

☐ Full-time student ☐ Part-time student

Q21 For each of these statements, which is most true for you?

Please mark **one** *response for each item*	**Strongly disagree**	**Disagree**	**Agree**	**Strongly agree**
I enjoy the work I do at TAFE/ Uni	☐	☐	☐	☐
I have lots of friends at my TAFE/ Uni	☐	☐	☐	☐
I am motivated to want to learn at my TAFE/ Uni	☐	☐	☐	☐
At school I learn things that will be useful to me when I finish	☐	☐	☐	☐
Learning is fun at my TAFE/ Uni	☐	☐	☐	☐
People at my TAFE/ Uni think a lot of me	☐	☐	☐	☐
I am keen to do well at TAFE/ Uni	☐	☐	☐	☐
What I learn at TAFE/ Uni will help me get a job when I finish	☐	☐	☐	☐
I get excited about the work I do at TAFE/ Uni	☐	☐	☐	☐
I get on well with others at TAFE/ Uni	☐	☐	☐	☐
I think it is worth trying hard at my TAFE/ Uni	☐	☐	☐	☐
What I learn at TAFE/ Uni is useful to me	☐	☐	☐	☐
I enjoy being at my TAFE/ Uni	☐	☐	☐	☐
I am popular with others at my TAFE/ Uni	☐	☐	☐	☐
I want to get good results at TAFE/ Uni	☐	☐	☐	☐
What I learn at TAFE/ Uni will be useful to me in the future	☐	☐	☐	☐

WORK

Q22 Do you currently have a full-time or part-time job of any kind (excluding home duties)?
Please mark only **one** *response- the main job*

- ☐ No, do not have a job - not seeking work → **Go to Q26**
- ☐ No, do not have a job - actively seeking work → **Go to Q26**
- ☐ Yes, do work for payment or profit
- ☐ Yes, do unpaid work in a family business
- ☐ Yes, do other unpaid work

Q23 In your main job (if you have more than one job, then 'main job' refers to the job in which you usually work the most hours) are you...
Please mark only ***one*** *response*

- ☐ A salary or wage earner?
- ☐ A helper not receiving wages?
- ☐ Conducting your own business - with employees?
- ☐ Conducting your own business - without employees?

Q24 Describe your current main job.
Please give details of job and description of work in detail

Job title:
Job Description:

Q25 How many hours do you usually work in all jobs?

- ☐ None or less than one hour
- ☐ One or more hours per week *(please specify):*……………………………………

INCOME

Q26 Are you receiving any government benefits, pension or allowance?

- ☐ No → **Go to Section 3**
- ☐ Yes

Q27 Which government benefits, pension or allowance are you receiving?
Please mark **all** *responses that apply*

- ☐ Austudy/Abstudy
- ☐ Carer payment - caring for child/relative
- ☐ Disability support pension - self
- ☐ Parenting payment
- ☐ Remote area allowance
- ☐ Rent assistance
- ☐ Sickness benefit
- ☐ Tax Benefit Part A
- ☐ Tax Benefit Part B
- ☐ Workers compensation
- ☐ Youth allowance
- ☐ Other *(please specify):*………………

OFFICE USE ONLY

Q24 ☐☐☐☐☐☐

SECTION 3 Housing Environment

Q28 Is your home air-conditioned?

- ☐ No
- ☐ Yes

Q29 Is your home heated?

- ☐ No
- ☐ Yes

Q30 Do you have gas cooking in your home?

- ☐ No
- ☐ Yes

Q31 Are there any pets at home?

- ☐ No → **Go to Q33**
- ☐ Yes

Q32 How many pets are there? *If you have no pets at home then please leave this section blank. If you have either no cats, no dogs or birds or no other pets, then print the number 0 in the spaces.*

Please print the number in the space provided	**Inside**	**Outside**	**Total**
Cats	☐☐	☐☐	☐☐
Dogs	☐☐	☐☐	☐☐
Birds (include ducks, geese, chickens)	☐☐	☐☐	☐☐

Other pets?

How many other pets inside? ☐☐ What type? ………………………………………………….

How many other pets outside? ☐☐ What type? ………………………………………………….

Q33 Do you have any regular contact with pets elsewhere (e.g. relatives, neighbours, friends)?

- ☐ No → **Go to Section 4**
- ☐ Yes

Q34 What types of animals?

Please mark ***all*** *applicable responses*	**Yes**
Cats	☐
Dogs	☐
Birds	☐
Fish	☐
Rodents (e.g. guinea pigs, rabbits, rats/mice)	☐
Reptiles/Amphibia	☐
Other (e.g. farm animals - sheep, horses cattle) *(please specify):* ………………………………………………………………………………………...	☐

SECTION 4 Your Health and Wellbeing - Strictly Confidential

The following questions ask about your health and wellbeing. This information will help keep track of how you feel and how well you are able to do your usual activities.
If you are unsure about how to answer a question please give the best answer you can.

Q35 In general, how would you describe your health?
Please mark only **one** *response*

☐ Excellent
☐ Very good
☐ Good
☐ Fair
☐ Poor

The following questions are about activities you might do during a typical day:

Q36 Does your health now limit you in these activities?

Please mark **one** *response for each item*	**Yes limited a lot**	**Yes limited a little**	**No not limited at all**
Vigorous activities such as running, lifting heavy objects, participating in strenuous sports	☐	☐	☐
Moderate activities such as moving a table, pushing a vacuum cleaner, bowling or playing golf	☐	☐	☐
Lifting or carrying groceries	☐	☐	☐
Climbing several flights of stairs	☐	☐	☐
Climbing one flight of stairs	☐	☐	☐
Bending, kneeling or stooping	☐	☐	☐
Walking more than one kilometre	☐	☐	☐
Walking several blocks	☐	☐	☐
Walking one block	☐	☐	☐
Bathing or dressing yourself	☐	☐	☐

Q37 During the past 4 weeks, have you had any problems with your physical health?

☐ No → **Go to Q41**
☐ Yes

↓

Q38 Have you had any of the following problems with your work, school work, or regular daily activities, as a result of these physical health problems?

Please mark **one** *response for each item*	**Yes**	**No**
Cut down on the amount of time you spent on work or other activities	☐	☐
Accomplished less than you would like	☐	☐
Were limited in the kind of work or other activities	☐	☐
Had difficulty performing the work or other activities (i.e. it took extra effort)	☐	☐

Q39 During the past 4 weeks, to what extent have these physical health problems interfered with your normal social activities with family, friends, neighbours or groups?

- ☐ Not at all → **Go to Q41**
- ☐ Slightly
- ☐ Moderately
- ☐ Quite a bit
- ☐ Extremely

Q40 During the past 4 weeks, how much of the time have these physical health problems interfered with your normal social activities (like visiting friends, relatives etc.)?

- ☐ All of the time
- ☐ Most of the time
- ☐ Some of the time
- ☐ A little of the time

Q41 During the past 4 weeks, have you had any problems with your emotional health?

- ☐ No → **Go to Q45**
- ☐ Yes

Q42 Have you had any of the following problems with your work, school work, or regular daily activities, as a result of these emotional health problems?

Please mark **one** *response for each item*	**Yes**	**No**
Cut down on the amount of time you spent on work or other activities	☐	☐
Accomplished less than you would like	☐	☐
Didn't do work or other activities as carefully as usual	☐	☐

Q43 During the past 4 weeks, to what extent have these emotional health problems interfered with your normal social activities with family, friends, neighbours or groups?

- ☐ Not at all → **Go to Q45**
- ☐ Slightly
- ☐ Moderately
- ☐ Quite a bit
- ☐ Extremely

Q44 During the past 4 weeks, how much of the time have these emotional health problems interfered with your normal social activities (like visiting friends, relatives etc.)?

- ☐ All of the time
- ☐ Most of the time
- ☐ Some of the time
- ☐ A little of the time

Q45 These questions are about how you feel and how things have been for you during the past 4 weeks. Please give the one answer that is closest to the way you have been feeling for each item:

*Please mark **one** response for each item*	**All of the time**	**Most of the time**	**A good bit of the time**	**Some of the time**	**A little of the time**	**None of the time**
Did you feel full of life?	☐	☐	☐	☐	☐	☐
Have you been a very nervous person?	☐	☐	☐	☐	☐	☐
Have you felt so down in the dumps that nothing could cheer you up?	☐	☐	☐	☐	☐	☐
Have you felt calm and peaceful?	☐	☐	☐	☐	☐	☐
Did you have a lot of energy?	☐	☐	☐	☐	☐	☐
Have you felt downhearted and blue?	☐	☐	☐	☐	☐	☐
Did you feel worn out?	☐	☐	☐	☐	☐	☐
Have you been a happy person?	☐	☐	☐	☐	☐	☐
Did you feel tired?	☐	☐	☐	☐	☐	☐

Q46 During the past 4 weeks, have you had any physical pain?

☐ No → **Go to Q49**
☐ Yes

Q47 During the past 4 weeks, how bad/severe was that physical pain?

☐ Very mild
☐ Mild
☐ Moderate
☐ Severe
☐ Very severe

Q48 During the past 4 weeks, how much did this physical pain interfere with your normal work (including both work outside the home, schoolwork and housework)?

☐ Not at all
☐ Slightly
☐ Moderately
☐ Quite a bit

Q49 How true or false are each of the following statements for you?

*Please mark **one** response for each item*	**Definitely true**	**Mostly true**	**Don't know**	**Mostly False**	**Definitely false**
I seem to get sick a little easier than other people	☐	☐	☐	☐	☐
I am as healthy as anybody I know	☐	☐	☐	☐	☐
I expect my health to get worse	☐	☐	☐	☐	☐
My health is excellent	☐	☐	☐	☐	☐

WHEEZE

Q50 Do you usually cough when you gets a cold these days?

☐ No
☐ Yes
☐ Don't know

Q51 Do you seem congested or bring up phlegm (spit) from his/her chest with colds?

☐ No
☐ Yes
☐ Don't know

Q52 Have you wheezed at any time in your life? (wheeze is a whistling or rattling noise in the chest, best heard when breathing out)

☐ No → **Go to Q58**
☐ Yes

↓

Q53 Have you wheezed in the <u>past 12 months</u>?

☐ No → **Go to Q58**
☐ Yes

↓

Q54 How many episodes of wheezing have you had in the past <u>12 months</u>?

☐ 1 to 2
☐ 3 to 12
☐ More than 12

In the past 12 months…

Q55 How often, on average has your sleep been disturbed due to wheezing?

☐ Never woken with wheezing
☐ Less than one night per week
☐ One or more nights per week
☐ Don't know

Q56 Has wheezing ever been severe enough to limit your speech to only one or two words at a time between breaths?

☐ No
☐ Yes
☐ Don't know

Q57 Has your chest sounded wheezy during or after exercise?

☐ No
☐ Yes
☐ Don't know

ASTHMA

Q58 Do you think you have ever had asthma?

- ☐ No
- ☐ Yes
- ☐ Don't know

Q59 Has a doctor (GP, pediatrician, respiratory specialist) ever told you that you have asthma?

- ☐ No
- ☐ Yes
- ☐ Don't know

Q60 Do you still have asthma?

- ☐ Not applicable (never had asthma)
- ☐ No
- ☐ Yes
- ☐ Don't know

Q61 Has you taken/used any of the following asthma medications in the past 12 months?

- ☐ No → **Go to Q62**
- ☐ Yes

Please mark **all** *appropriate answers*	**Yes**	**Ordered by a Doctor**	
		**Yes**	**No**
Ventolin (Asmol, Airomir, etc.)	☐	☐	☐
Respolin	☐	☐	☐
Nuelin	☐	☐	☐
Theo-dur	☐	☐	☐
Bricanyl	☐	☐	☐
Atrovent	☐	☐	☐
QVAR	☐	☐	☐
Flixotide	☐	☐	☐
Pulmacort	☐	☐	☐
Berotec	☐	☐	☐
OXIS	☐	☐	☐
Serevent	☐	☐	☐
Singlulaire	☐	☐	☐
Accolate	☐	☐	☐
Seretide	☐	☐	☐
Symbacort	☐	☐	☐
Prednisolone	☐	☐	☐
Other *(please specify):* …………………………………………………………	☐	☐	☐

The following questions are about problems which occurred when you DID NOT have a cold or flu:

RHINITIS (runny or blocked nose - including hayfever)

Q62 Have you ever had a problem with sneezing or a runny or blocked nose (including hayfever) when you DID NOT have a cold or the flu?

☐ No → **Go to Q70**
☐ Yes

↓

Q63 In the past 12 months, have you had a problem with sneezing or a runny or blocked nose (including hayfever) when you DID NOT have a cold or flu?

☐ No → **Go to Q67**
☐ Yes

↓

Q64 In the past 12 months, was this nose problem accompanied by itchy-watery eyes?

☐ No
☐ Yes

Q65 In the past 12 months, how many episodes of allergic nose problem have you had (including hayfever)?

☐ 1 to 2
☐ 3 to 12
☐ More than 12

Q66 In which of the past 12 months did this problem occur?
Please mark **all** *months which apply*

☐ January
☐ February
☐ March
☐ April
☐ May
☐ June
☐ July
☐ August
☐ September
☐ October
☐ November
☐ December

Q67 Has a doctor (GP, paediatrician) ever told you that you have an allergic nose problem (including hayfever)?

☐ No
☐ Yes

Q68 What was the trigger/cause of these episodes?
*Please mark **all** responses that apply*

- ☐ Grass
- ☐ Pollen
- ☐ Animal
- ☐ Dust
- ☐ Other *(please specify):*…………………………………………………………………
- ☐ Don't know

Q69 Have you taken/used any medication for an allergic nose problem (including hayfever) in the past 12 months?

- ☐ No → **Go to Q70**
- ☐ Yes

Please list the medication and indicate if it was prescription or non-prescription.

**Type of medication**	**Not Prescribed by doctor**	**Prescribed by doctor**
Steroid nasal spray *(please specify):*	☐	☐
Non-steroid nasal spray *(please specify):*	☐	☐
Antihistamine drops/tablets *(please specify):*	☐	☐
Other *non-prescription* medication *(please specify):*	☐	
Other *prescription* medication *(please specify):*		☐

ALLERGIC CONJUNCTIVITIS (itchy water eyes - including hayfever)

Q70 Have you ever had a problem with red/watery or itchy eyes?

- ☐ No → **Go to Q78**
- ☐ Yes

Q71 Do you think you have ever had allergic reaction in the eyes (including hayfever)?

- ☐ No
- ☐ Yes
- ☐ Don’t know

Q72 Has a doctor (GP, pediatrician) ever told you that you had an allergic reaction in the eyes (including hayfever)?

- ☐ No
- ☐ Yes
- ☐ Don’t know

Q73 In the past 12 months, have you suffered from an allergic reaction in the eyes (including hayfever)?

- ☐ No → **Go to Q78**
- ☐ Yes

Q74 In the past 12 months, how many episodes of allergic reaction in the eyes have you had (including hayfever)?

- ☐ 1 to 2
- ☐ 3 to 12
- ☐ More than 12

Q75 In which of the past 12 months did this problem occur?
Please mark **all** *months which apply*

- ☐ January
- ☐ February
- ☐ March
- ☐ April
- ☐ May
- ☐ June
- ☐ July
- ☐ August
- ☐ September
- ☐ October
- ☐ November
- ☐ December

Q76 What was the trigger/cause of these episodes?
Please mark **all** *responses that apply*

- ☐ Grass
- ☐ Pollen
- ☐ Animal
- ☐ Dust
- ☐ Other *(please specify):*…………………………………………………
- ☐ Don't know

Q77 Have you taken/used any medication for an allergic eye reaction (including hayfever) in the past 12 months?

- ☐ No → **Go to Q78**
- ☐ Yes

Please list the medication and indicate if it was prescription or non-prescription.

**Type of medication**	**Not Prescribed by doctor**	**Prescribed by doctor**
Eye drops *(please specify):*	☐	☐
Steroid tablets *(please specify):*	☐	☐
Antihistamine drops/tablets *(please specify):*	☐	☐
Other *non-prescription* medication *(please specify):*	☐	
Other *prescription* medication *(please specify):*		☐

ECZEMA (itchy rash)

Q78 Have you ever had eczema or an itchy rash which was coming and going for at least 6 months?

☐ No → **Go to Q89**
☐ Yes

Q79 Has this eczema / itchy rash at any time affected any one of the following places; the folds of the elbows, behind the knees, in front of the ankles, under the buttocks, or around the neck, ears, or eyes?

☐ No
☐ Yes

Q80 Have you had this eczema/itchy rash in the past 12 months?

☐ No → **Go to Q83**
☐ Yes

Q81 In the past 12 months, how often, on average, have you been kept awake at night by this itchy rash?

☐ Never in the past 12 months
☐ Less than one night per week
☐ One or more nights per week

Q82 Has this rash cleared completely at any time during the past 12 months?

☐ No
☐ Yes

Q83 Do you think that you have ever had eczema?

☐ No
☐ Yes
☐ Don't know

Q84 Has a doctor (GP, paediatrician) ever told you that you have eczema?

☐ No
☐ Yes
☐ Don't know

Q85 In the past 12 months, have you suffered from eczema?

- ☐ No → **Go to Q89**
- ☐ Yes

Q86 In the past 12 months, how many episodes of eczema have you had?

- ☐ 1 to 2
- ☐ 3 to 12
- ☐ More than 12

Q87 In which of the past 12 months did this problem occur?
Please mark **all** *months which apply*

☐ January	☐ July
☐ February	☐ August
☐ March	☐ September
☐ April	☐ October
☐ May	☐ November
☐ June	☐ December

Q88 Have you taken/used any medication for eczema in the past 12 months?

- ☐ No → **Go to Q89**
- ☐ Yes

Please list the medication and indicate if it was prescription or non-prescription.

**Type of medication**	**Not Prescribed by doctor**	**Prescribed by doctor**
Moisturiser *(please specify):*	☐	☐
Steroid cream *(please specify):*	☐	☐
Oral steroids *(please specify):*	☐	☐
Tacrolimus ointment *(please specify):*	☐	☐
Other *non-prescription* medication *(please specify):*	☐	
Other *prescription* medication *(please specify):*		☐

Q89 Do you have any food allergies?

☐ No → **Go to Q90**
☐ Yes

Example Only

Food Type	***What starts it***		***What reaction(s)***	***Severity of the reaction***		
	Eating	***Contact***		***Mild***	***Moderate***	***Severe***
Peanut Products	☒	☒	***Difficulty breathing***	☐	☒	☐

Please mark **all** *appropriate responses and write in the spaces provided*

**Food Type**	**What starts it**		**What reaction(s)**	**Severity of the reaction**		
	Eating	***Contact***		***Mild***	***Moderate***	***Severe***
Peanut Products	☐	☐		☐	☐	☐
Wheat/Yeast	☐	☐		☐	☐	☐
Dairy	☐	☐		☐	☐	☐
Fruit	☐	☐		☐	☐	☐
Eggs	☐	☐		☐	☐	☐
Seafood	☐	☐		☐	☐	☐
Preservatives/Coloring	☐	☐		☐	☐	☐
Other *(please specify below):*	☐	☐		☐	☐	☐

OFFICE USE ONLY

Q89	R	1	2	3	4	5	6	7	8	9	10	11	12
Pe		☐	☐	☐	☐	☐	☐	☐	☐	☐	☐	☐	☐
Wh		☐	☐	☐	☐	☐	☐	☐	☐	☐	☐	☐	☐
Da		☐	☐	☐	☐	☐	☐	☐	☐	☐	☐	☐	☐
Fr		☐	☐	☐	☐	☐	☐	☐	☐	☐	☐	☐	☐
Eg		☐	☐	☐	☐	☐	☐	☐	☐	☐	☐	☐	☐
Se		☐	☐	☐	☐	☐	☐	☐	☐	☐	☐	☐	☐
Pr		☐	☐	☐	☐	☐	☐	☐	☐	☐	☐	☐	☐
O		☐	☐	☐	☐	☐	☐	☐	☐	☐	☐	☐	☐

Q90 In the past 3 months, how often...

Please mark **one** *response for each item*	**Never**	**Less than once a month**	**1-3 times a month**	**Once a week**	**Several times a week**	**Every day**
Has your stomach felt bloated?	☐	☐	☐	☐	☐	☐
Have you required laxatives to ease constipation?	☐	☐	☐	☐	☐	☐
Have you required tablets to control diarrhoea?	☐	☐	☐	☐	☐	☐

Q91 Have you had a belly ache or abdominal pain (not including period pain) in the past 3 months?

☐ No → **Go to Section 6**
☐ Yes

↓

Q92 How often have you had a belly ache or abdominal pain in the past 3 months?

☐ Less than once a month
☐ 1 - 3 times a month
☐ Once a week
☐ Several times a week
☐ Everyday
☐ Less than once a month

SECTION 6 Physical Education and Physical Activity

If you no longer attend school please → Go to Q96

Q93 How many physical education classes do you usually attend at school each week?

- ☐ None → **Go to Q96**
- ☐ 1 per week
- ☐ 2 per week
- ☐ 3 or more per week

Q94 During physical education periods, how much time do you spend exercising that makes you out of breath or sweat?

- ☐ Not much time or none at all
- ☐ About a quarter of the time
- ☐ About half of the time
- ☐ More than half of the time
- ☐ Almost all of the time

Q95 What do you feel about your physical education periods?

- ☐ like them very much
- ☐ I like them
- ☐ I neither like nor dislike them
- ☐ I dislike them
- ☐ I dislike them very much
- ☐ I do not attend them

Q96 Outside school, TAFE or work hours:
How often do you usually exercise in your free time, so much that you get out of breath or sweat?

- ☐ Once a month or less
- ☐ Once a week
- ☐ 2 - 3 times a week
- ☐ 4 - 6 times a week
- ☐ Every day

Q97 Outside school, TAFE or work hours:
How many hours do you usually exercise in your free time, so much that you get out of breath or sweat?

- ☐ None
- ☐ About 1/2 hour per week
- ☐ About 1 hour per week
- ☐ About 2 - 3 hours per week
- ☐ About 4 - 6 hours per week
- ☐ 7 or more hours per week

We are interested in finding out about the kinds of physical activities that people do as part of their everyday lives. The following questions will ask you about the time you spent being physically active in the past 7 days.

Please answer each question even if you do not consider yourself to be an active person. Please think about the activities you do at work, as part of your house and yard work, to get from place to place, and in your spare time for recreation, exercise and sport.

Think about all the vigorous and moderate activities that you did in the past 7 days. Vigorous physical activities refer to activities that take hard physical effort and make you breathe much harder than normal. Moderate activities refer to activities that take moderate physical effort and make you breathe somewhat harder than normal.

Part 1: JOB RELATED PHYSICAL ACTIVITY

This first section is about your work/school/TAFE related physical activities. This includes paid jobs, farming, volunteer work, manual work carried out at school or TAFE, and any other unpaid work that you did outside your home. Do not include unpaid work that you might do around your home, like housework, yard work, general maintenance and caring for your family. These questions are asked later in part 3. Do not include sports or leisure activities as these are asked in part 4.

Q98 Do you currently have a job or do any unpaid work outside your home?

☐ No → **Go to Q105**
☐ Yes

↓

The next questions are about all the physical activity you did in the past 7 days (the past week) as part of your paid or unpaid work. This does not include travelling to and from work.

Q99 During the past 7 days, on how many days did you do vigorous physical activities like heavy lifting, digging, heavy construction or climbing up stairs as part of your work?
(think about only those vigorous physical activities that you did for at least 10 minutes at a time)

☐ days per week ☐ no vigorous job-related physical activity → **Go to Q101**

Q100 How much time did you usually spend on one of those days doing vigorous physical activities as part of your work?

☐☐ hours per day and ☐☐ minutes per day

Q101 Again, think about only those physical activities that you did for at least 10 minutes at a time. During the past 7 days, on how many days did you do moderate physical activities like carrying or lifting light loads as part of your work? (Please do not include walking)

☐ days per week ☐ no moderate job-related physical activity → **Go to Q103**

Q102 How much time did you usually spend on one of those days doing moderate physical activities as part of your work?

☐☐ hours per day and ☐☐ minutes per day

Q103 During the <u>past 7 days</u>, on how many days did you <u>walk</u> for at least 10 minutes at a time <u>as part of your work</u>? (Please do not count any walking you did to travel to or from work)

☐ days per week ☐ no job-related walking → **Go to Q105**

Q104 How much time did you usually spend on one of those days <u>walking</u> as part of your work?

☐☐ hours per day and ☐☐ minutes per day

Part 2: TRANSPORTATION PHYSICAL ACTIVITY

These questions are about how you travelled from place to place, including to and from work, school/TAFE, shopping stores, movies, doing errands and so on.

Q105 During the <u>past 7 days</u>, on how many days did you <u>travel in a motor vehicle</u> like a train, bus, car, tram?

☐ days per week ☐ no travelling in a motor vehicle → **Go to Q107**

Q106 How much time did you usually spend on one of those days <u>travelling</u> in a train, bus, car, tram or other kind of motor vehicle?

☐☐ hours per day and ☐☐ minutes per day

Now think only about the <u>bicycling</u> and <u>walking</u> you might have done to travel to and from school or work, to do errands, or to go from place to place.

Q107 During the <u>past 7 days</u>, on how many days did you <u>bicycle</u> for at least 10 minutes at a time to go <u>from place to place</u>?

☐ days per week ☐ no bicycling from place to place → **Go to Q109**

Q108 How much time did you usually spend on one of those days <u>bicycling</u> from place to place?

☐☐ hours per day and ☐☐ minutes per day

Q109 During the <u>past 7 days</u>, on how many days did you <u>walk</u> for at least 10 minutes at a time to go <u>from place to place</u>?

☐ days per week ☐ no walking from place to place → **Go to Q111**

Q110 How much time did you usually spend on one of those days <u>walking</u> from place to place?

☐☐ hours per day and ☐☐ minutes per day

Part 3: HOUSEWORK, HOUSE MAINTENANCE AND CARING FOR FAMILY

This part is about some of the physical activities you might have done in the past 7 days in and around your home, like housework, gardening, yard work, general maintenance work and caring for your family

Q111 Think about only those physical activities that you did for at least 10 minutes at a time. During the past 7 days, on how many days did you do vigorous physical activities like heavy lifting, chopping wood, shovelling snow or digging in the garden or yard?

☐ days per week ☐ no vigorous activity in garden or yard → **Go to Q113**

Q112 How much time did you usually spend on one of those days doing vigorous physical activities in the garden or yard?

☐☐ hours per day and ☐☐ minutes per day

Q113 Again, think about only those physical activities that you did for at least 10 minutes at a time. During the past 7 days, on how many days did you do moderate physical activities like carrying light loads, sweeping, washing windows and raking in the garden or yard?

☐ days per week ☐ no moderate activity in garden or yard → **Go to Q115**

Q114 How much time did you usually spend on one of those days doing moderate physical activities in the garden or yard?

☐☐ hours per day and ☐☐ minutes per day

Q115 Once again, think about only those physical activities you did for at least 10 minutes at a time. During the past 7 days, on how many days did you do moderate physical activities like carrying light loads, scrubbing floors, washing windows or sweeping inside your home?

☐ days per week ☐ no moderate activities inside home → **Go to Q117**

Q116 How much time did you usually spend on one of those days doing moderate physical activities inside your home?

☐☐ hours per day and ☐☐ minutes per day

Part 4: RECREATION, SPORT AND LEISURE-TIME PHYSICAL ACTIVITY

This part is about all the physical activities that you did in the past 7 days solely for recreation, sport, exercise or leisure. Please do not count any activities you have already mentioned in the previous several questions.

Q117 Not counting any walking you have already mentioned, during the past 7 days, on how many days did you walk for at least 10 minutes at a time in your leisure time?

☐ days per week ☐ no walking in leisure time → **Go to Q119**

Q118 How much time did you usually spend on one of those days walking in your leisure time?

☐☐ hours per day and ☐☐ minutes per day

Q119 Think about only those physical activities that you did for at least 10 minutes at a time. During the past 7 days, on how many days did you do vigorous physical activities like aerobics, running, fast bicycling or fast swimming in your leisure time?

☐ days per week ☐ no vigorous activity in leisure time → **Go to Q121**

Q120 How much time did you usually spend on one of those days doing vigorous physical activities in your leisure time?

☐☐ hours per day and ☐☐ minutes per day

Q121 Again, think about only those physical activities that you did for at least 10 minutes at a time. During the past 7 days, on how many days did you do moderate physical activities like bicycling at a regular pace, swimming at a regular pace or doubles tennis in your leisure time?

☐ days per week ☐ no moderate activity in leisure time → **Go to Q123**

Q122 How much time did you usually spend on one of these days doing moderate physical activities in your leisure time?

☐☐ hours per day and ☐☐ minutes per day

Part 5: TIME SPENT SITTING

This part is about the time you spend sitting while at work, school, TAFE, at home, while doing course work and during leisure time. This may include time spent sitting at a desk, visiting friends, reading or sitting down to watch television. Do not count time spent sitting in a motor vehicle or on a bicycle that you have previously mentioned.

Q123 During the past 7 days, how much time did you usually spend sitting ...

	Hours per day		Minutes per day
On a weekday?	☐☐	and	☐☐
On a weekend day?	☐☐	and	☐☐

Part 6: TIME SPENT STANDING

This question is about the time you spend standing while at work, school, TAFE, at home, and during leisure time. This may include time spent standing in a queue, in school assembly, or when talking with friends. Do not include time spent standing during sports/leisure time that you have already mentioned

Q124 During the past 7 days, how much time did you spend standing ...

	Hours per day		Minutes per day
On a weekday?	☐☐	and	☐☐
On a weekend day?	☐☐	and	☐☐

Part 7: TIME SPENT LYING DOWN

This question is about how much time you spend lying down. This should include time spent sleeping as well as any time spent lying on the sofa, bed, ground or lying down to watch television.

Q125 During the past 7 days, how much time did you spend lying down ...

	Hours per day		Minutes per day
On a weekday?	☐☐	and	☐☐
On a weekend day?	☐☐	and	☐☐

Q126 On average, how many hours per day do you usually…

Please mark **one** *response for each item*	Not at all	Less than 1 hour	About 1-2 hours	About 2-4 hours	More than 4 hours
Watch TV or videos on a week day	☐	☐	☐	☐	☐
Watch TV or videos on a weekend day	☐	☐	☐	☐	☐
Use a computer (e.g. for school/work, games, internet) on a week day	☐	☐	☐	☐	☐
Use a computer (e.g. for school/work, games, internet) on a weekend day	☐	☐	☐	☐	☐

Q127 How often do each of the following people play some sort of sport or exercise (e.g., golf, tennis, football) or other activity like walking for exercise, cycling or swimming?

Please mark **one** *response for each item*

	I don't have	Never or rarely	Sometimes	About once a week	2-3 times a week	More than 3 times a week	Don't know
Your Mother/female carer	☐	☐	☐	☐	☐	☐	☐
Your Father/male carer	☐	☐	☐	☐	☐	☐	☐
Your best friend/partner	☐	☐	☐	☐	☐	☐	☐

Q128 How often do each of the following people <u>praise you or encourage you</u> to play some sort of sport or to participate in other physical activity (e.g., watch you participate, say positive things to you, seem happy to do it)?

Please mark **one** *response for each item*

	I don't have	Never or rarely	Sometimes	About once a week	2-3 times a week	More than 3 times a week	Don't know
Your Mother/female carer	☐	☐	☐	☐	☐	☐	☐
Your Father/male carer	☐	☐	☐	☐	☐	☐	☐
Your best friend/partner	☐	☐	☐	☐	☐	☐	☐
A teacher at your school	☐	☐	☐	☐	☐	☐	☐

Q129 How often do each of the following people <u>help you</u> to play some sort of sport or to participate in other physical activity (e.g., take you to training, give sport money)?

Please mark **one** *response for each item*

	I don't have	Never or rarely	Sometimes	About once a week	2-3 times a week	More than 3 times a week	Don't know
Your Mother/female carer	☐	☐	☐	☐	☐	☐	☐
Your Father/male carer	☐	☐	☐	☐	☐	☐	☐
Your best friend/partner	☐	☐	☐	☐	☐	☐	☐
A teacher at your school	☐	☐	☐	☐	☐	☐	☐

Q130 Below are some reasons for not doing more exercise or activities than you do.
Please show how strongly each statement applies to you:

Please mark **one** *response for each line*	**Does not apply at all**	**Applies a little**	**Applies a fair amount**	**Applies strongly**	**Applies very strongly**
I already do a lot of exercise	☐	☐	☐	☐	☐
I am self-conscious about my looks when I exercise or play sport	☐	☐	☐	☐	☐
I don't have enough time	☐	☐	☐	☐	☐
I don't have enough energy	☐	☐	☐	☐	☐
There are other things I like doing more	☐	☐	☐	☐	☐
I don't have anyone to exercise or play with	☐	☐	☐	☐	☐
I just don't enjoy exercise or sport	☐	☐	☐	☐	☐
My parents don't encourage or help me	☐	☐	☐	☐	☐
The right facilities are not available	☐	☐	☐	☐	☐
I don't have the skills	☐	☐	☐	☐	☐
I am just not very good at any sports or activities	☐	☐	☐	☐	☐
Others laugh/make fun of me when I try to play	☐	☐	☐	☐	☐
My health is not good enough	☐	☐	☐	☐	☐
I have an injury which prevents me	☐	☐	☐	☐	☐
Some other reason *(please specify):* ………………………………………………………………	☐	☐	☐	☐	☐

Q131 Do you have any concerns or worries about your health or development? (e.g. speech, language development, physical development, emotional development)

☐ No → **Go to Q132**
☐ Yes

Please tell us about these concerns if you wish to do so in the space provided:

Q132 Date questionnaire completed: ☐☐ / ☐☐ / ☐☐☐☐

Q133 Please write below any comments concerning this questionnaire, the research or anything else you would like to tell us about:

THANK YOU

WE APPRECIATE THE TIME THAT YOU HAVE SPENT COMPLETING THIS QUESTIONNAIRE

ID | | | | |

OFFICE USE ONLY

RA-CH ☐☐ RA-CO ☐☐ RA1-E ☐☐ RA2-E ☐☐

ID ☐☐☐☐☐

GROWING UP HEALTHY STUDY

Participant Confidential Questionnaire

FEMALE

16-18

The purpose of this teenager questionnaire is to obtain information about a range of topics including your relationships at home, school and work, your self-confidence and perceptions of care and support that you receive from others, your health and recent medical history, and your knowledge around and participation in risk taking behaviours
such as smoking, drinking and sexual activity

Please read each question carefully and answer all of the questions.
Write your answers clearly in the space provided or mark the most appropriate response

☒

Please take your time in answering all of the questions

If you are uncomfortable about a question or unsure of an answer, please leave it blank or ask one of the Growing Up Healthy Study team for assistance

Please complete this questionnaire independently (without discussing it with anyone)

Remember all answers are STRICTLY confidential

Questionnaire

Today's date: ☐☐ / ☐☐ / ☐☐☐☐

Are you **FEMALE**?

☐ Yes
☐ No (Please ask one of the Growing Up Healthy Study team for assistance)

SECTION 1 Eating Habits, Weight, Physical Health and Development

Q1 How often do you eat the following foods?

Please mark **one** *response for each item*	6 + times a week	3-5 times a week	1-2 times a week	1-2 times a month	Rarely or never
**1.** Fried food with a batter or breadcrumb coating	☐	☐	☐	☐	☐
**2.** Gravy, creamy sauces or cheese sauces	☐	☐	☐	☐	☐
**3.** Vegetables, rice or pasta with added butter, margarine, oil or sour cream	☐	☐	☐	☐	☐
**4.** Vegetables that are fried or roasted with fat or oil (don't count oil sprays)	☐	☐	☐	☐	☐
**5.** Sausages, polony, salami, meat pies, pasties, hamburger or bacon	☐	☐	☐	☐	☐
**6.** Hot potato chips or French fries	☐	☐	☐	☐	☐
**7.** Pastries, cakes, sweet biscuits or croissants	☐	☐	☐	☐	☐
**8.** Chocolate, chocolate biscuits or sweet snack bars	☐	☐	☐	☐	☐
**9.** Potato crisps, corn chips, cheezels, twisties or nuts	☐	☐	☐	☐	☐
**10.** Ice cream (any variety)	☐	☐	☐	☐	☐
**11.** Cream or sour cream	☐	☐	☐	☐	☐
**12.** Cheddar, edam or other hard cheese, cream cheese or soft cheeses such as camembert or brie (but excluding ricotta or cottage cheese)	☐	☐	☐	☐	☐

Q2 How much of the following do you usually eat? (*Please mark* **one** *response for each item)*

a Fat on meat?

☐ Most or all
☐ Some
☐ None
☐ Don't eat meat

b Skin on chicken?

☐ Most or all
☐ Some
☐ None
☐ Don't eat chicken

Q3 How often do you eat the following foods?

Please mark **one** *response for each item*	6 + times a week	3-5 times a week	1-2 times a week	1-2 times a month	Rarely or never
**Fruit**, including fresh and canned fruit (do not include dried fruit, fruit juices, fruit drinks, fruit bars or frozen fruit desserts)	☐	☐	☐	☐	☐
**Vegetables**, including all forms of vegetables, e.g. fresh, frozen, canned and salads	☐	☐	☐	☐	☐
**Fish**	☐	☐	☐	☐	☐

Q4 How many days per week do you usually have something for breakfast?

Rarely or never	1-2 days per week	3-4 days per week	5-6 days per week	Everyday
☐	☐	☐	☐	☐

Q5 How many days per week did you eat your evening meal with the family (including at least one adult)?

Rarely or never	1-4 days per week	5-7 days per week	Irregularly
☐	☐	☐	☐

Q6 How often do you eat meals or snacks from fast food chains (e.g. MacDonalds, Hungry Jacks, Pizza Hut, Red Rooster, River Rooster, Kentucky Fried)?

Never	Once per fortnight or less	Once per week	2-4 times per week	5-7 time per week
☐	☐	☐	☐	☐

Q7 Did you eat special foods or have a special diet over the past 12 months?

☐ No → **Go to Q9**
☐ Yes

Q8 Why were you on a special diet? *(Please mark* **all** *responses that apply to you)*

☐ Vegetarian
☐ To avoid milk
☐ For diabetes
☐ For allergy
☐ For asthma
☐ For behaviour
☐ For sport
☐ To lose weight
☐ To gain weight, build muscles
☐ Other reason (*Please specify)*……………………………….

Q9 How many serves of fruit do you usually eat each day?
(1 serve = 1 medium piece or 2 small pieces of fruit or 1 cup of diced pieces)

Rarely eat fruit	1 serve or less per day	2 serves per day	3 serves per day	4 serves or more per day
☐	☐	☐	☐	☐

Q10 How many serves of vegetables do you usually eat each day?
(1 serve =1/2 cup cooked vegetables or 1 cup of salad vegetables)

Rarely eat vegetables	1 serves or less per day	2 serves per day	3 serves per day	4 serves per day	5 serves per day	6 serves per day
☐	☐	☐	☐	☐	☐	☐

Q11 Here we are asking for some additional information on <u>how often</u> and <u>how much</u> of the following drinks you usually consume.

When answering these questions, please mark how often you have the drink and write the total number of glasses, cans, or cups you would usually drink (see example). To assist you, below each type of drink is the type of measurement.

	Never	less than once/ month	**1** day/ month	**2** days/ month	**3** days/ month	**1** day/ week	**2** days/ week	**3** days/ week	**4** days/ week	**5** days/ week	**6** days/ week	every day	**Total number of glasses/ cups/cans you usually drink**
***i.e.* Water (250 ml glass)**	☐	☐	☐	☐	☐	☐	☐	☐	☐	☐	☒	☐	**8**
**1.** Water (250 ml glass)	☐	☐	☐	☐	☐	☐	☐	☐	☐	☐	☐	☐	
**2.** Fizzy drink (e.g. cola, lemonade) (can, glass)	☐	☐	☐	☐	☐	☐	☐	☐	☐	☐	☐	☐	
**3.**Diet fizzy drink (e.g. diet cola, diet lemonade) (can, glass)	☐	☐	☐	☐	☐	☐	☐	☐	☐	☐	☐	☐	
**4.** Energy drink (e.g. Redbull, V, Monster) (can)	☐	☐	☐	☐	☐	☐	☐	☐	☐	☐	☐	☐	
**5.** Diet energy drink (can)	☐	☐	☐	☐	☐	☐	☐	☐	☐	☐	☐	☐	
**6.** Tea (cup)	☐	☐	☐	☐	☐	☐	☐	☐	☐	☐	☐	☐	
**7.** Herbal tea (cup)	☐	☐	☐	☐	☐	☐	☐	☐	☐	☐	☐	☐	
**8.** Green tea (cup)	☐	☐	☐	☐	☐	☐	☐	☐	☐	☐	☐	☐	
**9.** Instant coffee (cup)	☐	☐	☐	☐	☐	☐	☐	☐	☐	☐	☐	☐	
**10.** Ground coffee (e.g. filter coffee, cappuccino, flat white) (cup, mug)	☐	☐	☐	☐	☐	☐	☐	☐	☐	☐	☐	☐	

OFFICE USE ONLY
Q11
1☐☐ 2☐☐ 3☐☐ 4☐☐ 5☐☐ 6☐☐ 7☐☐ 8☐☐ 9☐☐ 10☐☐

Q12 Do you know how much you weigh?

☐ No → **Go to Q14**
☐ Yes

↓

Q13 What is your current weight? ☐☐☐ . ☐ kg

Please mark only **one** *response for each question below*

Q14 Are you worried about your weight?

**No, not at all**	**A little**	**Moderately**	**Very**
☐	☐	☐	☐

Q15 Do you consider yourself to be?

**Underweight**	**Normal weight**	**A bit overweight**	**Very overweight**
☐	☐	☐	☐

Q16 How often do you weigh yourself?

**Never**	**Once in a while**	**Often**	**Nearly every day**
☐	☐	☐	☐

Q17 **Over the last two weeks…**

1. Have you been trying hard to eat less to change your shape or weight (even if you haven't managed to do so)?

**Not at all**	**Some of the time**	**A lot of the time**	**Most of the time**
☐	☐	☐	☐

2. Have you gone for long periods of time (8 hours or more) without eating anything to try and change your shape or weight?

**Not at all**	**Some of the time**	**A lot of the time**	**Most of the time**
☐	☐	☐	☐

3. Have you tried not to eat certain foods (like chocolate or chips to try to change your shape or weight (even if you haven't been able to do so)?

**Not at all**	**Some of the time**	**A lot of the time**	**Most of the time**
☐	☐	☐	☐

4. Have you tried to stick to any definite rules about diet or eating (e.g., sticking to a calorie limit, a set amount of food or rules about what or when you should eat (even if you haven't been able to do so)?

**Not at all**	**Some of the time**	**A lot of the time**	**Most of the time**
☐	☐	☐	☐

5. Have you been thinking about food or calories so much that you've found it hard to concentrate on things you are interested in (e.g., reading, watching TV or following a conversation)?

**Not at all**	**Some of the time**	**A lot of the time**	**Most of the time**
☐	☐	☐	☐

6. Have there been times when you feel that you have eaten an unusually large amount of food (more than what most people would eat in the same situation)?

**Not at all**	**Some of the time**	**A lot of the time**	**Most of the time**
☐	☐	☐	☐

7. Have you been afraid of losing control over your eating?

**Not at all**	**Some of the time**	**A lot of the time**	**Most of the time**
☐	☐	☐	☐

8. Have you felt that you couldn't control what or how much you were eating?

**Not at all**	**Some of the time**	**A lot of the time**	**Most of the time**
☐	☐	☐	☐

9. Have you felt that you couldn't stop eating once you had started?

**Not at all**	**Some of the time**	**A lot of the time**	**Most of the time**
☐	☐	☐	☐

10. Have you felt guilty after eating?

**Not at all**	**Some of the time**	**A lot of the time**	**Most of the time**
☐	☐	☐	☐

11. Have you eaten in secret because you are embarrassed by how much you eat?

**Not at all**	**Some of the time**	**A lot of the time**	**Most of the time**
☐	☐	☐	☐

12. Have you been afraid that you might gain weight or become fat?

**Not at all**	**Some of the time**	**A lot of the time**	**Most of the time**
☐	☐	☐	☐

13. Have you felt fat?

**Not at all**	**Some of the time**	**A lot of the time**	**Most of the time**
☐	☐	☐	☐

14. Have you had a strong desire to lose weight?

**Not at all**	**Some of the time**	**A lot of the time**	**Most of the time**
☐	☐	☐	☐

15. Have you made yourself sick (vomit) after eating to control your weight?

**Not at all**	**Some of the time**	**A lot of the time**	**Most of the time**
☐	☐	☐	☐

16. Have you taken any pills (like laxatives, water pills, diet tubes) to try to control your weight?

**Not at all**	**Some of the time**	**A lot of the time**	**Most of the time**
☐	☐	☐	☐

17. Have you exercised hard to control your weight?

**Not at all**	**Some of the time**	**A lot of the time**	**Most of the time**
☐	☐	☐	☐

People have different ideas about what sort of things are important to them in how they think about themselves. For some people doing well at school is very important to them, for others, how they are getting on with friends is very important. We're now going to ask you to think about how important weight and shape is to you:

18. Has your weight been important in how you think of yourself as a person?

**Not at all**	**Some of the time**	**A lot of the time**	**Most of the time**
☐	☐	☐	☐

19. Has your shape been important in how you think of yourself as a person?

**Not at all**	**Some of the time**	**A lot of the time**	**Most of the time**
☐	☐	☐	☐

Q18 Please look at the figures and select from the list of numbers and letters A and B provided:

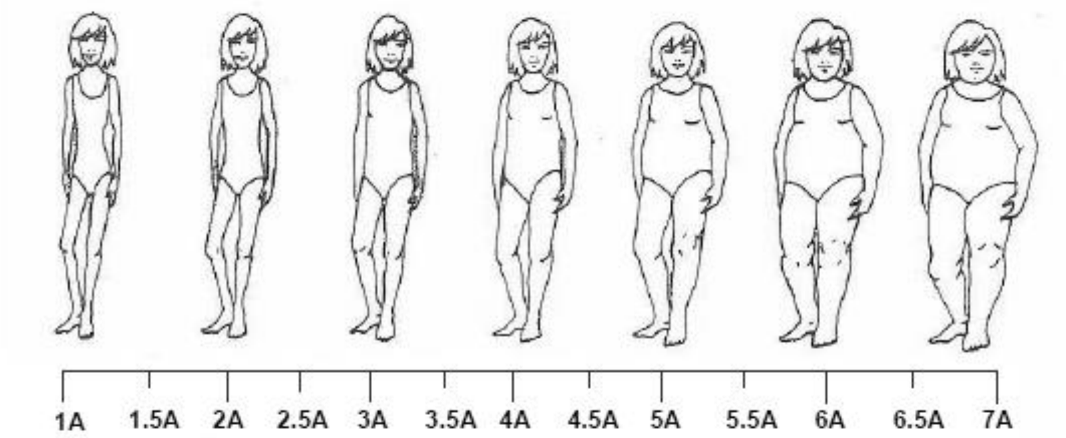

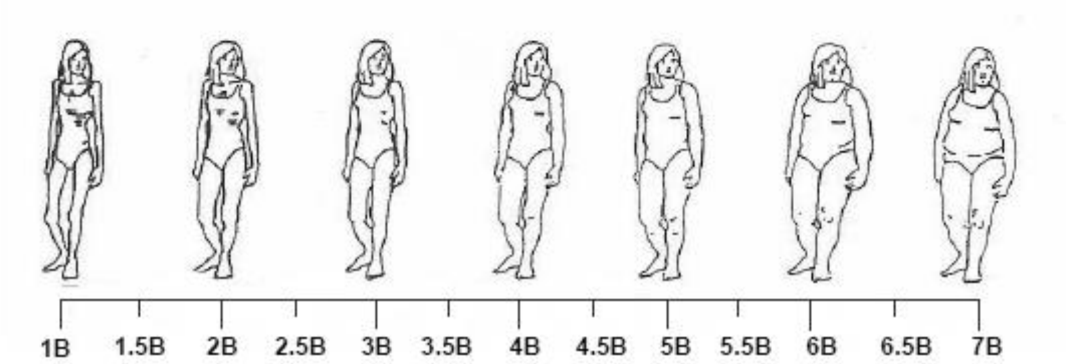

	Number	***Letter***
Which figure best represents what you currently look like?	☐	☐
Which figure would you most like to look like?	☐	☐

The drawings on this page show different amounts of female pubic hair. A girl passes through each of the four stages shown by these drawings.

19*a* Please look at each drawing then choose the one closest to your stage of development by placing an X in the corresponding box.

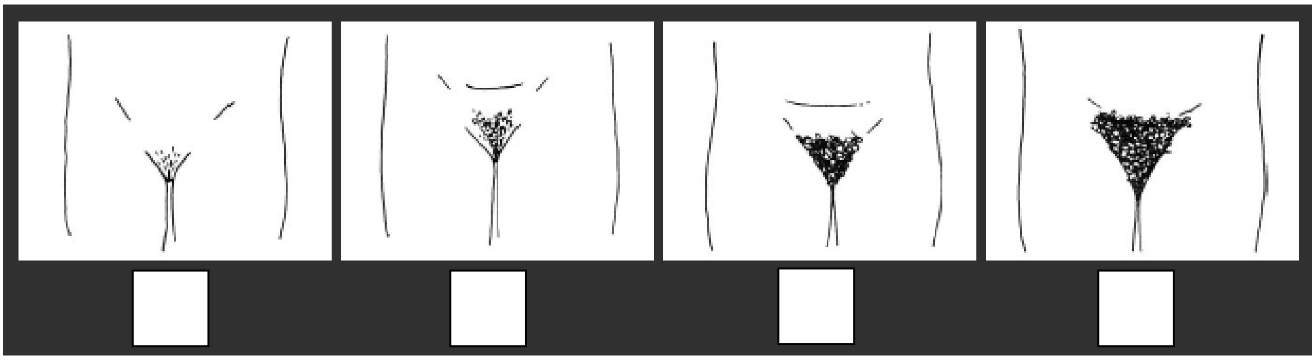

The drawings on this page show different stages of development of the breasts. A female passes through each of the five stages shown by these drawings.

19*b* Please look at each drawing then choose the one closest to your stage of development by placing an X in the corresponding box.

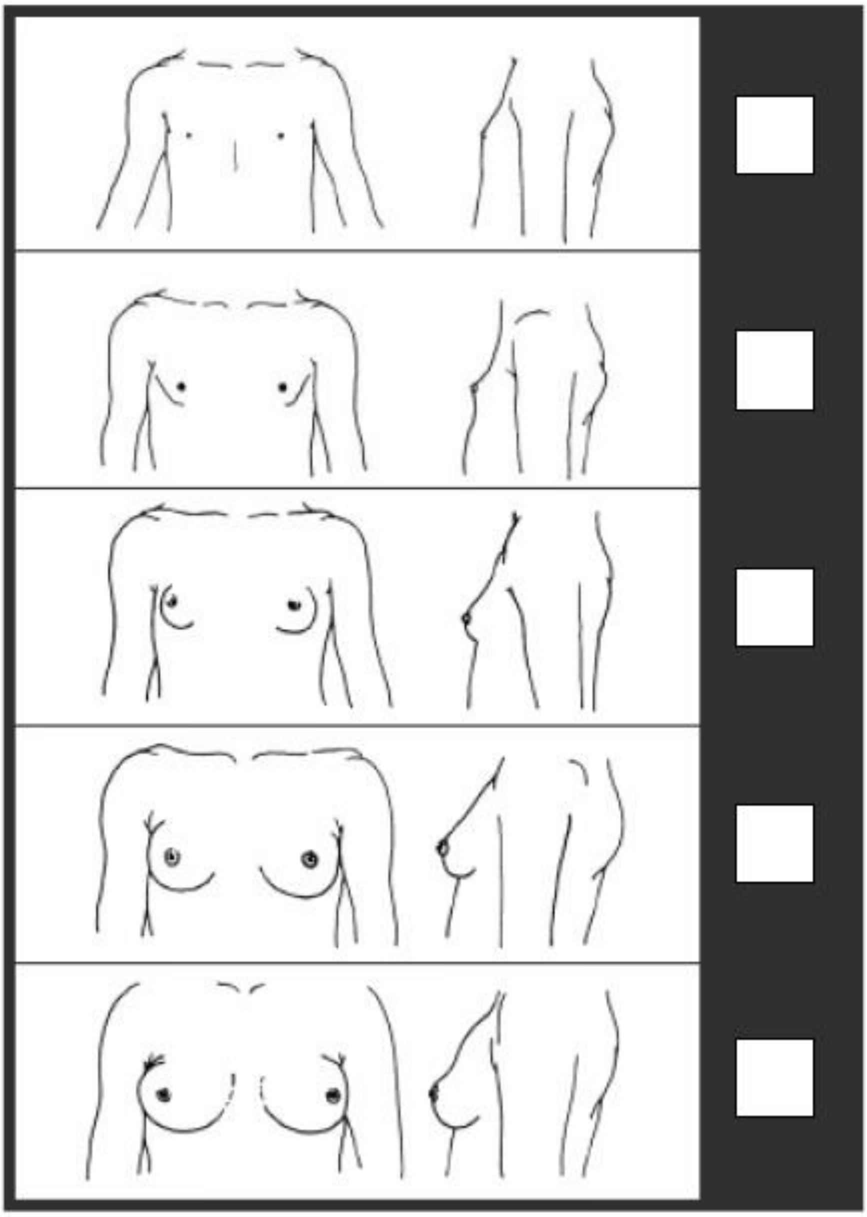

Q20 Below is a list of items that describes adolescents. For each item please mark the box that best represents whether the statement is true, somewhat/sometimes true or very true/often true for you now or within the past 6 months. Please answer all items as well as you can, even if some do not seem to apply to you:

Please mark **one** *response for each item*	**Not true**	**Somewhat/ sometimes true**	**Very true/ often true**
**1.** I act too young for my age	☐	☐	☐
**2.** I have an allergy	☐	☐	☐
**3.** I argue a lot	☐	☐	☐
**4.** I have asthma	☐	☐	☐
**5.** I like the opposite sex	☐	☐	☐
**6.** I like animals	☐	☐	☐
**7.** I brag	☐	☐	☐
**8.** I have trouble concentrating or paying attention	☐	☐	☐
**9.** I can't get my mind off certain thoughts	☐	☐	☐
**10.** I have trouble sitting still	☐	☐	☐
**11.** I am too dependent on adults	☐	☐	☐
**12.** I feel lonely	☐	☐	☐
**13.** I feel confused or in a fog	☐	☐	☐
**14.** I cry a lot	☐	☐	☐
**15.** I am pretty honest	☐	☐	☐
**16.** I am mean to others	☐	☐	☐
**17.** I day dream a lot	☐	☐	☐
**18.** I deliberately try to hurt or kill myself	☐	☐	☐
**19.** I try to get a lot of attention	☐	☐	☐
**20.** I destroy my own things	☐	☐	☐
**21.** I destroy things belonging to others	☐	☐	☐
**22.** I disobey my parents	☐	☐	☐
**23.** I disobey at school	☐	☐	☐
**24.** I don't eat as well as I should	☐	☐	☐
**25.** I don't get along with other kids	☐	☐	☐
**26.** I don't feel guilty after doing something I shouldn't	☐	☐	☐
**27.** I am jealous of others	☐	☐	☐
**28.** I am willing to help others when they need help	☐	☐	☐
**29.** I am afraid of certain animals, situations, or places other than school	☐	☐	☐
**30.** I am afraid of going to school	☐	☐	☐

Please mark **one** *response for each item*	**Not true**	**Somewhat/ sometimes true**	**Very true/ often true**
**31.** I am afraid I might think or do something bad	☐	☐	☐
**32.** I feel that I have to be perfect	☐	☐	☐
**33.** I feel that no one loves me	☐	☐	☐
**34.** I feel that others are out to get me	☐	☐	☐
**35.** I feel worthless or inferior	☐	☐	☐
**36.** I accidentally get hurt a lot	☐	☐	☐
**37.** I get in many fights	☐	☐	☐
**38.** I get teased a lot	☐	☐	☐
**39.** I hang around with kids who get in trouble	☐	☐	☐
**40.** I hear sounds or voices that other people think aren't there	☐	☐	☐
**41.** I act without stopping to think	☐	☐	☐
**42.** I would rather be alone than with others	☐	☐	☐
**43.** I lie or cheat	☐	☐	☐
**44.** I bite my fingernails	☐	☐	☐
**45.** I am nervous or tense	☐	☐	☐
**46.** Parts of my body twitch or make nervous movements	☐	☐	☐
**47.** I have nightmares	☐	☐	☐
**48.** I am not liked by other kids	☐	☐	☐
**49.** I can do certain things better than most kids	☐	☐	☐
**50.** I am too fearful or anxious	☐	☐	☐
**51.** I feel dizzy	☐	☐	☐
**52.** I feel too guilty	☐	☐	☐
**53.** I eat too much	☐	☐	☐
**54.** I feel overtired	☐	☐	☐
**55.** I am overweight	☐	☐	☐
**56.** Physical problems without known medical cause:			
a. Aches or pains (not headaches)	☐	☐	☐
b. Headaches	☐	☐	☐
c. Nausea, feel sick	☐	☐	☐
d. Problems with eyes	☐	☐	☐
e. Rashes or other skin problems	☐	☐	☐
f. Stomach-aches or cramps	☐	☐	☐
g. Vomiting, throwing up	☐	☐	☐
h. Other *(please describe)*…………………………..	☐	☐	☐

Please mark **one** *response for each item*	**Not true**	**Somewhat/ sometimes true**	**Very true/ often true**
**57.** I physically attack people	☐	☐	☐
**58.** I pick my skin or other parts of my body	☐	☐	☐
**59.** I can be pretty friendly	☐	☐	☐
**60.** I like to try new things	☐	☐	☐
**61.** My school work is poor	☐	☐	☐
**62.** I am poorly coordinated or clumsy	☐	☐	☐
**63.** I would rather be with older kids than kids my own age	☐	☐	☐
**64.** I would rather be with younger kids than kids my own age	☐	☐	☐
**65.** I refuse to talk	☐	☐	☐
**66.** I repeat certain actions over and over	☐	☐	☐
**67.** I run away from home	☐	☐	☐
**68.** I scream a lot	☐	☐	☐
**69.** I am secretive or keep things to myself	☐	☐	☐
**70.** I see things that other people think aren’t there	☐	☐	☐
**71.** I am self-conscious or easily embarrassed	☐	☐	☐
**72.** I set fires	☐	☐	☐
**73.** I can work well with my hands	☐	☐	☐
**74.** I show off or clown	☐	☐	☐
**75.** I am shy	☐	☐	☐
**76.** I sleep less than most kids	☐	☐	☐
**77.** I sleep more than most kids during the day and/or night	☐	☐	☐
**78.** I have a good imagination	☐	☐	☐
**79.** I have a speech problem	☐	☐	☐
**80.** I stand up for my rights	☐	☐	☐
**81.** I steal at home	☐	☐	☐
**82.** I steal from places other than home	☐	☐	☐
**83.** I store things up I don’t need	☐	☐	☐
**84.** I do things other people think are strange	☐	☐	☐
**85.** I have thoughts that other people would think are strange	☐	☐	☐
**86.** I am stubborn	☐	☐	☐
**87.** My moods or feelings change suddenly	☐	☐	☐
**88.** I enjoy being with other people	☐	☐	☐
**89.** I am suspicious	☐	☐	☐
**90.** I swear or use dirty language	☐	☐	☐

Please mark **one** *response for each item*	Not true	Somewhat/ sometimes true	Very true/ often true
**91.** I think about killing myself	☐	☐	☐
**92.** I like to make others laugh	☐	☐	☐
**93.** I talk too much	☐	☐	☐
**94.** I tease others a lot	☐	☐	☐
**95.** I have a hot temper	☐	☐	☐
**96.** I think about sex too much	☐	☐	☐
**97.** I threaten to hurt people	☐	☐	☐
**98.** I like to help others	☐	☐	☐
**99.** I am too concerned about being neat or clean	☐	☐	☐
**100.** I have trouble sleeping	☐	☐	☐
**101.** I skip classes or wag school	☐	☐	☐
**102.** I don't have much energy	☐	☐	☐
**103.** I am unhappy, sad or depressed	☐	☐	☐
**104.** I am louder than other kids	☐	☐	☐
**105.** I use alcohol or drugs for nonmedical purposes	☐	☐	☐
**106.** I try to be fair to others	☐	☐	☐
**107.** I enjoy a good joke	☐	☐	☐
**108.** I like to take life easy	☐	☐	☐
**109.** I try to help other people when I can	☐	☐	☐
**110.** I wish I were of the opposite sex	☐	☐	☐
**111.** I keep from getting involved with others	☐	☐	☐
**112.** I worry a lot	☐	☐	☐

SECTION 2 Bullying, Mental Health and Development

The next questions are about bullying at school: Bullying is when someone is picked on by another person, or a group of people say nasty and unpleasant things to him or her. It is also when someone is hit, kicked, threatened, sent nasty notes, when no one talks to them and things like that. *Please mark* **one** *response for each item:*

Q21 Have you ever been bullied at school/TAFE/Uni or at work?

☐ No → **Go to Q25**
☐ Yes

↓

Q22 Has this happened at your current school/TAFE/Uni or workplace?

☐ No
☐ Yes

Q23 Was this in the past three months?

- ☐ No
- ☐ Yes

Q24 How did you feel about being bullied? *(Please mark* **all** *responses that apply to you)*

- ☐ Made you sad
- ☐ Made you angry
- ☐ Doesn't bother you
- ☐ Stressed you out
- ☐ Other feelings (*Please specify*) ……………………………………

Q25 Have you ever bullied other kids?

- ☐ No
- ☐ Yes

Q26 Are you satisfied with the way your school/TAFE/Uni workplace handles bullying?

- ☐ Very satisfied
- ☐ Fairly satisfied
- ☐ Unsatisfied (they could do a lot more)
- ☐ Very unsatisfactory (nothing is done about it)

Q27 Have any of the following things ever happened to you?

Please mark ***one*** *response for each item*	**4 or more times**	**2 or 3 times**	**Once**	**Never**
**1.** You have been treated with less courtesy than other people	☐	☐	☐	☐
**2.** You have been treated with less respect than other people	☐	☐	☐	☐
**3.** You have received poorer service than other people at restaurants or shops	☐	☐	☐	☐
**4.** People have acted as if they think you are not smart	☐	☐	☐	☐
**5.** People have acted as if they are afraid of you	☐	☐	☐	☐
**6.** People have acted as if they think you are dishonest	☐	☐	☐	☐
**7.** People have acted as if they're better than you are	☐	☐	☐	☐
**8.** You have been called names or insulted	☐	☐	☐	☐
**9.** You have been threatened or harassed	☐	☐	☐	☐
**10.** You have been followed around in shops	☐	☐	☐	☐

→ ***Go to Q29 if you didn't have any of these experiences***

Q28 If you had any of these happen to you what do you think were the main reasons for it? *(Please mark **all** responses that apply to you)*

- ☐ Your ancestry or national origins
- ☐ Your gender
- ☐ Your race
- ☐ Your age
- ☐ Your religion
- ☐ Your height or weight
- ☐ Your shade of skin colour
- ☐ Your sexual orientation
- ☐ Your education or income level
- ☐ A physical disability
- ☐ The way you look
- ☐ Other (*Please specify*) ……………………………………………

Q29 Below is a list of statements dealing with your general feelings about yourself. Please mark the response for how much you agree or disagree with each statement:

*Please mark **one** response for each item*	**Strongly agree**	**Agree**	**Disagree**	**Strongly disagree**
**1.** On the whole, I am satisfied with myself	☐	☐	☐	☐
**2.** At times, I think I am no good at all	☐	☐	☐	☐
**3.** I feel that I have a number of good qualities	☐	☐	☐	☐
**4.** I am able to do things as well as most other people	☐	☐	☐	☐
**5.** I feel I do not have much to be proud of	☐	☐	☐	☐
**6.** I certainly feel useless at times	☐	☐	☐	☐
**7.** I feel that I'm a person of worth, at least on an equal level with others	☐	☐	☐	☐
**8.** I wish I could have more respect for myself	☐	☐	☐	☐
**9.** All in all, I am inclined to feel that I am a failure	☐	☐	☐	☐
**10.** I take a positive attitude toward myself	☐	☐	☐	☐

Q30 This question is about how you feel right now. Please read each statement carefully and mark the response that best describes how you feel. There are no right or wrong answers. Don't spend too much time on any one statement. For each statement below, please mark the response which best describes how best you feel right now, at this very moment:

**1.**	I feel…	☐ Very calm	☐ Calm	☐ Not calm
**2.**	I feel…	☐ Very upset	☐ Upset	☐ Not upset
**3.**	I feel…	☐ Very pleasant	☐ Pleasant	☐ Not pleasant
**4.**	I feel…	☐ Very nervous	☐ Nervous	☐ Not nervous
**5.**	I feel…	☐ Very jittery	☐ Jittery	☐ Not jittery
**6.**	I feel…	☐ Very rested	☐ Rested	☐ Not rested
**7.**	I feel…	☐ Very scared	☐ Scared	☐ Not scared
**8.**	I feel…	☐ Very relaxed	☐ Relaxed	☐ Not relaxed
**9.**	I feel…	☐ Very worried	☐ Worried	☐ Not worried
**10.**	I feel…	☐ Very satisfied	☐ Satisfied	☐ Not satisfied
**11.**	I feel…	☐ Very frightened	☐ Frightened	☐ Mot frightened
**12.**	I feel…	☐ Very happy	☐ Happy	☐ Not happy
**13.**	I feel…	☐ Very sure	☐ Sure	☐ Not sure
**14.**	I feel…	☐ Very good	☐ Good	☐ Not good
**15.**	I feel…	☐ Very troubled	☐ Troubled	☐ Not troubled
**16.**	I feel…	☐ Very bothered	☐ Bothered	☐ Not bothered
**17.**	I feel…	☐ Very nice	☐ Nice	☐ Not nice
**18.**	I feel…	☐ Very terrified	☐ Terrified	☐ Not terrified
**19.**	I feel…	☐ Very mixed-up	☐ Mixed-up	☐ Not mixed-up
**20.**	I feel…	☐ Very cheerful	☐ Cheerful	☐ Not cheerful

Q31 A number of statements which boys and girls use to describe themselves are listed below. Read each statement carefully and decide if it is hardly-ever, or sometimes, or often true for you. There are no right or wrong answers. Don't spend too much time on any one statement. For each statement, mark the response that seems to describe you best. Remember to choose the word which best seems to describe how you usually feel:

Please mark **one** *response for each item*	**Hardly ever**	**Sometimes**	**Often**
**1.** I worry about making mistakes	☐	☐	☐
**2.** I feel like crying	☐	☐	☐
**3.** I feel unhappy	☐	☐	☐
**4.** I have trouble making up my mind	☐	☐	☐
**5.** It is difficult for me to face my problems	☐	☐	☐
**6.** I worry too much	☐	☐	☐
**7.** I get upset at home	☐	☐	☐
**8.** I am shy	☐	☐	☐
**9.** I feel troubled	☐	☐	☐
**10.** Unimportant thoughts run through my mind and bother me	☐	☐	☐
**11.** I worry about school / work	☐	☐	☐
**12.** I have trouble deciding what to do	☐	☐	☐
**13.** I notice my heart beats fast	☐	☐	☐
**14.** I am secretly afraid	☐	☐	☐
**15.** I worry about my parents	☐	☐	☐
**16.** My hands get sweaty	☐	☐	☐
**17.** I worry about things that may happen	☐	☐	☐
**18.** It is hard for me to fall asleep at night	☐	☐	☐
**19.** I get a funny feeling in my stomach	☐	☐	☐
**20.** I worry about what others may think of me	☐	☐	☐

Q32 In some situations we feel sure that we can manage well and make things turn out the way we want; in other situations we feel less sure of managing well and less able to make things turn out the way we want. Please select the response that shows how sure you feel in managing each of the following situations. There are no right or wrong answers - just say what you think would be true for you:

Please mark **one** *response for each item*	**Not at all sure**	**A little sure**	**Some-what sure**	**Quite sure**	**Very sure**
**1.** You meet a person for the first time	☐	☐	☐	☐	☐
**2.** You are in a place you don't know anything about	☐	☐	☐	☐	☐
**3.** You have new work to do at school /Work/TAFE	☐	☐	☐	☐	☐
**4.** You have to get something done and there is a lot of pressure	☐	☐	☐	☐	☐
**5.** You have to work out a problem with a teacher/ lecturer/ employer	☐	☐	☐	☐	☐
**6.** You have to work out a problem with your mother	☐	☐	☐	☐	☐
**7.** You have to give a talk in front of people	☐	☐	☐	☐	☐
**8.** You have to do something for the first time	☐	☐	☐	☐	☐
**9.** You have to travel to a new place by yourself	☐	☐	☐	☐	☐
**10.** You have to work out a problem with a friend	☐	☐	☐	☐	☐
**11.** You have trouble solving a problem in school/work/TAFE	☐	☐	☐	☐	☐
**12.** You feel very unhappy	☐	☐	☐	☐	☐
**13.** You lose something important	☐	☐	☐	☐	☐
**14.** You have to do things people expect you to do	☐	☐	☐	☐	☐
**15.** You have to figure out something by yourself	☐	☐	☐	☐	☐
**16.** You have to make an important decision	☐	☐	☐	☐	☐
**17.** Someone counts on you to do something important	☐	☐	☐	☐	☐
**18.** You are bored and want to find something interesting to do	☐	☐	☐	☐	☐
**19.** Things are going wrong	☐	☐	☐	☐	☐
**20.** You become older	☐	☐	☐	☐	☐
**21.** You have to work out a problem with your father	☐	☐	☐	☐	☐
**22.** You have done something wrong	☐	☐	☐	☐	☐

Q33 Here is a list of things that happen to people and that people think or feel. Please read each statement carefully and thinking over the past two weeks, select the response that best describes how you feel about each statement. There are no right or wrong answers:

Please mark **one** *response for each item*	**Never**	**Sometimes**	**Often**	**Always**
**1.** I think that my life is bad	☐	☐	☐	☐
**2.** I have trouble doing things	☐	☐	☐	☐
**3.** I feel that I am a bad person	☐	☐	☐	☐
**4.** I wish I were dead	☐	☐	☐	☐
**5.** I have trouble sleeping	☐	☐	☐	☐
**6.** I feel no one loves me	☐	☐	☐	☐
**7.** I think bad things happen because of me	☐	☐	☐	☐
**8.** I feel lonely	☐	☐	☐	☐
**9.** My Stomach hurts	☐	☐	☐	☐
**10.** I feel like bad things happen to me	☐	☐	☐	☐
**11.** I feel like I am stupid	☐	☐	☐	☐
**12.** I feel sorry for myself	☐	☐	☐	☐
**13.** I think I do things badly	☐	☐	☐	☐
**14.** I feel bad about what I do	☐	☐	☐	☐
**15.** I hate myself	☐	☐	☐	☐
**16.** I want to be alone	☐	☐	☐	☐
**17.** I feel like crying	☐	☐	☐	☐
**18.** I feel sad	☐	☐	☐	☐
**19.** I feel empty inside	☐	☐	☐	☐
**20.** I think my life will be bad	☐	☐	☐	☐

Q34 The following statements have been used by many people to describe how much support they get from other people. How much do you agree or disagree with each of these statements? The more you agree the higher the number you should mark. The more you disagree, the lower the number you should mark:

Please mark **one** *response for each item*	**Disagree**						**Agree**
**1.** People don't come to visit me as often as I would like	☐ 1	☐ 2	☐ 3	☐ 4	☐ 5	☐ 6	☐ 7
**2.** I often need help from other people but can't get it	☐ 1	☐ 2	☐ 3	☐ 4	☐ 5	☐ 6	☐ 7
**3.** I seem to have a lot of friends	☐ 1	☐ 2	☐ 3	☐ 4	☐ 5	☐ 6	☐ 7
**4.** I don't have anyone that I can confide in	☐ 1	☐ 2	☐ 3	☐ 4	☐ 5	☐ 6	☐ 7
**5.** I have no one to lean on in times of trouble	☐ 1	☐ 2	☐ 3	☐ 4	☐ 5	☐ 6	☐ 7
**6.** There is someone who can always cheer me up when I'm down	☐ 1	☐ 2	☐ 3	☐ 4	☐ 5	☐ 6	☐ 7
**7.** I often feel very lonely	☐ 1	☐ 2	☐ 3	☐ 4	☐ 5	☐ 6	☐ 7
**8.** I enjoy the time I spend with the people who are important to me	☐ 1	☐ 2	☐ 3	☐ 4	☐ 5	☐ 6	☐ 7
**9.** When something's on my mind, just talking with the people I know can make me feel better	☐ 1	☐ 2	☐ 3	☐ 4	☐ 5	☐ 6	☐ 7
**10.** When I need someone to help me out, I can usually find someone	☐ 1	☐ 2	☐ 3	☐ 4	☐ 5	☐ 6	☐ 7

Q35 How much do you feel that...

Please mark **one** *response for each item*	**Not at all**	**Little**	**Some**	**Quite a bit**	**Very much**	**Don't know**
**1.** Adults care about you?	☐	☐	☐	☐	☐	☐
**2.** School / work people care about you?	☐	☐	☐	☐	☐	☐
**3.** Your parents care about you?	☐	☐	☐	☐	☐	☐
**4.** Your friends care about you?	☐	☐	☐	☐	☐	☐
**5.** Church leaders care about you?	☐	☐	☐	☐	☐	☐
**6.** You get upset at home?	☐	☐	☐	☐	☐	☐
**7.** Your family cares about your feelings?	☐	☐	☐	☐	☐	☐
**8.** People in your family understand you?	☐	☐	☐	☐	☐	☐
**9.** You want to leave home?	☐	☐	☐	☐	☐	☐
**10.** You and your family have lots of fun together?	☐	☐	☐	☐	☐	☐
**11.** Your family pays a lot of attention to you?	☐	☐	☐	☐	☐	☐

Q36 Please read the following statements and choose the answer that best describes the way your parents (or step-parents or foster parents) in general acted towards you during the past 6 months:

My parents (or step-parents or foster parents) …….

Please mark **one** *response for each item*	**Never**	**Sometimes**	**Often**	**Very often**
**1.** Smile at me	☐	☐	☐	☐
**2.** Soon forget a rule they have made	☐	☐	☐	☐
**3.** Praise me	☐	☐	☐	☐
**4.** Nag me about little things	☐	☐	☐	☐
**5.** Only keep rules when it suits them	☐	☐	☐	☐
**6.** Make sure I know I am appreciated	☐	☐	☐	☐
**7.** Threaten punishment more often than they use it	☐	☐	☐	☐
**8.** Speak of the good things I do	☐	☐	☐	☐
**9.** Enforce a rule or do not enforce a rule depending upon their mood	☐	☐	☐	☐
**10.** Hit me or threaten to do so	☐	☐	☐	☐
**11.** Seem proud of the things I do	☐	☐	☐	☐

SECTION 3 Risk Taking Behaviours - Smoking - Alcohol

We have much to learn about the actual attitudes, knowledge and experiences of young people. So, your honest responses to the questions in this section will provide valuable information on this important topic. If there is a question you'd prefer not to answer, please skip it, rather than give a false answer. All your answers are CONFIDENTIAL:

Q37 Have you ever smoked even part of a cigarette?

- ☐ No → **Go to Q40**
- ☐ Yes, just a few puffs
- ☐ Yes, I have smoked fewer than 10 cigarettes in my life
- ☐ Yes, I have smoked more than 10 cigarettes in my life

Q38 Have you smoked cigarettes in the past 12 months?

- ☐ No → **Go to Q40**
- ☐ Yes

Q39 Have you smoked cigarettes in the past 4 weeks?

- ☐ No
- ☐ Yes

Q40 Have you ever had even part of an alcoholic drink?

- ☐ No → **Go to Q44**
- ☐ Yes, just a few sips
- ☐ Yes, I have had fewer than 10 alcoholic drinks in my life
- ☐ Yes, I have had more than 10 alcoholic drinks in my life

Q41 Have you ever drunk 6 or more alcoholic drinks at one time or drunk so much alcohol that you threw up (vomited)?

- ☐ Never
- ☐ Yes, once only
- ☐ Yes, more than once

Q42 Have you had an alcoholic drink in the past 12 months?

- ☐ No → **Go to Q44**
- ☐ Yes

Q43 Have you been drunk at any time in the past 6 months?

- ☐ No
- ☐ Yes

Q44 How often do you use any of the following drugs for *non-medical* purposes?

Please mark **one** *response for each item*	**Never**	**Over 1 year ago**	**Less than monthly**	**About monthly**	**About weekly**	**Daily**	**Don't know**
**1.** Marijuana (mull, grass, pot)	☐	☐	☐	☐	☐	☐	☐
**2.** Inhalants (glue, petrol)	☐	☐	☐	☐	☐	☐	☐
**3.** Pain killers (panadol etc.)	☐	☐	☐	☐	☐	☐	☐
**4.** Amphetamines (Speed, ice, dexis etc.)	☐	☐	☐	☐	☐	☐	☐
**5.** Party drugs (Ecstasy, acid, nangs/nitrous oxide)	☐	☐	☐	☐	☐	☐	☐
**6.** Something else *(Please specify)* …………………………….	☐	☐	☐	☐	☐	☐	☐

SECTION 4 Friendships and Relationships

Q45 About how many close friends would you say you have?

None	1 – 2	3 – 4	5 or more
☐	☐	☐	☐

Q46 How important are your friends to you in your life?
(*Please mark the* **one** *response that best fits for you)*

Not at all important			Important				Very Important
☐	☐	☐	☐	☐	☐	☐	☐
0	1	2	3	4	5	6	7

Q47 In general how do you feel about your friendships?

Very satisfied	Quite satisfied	Neither satisfied nor dissatisfied	Somewhat dissatisfied	Very dissatisfied
☐	☐	☐	☐	☐

Q48 How supportive is your family to you? (*Please mark only* **one** *response)*

- ☐ Very supportive
- ☐ Supportive
- ☐ Neither supportive nor unsupportive
- ☐ Unsupportive
- ☐ Very unsupportive

Q49 In general, how do you feel about your home life? *(Please mark only* **one** *response)*

- ☐ Very satisfied
- ☐ Quite satisfied
- ☐ Neither satisfied nor dissatisfied
- ☐ Somewhat dissatisfied
- ☐ Very dissatisfied

Q50 What is your current marital status?

- ☐ Single and not in a relationship → **Go to Q59**
- ☐ In a relationship but NOT living together (e.g. boyfriend/girlfriend)
- ☐ In a relationship AND living together (de facto marriage)
- ☐ Married (in a registered marriage) → **Go to Q55**

Q51 How likely are you to...

Please mark ***one*** *response for each item*	Very likely	Likely	Not sure	Unlikely	Very unlikely	Prefer not to say
**1.** Marry your current partner?	☐	☐	☐	☐	☐	☐
**2.** Marry someone other than your present partner at some time in the future?	☐	☐	☐	☐	☐	☐

If you are in a relationship but not living together please answer the following three questions:

Q52 Is this an ongoing sexual relationship?

- ☐ Yes
- ☐ No

Q53 Have you and/or your partner made a definite decision not to live together (at least for the time being)?

- ☐ No, no definite decision made
- ☐ Yes, result of a definite decision

Q54 Whose decision was it to live apart?

- ☐ Yours
- ☐ Your partners
- ☐ Joint decision

Q55 Is your primary partner male or female?

☐ Male ☐ Female

Q56 How old is your partner? ☐☐ years

Q57 Do you know your partner's date of birth?

- ☐ No → **Go to Section 5**
- ☐ Yes

↓

Q58 Partner's date of birth *(Please leave blank if you don't know it)*

☐☐ / ☐☐ / ☐☐☐☐

SECTION 5 Sexual Feelings and Experiences

Q59 Which of these statements best describes your sexual feelings at the moment?
(Please mark only **one** *response)*

- ☐ I am attracted only to people of the opposite sex
- ☐ I am attracted to people of both sexes
- ☐ I am attracted only to people of my own sex
- ☐ Not sure

Q60 Have you ever been diagnosed with a sexually transmissible infection (STI)?

- ☐ No → **Go to Q62**
- ☐ Yes

Q61 Which STI (s) have you been diagnosed with?
(Please mark **any** *that apply to you)*

- ☐ Candaisis/Thrush
- ☐ Chlamydia
- ☐ Genital herpes
- ☐ Genital warts
- ☐ Gonorrhoea
- ☐ Hepatitis B
- ☐ HIV/AIDS
- ☐ Pubic lice (crabs)
- ☐ Syphillis
- ☐ Other

Q62 Have you, and how old were you when you first had an experience of...

*Please mark **one** response for each item*	**Haven't yet**	**Under 13 years**	**13 years**	**14 years**	**15 years**	**16 years**	**17 years**
**1.** Deep kissing?	☐	☐	☐	☐	☐	☐	☐
**2.** Touching a partner's genitals with your hands?	☐	☐	☐	☐	☐	☐	☐
**3.** Being touched on your genitals by a partner's hand?	☐	☐	☐	☐	☐	☐	☐
**4.** Giving oral sex?	☐	☐	☐	☐	☐	☐	☐
**5.** Receiving oral sex?	☐	☐	☐	☐	☐	☐	☐
**6.** Intercourse without a condom?	☐	☐	☐	☐	☐	☐	☐
**7.** Intercourse with a condom?	☐	☐	☐	☐	☐	☐	☐

Q63 Over the last year, with how many people have you had intercourse?

- ☐ I have not had intercourse in the past year
- ☐ 1 person
- ☐ 2 people
- ☐ 3 people
- ☐ 4 people
- ☐ 5 to 10 people
- ☐ 11 or more people

Q64 The last time you had sex, which of the following did you (or your partner) use to stop pregnancy? *(Please mark* **any** *responses that apply)*

- ☐ Nothing
- ☐ Condoms
- ☐ Oral contraceptive (the Pill)
- ☐ Depo Provera (injection)
- ☐ Implanon (implant)
- ☐ IUD
- ☐ Morning after pill
- ☐ Diaphragm or cap
- ☐ Withdrawal (pulling out)
- ☐ Other (*Please specify*) ……………………………………………….

Q65 What have you (or your partner) used in the past to stop pregnancy?
(Please mark **any** *responses that apply)*

- ☐ Nothing
- ☐ Condoms
- ☐ Oral contraceptive (the Pill)
- ☐ Depo Provera (injection)
- ☐ Implanon (implant)
- ☐ IUD
- ☐ Morning after pill
- ☐ Diaphragm or cap
- ☐ Withdrawal (pulling out)
- ☐ Other (*Please specify*) ……………………………………………….

THANK YOU

WE APPRECIATE THE TIME THAT YOU HAVE SPENT COMPLETING THIS QUESTIONNAIRE

ID ☐☐☐☐☐

OFFICE USE ONLY

RA-CH RA-CO RA1-E RA2-E

ID

GROWING UP HEALTHY STUDY

Participant Confidential Questionnaire

MALE

16-18

The purpose of this teenager questionnaire is to obtain information about a range of topics including your relationships at home, school and work, your self-confidence and perceptions of care and support that you receive from others, your health and recent medical history, and your knowledge around and participation in risk taking behaviours such as smoking, drinking and sexual activity

Please read each question carefully and answer all of the questions.
Write your answers clearly in the space provided or mark the most appropriate response

☒

Please take your time in answering all of the questions

If you are uncomfortable about a question or unsure of an answer, please leave it blank or ask one of the Growing Up Healthy Study team for assistance

Please complete this questionnaire independently (without discussing it with anyone)

Remember all answers are STRICTLY confidential

Questionnaire

Today's date: ☐☐ / ☐☐ / ☐☐☐☐

Are you **MALE**?

☐ Yes
☐ No (Please ask one of the Growing Up Healthy Study team for assistance)

SECTION 1 Eating Habits, Weight, Physical Health and Development

Q1 How often do you eat the following foods?

Please mark **one** *response for each item*	**6 + times a week**	**3-5 times a week**	**1-2 times a week**	**1-2 times a month**	**Rarely or never**
**1.** Fried food with a batter or breadcrumb coating	☐	☐	☐	☐	☐
**2.** Gravy, creamy sauces or cheese sauces	☐	☐	☐	☐	☐
**3.** Vegetables, rice or pasta with added butter, margarine, oil or sour cream	☐	☐	☐	☐	☐
**4.** Vegetables that are fried or roasted with fat or oil (don't count oil sprays)	☐	☐	☐	☐	☐
**5.** Sausages, polony, salami, meat pies, pasties, hamburger or bacon	☐	☐	☐	☐	☐
**6.** Hot potato chips or French fries	☐	☐	☐	☐	☐
**7.** Pastries, cakes, sweet biscuits or croissants	☐	☐	☐	☐	☐
**8.** Chocolate, chocolate biscuits or sweet snack bars	☐	☐	☐	☐	☐
**9.** Potato crisps, corn chips, cheezels, twisties or nuts	☐	☐	☐	☐	☐
**10.** Ice cream (any variety)	☐	☐	☐	☐	☐
**11.** Cream or sour cream	☐	☐	☐	☐	☐
**12.** Cheddar, edam or other hard cheese, cream cheese or soft cheeses such as camembert or brie (but excluding ricotta or cottage cheese)	☐	☐	☐	☐	☐

Q2 How much of the following do you usually eat? (*Please mark* **one** *response for each item)*

a Fat on meat?

- ☐ Most or all
- ☐ Some
- ☐ None
- ☐ Don't eat meat

b Skin on chicken?

- ☐ Most or all
- ☐ Some
- ☐ None
- ☐ Don't eat chicken

Q3 How often do you eat the following foods?

Please mark **one** *response for each item*	**6 + times a week**	**3-5 times a week**	**1-2 times a week**	**1-2 times a month**	**Rarely or never**
**Fruit**, including fresh and canned fruit (do not include dried fruit, fruit juices, fruit drinks, fruit bars or frozen fruit desserts)	☐	☐	☐	☐	☐
**Vegetables**, including all forms of vegetables, e.g. fresh, frozen, canned and salads	☐	☐	☐	☐	☐
**Fish**	☐	☐	☐	☐	☐

Q4 How many days per week do you usually have something for breakfast?

Rarely or never	1-2 days per week	3-4 days per week	5-6 days per week	Everyday
☐	☐	☐	☐	☐

Q5 How many days per week did you eat your evening meal with the family (including at least one adult)?

Rarely or never	1-4 days per week	5-7 days per week	Irregularly
☐	☐	☐	☐

Q6 How often do you eat meals or snacks from fast food chains (e.g. MacDonalds, Hungry Jacks, Pizza Hut, Red Rooster, River Rooster, Kentucky Fried)?

Never	Once per fortnight or less	Once per week	2-4 times per week	5-7 time per week
☐	☐	☐	☐	☐

Q7 Did you eat special foods or have a special diet over the past 12 months?

☐ No → **Go to Q9**
☐ Yes

Q8 Why were you on a special diet? *(Please mark* **all** *responses that apply to you)*

☐ Vegetarian
☐ To avoid milk
☐ For diabetes
☐ For allergy
☐ For asthma
☐ For behaviour
☐ For sport
☐ To lose weight
☐ To gain weight, build muscles
☐ Other reason (*Please specify)*…………………………………

Q9 How many serves of fruit do you usually eat each day?
(1 serve = 1 medium piece or 2 small pieces of fruit or 1 cup of diced pieces)

Rarely eat fruit	1 serve or less per day	2 serves per day	3 serves per day	4 serves or more per day
☐	☐	☐	☐	☐

Q10 How many serves of vegetables do you usually eat each day?
(1 serve =1/2 cup cooked vegetables or 1 cup of salad vegetables)

Rarely eat vegetables	1 serves or less per day	2 serves per day	3 serves per day	4 serves per day	5 serves per day	6 serves per day
☐	☐	☐	☐	☐	☐	☐

Q11 Here we are asking for some additional information on how often and how much of the following drinks you usually consume.

When answering these questions, please mark how often you have the drink and write the total number of glasses, cans, or cups you would usually drink (see example). To assist you, below each type of drink is the type of measurement.

	Never	less than once/ month	**1** day/ month	**2** days/ month	**3** days/ month	**1** day/ week	**2** days/ week	**3** days/ week	**4** days/ week	**5** days/ week	**6** days/ week	every day	**Total number of glasses/ cups/cans you usually drink**
i.e. **Water (250 ml glass)**	☐	☐	☐	☐	☐	☐	☐	☐	☐	☐	☒	☐	**8**
**1.** Water (250 ml glass)	☐	☐	☐	☐	☐	☐	☐	☐	☐	☐	☐	☐	
**2.** Fizzy drink (e.g. cola, lemonade) (can, glass)	☐	☐	☐	☐	☐	☐	☐	☐	☐	☐	☐	☐	
**3.**Diet fizzy drink (e.g. diet cola, diet lemonade) (can, glass)	☐	☐	☐	☐	☐	☐	☐	☐	☐	☐	☐	☐	
**4.** Energy drink (e.g. Redbull, V, Monster) (can)	☐	☐	☐	☐	☐	☐	☐	☐	☐	☐	☐	☐	
**5.** Diet energy drink (can)	☐	☐	☐	☐	☐	☐	☐	☐	☐	☐	☐	☐	
**6.** Tea (cup)	☐	☐	☐	☐	☐	☐	☐	☐	☐	☐	☐	☐	
**7.** Herbal tea (cup)	☐	☐	☐	☐	☐	☐	☐	☐	☐	☐	☐	☐	
**8.** Green tea (cup)	☐	☐	☐	☐	☐	☐	☐	☐	☐	☐	☐	☐	
**9.** Instant coffee (cup)	☐	☐	☐	☐	☐	☐	☐	☐	☐	☐	☐	☐	
**10.** Ground coffee (e.g. filter coffee, cappuccino, flat white) (cup, mug)	☐	☐	☐	☐	☐	☐	☐	☐	☐	☐	☐	☐	

OFFICE USE ONLY
Q11
1☐☐ 2☐☐ 3☐☐ 4☐☐ 5☐☐ 6☐☐ 7☐☐ 8☐☐ 9☐☐ 10☐☐

Q12 Do you know how much you weigh?

☐ No → **Go to Q14**
☐ Yes

↓

Q13 What is your current weight? ☐☐☐ . ☐ kg

Please mark only **one** *response for each question below*

Q14 Are you worried about your weight?

**No, not at all**	**A little**	**Moderately**	**Very**
☐	☐	☐	☐

Q15 Do you consider yourself to be?

**Underweight**	**Normal weight**	**A bit overweight**	**Very overweight**
☐	☐	☐	☐

Q16 How often do you weigh yourself?

**Never**	**Once in a while**	**Often**	**Nearly every day**
☐	☐	☐	☐

Q17 **Over the last two weeks…**

1. Have you been trying hard to eat less to change your shape or weight (even if you haven't managed to do so)?

**Not at all**	**Some of the time**	**A lot of the time**	**Most of the time**
☐	☐	☐	☐

2. Have you gone for long periods of time (8 hours or more) without eating anything to try and change your shape or weight?

**Not at all**	**Some of the time**	**A lot of the time**	**Most of the time**
☐	☐	☐	☐

3. Have you tried not to eat certain foods (like chocolate or chips to try to change your shape or weight (even if you haven't been able to do so)?

**Not at all**	**Some of the time**	**A lot of the time**	**Most of the time**
☐	☐	☐	☐

4. Have you tried to stick to any definite rules about diet or eating (e.g., sticking to a calorie limit, a set amount of food or rules about what or when you should eat (even if you haven't been able to do so)?

**Not at all**	**Some of the time**	**A lot of the time**	**Most of the time**
☐	☐	☐	☐

5. Have you been thinking about food or calories so much that you've found it hard to concentrate on things you are interested in (e.g., reading, watching TV or following a conversation)?

**Not at all**	**Some of the time**	**A lot of the time**	**Most of the time**
☐	☐	☐	☐

6. Have there been times when you feel that you have eaten an unusually large amount of food (more than what most people would eat in the same situation)?

**Not at all**	**Some of the time**	**A lot of the time**	**Most of the time**
☐	☐	☐	☐

7. Have you been afraid of losing control over your eating?

**Not at all**	**Some of the time**	**A lot of the time**	**Most of the time**
☐	☐	☐	☐

8. Have you felt that you couldn't control what or how much you were eating?

**Not at all**	**Some of the time**	**A lot of the time**	**Most of the time**
☐	☐	☐	☐

9. Have you felt that you couldn't stop eating once you had started?

**Not at all**	**Some of the time**	**A lot of the time**	**Most of the time**
☐	☐	☐	☐

10. Have you felt guilty after eating?

**Not at all**	**Some of the time**	**A lot of the time**	**Most of the time**
☐	☐	☐	☐

11. Have you eaten in secret because you are embarrassed by how much you eat?

**Not at all**	**Some of the time**	**A lot of the time**	**Most of the time**
☐	☐	☐	☐

12. Have you been afraid that you might gain weight or become fat?

**Not at all**	**Some of the time**	**A lot of the time**	**Most of the time**
☐	☐	☐	☐

13. Have you felt fat?

**Not at all**	**Some of the time**	**A lot of the time**	**Most of the time**
☐	☐	☐	☐

14. Have you had a strong desire to lose weight?

**Not at all**	**Some of the time**	**A lot of the time**	**Most of the time**
☐	☐	☐	☐

15. Have you made yourself sick (vomit) after eating to control your weight?

**Not at all**	**Some of the time**	**A lot of the time**	**Most of the time**
☐	☐	☐	☐

16. Have you taken any pills (like laxatives, water pills, diet tubes) to try to control your weight?

**Not at all**	**Some of the time**	**A lot of the time**	**Most of the time**
☐	☐	☐	☐

17. Have you exercised hard to control your weight?

**Not at all**	**Some of the time**	**A lot of the time**	**Most of the time**
☐	☐	☐	☐

People have different ideas about what sort of things are important to them in how they think about themselves. For some people doing well at school is very important to them, for others, how they are getting on with friends is very important. We're now going to ask you to think about how important weight and shape is to you:

18. Has your weight been important in how you think of yourself as a person?

**Not at all**	**Some of the time**	**A lot of the time**	**Most of the time**
☐	☐	☐	☐

19. Has your shape been important in how you think of yourself as a person?

**Not at all**	**Some of the time**	**A lot of the time**	**Most of the time**
☐	☐	☐	☐

Q18 Please look at the figures and select from the list of numbers and letters A and B provided:

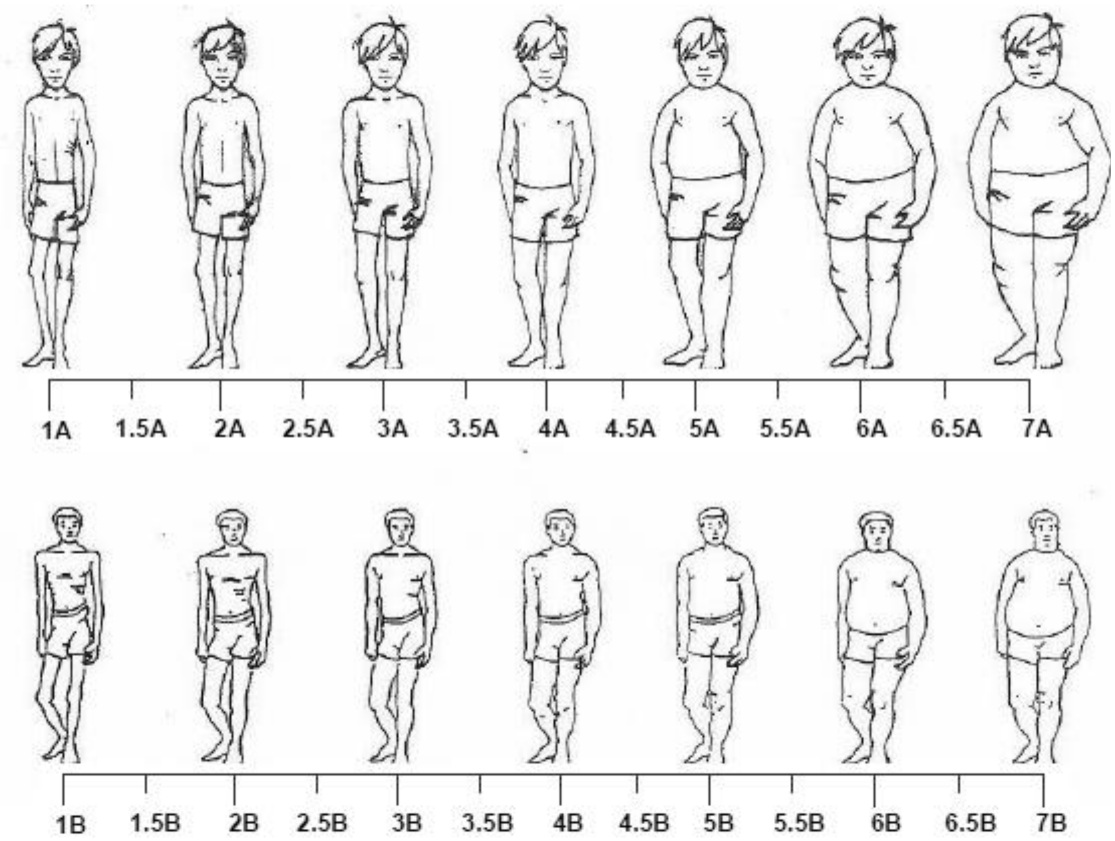

	Number	***Letter***
Which figure best represents what you currently look like?	☐	☐
Which figure would you most like to look like?	☐	☐

The drawings on this page show different amounts of male pubic hair and stages of development of the testes, scrotum and penis. A boy passes through each of the four stages shown by these drawings.

19 Please look at each drawing then choose the one closest to your stage of development by placing an X in the corresponding box.

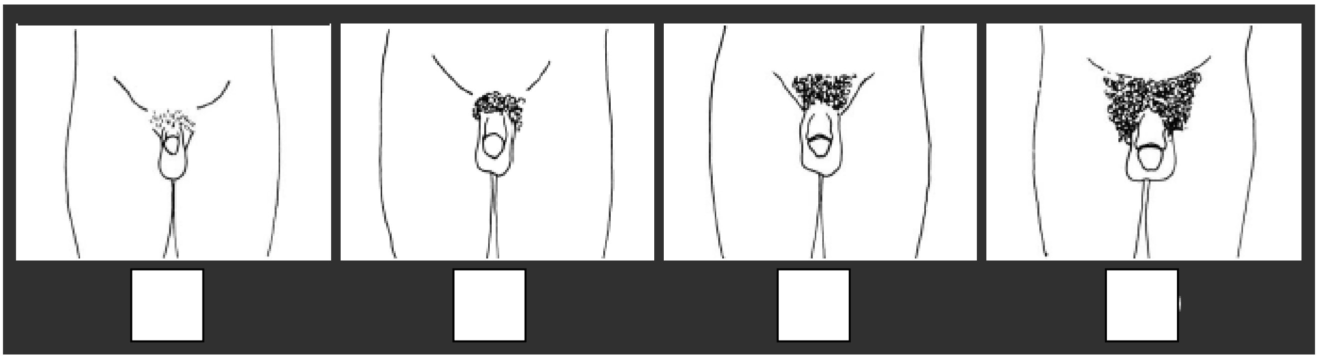

Q20 Below is a list of items that describes adolescents. For each item please mark the box that best represents whether the statement is true, somewhat/sometimes true or very true/often true for you now or within the past 6 months. Please answer all items as well as you can, even if some do not seem to apply to you:

Please mark **one** *response for each item*	**Not true**	**Somewhat/ sometimes true**	**Very true/ often true**
**1.** I act too young for my age	☐	☐	☐
**2.** I have an allergy	☐	☐	☐
**3.** I argue a lot	☐	☐	☐
**4.** I have asthma	☐	☐	☐
**5.** I like the opposite sex	☐	☐	☐
**6.** I like animals	☐	☐	☐
**7.** I brag	☐	☐	☐
**8.** I have trouble concentrating or paying attention	☐	☐	☐
**9.** I can't get my mind off certain thoughts	☐	☐	☐
**10.** I have trouble sitting still	☐	☐	☐
**11.** I am too dependent on adults	☐	☐	☐
**12.** I feel lonely	☐	☐	☐
**13.** I feel confused or in a fog	☐	☐	☐
**14.** I cry a lot	☐	☐	☐
**15.** I am pretty honest	☐	☐	☐
**16.** I am mean to others	☐	☐	☐
**17.** I day dream a lot	☐	☐	☐
**18.** I deliberately try to hurt or kill myself	☐	☐	☐
**19.** I try to get a lot of attention	☐	☐	☐
**20.** I destroy my own things	☐	☐	☐
**21.** I destroy things belonging to others	☐	☐	☐
**22.** I disobey my parents	☐	☐	☐
**23.** I disobey at school	☐	☐	☐
**24.** I don't eat as well as I should	☐	☐	☐
**25.** I don't get along with other kids	☐	☐	☐
**26.** I don't feel guilty after doing something I shouldn't	☐	☐	☐
**27.** I am jealous of others	☐	☐	☐
**28.** I am willing to help others when they need help	☐	☐	☐
**29.** I am afraid of certain animals, situations, or places other than school	☐	☐	☐
**30.** I am afraid of going to school	☐	☐	☐

Please mark **one** *response for each item*	**Not true**	**Somewhat/ sometimes true**	**Very true/ often true**
**31.** I am afraid I might think or do something bad	☐	☐	☐
**32.** I feel that I have to be perfect	☐	☐	☐
**33.** I feel that no one loves me	☐	☐	☐
**34.** I feel that others are out to get me	☐	☐	☐
**35.** I feel worthless or inferior	☐	☐	☐
**36.** I accidentally get hurt a lot	☐	☐	☐
**37.** I get in many fights	☐	☐	☐
**38.** I get teased a lot	☐	☐	☐
**39.** I hang around with kids who get in trouble	☐	☐	☐
**40.** I hear sounds or voices that other people think aren't there	☐	☐	☐
**41.** I act without stopping to think	☐	☐	☐
**42.** I would rather be alone than with others	☐	☐	☐
**43.** I lie or cheat	☐	☐	☐
**44.** I bite my fingernails	☐	☐	☐
**45.** I am nervous or tense	☐	☐	☐
**46.** Parts of my body twitch or make nervous movements	☐	☐	☐
**47.** I have nightmares	☐	☐	☐
**48.** I am not liked by other kids	☐	☐	☐
**49.** I can do certain things better than most kids	☐	☐	☐
**50.** I am too fearful or anxious	☐	☐	☐
**51.** I feel dizzy	☐	☐	☐
**52.** I feel too guilty	☐	☐	☐
**53.** I eat too much	☐	☐	☐
**54.** I feel overtired	☐	☐	☐
**55.** I am overweight	☐	☐	☐
**56.** Physical problems without known medical cause: *a.* Aches or pains (not headaches)	☐	☐	☐
b. Headaches	☐	☐	☐
c. Nausea, feel sick	☐	☐	☐
d. Problems with eyes	☐	☐	☐
e. Rashes or other skin problems	☐	☐	☐
f. Stomach-aches or cramps	☐	☐	☐
g. Vomiting, throwing up	☐	☐	☐
h. Other *(please describe)*…………………………..	☐	☐	☐

Please mark **one** *response for each item*	**Not true**	**Somewhat/ sometimes true**	**Very true/ often true**
**57.** I physically attack people	☐	☐	☐
**58.** I pick my skin or other parts of my body	☐	☐	☐
**59.** I can be pretty friendly	☐	☐	☐
**60.** I like to try new things	☐	☐	☐
**61.** My school work is poor	☐	☐	☐
**62.** I am poorly coordinated or clumsy	☐	☐	☐
**63.** I would rather be with older kids than kids my own age	☐	☐	☐
**64.** I would rather be with younger kids than kids my own age	☐	☐	☐
**65.** I refuse to talk	☐	☐	☐
**66.** I repeat certain actions over and over	☐	☐	☐
**67.** I run away from home	☐	☐	☐
**68.** I scream a lot	☐	☐	☐
**69.** I am secretive or keep things to myself	☐	☐	☐
**70.** I see things that other people think aren’t there	☐	☐	☐
**71.** I am self-conscious or easily embarrassed	☐	☐	☐
**72.** I set fires	☐	☐	☐
**73.** I can work well with my hands	☐	☐	☐
**74.** I show off or clown	☐	☐	☐
**75.** I am shy	☐	☐	☐
**76.** I sleep less than most kids	☐	☐	☐
**77.** I sleep more than most kids during the day and/or night	☐	☐	☐
**78.** I have a good imagination	☐	☐	☐
**79.** I have a speech problem	☐	☐	☐
**80.** I stand up for my rights	☐	☐	☐
**81.** I steal at home	☐	☐	☐
**82.** I steal from places other than home	☐	☐	☐
**83.** I store things up I don’t need	☐	☐	☐
**84.** I do things other people think are strange	☐	☐	☐
**85.** I have thoughts that other people would think are strange	☐	☐	☐
**86.** I am stubborn	☐	☐	☐
**87.** My moods or feelings change suddenly	☐	☐	☐
**88.** I enjoy being with other people	☐	☐	☐
**89.** I am suspicious	☐	☐	☐
**90.** I swear or use dirty language	☐	☐	☐

Please mark **one** *response for each item*	Not true	Somewhat/ sometimes true	Very true/ often true
**91.** I think about killing myself	☐	☐	☐
**92.** I like to make others laugh	☐	☐	☐
**93.** I talk too much	☐	☐	☐
**94.** I tease others a lot	☐	☐	☐
**95.** I have a hot temper	☐	☐	☐
**96.** I think about sex too much	☐	☐	☐
**97.** I threaten to hurt people	☐	☐	☐
**98.** I like to help others	☐	☐	☐
**99.** I am too concerned about being neat or clean	☐	☐	☐
**100.** I have trouble sleeping	☐	☐	☐
**101.** I skip classes or wag school	☐	☐	☐
**102.** I don't have much energy	☐	☐	☐
**103.** I am unhappy, sad or depressed	☐	☐	☐
**104.** I am louder than other kids	☐	☐	☐
**105.** I use alcohol or drugs for nonmedical purposes	☐	☐	☐
**106.** I try to be fair to others	☐	☐	☐
**107.** I enjoy a good joke	☐	☐	☐
**108.** I like to take life easy	☐	☐	☐
**109.** I try to help other people when I can	☐	☐	☐
**110.** I wish I were of the opposite sex	☐	☐	☐
**111.** I keep from getting involved with others	☐	☐	☐
**112.** I worry a lot	☐	☐	☐

SECTION 2 Bullying, Mental Health and Development

The next questions are about bullying at school: Bullying is when someone is picked on by another person, or a group of people say nasty and unpleasant things to him or her. It is also when someone is hit, kicked, threatened, sent nasty notes, when no one talks to them and things like that. *Please mark* **one** *response for each item:*

Q21 Have you ever been bullied at school/TAFE/Uni or at work?

☐ No → **Go to Q25**
☐ Yes

↓

Q22 Has this happened at your current school/TAFE/Uni or workplace?

☐ No
☐ Yes

Q23 Was this in the past three months?

☐ No
☐ Yes

Q24 How did you feel about being bullied? *(Please mark* **all** *responses that apply to you)*

☐ Made you sad
☐ Made you angry
☐ Doesn't bother you
☐ Stressed you out
☐ Other feelings (*Please specify*) ……………………………………

Q25 Have you ever bullied other kids?

☐ No
☐ Yes

Q26 Are you satisfied with the way your school/TAFE/Uni workplace handles bullying?

☐ Very satisfied
☐ Fairly satisfied
☐ Unsatisfied (they could do a lot more)
☐ Very unsatisfactory (nothing is done about it)

Q27 Have any of the following things ever happened to you?

Please mark ***one*** *response for each item*	**4 or more times**	**2 or 3 times**	**Once**	**Never**
**1.** You have been treated with less courtesy than other people	☐	☐	☐	☐
**2.** You have been treated with less respect than other people	☐	☐	☐	☐
**3.** You have received poorer service than other people at restaurants or shops	☐	☐	☐	☐
**4.** People have acted as if they think you are not smart	☐	☐	☐	☐
**5.** People have acted as if they are afraid of you	☐	☐	☐	☐
**6.** People have acted as if they think you are dishonest	☐	☐	☐	☐
**7.** People have acted as if they're better than you are	☐	☐	☐	☐
**8.** You have been called names or insulted	☐	☐	☐	☐
**9.** You have been threatened or harassed	☐	☐	☐	☐
**10.** You have been followed around in shops	☐	☐	☐	☐

→ ***Go to Q29 if you didn't have any of these experiences***

Q28 If you had any of these happen to you what do you think were the main reasons for it?
(Please mark **all** *responses that apply to you)*

- ☐ Your ancestry or national origins
- ☐ Your gender
- ☐ Your race
- ☐ Your age
- ☐ Your religion
- ☐ Your height or weight
- ☐ Your shade of skin colour
- ☐ Your sexual orientation
- ☐ Your education or income level
- ☐ A physical disability
- ☐ The way you look
- ☐ Other (*Please specify*) ……………………………………………

Q29 Below is a list of statements dealing with your general feelings about yourself. Please mark the response for how much you agree or disagree with each statement:

Please mark **one** *response for each item*	**Strongly agree**	**Agree**	**Disagree**	**Strongly disagree**
**1.** On the whole, I am satisfied with myself	☐	☐	☐	☐
**2.** At times, I think I am no good at all	☐	☐	☐	☐
**3.** I feel that I have a number of good qualities	☐	☐	☐	☐
**4.** I am able to do things as well as most other people	☐	☐	☐	☐
**5.** I feel I do not have much to be proud of	☐	☐	☐	☐
**6.** I certainly feel useless at times	☐	☐	☐	☐
**7.** I feel that I'm a person of worth, at least on an equal level with others	☐	☐	☐	☐
**8.** I wish I could have more respect for myself	☐	☐	☐	☐
**9.** All in all, I am inclined to feel that I am a failure	☐	☐	☐	☐
**10.** I take a positive attitude toward myself	☐	☐	☐	☐

Q30 This question is about how you feel right now. Please read each statement carefully and mark the response that best describes how you feel. There are no right or wrong answers. Don't spend too much time on any one statement. For each statement below, please mark the response which best describes how best you feel right now, at this very moment:

**1.** I feel…	☐ Very calm	☐ Calm	☐ Not calm
**2.** I feel…	☐ Very upset	☐ Upset	☐ Not upset
**3.** I feel…	☐ Very pleasant	☐ Pleasant	☐ Not pleasant
**4.** I feel…	☐ Very nervous	☐ Nervous	☐ Not nervous
**5.** I feel…	☐ Very jittery	☐ Jittery	☐ Not jittery
**6.** I feel…	☐ Very rested	☐ Rested	☐ Not rested
**7.** I feel…	☐ Very scared	☐ Scared	☐ Not scared
**8.** I feel…	☐ Very relaxed	☐ Relaxed	☐ Not relaxed
**9.** I feel…	☐ Very worried	☐ Worried	☐ Not worried
**10.** I feel…	☐ Very satisfied	☐ Satisfied	☐ Not satisfied
**11.** I feel…	☐ Very frightened	☐ Frightened	☐ Mot frightened
**12.** I feel…	☐ Very happy	☐ Happy	☐ Not happy
**13.** I feel…	☐ Very sure	☐ Sure	☐ Not sure
**14.** I feel…	☐ Very good	☐ Good	☐ Not good
**15.** I feel…	☐ Very troubled	☐ Troubled	☐ Not troubled
**16.** I feel…	☐ Very bothered	☐ Bothered	☐ Not bothered
**17.** I feel…	☐ Very nice	☐ Nice	☐ Not nice
**18.** I feel…	☐ Very terrified	☐ Terrified	☐ Not terrified
**19.** I feel…	☐ Very mixed-up	☐ Mixed-up	☐ Not mixed-up
**20.** I feel…	☐ Very cheerful	☐ Cheerful	☐ Not cheerful

Q31 A number of statements which boys and girls use to describe themselves are listed below. Read each statement carefully and decide if it is hardly-ever, or sometimes, or often true for you. There are no right or wrong answers. Don't spend too much time on any one statement. For each statement, mark the response that seems to describe you best. Remember to choose the word which best seems to describe how you usually feel:

Please mark **one** *response for each item*	**Hardly ever**	**Sometimes**	**Often**
**1.** I worry about making mistakes	☐	☐	☐
**2.** I feel like crying	☐	☐	☐
**3.** I feel unhappy	☐	☐	☐
**4.** I have trouble making up my mind	☐	☐	☐
**5.** It is difficult for me to face my problems	☐	☐	☐
**6.** I worry too much	☐	☐	☐
**7.** I get upset at home	☐	☐	☐
**8.** I am shy	☐	☐	☐
**9.** I feel troubled	☐	☐	☐
**10.** Unimportant thoughts run through my mind and bother me	☐	☐	☐
**11.** I worry about school / work	☐	☐	☐
**12.** I have trouble deciding what to do	☐	☐	☐
**13.** I notice my heart beats fast	☐	☐	☐
**14.** I am secretly afraid	☐	☐	☐
**15.** I worry about my parents	☐	☐	☐
**16.** My hands get sweaty	☐	☐	☐
**17.** I worry about things that may happen	☐	☐	☐
**18.** It is hard for me to fall asleep at night	☐	☐	☐
**19.** I get a funny feeling in my stomach	☐	☐	☐
**20.** I worry about what others may think of me	☐	☐	☐

Q32 In some situations we feel sure that we can manage well and make things turn out the way we want; in other situations we feel less sure of managing well and less able to make things turn out the way we want. Please select the response that shows how sure you feel in managing each of the following situations. There are no right or wrong answers - just say what you think would be true for you:

Please mark **one** *response for each item*	**Not at all sure**	**A little sure**	**Some-what sure**	**Quite sure**	**Very sure**
**1.** You meet a person for the first time	☐	☐	☐	☐	☐
**2.** You are in a place you don't know anything about	☐	☐	☐	☐	☐
**3.** You have new work to do at school /Work/TAFE	☐	☐	☐	☐	☐
**4.** You have to get something done and there is a lot of pressure	☐	☐	☐	☐	☐
**5.** You have to work out a problem with a teacher/ lecturer/ employer	☐	☐	☐	☐	☐
**6.** You have to work out a problem with your mother	☐	☐	☐	☐	☐
**7.** You have to give a talk in front of people	☐	☐	☐	☐	☐
**8.** You have to do something for the first time	☐	☐	☐	☐	☐
**9.** You have to travel to a new place by yourself	☐	☐	☐	☐	☐
**10.** You have to work out a problem with a friend	☐	☐	☐	☐	☐
**11.** You have trouble solving a problem in school/work/TAFE	☐	☐	☐	☐	☐
**12.** You feel very unhappy	☐	☐	☐	☐	☐
**13.** You lose something important	☐	☐	☐	☐	☐
**14.** You have to do things people expect you to do	☐	☐	☐	☐	☐
**15.** You have to figure out something by yourself	☐	☐	☐	☐	☐
**16.** You have to make an important decision	☐	☐	☐	☐	☐
**17.** Someone counts on you to do something important	☐	☐	☐	☐	☐
**18.** You are bored and want to find something interesting to do	☐	☐	☐	☐	☐
**19.** Things are going wrong	☐	☐	☐	☐	☐
**20.** You become older	☐	☐	☐	☐	☐
**21.** You have to work out a problem with your father	☐	☐	☐	☐	☐
**22.** You have done something wrong	☐	☐	☐	☐	☐

Q33 Here is a list of things that happen to people and that people think or feel. Please read each statement carefully and thinking over the past two weeks, select the response that best describes how you feel about each statement. There are no right or wrong answers:

Please mark **one** *response for each item*	**Never**	**Sometimes**	**Often**	**Always**
**1.** I think that my life is bad	☐	☐	☐	☐
**2.** I have trouble doing things	☐	☐	☐	☐
**3.** I feel that I am a bad person	☐	☐	☐	☐
**4.** I wish I were dead	☐	☐	☐	☐
**5.** I have trouble sleeping	☐	☐	☐	☐
**6.** I feel no one loves me	☐	☐	☐	☐
**7.** I think bad things happen because of me	☐	☐	☐	☐
**8.** I feel lonely	☐	☐	☐	☐
**9.** My Stomach hurts	☐	☐	☐	☐
**10.** I feel like bad things happen to me	☐	☐	☐	☐
**11.** I feel like I am stupid	☐	☐	☐	☐
**12.** I feel sorry for myself	☐	☐	☐	☐
**13.** I think I do things badly	☐	☐	☐	☐
**14.** I feel bad about what I do	☐	☐	☐	☐
**15.** I hate myself	☐	☐	☐	☐
**16.** I want to be alone	☐	☐	☐	☐
**17.** I feel like crying	☐	☐	☐	☐
**18.** I feel sad	☐	☐	☐	☐
**19.** I feel empty inside	☐	☐	☐	☐
**20.** I think my life will be bad	☐	☐	☐	☐

Q34 The following statements have been used by many people to describe how much support they get from other people. How much do you agree or disagree with each of these statements? The more you agree the higher the number you should mark. The more you disagree, the lower the number you should mark:

Please mark **one** *response for each item*	**Disagree**					**Agree**
**1.** People don't come to visit me as often as I would like	☐1	☐2	☐3	☐4	☐5	☐6	☐7
**2.** I often need help from other people but can't get it	☐1	☐2	☐3	☐4	☐5	☐6	☐7
**3.** I seem to have a lot of friends	☐1	☐2	☐3	☐4	☐5	☐6	☐7
**4.** I don't have anyone that I can confide in	☐1	☐2	☐3	☐4	☐5	☐6	☐7
**5.** I have no one to lean on in times of trouble	☐1	☐2	☐3	☐4	☐5	☐6	☐7
**6.** There is someone who can always cheer me up when I'm down	☐1	☐2	☐3	☐4	☐5	☐6	☐7
**7.** I often feel very lonely	☐1	☐2	☐3	☐4	☐5	☐6	☐7
**8.** I enjoy the time I spend with the people who are important to me	☐1	☐2	☐3	☐4	☐5	☐6	☐7
**9.** When something's on my mind, just talking with the people I know can make me feel better	☐1	☐2	☐3	☐4	☐5	☐6	☐7
**10.** When I need someone to help me out, I can usually find someone	☐1	☐2	☐3	☐4	☐5	☐6	☐7

Q35 How much do you feel that...

Please mark **one** *response for each item*	**Not at all**	**Little**	**Some**	**Quite a bit**	**Very much**	**Don't know**
**1.** Adults care about you?	☐	☐	☐	☐	☐	☐
**2.** School / work people care about you?	☐	☐	☐	☐	☐	☐
**3.** Your parents care about you?	☐	☐	☐	☐	☐	☐
**4.** Your friends care about you?	☐	☐	☐	☐	☐	☐
**5.** Church leaders care about you?	☐	☐	☐	☐	☐	☐
**6.** You get upset at home?	☐	☐	☐	☐	☐	☐
**7.** Your family cares about your feelings?	☐	☐	☐	☐	☐	☐
**8.** People in your family understand you?	☐	☐	☐	☐	☐	☐
**9.** You want to leave home?	☐	☐	☐	☐	☐	☐
**10.** You and your family have lots of fun together?	☐	☐	☐	☐	☐	☐
**11.** Your family pays a lot of attention to you?	☐	☐	☐	☐	☐	☐

Q36 Please read the following statements and choose the answer that best describes the way your parents (or step-parents or foster parents) in general acted towards you during the past 6 months:

My parents (or step-parents or foster parents) …….

Please mark **one** *response for each item*	**Never**	**Sometimes**	**Often**	**Very often**
**1.** Smile at me	☐	☐	☐	☐
**2.** Soon forget a rule they have made	☐	☐	☐	☐
**3.** Praise me	☐	☐	☐	☐
**4.** Nag me about little things	☐	☐	☐	☐
**5.** Only keep rules when it suits them	☐	☐	☐	☐
**6.** Make sure I know I am appreciated	☐	☐	☐	☐
**7.** Threaten punishment more often than they use it	☐	☐	☐	☐
**8.** Speak of the good things I do	☐	☐	☐	☐
**9.** Enforce a rule or do not enforce a rule depending upon their mood	☐	☐	☐	☐
**10.** Hit me or threaten to do so	☐	☐	☐	☐
**11.** Seem proud of the things I do	☐	☐	☐	☐

SECTION 3 Risk Taking Behaviours - Smoking - Alcohol

We have much to learn about the actual attitudes, knowledge and experiences of young people. So, your honest responses to the questions in this section will provide valuable information on this important topic. If there is a question you'd prefer not to answer, please skip it, rather than give a false answer. All your answers are CONFIDENTIAL:

Q37 Have you ever smoked even part of a cigarette?

- ☐ No → **Go to Q40**
- ☐ Yes, just a few puffs
- ☐ Yes, I have smoked fewer than 10 cigarettes in my life
- ☐ Yes, I have smoked more than 10 cigarettes in my life

Q38 Have you smoked cigarettes in the past 12 months?

- ☐ No → **Go to Q40**
- ☐ Yes

Q39 Have you smoked cigarettes in the past 4 weeks?

- ☐ No
- ☐ Yes

Q40 Have you ever had even part of an alcoholic drink?

- ☐ No → **Go to Q44**
- ☐ Yes, just a few sips
- ☐ Yes, I have had fewer than 10 alcoholic drinks in my life
- ☐ Yes, I have had more than 10 alcoholic drinks in my life

Q41 Have you ever drunk 6 or more alcoholic drinks at one time or drunk so much alcohol that you threw up (vomited)?

- ☐ Never
- ☐ Yes, once only
- ☐ Yes, more than once

Q42 Have you had an alcoholic drink in the past 12 months?

- ☐ No → **Go to Q44**
- ☐ Yes

Q43 Have you been drunk at any time in the past 6 months?

- ☐ No
- ☐ Yes

Q44 How often do you use any of the following drugs for *non-medical* purposes?

Please mark **one** *response for each item*	**Never**	**Over 1 year ago**	**Less than monthly**	**About monthly**	**About weekly**	**Daily**	**Don't know**
**1.** Marijuana (mull, grass, pot)	☐	☐	☐	☐	☐	☐	☐
**2.** Inhalants (glue, petrol)	☐	☐	☐	☐	☐	☐	☐
**3.** Pain killers (panadol etc.)	☐	☐	☐	☐	☐	☐	☐
**4.** Amphetamines (Speed, ice, dexis etc.)	☐	☐	☐	☐	☐	☐	☐
**5.** Party drugs (Ecstasy, acid, nangs/nitrous oxide)	☐	☐	☐	☐	☐	☐	☐
**6.** Something else *(Please specify)* ………………………………	☐	☐	☐	☐	☐	☐	☐

SECTION 4 Friendships and Relationships

Q45 About how many close friends would you say you have?

None	1 – 2	3 – 4	5 or more
☐	☐	☐	☐

Q46 How important are your friends to you in your life?
(*Please mark the* **one** *response that best fits for you)*

Not at all important			Important				Very Important
☐	☐	☐	☐	☐	☐	☐	☐
0	1	2	3	4	5	6	7

Q47 In general how do you feel about your friendships?

Very satisfied	Quite satisfied	Neither satisfied nor dissatisfied	Somewhat dissatisfied	Very dissatisfied
☐	☐	☐	☐	☐

Q48 How supportive is your family to you? (*Please mark only* **one** *response)*

- ☐ Very supportive
- ☐ Supportive
- ☐ Neither supportive nor unsupportive
- ☐ Unsupportive
- ☐ Very unsupportive

Q49 In general, how do you feel about your home life? *(Please mark only* **one** *response)*

- ☐ Very satisfied
- ☐ Quite satisfied
- ☐ Neither satisfied nor dissatisfied
- ☐ Somewhat dissatisfied
- ☐ Very dissatisfied

Q50 What is your current marital status?

- ☐ Single and not in a relationship → **Go to Q59**
- ☐ In a relationship but NOT living together (e.g. boyfriend/girlfriend)
- ☐ In a relationship AND living together (de facto marriage)
- ☐ Married (in a registered marriage) → **Go to Q55**

Q51 How likely are you to...

Please mark ***one*** *response for each item*	Very likely	Likely	Not sure	Unlikely	Very unlikely	Prefer not to say
**1.** Marry your current partner?	☐	☐	☐	☐	☐	☐
**2.** Marry someone other than your present partner at some time in the future?	☐	☐	☐	☐	☐	☐

If you are in a relationship but not living together please answer the following three questions:

Q52 Is this an ongoing sexual relationship?

- [ ] Yes
- [ ] No

Q53 Have you and/or your partner made a definite decision not to live together (at least for the time being)?

- [ ] No, no definite decision made
- [ ] Yes, result of a definite decision

Q54 Whose decision was it to live apart?

- [ ] Yours
- [ ] Your partners
- [ ] Joint decision

Q55 Is your primary partner male or female?

- [ ] Male
- [ ] Female

Q56 How old is your partner? ☐☐ years

Q57 Do you know your partner's date of birth?

- [ ] No → **Go to Section 5**
- [ ] Yes

↓

Q58 Partner's date of birth *(Please leave blank if you don't know it)*

☐☐ / ☐☐ / ☐☐☐☐

SECTION 5 Sexual Feelings and Experiences

Q59 Which of these statements best describes your sexual feelings at the moment?
(Please mark only **one** *response)*

- ☐ I am attracted only to people of the opposite sex
- ☐ I am attracted to people of both sexes
- ☐ I am attracted only to people of my own sex
- ☐ Not sure

Q60 Have you ever been diagnosed with a sexually transmissible infection (STI)?

- ☐ No → **Go to Q62**
- ☐ Yes

Q61 Which STI (s) have you been diagnosed with?
(Please mark **any** *that apply to you)*

- ☐ Candaisis/Thrush
- ☐ Chlamydia
- ☐ Genital herpes
- ☐ Genital warts
- ☐ Gonorrhoea
- ☐ Hepatitis B
- ☐ HIV/AIDS
- ☐ Pubic lice (crabs)
- ☐ Syphillis
- ☐ Other

Q62 Have you, and how old were you when you first had an experience of...

*Please mark **one** response for each item*	**Haven't yet**	**Under 13 years**	**13 years**	**14 years**	**15 years**	**16 years**	**17 years**
**1.** Deep kissing?	☐	☐	☐	☐	☐	☐	☐
**2.** Touching a partner's genitals with your hands?	☐	☐	☐	☐	☐	☐	☐
**3.** Being touched on your genitals by a partner's hand?	☐	☐	☐	☐	☐	☐	☐
**4.** Giving oral sex?	☐	☐	☐	☐	☐	☐	☐
**5.** Receiving oral sex?	☐	☐	☐	☐	☐	☐	☐
**6.** Intercourse <u>without</u> a condom?	☐	☐	☐	☐	☐	☐	☐
**7.** Intercourse <u>with</u> a condom?	☐	☐	☐	☐	☐	☐	☐

Q63 Over the last year, with how many people have you had intercourse?

- ☐ I have not had intercourse in the past year
- ☐ 1 person
- ☐ 2 people
- ☐ 3 people
- ☐ 4 people
- ☐ 5 to 10 people
- ☐ 11 or more people

Q64 The last time you had sex, which of the following did you (or your partner) use to stop pregnancy? *(Please mark* **any** *responses that apply)*

- ☐ Nothing
- ☐ Condoms
- ☐ Oral contraceptive (the Pill)
- ☐ Depo Provera (injection)
- ☐ Implanon (implant)
- ☐ IUD
- ☐ Morning after pill
- ☐ Diaphragm or cap
- ☐ Withdrawal (pulling out)
- ☐ Other (*Please specify*) ……………………………………………………

Q65 What have you (or your partner) used in the past to stop pregnancy?
(Please mark **any** *responses that apply)*

- ☐ Nothing
- ☐ Condoms
- ☐ Oral contraceptive (the Pill)
- ☐ Depo Provera (injection)
- ☐ Implanon (implant)
- ☐ IUD
- ☐ Morning after pill
- ☐ Diaphragm or cap
- ☐ Withdrawal (pulling out)
- ☐ Other (*Please specify*) ……………………………………………………

THAN K YOU

WE APPRECIATE THE TIME THAT YOU HAVE SPENT COMPLETING THIS QUESTIONNAIRE

ID

OFFICE USE ONLY

RA-CH ☐☐ RA-CO ☐☐ RA1-E ☐☐ RA2-E ☐☐

ID ☐☐☐☐☐

GROWING UP HEALTHY STUDY

Participant Medical History Questionnaire

16-18

Thank you for giving your time to fill in this questionnaire

The purpose of this questionnaire is to obtain information about your health and wellbeing

Please read each question carefully and answer all of the questions.
Write your answers clearly in the space provided or mark the most appropriate response

☒

All information will be strictly confidential

Please take your time in answering all of the questions

If you require assistance to answer any of the questions please contact the Study Coordinators:

Blagica & Tina

T: +61 6458 1443
M: 0439 266 434
Email: ***guhstudy-swih@uwa.edu.au***

If you are coming in for an appointment, please bring your completed questionnaire with you on the day.

If you are unable to attend an appointment, please use the Reply Paid envelope enclosed to return your completed questionnaire.

If possible, could you please return your completed questionnaire to us by:

☐☐ / ☐☐ / ☐☐☐☐

Confidential

Q1 Have you ever attended the School Dental Service in Western Australia (this includes dental vans visiting schools)?

☐ No
☐ Yes
☐ Don't know

Q2 In the past 12 months, have you attended any of the following?

☐ No → **Go to Q3**
☐ Yes

Please mark **all** *appropriate responses applicable to you*

	**No**	**Yes** Now completed	**Yes** Still attending regularly/occasionally
GP or family doctor	☐	☐	☐
Accident and emergency	☐	☐	☐
Hospital outpatient (department or clinic)	☐	☐	☐
Private medical specialist	☐	☐	☐
Dentist/Dental therapist/Orthodontist	☐	☐	☐
School nurse	☐	☐	☐
Optician/Optometrist	☐	☐	☐
Dietician/Nutritionist	☐	☐	☐
Physiotherapist	☐	☐	☐
Occupational therapist (OT)	☐	☐	☐
Speech therapist	☐	☐	☐
Psychologist/Psychiatrist	☐	☐	☐
Podiatrist	☐	☐	☐
Chiropractor	☐	☐	☐
Alternative therapist (e.g. iridologist)	☐	☐	☐

Q3 Do you have now, or have you had in the past, any of the following health professional diagnosed medical conditions or health problems?

Please mark **one** *response for each item*	**No**	**Yes, in the past**	**Yes, now**	**Yes, now and in the past**
Acne	☐	☐	☐	☐
Anxiety problems	☐	☐	☐	☐
Arthritis or joint problems	☐	☐	☐	☐
Asthma	☐	☐	☐	☐
Attentional problems	☐	☐	☐	☐
Back pain	☐	☐	☐	☐
Behavioural problems	☐	☐	☐	☐
Bladder control problems	☐	☐	☐	☐
Chronic respiratory or breathing problems (other than asthma)	☐	☐	☐	☐
Coeliac disease	☐	☐	☐	☐
Co-ordination or clumsiness difficulties	☐	☐	☐	☐
Depression	☐	☐	☐	☐
Developmental disorder (e.g. attention deficit disorder, autism, intellectual disability)	☐	☐	☐	☐
Diabetes	☐	☐	☐	☐
Eating disorder/weight problems	☐	☐	☐	☐
Hayfever or some other allergy	☐	☐	☐	☐
Hearing impairment or deafness	☐	☐	☐	☐
Heart condition	☐	☐	☐	☐
Hemochromatosis (iron overload disease)	☐	☐	☐	☐
Intellectual disability	☐	☐	☐	☐
Learning problems	☐	☐	☐	☐
Menstrual problems	☐	☐	☐	☐
Migraine or severe headache	☐	☐	☐	☐
Neck pain	☐	☐	☐	☐
Sleep disturbance	☐	☐	☐	☐
Speech and/or language problems	☐	☐	☐	☐
Thyroid gland problems	☐	☐	☐	☐
Vision problems	☐	☐	☐	☐
Any other medical condition or health problem not mentioned here	☐	☐	☐	☐

Q4 If you have answered "Yes" to any of the health problems in the previous question, or have any other health professional diagnosed problem or condition, please describe the condition or problem in more detail below (e.g. long sighted - wear glasses for reading; diagnosed with attention deficit disorder; asthma requiring medication)

Please list every medical condition/health problem separately - otherwise leave this blank.

**What condition/problem?**	**Who diagnosed it?**	**When was it diagnosed?**	**Treatment**
e.g. Impacted wisdom teeth	*Dentist*	*6 months ago*	*Referral to dental surgeon, antibiotics*

OFFICE USE ONLY
Q4
1
5
9
13

Q5 In the <u>past 6 months</u>, have you taken/used any prescription medication(s)?

☐ No → **Go to Q6**
☐ Yes

Which medication(s)?

Name	Reason for taking it	Are you still taking it?
e.g. Antibiotics	*For acne*	*Yes*
Ventolin	*For asthma*	*Yes*
Cortisone cream	*For eczema*	*No*
The Pill or Depo-Provera	*For acne, menstrual disorders or contraception*	*Yes*

Q6 In the **past 6 months**, have you taken/used any 'over the counter' medication(s) *(including vitamins, minerals and health food products)?*

☐ No → **Go to Q7**
☐ Yes

Which medication(s)?

Name	Reason for taking it	Are you still taking it?
e.g. Neurofen	*For period pain*	*Yes*
Antihistamine	*For hayfever*	*No*
Fish oil capsules	*For ADD*	*Yes*

OFFICE USE ONLY

Q5 1 ... 10 ... 20

Q6 1 ... 10 ... 20

Q7 Have you ever had any accidents or injuries which required you to go to a Doctor (GP), hospital or clinic?

☐ No → **Go to Q8**
☐ Yes

Please describe the accident, the injury and any treatment (e.g. fell off bike, cut arm, 3 stitches), and list every accident/injury separately, giving as much detail as possible.

**Injury**	**How did it happen?**	**When did it happen?**	**Treatment**
e.g. Sprained wrist	*Fell down stairs*	*3 months ago*	*Physiotherapy/bandage*

Q8 Have you ever been admitted to a hospital/day surgery?

☐ No → **Go to Q9**
☐ Yes

Please list each admission separately, giving as much detail as possible.

**Date**	**Which hospital?**	**Reason for admission**
e.g. October 2005	*McCourt St Day Surgery*	*Removal of impacted wisdom teeth*

OFFICE USE ONLY

Q7
1 ☐ ☐☐☐☐☐☐☐☐☐☐
2 ☐ ☐☐☐☐☐☐☐☐☐☐
3 ☐ ☐☐☐☐☐☐☐☐☐☐
4 ☐ ☐☐☐☐☐☐☐☐☐☐
5 ☐ ☐☐☐☐☐☐☐☐☐☐

Q8
1 ☐☐/☐☐/☐☐☐☐ ☐☐☐ ☐☐☐☐☐☐☐☐☐☐
2 ☐☐/☐☐/☐☐☐☐ ☐☐☐ ☐☐☐☐☐☐☐☐☐☐
3 ☐☐/☐☐/☐☐☐☐ ☐☐☐ ☐☐☐☐☐☐☐☐☐☐
4 ☐☐/☐☐/☐☐☐☐ ☐☐☐ ☐☐☐☐☐☐☐☐☐☐
5 ☐☐/☐☐/☐☐☐☐ ☐☐☐ ☐☐☐☐☐☐☐☐☐☐

Q9 This question asks about your biological family's history of coeliac disease and hemochromatosis (iron overload disease) ***and*** whether or not it was diagnosed by a doctor. *(Please include half-brothers and half-sisters but not step-brothers or step-sisters)*

Please mark **all** *applicable responses*	**No**	**Yes**	**Don't Know**	**Diagnosed by a Doctor** **No**	**Diagnosed by a Doctor** **Yes**
Does your mother have…					
Coeliac disease	☐	☐	☐	☐	☐
Hemochromatosis (iron overload)	☐	☐	☐	☐	☐
Does your father have...					
Coeliac disease	☐	☐	☐	☐	☐
Hemochromatosis (iron overload)	☐	☐	☐	☐	☐
Do any of your biological brothers or sisters (siblings) have...					
Coeliac disease	☐	☐	☐	☐	☐
Hemochromatosis (iron overload)	☐	☐	☐	☐	☐

Q10 Please write below any comments concerning this questionnaire, the research, or anything else you would like to tell us about:

Q11 Date questionnaire completed: ☐☐/☐☐/☐☐☐☐

THANK YOU

WE APPRECIATE THE TIME THAT YOU HAVE SPENT COMPLETING THIS QUESTIONNAIRE

ID | | | | | |
