# Supplementary material for: Cohort profile: The Growing Up Healthy Study (GUHS)—A prospective and observational cohort study investigating the long-term health outcomes of offspring conceived after assisted reproductive technologies
Source: PLoS One. 2022 Jul 22;17(7):e0272064. doi: 10.1371/journal.pone.0272064 (PMC9307151; doi:10.1371/journal.pone.0272064)
Supplement: S4 File — (PDF) [file pone.0272064.s005.pdf]

OFFICE USE ONLY

RA-CH ☐☐ RA-CO ☐☐ RA1-E ☐☐ RA2-E ☐☐

ID ☐☐☐☐☐

GROWING UP HEALTHY STUDY

Participant Questionnaire

20-22

Thank you for giving your time to fill in this questionnaire

Please read each question carefully and answer all of the questions.
Write your answers clearly in the space provided or mark the most appropriate response

☒

All information will be strictly confidential

Please take your time in answering all of the questions

If you require assistance to answer any of the questions please contact the Study Coordinators:

Blagica & Tina
T: +61 6458 1443
M: 0439 266 434
Email: ***guhstudy-swih@uwa.edu.au***

Please complete this questionnaire independently
(without discussing it with anyone)

If you are coming in for an appointment, please bring your completed questionnaire with you on the day.

If you are unable to attend an appointment, please use the Reply Paid envelope enclosed to return your completed questionnaire.

If possible, could you please return your completed questionnaire to us by:

☐☐ / ☐☐ / ☐☐☐☐

Questionnaire

The purpose of this questionnaire is to obtain information about what you are doing now and your health and wellbeing.

What is your date of birth?

☐☐ / ☐☐ / ☐☐☐☐

What is your sex?

☐ M ☐ F

SECTION 1 – Housing & Family

Q1 What is your current residential postcode? ☐☐☐☐

Q2 Where do you live?
*Please mark only **one** response*

- ☐ Separate house
- ☐ Semi-detached house/row or terrace house/townhouse etc.
- ☐ Flat/unit/apartment
- ☐ University or college accommodation
- ☐ Boarding house, hostel
- ☐ Caravan/tent/cabin/houseboat
- ☐ Other *(please specify):* ....

Q3 Who do you live with?
*Please mark only **one** response*

- ☐ I live alone
- ☐ My partner
- ☐ My child/children
- ☐ My parent(s)/step-parent(s)
- ☐ Other relatives (e.g. grandparents, aunt etc.)
- ☐ My friend(s)/flatmate(s)
- ☐ Other *(please specify):* ....

Q4 Do you have any children?

- ☐ No → **Go to Q5**
- ☐ Yes

↓

Please list each of your children's first name, sex and date of birth

	First Name	Sex (M/F)	Date of birth
1			☐☐ ☐☐ ☐☐☐☐
2			☐☐ ☐☐ ☐☐☐☐
3			☐☐ ☐☐ ☐☐☐☐
4			☐☐ ☐☐ ☐☐☐☐
5			☐☐ ☐☐ ☐☐☐☐

SECTION 2 – Education

Q5 What is the highest level of education you have completed?

- ☐ Primary school
- ☐ Secondary school (high school)
- ☐ University
- ☐ Other educational institution (e.g. TAFE, college)

Q6 What is the highest year of school you have completed?
Please mark only **one** *response*

- ☐ Year 12 (or equivalent)
- ☐ Year 11 (or equivalent)
- ☐ Year 10 (or equivalent)
- ☐ Year 9 (or equivalent)
- ☐ Other *(please specify):* …………………………………………………..

Q7 Are you currently studying or doing a course?

- ☐ No → **Go to Q9**
- ☐ Yes

Q8 Where are you studying?
Please mark only **one** *response*

- ☐ At school
- ☐ At university
- ☐ At TAFE/College
- ☐ Other *(please specify):* ……………………………………………………

Q9 What are you doing now?
Please mark **all** *responses that apply*

- ☐ Studying full-time
- ☐ Studying part-time
- ☐ An apprenticeship
- ☐ Working full-time
- ☐ Working part-time
- ☐ Looking for work
- ☐ Gap year
- ☐ Carer for my child
- ☐ Carer for a family member
- ☐ Other *(please specify):* ……………………………

OFFICE USE ONLY

Q11 ☐☐☐☐☐☐

SECTION 3 – Occupation, Work and Income

Q10 Do you currently have a full-time or part-time job of any kind?

*Please mark only **one** response*

- ☐ No, do not have a job - not seeking work → **Go to Q14**
- ☐ No, do not have a job - actively seeking work → **Go to Q14**
- ☐ Yes, do work for payment or profit
- ☐ Yes, do unpaid work in a family business
- ☐ Yes, do other unpaid work

Q11 Please note your job title and describe what you do for your job

Job title:
Job Description:

Q12 How many hours per week do you usually work in all jobs?

☐☐☐ hours

Q13 What is the total amount of your usual salary/wage after tax per week (how much money do you take home, including any benefits/assistance)?

*Please mark only **one** response*

- ☐ <$50
- ☐ $50-99 per week
- ☐ $100-199 per week
- ☐ $200-299 per week
- ☐ $300-399 per week
- ☐ $400-499 per week
- ☐ > $500 per week

Q14 Are you receiving any government benefits, pension or allowance?

- ☐ No → **Go to Q16**
- ☐ Yes

Q15 Which government benefits, pension or allowance are you receiving?

*Please mark **all** responses that apply*

- ☐ Baby Bonus
- ☐ Carer Allowance (child)
- ☐ Carer Payment (child)
- ☐ Child Care Benefit
- ☐ Child Care Rebate
- ☐ Family Tax Benefit Part A
- ☐ Family Tax Benefit Part B
- ☐ JET Child Care Fee Assistance
- ☐ Newstart Allowance
- ☐ Parenting Payment
- ☐ Rent Assistance
- ☐ Youth Allowance
- ☐ Assistance for Isolated Children
- ☐ Carer Allowance (adult)
- ☐ Carer Payment (adult)
- ☐ Crisis Payment
- ☐ Disability Support Pension
- ☐ Double Orphan Pension
- ☐ Maternity Immunisation Allowance
- ☐ Mobility Allowance
- ☐ Pensioner Education Supplement
- ☐ Sickness Allowance
- ☐ Youth Disability Supplement
- ☐ Other *(please specify):* ……………………

SECTION 4 – Physical Activity

We are interested in finding out about the kinds of physical activities that people do as part of their everyday lives. These questions are about the time you spent being physically active in the past 7 days.

Please answer each question even if you do not consider yourself to be an active person. Include activities you do at work, as part of your house and yard work, to get from place to place and in your spare time for recreation, exercise or sport:

Think about all the vigorous physical activities that you did in the past 7 days. Vigorous physical activities refer to activities that take hard physical effort and make you breathe much harder than normal. Think only about those physical activities that you did for at least 10 minutes at a time.

Q16 During the last 7 days, on how many days did you do vigorous physical activities like heavy lifting, digging, aerobics or fast cycling?

☐ Days per week or ☐ None → **Go to Q17**

How much time did you usually spend on one of those days doing vigorous physical activities?

☐☐ Hours per day ☐☐ Minutes per day ☐ Don't know/unsure

Think about all the moderate activities that you did in the past 7 days. Moderate physical activities refer to activities that take moderate physical effort and make you breathe somewhat harder than normal. Think only about those physical activities that you did for at least 10 minutes at a time.

Q17 During the past 7 days, on how many days did you do moderate physical activities like carrying light loads, bicycling at a regular pace or doubles tennis? *Do not include walking*.

☐ Days per week or ☐ None → **Go to Q18**

How much time did you usually spend on one of those days doing moderate physical activities?

☐☐ Hours per day ☐☐ Minutes per day ☐ Don't know/unsure

Think about all the time you spent walking in the past 7 days. This includes at work and at home, walking to travel from place to place, and any other walking that you might do solely for recreation, sport, exercise, or leisure.

Q18 During the past 7 days, on how many days did you walk for at least 10 minutes at a time?

☐ Days per week or ☐ None → **Go to Q19**

How much time did you spend walking on one of those days?

☐☐ Hours per day ☐☐ Minutes per day ☐ Don't know/unsure

The last question is about the time you spent sitting on weekdays during the past 7 days. Include time spent at work, at home, while doing course work and during leisure time. This may include time spent sitting at a desk, visiting friends, reading or sitting to watch television.

Q19 During the past 7 days, how much time did you spend sitting on a weekday?

☐☐ Hours per day ☐☐ Minutes per day ☐ Don't know/unsure

SECTION 5 – Dietary Habits

Q20 Here we are asking for information about how often and how much of the following drinks you usually consume.

When answering these questions, please mark how often you have the drink and write the total number of glasses, cans, or cups you would usually drink (see example). To assist you, next to each type of drink is the measurement.

	Never	less than once/ month	**1** day/ month	**2** days/ month	**3** days/ month	**1** day/ week	**2** days/ week	**3** days/ week	**4** days/ week	**5** days/ week	**6** days/ week	every day	**Total number of glasses/ cups/cans you usually drink**
***i.e.* Water (250 ml glass)**	☐	☐	☐	☐	☐	☐	☐	☐	☐	☐	☒	☐	**8**
**1.** Water (250 ml glass)	☐	☐	☐	☐	☐	☐	☐	☐	☐	☐	☐	☐	
**2.** Fizzy drink (e.g. cola, lemonade) (can, glass)	☐	☐	☐	☐	☐	☐	☐	☐	☐	☐	☐	☐	
**3.** Diet fizzy drink (e.g. diet cola, diet lemonade) (can, glass)	☐	☐	☐	☐	☐	☐	☐	☐	☐	☐	☐	☐	
**4.** Energy drink (e.g. Redbull, V, Monster) (can)	☐	☐	☐	☐	☐	☐	☐	☐	☐	☐	☐	☐	
**5.** Diet energy drink (can)	☐	☐	☐	☐	☐	☐	☐	☐	☐	☐	☐	☐	
**6.** Tea (cup)	☐	☐	☐	☐	☐	☐	☐	☐	☐	☐	☐	☐	
**7.** Herbal tea (cup)	☐	☐	☐	☐	☐	☐	☐	☐	☐	☐	☐	☐	
**8.** Green tea (cup)	☐	☐	☐	☐	☐	☐	☐	☐	☐	☐	☐	☐	
**9.** Instant coffee (cup)	☐	☐	☐	☐	☐	☐	☐	☐	☐	☐	☐	☐	
**10.** Ground coffee (e.g. filter coffee, cappuccino, flat white) (cup, mug)	☐	☐	☐	☐	☐	☐	☐	☐	☐	☐	☐	☐	

OFFICE USE ONLY
Q20
1☐☐ 2☐☐ 3☐☐ 4☐☐ 5☐☐ 6☐☐ 7☐☐ 8☐☐ 9☐☐ 10☐☐

Q21 Do you know how much you weigh?

☐ Yes ➔ What is your current weight? ☐☐☐.☐ kg

☐ No → **Go to Q22**

Q22 Are you worried about your weight?

☐ No, not at all ☐ A little ☐ Moderately ☐ Very

Q23 Do you consider yourself to be…

☐ Underweight ☐ Normal weight ☐ A bit overweight ☐ Very overweight

Q24 Over the last 2 weeks...

Please mark **one** *response for each item*	**Not At all**	**Some of the time**	**A lot of the time**	**Most of the time**
**1.** Have you been trying hard to eat less to change your shape or weight? (even if you haven't been able to do so)	☐	☐	☐	☐
**2.** Have you gone for long periods of time (8 h or more) without eating anything to try to change your shape or weight?	☐	☐	☐	☐
**3.** Have you tried not to eat certain foods (like chocolate or chips) to try to change your shape or weight? (even if you haven't been able to do so)	☐	☐	☐	☐
**4.** Have you tried to stick to any definite rules about dieting or eating? (e.g. sticking to calorie limit, a set amount of food or rules about what or when you should eat even if you haven't been able to do so)	☐	☐	☐	☐
**5.** Have you been thinking about food or calories so much that you've found it hard to concentrate or things you are interested in? (e.g. reading, watching TV or following a conversation)	☐	☐	☐	☐
**6.** Have there been times when you feel that you have eaten an unusually large amount of food?	☐	☐	☐	☐
**7.** Have you been afraid of losing control over your eating?	☐	☐	☐	☐
**8.** Have you felt that you couldn't control what or how much you were eating?	☐	☐	☐	☐
**9.** Have you felt that you couldn't stop eating once you had started?	☐	☐	☐	☐
**10.** Have you felt guilty after eating?	☐	☐	☐	☐
**11.** Have you eaten in secret because you are embarrassed by how much you eat?	☐	☐	☐	☐
**12.** Have you been afraid that you might gain weight or become fat?	☐	☐	☐	☐
**13.** Have you felt fat?	☐	☐	☐	☐
**14.** Have you had a strong desire to lose weight?	☐	☐	☐	☐
**15.** Have you made yourself sick (vomit) after eating to try to control your weight?	☐	☐	☐	☐
**16.** Have you taken any pills (like laxatives, water pills or diet pills) to try to control your weight?	☐	☐	☐	☐
**17.** Have you exercised hard to try to control your weight?	☐	☐	☐	☐
People have different ideas about what sort of things are important to them in how they think about themselves. For some people doing well at school is very important to them, for others, how they are getting on with friends is very important. We're now going to ask you to think about how important weight and shape is to you:				
**18.** Has your weight been important in how you think of yourself as a person?	☐	☐	☐	☐
**19.** Has your shape been important in how you think of yourself as a person?	☐	☐	☐	☐

SECTION 6 – Smoking & Drugs

Q25 Do you currently smoke cigarettes/cigars?

- ☐ No → **Go to Q28**
- ☐ Yes

Q26 How many cigarettes/cigars do you smoke per day?
Please mark only **one** *response*

- ☐ Less than one
- ☐ 1 - 5
- ☐ 6 - 10
- ☐ 11 - 15
- ☐ 16 - 20
- ☐ More than 20

Q27 At what age did you start smoking regularly? ☐☐ Years

Q28 Do you currently live with someone who smokes? ☐ No ☐ Yes

Q29 Over the past 3 years, have you lived for more than 6 months with anyone that smokes cigarettes/cigars? ☐ No ☐ Yes

Q30 Have you ever tried or used the following drugs, and if so, on average, how often?

Please mark **one** *response for each item*	Never	Only tried once	Less than monthly	About monthly	About weekly	Daily	Don't know
Marijuana/cannabis	☐	☐	☐	☐	☐	☐	☐
Inhalants (liquids, sprays, gases, nitrites)	☐	☐	☐	☐	☐	☐	☐
Ecstasy	☐	☐	☐	☐	☐	☐	☐
Heroin	☐	☐	☐	☐	☐	☐	☐
Amphetamines (speed, ice, base)	☐	☐	☐	☐	☐	☐	☐
Hallucinogens (acid/LSD)	☐	☐	☐	☐	☐	☐	☐
Nitrous oxide/nangs	☐	☐	☐	☐	☐	☐	☐
Cocaine	☐	☐	☐	☐	☐	☐	☐
Methadone	☐	☐	☐	☐	☐	☐	☐
GHB (liquid ecstasy)	☐	☐	☐	☐	☐	☐	☐
Ketamine "K"	☐	☐	☐	☐	☐	☐	☐
Benzodiazepines	☐	☐	☐	☐	☐	☐	☐
Rohypnol	☐	☐	☐	☐	☐	☐	☐
Something else *(please specify):…………………………..*	☐	☐	☐	☐	☐	☐	☐

SECTION 7 – Eyes & Vision

Q31 Do you, or your mother, or father, or any of your brothers or sisters have or have had, any of the eye problems listed below?

If you don't know, please leave blank.

Please mark **all** *responses that apply*	**You**	**Biological mother**	**Biological father**	**Sister/half-sister**	**Brother/half-brother**
Wear glasses/contact lenses	☐	☐	☐	☐	☐
Blindness	☐	☐	☐	☐	☐
Cataracts	☐	☐	☐	☐	☐
Colourblind	☐	☐	☐	☐	☐
Corneal ulcer	☐	☐	☐	☐	☐
Diabetic retinopathy	☐	☐	☐	☐	☐
Double vision (diplopia)	☐	☐	☐	☐	☐
Dry eye syndrome	☐	☐	☐	☐	☐
Eye injury	☐	☐	☐	☐	☐
Glaucoma	☐	☐	☐	☐	☐
Laser eye surgery	☐	☐	☐	☐	☐
Lazy eye	☐	☐	☐	☐	☐
Long sighted (hypermetropia)	☐	☐	☐	☐	☐
Macular degeneration	☐	☐	☐	☐	☐
Nystagmus	☐	☐	☐	☐	☐
Pterygium (sun damage)	☐	☐	☐	☐	☐
Presbyopia	☐	☐	☐	☐	☐
Ptosis (droopy eyelid)	☐	☐	☐	☐	☐
Retinal detachment	☐	☐	☐	☐	☐
Stargarts disease	☐	☐	☐	☐	☐
Short sighted (myopia)	☐	☐	☐	☐	☐
Strabismus (cross-eyed/squint)	☐	☐	☐	☐	☐
Other eye surgery *(please specify)*	☐ ………..….	☐ …………....	☐ ……..…….	☐ ……….……	☐ ……..…….
Other eye problems *(please specify)*	☐ …………....	☐ …………....	☐ ……….…..	☐ …………...	☐ ……….……
None of these	☐	☐	☐	☐	☐

SECTION 8 – Ultra-Violet (Sun) Exposure

Q32 What is the natural colour of your hair?
Please mark only **one** *response*

- ☐ Blonde
- ☐ Red
- ☐ Brown
- ☐ Black
- ☐ Other *(please specify):* ………………………………………………
- ☐ Don't know

Q33 Without sun tan lotion, what usually happens to your skin after a half hour of being exposed to the bright summer sun for the first time?
Please mark only **one** *response*

- ☐ Never burns or tans
- ☐ Never burns but does tan
- ☐ Burns and then tans
- ☐ Burns but does not tan
- ☐ Don't know

Q34 How many bad sun burns with pain lasting longer than a day would you guess you have had?
Please mark only **one** *response*

- ☐ Never
- ☐ Once
- ☐ 2-10 times
- ☐ More than 10 times
- ☐ Don't know

Q35 In the summer, when not working at your job or at school, what part of the day do you spend outside? *Please mark only* **one** *response*

- ☐ None
- ☐ Less than ¼ of the day
- ☐ ½ of the day
- ☐ Greater than ¾ of the day
- ☐ Cannot judge

Q36 When outdoors in the sun, about what part of the time do you ...?

	Never	Seldom	½ of the time	Usually	Always	Cannot judge
Wear a hat with a brim or a visor?	☐	☐	☐	☐	☐	☐
Wear sunglasses?	☐	☐	☐	☐	☐	☐

Q37 In the winter, where has your leisure or recreation time usually been spent?
Please mark only **one** *response*

- ☐ Mostly indoors
- ☐ 1/2 and 1/2
- ☐ Mostly outdoors
- ☐ Don't know

Q38 Do you often feel colder than the people who are around you?
Please mark only **one** *response*

- ☐ Never
- ☐ Seldom
- ☐ 1/2 of the time
- ☐ Usually
- ☐ Always
- ☐ Cannot judge

Q39 At work or school, do you wear a hat with a visor or brim or sunglasses for more than half of the time?
Please mark only **one** *response*

- ☐ Neither, I don't wear a hat or sunglasses
- ☐ Yes, hat only
- ☐ Yes, sunglasses only
- ☐ Yes, both hat and sunglasses
- ☐ Don't know

Q40 What is the main reason you wear sunglasses?
Please mark only **one** *response*

- ☐ Protection from eye disease
- ☐ Driving
- ☐ Medical condition/doctors' advice
- ☐ Glare
- ☐ Sport
- ☐ Fashion/looks cool
- ☐ School requirement
- ☐ Influenced by family member
- ☐ Other *(please specify):* …………………………………………………

Q41 What is the main reason you do <u>NOT</u> wear sunglasses?
Please mark only **one** *response*

- ☐ Inconvenient
- ☐ Uncomfortable
- ☐ Decreases vision
- ☐ Wear prescription glasses
- ☐ Expensive
- ☐ Not fashionable
- ☐ Not necessary
- ☐ Other *(please specify):* …………………………………………………

SECTION 9 – Mobile Phone Use

Q42 Do you have a mobile phone? *(not a cordless home phone)*

- ☐ No → **Go to Q46**
- ☐ Yes

⬇

Q43 How long have you had your own mobile phone? ☐☐ Years

Q44 Where do you most often keep your mobile phone while you are <u>awake</u>?
Please mark only ***one*** *response*

- ☐ Front jeans/trouser pocket
- ☐ Back jeans/trouser pocket
- ☐ Breast (shirt or jacket) pocket
- ☐ Clipped on belt
- ☐ Handbag
- ☐ Backpack
- ☐ Next to you (e.g. on desk, in car etc.)
- ☐ Around your neck (on a lanyard)
- ☐ In your hand
- ☐ Other *(please specify):* …………………

Q45 Where do you most often keep your mobile phone while you are <u>asleep</u>?
Please mark only ***one*** *response*

- ☐ Handbag
- ☐ Backpack
- ☐ Bedside table
- ☐ Other *(please specify):* ………..…….

SECTION 10 – Mood & Emotions

These questions ask for your views about your health.

Q46 In general, would you say your health is:

☐ Excellent ☐ Very good ☐ Good ☐ Fair Poor

Q47 The following questions are about activities you might do during a typical day. Does your health <u>now</u> limit you in these activities? If so, how much?

Please mark ***one*** *response for each item*	**Yes, limited a lot**	**Yes, limited a little**	**No, not limited at all**
Moderate activities, such as moving a table, pushing a vacuum cleaner, bowling or playing golf	☐	☐	☐
Climbing several flights of stairs	☐	☐	☐

Q48 During the past 4 weeks, how much of the time have you had any of the following problems With your work or other regular daily activities as a result of your physical health?

Please mark **one** *response for each item*	**All of the time**	**Most of the time**	**Some of the time**	**A little of the time**	**None of the time**
Accomplished less than you would like	☐	☐	☐	☐	☐
Were limited in the kind of work or other activities	☐	☐	☐	☐	☐

Q49 During the past 4 weeks, how much of the time have you had any of the following problems with your work or other regular daily activities as a result of any emotional problems (such as feeling depressed or anxious)?

Please mark **one** *response for each item*	**All of the time**	**Most of the time**	**Some of the time**	**A little of the time**	**None of the time**
Accomplished less than you would like	☐	☐	☐	☐	☐
Did work or other activities less carefully than usual	☐	☐	☐	☐	☐

Q50 During the past 4 weeks, how much did pain interfere with your normal work (including both work outside the home and housework)?

☐ Not at all ☐ A little bit ☐ Moderately ☐ Quite a bit ☐ Extremely

These questions are about how you feel and how things have been during the past 4 weeks. For each question, please give the one answer that comes closest to the way you have been feeling.

Q51 How much of the time during the past 4 weeks...

Please mark **one** *response for each item*	**All of the time**	**Most of the time**	**Some of the time**	**A little of the time**	**None of the time**
Have you felt calm and peaceful?	☐	☐	☐	☐	☐
Did you have a lot of energy?	☐	☐	☐	☐	☐
Have you felt downhearted and depressed?	☐	☐	☐	☐	☐

Q52 During the past 4 weeks, how much of the time has your physical health or emotional problems interfered with your social activities (like visiting friends, relatives etc.)?

☐ All of the time ☐ Most of the time ☐ Some of the time ☐ A little of the time ☐ None of the time

The following series of questions relate to your levels of depression, anxiety and stress and provide us with valuable information about your emotional wellbeing:

Q53 Please read each statement and mark either 0, 1, 2 or 3 to indicate how much the statement applied to you over the past week. There are no right or wrong answers. Do not spend too much time on any statement.

The rating scale is as follows:

0 = Did not apply to me at all - Never
1 = Applied to me to some degree, or some of the time - Sometimes
2 = Applied to me a considerable degree, or a good part of the time - Often
3 = Applied to me very much, or most of the time – Almost always

Please mark **one** *response for each item*	**0**	**1**	**2**	**3**
**1.** I found it hard to wind down	☐	☐	☐	☐
**2.** I was aware of dryness of my mouth	☐	☐	☐	☐
**3.** I couldn’t seem to experience any positive feeling at all	☐	☐	☐	☐
**4.** I experienced breathing difficulty (e.g., excessively rapid breathing, breathlessness in the absence of physical exertion)	☐	☐	☐	☐
**5.** I found it difficult to work up the initiative to do things	☐	☐	☐	☐
**6.** I tended to over-react to situations	☐	☐	☐	☐
**7.** I experienced trembling (e.g., in the hands)	☐	☐	☐	☐
**8.** I felt that I was using a lot of nervous energy	☐	☐	☐	☐
**9.** I was worried about situations in which I might panic and make a fool of myself	☐	☐	☐	☐
**10.** I felt that I had nothing to look forward to	☐	☐	☐	☐
**11.** I found myself getting agitated	☐	☐	☐	☐
**12.** I found it difficult to relax	☐	☐	☐	☐
**13.** I felt down-hearted and blue	☐	☐	☐	☐
**14.** I was intolerant of anything that kept me from getting on with what I was doing	☐	☐	☐	☐
**15.** I felt I was close to panic	☐	☐	☐	☐
**16.** I was unable to become enthusiastic about anything	☐	☐	☐	☐
**17.** I felt I wasn’t worth much as a person	☐	☐	☐	☐
**18.** I felt that I was rather touchy	☐	☐	☐	☐
**19.** I was aware of the action of my heart in the absence of physical exertion (e.g., sense of heart rate increase, heart missing a beat)	☐	☐	☐	☐
**20.** I felt scared without any good reason	☐	☐	☐	☐
**21.** I felt that life was meaningless	☐	☐	☐	☐

SECTION 11 – Relationships & Sexual Behaviour

Q54 What is your current relationship status? *Please mark only **one** response*

- ☐ Single and not in a relationship → **Go to Q57**
- ☐ In a relationship but NOT living together
- ☐ In a relationship AND living together
- ☐ Married (in a registered marriage)

Q55 Is your primary partner male or female? ☐ Male ☐ Female

Q56 How old is your partner? ☐☐ Years ☐ Don't know/unsure

Q57 Which of these statements best describes you?
*Please mark only **one** response*

- ☐ I have felt attracted only to females, never to males
- ☐ I have felt attracted more often to females and at least once to a male
- ☐ I am about equally attracted to females and males
- ☐ I have felt attracted more often to males and at least once to a female
- ☐ I have felt attracted only to males, never to females
- ☐ I have never felt attracted to anyone at all

Q58 What do you identify as:
*Please mark only **one** response*

- ☐ Heterosexual (straight)
- ☐ Gay/Lesbian
- ☐ Bisexual
- ☐ Transgender
- ☐ Not sure
- ☐ Other *(please specify):* ……………………………………………………

Regarding your sexual experiences...

Q59 How old were you when you first had an experience of:

*Please mark **one** response for each item*

	Haven't	Under 17 Years	17 Years	18 Years	19 Years	20 Years	Over 20 Years
Deep kissing	☐	☐	☐	☐	☐	☐	☐
Touching a partner's genitals with your hands	☐	☐	☐	☐	☐	☐	☐
Being touched on your genitals by a partner's hand	☐	☐	☐	☐	☐	☐	☐
Giving oral sex	☐	☐	☐	☐	☐	☐	☐
Receiving oral sex	☐	☐	☐	☐	☐	☐	☐
Penis-vaginal intercourse	☐	☐	☐	☐	☐	☐	☐
Anal intercourse (giving or receiving)	☐	☐	☐	☐	☐	☐	☐

Q60 In the past year, have you ever had oral sex or vaginal/anal intercourse when you didn't want to?

- ☐ No → **Go to Q62**
- ☐ Yes → **Go to Q61**

Q61 What were the reasons for this?
Please mark **all** *responses that apply*

- ☐ Had been drinking at the time
- ☐ Was high at the time
- ☐ Partner thought I should
- ☐ Friends thought I should
- ☐ Felt I could not say no
- ☐ Other *(please specify):* ……………………………………………………

Q62 What did you use to avoid pregnancy over the past year?
Please mark **all** *responses that apply*

- ☐ Haven't had intercourse in the last year
- ☐ Nothing
- ☐ Condoms
- ☐ Oral contraceptive (the Pill)
- ☐ Depo provera (injection)
- ☐ Implanon (implant)
- ☐ IUD
- ☐ Morning after pill
- ☐ Diaphragm or cap
- ☐ Withdrawal (pulling out)
- ☐ Other *(please specify):* ……………………………………………………

Q63 In your opinion how likely is it that you might catch a sexually transmissible infection?

☐ Never ☐ Very unlikely ☐ Unlikely ☐ Likely ☐ Very likely

Q64 In the last year, have you ever been diagnosed with a sexually transmissible infection?

- ☐ No → **Go to Q66**
- ☐ Yes

Q65 Which sexually transmitted infections have you been diagnosed with?
Please mark **all** *responses that apply*

- ☐ Candaisis/Thrush
- ☐ Chlamydia
- ☐ Genital herpes
- ☐ Genital warts
- ☐ Gonorrhoea
- ☐ Hepatitis B
- ☐ HIV/AIDS
- ☐ Pubic lice/crabs
- ☐ Syphillis
- ☐ Other *(please specify):* …………………………………………………..

Q66 How much would you like to become a parent sometime soon?
Please mark only **one** *response*

- ☐ I am already a parent
- ☐ I really want to be a parent soon
- ☐ It would be nice to be a parent soon
- ☐ I don't care if I do or don't become a parent soon
- ☐ I would prefer not to be a parent soon
- ☐ I really don't want to be a parent soon

For Women Only – Men Go To Section 12

Q67 How often do you usually have a menstrual period?

- ☐ Never → **Go to Q71**
- ☐ Very irregularly
- ☐ Less than once per month
- ☐ More than once per month
- ☐ Every month

Q68 Using the scale below where 0 is the least pain and 10 is the worst pain, how would you describe the worst pain you commonly experience during your menstrual cycle?

0 ☐ ☐ ☐ ☐ ☐ ☐ ☐ ☐ ☐ ☐ **10**

None … **Unbearable**

Q69 Pain	Yes	No	N/A
Do you regularly experience pelvic pain that is not during your period?	☐	☐	☐
Do you regularly experience pain during intercourse	☐	☐	☐
Do you regularly take medication for cramps or pelvic pain?	☐	☐	☐

Q70 How heavy is your bleeding?	Yes	No	N/A
Do you regularly use "super" or "super plus" pads or tampons?	☐	☐	☐
Do you regularly need to use two pads or a pad and a tampon at the same time?	☐	☐	☐
Do you ever soak your clothes or bed clothes with blood?	☐	☐	☐
How often do you need to change your pad or tampon on the heaviest day of bleeding?	☐☐ **Times**		

Q71 Do you currently use contraception?

- ☐ No → **Go to Section 12**
- ☐ Yes

Q72 What kind(s) do you use?

If you do not use the contraceptive pill please go to Section 12

Q73 Why do you take hormones (the pill)? *Please mark* **all** *responses that apply*

- ☐ To prevent pregnancy
- ☐ For painful periods
- ☐ For heavy periods
- ☐ Other *(please specify):* ………………………………………..

OFFICE USE ONLY

Q72 ☐☐

SECTION 12 – Respiratory Questions

WHEEZE

Q74 Have you wheezed in the past 12 months?

☐ No → **Go to Q78**
☐ Yes

Q75 In the past 12 months, how often on average has your sleep been disturbed due to wheezing?

☐ Never woken with wheezing
☐ Less than one night per week
☐ One or more nights per week
☐ Don't know

Q76 Has the wheezing been severe enough to limit your speech to only one or two words at a time between breaths?

☐ No ☐ Yes ☐ Don't Know

Q77 Has your chest sounded wheezy during or after exercise?

☐ No ☐ Yes ☐ Don't Know

ASTHMA

Q78 Do you think you have ever had asthma?

☐ No ☐ Yes ☐ Don't Know

Q79 Has a doctor (GP, paediatrician, respiratory specialist) ever told you that you have asthma?

☐ No ☐ Yes ☐ Don't Know

Q80 Do you still have asthma?

☐ No ☐ Yes ☐ Don't Know ☐ Never had asthma

Q81 Have you used/taken any asthma medications in the past 12 months?

☐ No → **Go to Q83**
☐ Yes → **Go to Q82**

Q82 Which asthma medications have you used/taken in the past 12 months?

*Please mark **all** appropriate answers*	Yes	Ordered by a Doctor Yes	Ordered by a Doctor No
Ventolin (Asmol, Airomir, etc.)	☐	☐	☐
Respolin	☐	☐	☐
Bricanyl	☐	☐	☐
QVAR	☐	☐	☐
Flixotide	☐	☐	☐
Pulmacort	☐	☐	☐
OXIS	☐	☐	☐
Serevent	☐	☐	☐
Singulaire	☐	☐	☐
Seretide	☐	☐	☐
Symbicort	☐	☐	☐
Prednisolone	☐	☐	☐
Other *(please specify)*:……………………………………………	☐	☐	☐

Q83 What triggers your asthma?
*Please mark **all** responses that apply*

- ☐ Viral infection
- ☐ Grass
- ☐ Pollen
- ☐ Animal
- ☐ Dust
- ☐ Other *(please specify)*: ………………………………………………..
- ☐ Don't know
- ☐ Don't have asthma

RHINITIS (runny or blocked nose - including hayfever)

Q84 In the past 12 months, have you had a problem with sneezing or a runny or blocked nose (including hayfever) when you DID NOT have a cold or flu?

- ☐ No → **Go to Q91**
- ☐ Yes

Q85 In the past 12 months, was this nose problem accompanied by itchy-watery eyes?

☐ No ☐ Yes

Q86 In the past 12 months, how many episodes of allergic nose problem have you had (including hayfever)?

- ☐ 1 - 2 episodes
- ☐ 3 - 12 episodes

☐ More than 12 episodes

Q87 In which of the past 12 months did this problem occur?
*Please mark **all** responses that apply*

☐ January ☐ July
☐ February ☐ August
☐ March ☐ September
☐ April ☐ October
☐ May ☐ November
☐ June ☐ December

Q88 Has a doctor (GP, paediatrician, respiratory specialist) ever told you that you have an allergic nose problem (including hayfever)?

☐ No ☐ Yes

Q89 What was the trigger/cause of these problems?
*Please mark **all** responses that apply*

☐ Grass
☐ Pollen
☐ Animal
☐ Dust
☐ Other *(please specify)*: ……………………………………………...
☐ Don't know

Q90 In the past 12 months, have you taken or used any medication for allergic nose (including hayfever)?

☐ No → **Go to Q91**
☐ Yes

↓

Please list the medication and indicate if it was prescription or non-prescription.

Type of medication	Not Prescribed by doctor	Prescribed by doctor
Steroid nasal spray *(please specify):*	☐	☐
Non-steroid nasal spray *(please specify):*	☐	☐
Antihistamine drops/tablets *(please specify):*	☐	☐
Other *non-prescription* medication *(please specify):*	☐	
Other *prescription* medication *(please specify):*		☐

ALLERGIC CONJUNCTIVITIS (itchy water eyes - including hayfever)

Q91 Do you think that you have ever had an allergic reaction in the eyes (including hayfever)?

☐ No
☐ Yes
☐ Don’t know

Q92 Has a doctor (GP, paediatrician, respiratory specialist) ever told you that you had an allergic

reaction in the eyes (including hayfever)?

- ☐ No
- ☐ Yes
- ☐ Don't know

Q93 In the past 12 months, have you suffered from an allergic reaction in the eyes (including hayfever)?

- ☐ No → **Go to Q98**
- ☐ Yes

Q94 In the past 12 months, how many episodes of allergic reaction in the eyes have you had (including hayfever)?

- ☐ 1 - 2 episodes
- ☐ 3 - 12 episodes
- ☐ More than 12 episodes

Q95 In which of the past 12 months did this problem occur?
*Please mark **all** responses that apply*

- ☐ January
- ☐ February
- ☐ March
- ☐ April
- ☐ May
- ☐ June
- ☐ July
- ☐ August
- ☐ September
- ☐ October
- ☐ November
- ☐ December

Q96 What was the trigger/cause of these problems?
*Please mark **all** responses that apply*

- ☐ Grass
- ☐ Pollen
- ☐ Animal
- ☐ Dust
- ☐ Other *Please specify:*………………………………………………..
- ☐ Don't know

Q97 In the past 12 months, have you taken or used any medication for allergic reaction in the eyes (including hayfever)?

- ☐ No → **Go to Q98**
- ☐ Yes

Please list the medication and indicate if it was prescription or non-prescription.

**Type of medication**	**Not Prescribed by doctor**	**Prescribed by doctor**
Eye drops *(please specify):*	☐	☐
Steroid tablets *(please specify):*	☐	☐
Antihistamine drops/tablets *(please specify):*	☐	☐

Other *non-prescription* medication *(please specify):*	☐
Other *prescription* medication *(please specify):*	☐

ECZEMA (itchy rash)

Q98 Have you <u>ever</u> had eczema or an itchy rash which was coming and going for at least <u>12 months</u>?

☐ No → **Go to Q108**
☐ Yes

Q99 Has this eczema/itchy rash at any time affected any one of the following places: the folds of the elbows, behind the knees, in front of the ankles, under the buttocks or around the neck, ears or eyes?

☐ No ☐ Yes

Q100 In the <u>past 12 months</u>, how often on average have you been kept awake at night by this itchy rash?

☐ Never in the last 12 months
☐ Less than one night per week
☐ One or more nights per week
☐ Don't know

Q101 Has this rash cleared completely during the <u>past 12 months</u>?

☐ No ☐ Yes

Q102 Do you think you have ever had eczema?

☐ No ☐ Yes

Q103 Has a doctor (GP, paediatrician) ever told you that you have eczema?

☐ No ☐ Yes ☐ Don’t Know

Q104 In the <u>past 12 months</u>, have you suffered from eczema?

☐ No → **Go to Q108**

☐ Yes

↓

Q105 In the past 12 months, how many episodes of eczema have you had?

☐ 1 - 2 episodes
☐ 3 - 12 episodes
☐ More than 12 episodes

Q106 In which of the past 12 months did the eczema occur?
*Please mark **all** responses that apply*

☐ January ☐ July
☐ February ☐ August
☐ March ☐ September
☐ April ☐ October
☐ May ☐ November
☐ June ☐ December

Q107 In the past 12 months, have you taken or used any medication for eczema?

☐ No → **Go to Q108**
☐ Yes

↓

Please list the medication and indicate if it was prescription or non-prescription.

**Type of medication**	**Not Prescribed by doctor**	**Prescribed by doctor**
Moisturiser *(please specify):*	☐	☐
Steroid cream *(please specify):*	☐	☐
Oral steroids *(please specify):*	☐	☐
Tacrolimus ointment *(please specify):*	☐	☐
Other *non-prescription* medication *(please specify):*	☐	
Other *prescription* medication *(please specify):*		☐

Q108 Do you have any food allergies?

☐ No → **Go to Q110**
☐ Yes

↓

Q109 What are you allergic to?
*Please mark **all** responses that apply*

- [ ] Peanut products
- [ ] Wheat/yeast
- [ ] Dairy
- [ ] Fruit
- [ ] Eggs
- [ ] Seafood
- [ ] Preservatives/colouring
- [ ] Other *(please specify):* ……………………………………………………

Q110 Date questionnaire completed: ☐☐ / ☐☐ / ☐☐☐☐

THANK YOU

WE APPRECIATE THE TIME THAT YOU HAVE SPENT COMPLETING THIS QUESTIONNAIRE

ID

OFFICE USE ONLY
RA-CH RA-CO RA1-E RA2-E

ID

GROWING UP HEALTHY STUDY

Medical History Questionnaire

20-22

Thank you for giving your time to fill in this questionnaire

The purpose of this questionnaire is to obtain information about your health and wellbeing

Please read each question carefully and answer all of the questions.
Write your answers clearly in the space provided or mark the most appropriate response.

☒

All information will be strictly confidential

Please take your time in answering all of the questions

If you require assistance to answer any of the questions please contact the Study Coordinator:

Blagica & Tina
T: +61 6458 1443
M: 0439 266 434
Email: ***guhstudy-swih@uwa.edu.au***

Confidential

Q1 Do you have now, or have you had in the past, any of the following health professional diagnosed medical conditions or health problems?

Please mark **one** *response for each item*	**No**	**Yes, in the past**	**Yes, now**	**Yes, now and in the past**
Acne	☐	☐	☐	☐
Anxiety problems	☐	☐	☐	☐
Arthritis or joint problems	☐	☐	☐	☐
Asthma	☐	☐	☐	☐
Attentional problems	☐	☐	☐	☐
Back pain	☐	☐	☐	☐
Behavioural problems	☐	☐	☐	☐
Bladder control problems	☐	☐	☐	☐
Chronic respiratory or breathing problems (other than asthma)	☐	☐	☐	☐
Coeliac disease	☐	☐	☐	☐
Co-ordination or clumsiness difficulties	☐	☐	☐	☐
Depression	☐	☐	☐	☐
Developmental disorder (e.g. attention deficit disorder, autism, intellectual disability)	☐	☐	☐	☐
Diabetes	☐	☐	☐	☐
Eating disorder/weight problems	☐	☐	☐	☐
Hayfever or some other allergy	☐	☐	☐	☐
Hearing impairment or deafness	☐	☐	☐	☐
Heart condition	☐	☐	☐	☐
Hemochromatosis (iron overload disease)	☐	☐	☐	☐
Intellectual disability	☐	☐	☐	☐
Learning problems	☐	☐	☐	☐
Menstrual problems	☐	☐	☐	☐
Migraine or severe headache	☐	☐	☐	☐
Neck pain	☐	☐	☐	☐
Sleep disturbance	☐	☐	☐	☐
Speech and/or language problems	☐	☐	☐	☐
Thyroid gland problems	☐	☐	☐	☐
Vision problems	☐	☐	☐	☐
Any other medical condition or health problem not mentioned here	☐	☐	☐	☐

Q2 If you have answered "Yes" to any of the health problems in the previous question, or have any other health professional diagnosed problem or condition, please describe the condition or problem in more detail below (e.g. long sighted - wear glasses for reading; diagnosed with attention deficit disorder; asthma requiring medication)

Please list every medical condition/health problem separately - otherwise leave this blank.

What condition/problem?	Who diagnosed it?	When was it diagnosed?	Treatment
e.g. Impacted wisdom teeth	*Dentist*	*6 months ago*	*Referral to dental surgeon, antibiotics*

Q3 In the past 12 months, have you attended any of the following?

☐ No → **Go to Q4**
☐ Yes

*Please mark **one** response for each item*	No	Yes, in the past	Yes, now	Yes, now and in the past
GP or family doctor	☐	☐	☐	☐
Accident and emergency	☐	☐	☐	☐
Hospital outpatient (department or clinic)	☐	☐	☐	☐
Private medical specialist	☐	☐	☐	☐
Dentist/Dental therapist/Orthodontist	☐	☐	☐	☐
School nurse	☐	☐	☐	☐
Optician/Optometrist	☐	☐	☐	☐
Dietician/Nutritionist	☐	☐	☐	☐
Physiotherapist	☐	☐	☐	☐
Occupational therapist (OT)	☐	☐	☐	☐
Speech therapist	☐	☐	☐	☐
Psychologist/Psychiatrist	☐	☐	☐	☐
Podiatrist	☐	☐	☐	☐
Chiropractor	☐	☐	☐	☐
Alternative therapist (e.g. iridologist)	☐	☐	☐	☐

OFFICE USE ONLY
Q2
1
5
9
13

Q4 In the past 6 months, have you taken/used any prescription medication(s)?

☐ No → **Go to Q5**
☐ Yes

Which medication(s)?

Name	Reason for taking it	Are you still taking it?
e.g. Antibiotics<br>*Ventolin*<br>*Cortisone cream*<br>*The Pill or Depo-Provera*	*For acne*<br>*For asthma*<br>*For eczema*<br>*For acne, menstrual disorders or contraception*	*Yes*<br>*Yes*<br>*No*<br>*Yes*

Q5 In the past 6 months, have you taken/used any 'over the counter' medication(s) *(including vitamins, minerals and health food products)?*

☐ No → **Go to Q6**
☐ Yes

Which medication(s)?

Name	Reason for taking it	Are you still taking it?
e.g. Neurofen<br>*Antihistamine*<br>*Fish oil capsules*	*For period pain*<br>*For hayfever*<br>*For ADD*	*Yes*<br>*No*<br>*Yes*

OFFICE USE ONLY

Q4 (boxes 1–20, two rows)

Q5 (boxes 1–20, plus 1–10)

Q6 Have you ever had any accidents or injuries which required you to go to a Doctor (GP), hospital or clinic?

☐ No → **Go to Q7**
☐ Yes

Please describe the accident, the injury and any treatment (e.g. fell off bike, cut arm, 3 stitches), and list every accident/injury separately, giving as much detail as possible.

**Injury**	**How did it happen?**	**When did it happen?**	**Treatment**
e.g. Sprained wrist	*Fell down stairs*	*3 months ago*	*Physiotherapy/bandage*

Q7 Have you ever been admitted to a hospital/day surgery?

☐ No → **Go to Q8**
☐ Yes

Please list each admission separately, giving as much detail as possible.

**Date**	**Which hospital?**	**Reason for admission**
e.g. October 2005	*McCourt St Day Surgery*	*Removal of impacted wisdom teeth*

OFFICE USE ONLY

Q6
1
2
3
4
5

Q7
1 / /
2 / /
3 / /
4 / /
5 / /

Q8 Please write below any comments concerning this questionnaire, the research, or anything else you would like to tell us about:

Q9 Date questionnaire completed: __ __ / __ __ / __ __ __ __

THANK YOU

WE APPRECIATE THE TIME THAT YOU HAVE SPENT COMPLETING THIS QUESTIONNAIRE

ID __ __ __ __ __

OFFICE USE ONLY

RA-CH ☐☐ RA-CO ☐☐ RA1-E ☐☐ RA2-E ☐☐

ID ☐☐☐☐☐

GROWING UP HEALTHY STUDY – 20-22
THINKING STYLES Questionnaire

All information is strictly confidential

Date ☐☐ / ☐☐ / ☐☐☐☐

This questionnaire assesses your systematic (logical/ organised) and empathetic (understanding/ sympathetic) patterns of thinking.

- *Please read each statement very carefully and rate how strongly you agree or disagree with it*
- *Please do not miss any statement out (there are 3 pages)*
- *Please answer as honestly as possible*
- *Please return with the completed Participant and Medical History Questionnaire.*

	Example of how to complete the questions:	***Definitely agree***	***Slightly agree***	***Slightly disagree***	***Definitely disagree***
1	*I am willing to take risks.*	☒	☐	☐	☐
2	*I find learning to play musical instruments easy.*	☐	☐	☐	☒

Please read the following and indicate how strongly you agree or disagree with the statement:

		**Definitely agree**	**Slightly agree**	**Slightly disagree**	**Definitely disagree**
**1**	I prefer to do things with others rather than on my own.	☐	☐	☐	☐
**2**	I prefer to do things the same way over and over again.	☐	☐	☐	☐
**3**	If I try to imagine something, I find it very easy to create a picture in my mind.	☐	☐	☐	☐
**4**	I frequently get so strongly absorbed in one thing that I lose sight of other things.	☐	☐	☐	☐
**5**	I often notice small sounds when others do not.	☐	☐	☐	☐
**6**	I usually notice car number plates or similar strings of information.	☐	☐	☐	☐
**7**	Other people frequently tell me that what I've said is impolite, even though I think it is polite.	☐	☐	☐	☐
**8**	When I'm reading a story, I can easily imagine what the characters might look like.	☐	☐	☐	☐
**9**	I am fascinated by dates.	☐	☐	☐	☐
**10**	In a social group, I can easily keep track of several different people's conversations.	☐	☐	☐	☐

		Definitely agree	Slightly agree	Slightly disagree	Definitely disagree
11	I find social situations easy.	☐	☐	☐	☐
12	I tend to notice details that others do not.	☐	☐	☐	☐
13	I would rather go to a library than a party.	☐	☐	☐	☐
14	I find making up stories easy.	☐	☐	☐	☐
15	I find myself drawn more strongly to people than to things.	☐	☐	☐	☐
16	I tend to have very strong interests which I get upset about if I can't pursue.	☐	☐	☐	☐
17	I enjoy social chit-chat.	☐	☐	☐	☐
18	When I talk, it isn't always easy for others to get a word in edgeways.	☐	☐	☐	☐
19	I am fascinated by numbers.	☐	☐	☐	☐
20	When I'm reading a story, I find it difficult to work out the characters' intentions.	☐	☐	☐	☐
21	I don't particularly enjoy reading fiction.	☐	☐	☐	☐
22	I find it hard to make new friends.	☐	☐	☐	☐
23	I notice patterns in things all the time.	☐	☐	☐	☐
24	I would rather go to the theatre than a museum.	☐	☐	☐	☐
25	It does not upset me if my daily routine is disturbed.	☐	☐	☐	☐
26	I frequently find that I don't know how to keep a conversation going.	☐	☐	☐	☐
27	I find it easy to "read between the lines" when someone is talking to me.	☐	☐	☐	☐
28	I usually concentrate more on the whole picture, rather than the small details.	☐	☐	☐	☐
29	I am not very good at remembering phone numbers.	☐	☐	☐	☐
30	I don't usually notice small changes in a situation, or a person's appearance.	☐	☐	☐	☐
31	I know how to tell if someone listening to me is getting bored.	☐	☐	☐	☐
32	I find it easy to do more than one thing at once.	☐	☐	☐	☐

		Definitely agree	Slightly agree	Slightly disagree	Definitely disagree
33	When I talk on the phone, I'm not sure when it's my turn to speak.	☐	☐	☐	☐
34	I enjoy doing things spontaneously.	☐	☐	☐	☐
35	I am often the last to understand the point of a joke.	☐	☐	☐	☐
36	I find it easy to work out what someone is thinking or feeling just by looking at their face.	☐	☐	☐	☐
37	If there is an interruption, I can switch back to what I was doing very quickly.	☐	☐	☐	☐
38	I am good at social chit-chat.	☐	☐	☐	☐
39	People often tell me that I keep going on and on about the same thing.	☐	☐	☐	☐
40	When I was young, I used to enjoy playing games involving pretending with other children.	☐	☐	☐	☐
41	I like to collect information about categories of things (e.g. types of car, types of bird, types of train, types of plant, etc.).	☐	☐	☐	☐
42	I find it difficult to imagine what it would be like to be someone else.	☐	☐	☐	☐
43	I like to plan any activities I participate in carefully.	☐	☐	☐	☐
44	I enjoy social occasions.	☐	☐	☐	☐
45	I find it difficult to work out people's intentions.	☐	☐	☐	☐
46	New situations make me anxious.	☐	☐	☐	☐
47	I enjoy meeting new people.	☐	☐	☐	☐
48	I am a good diplomat.	☐	☐	☐	☐
49	I am not very good at remembering people's date of birth.	☐	☐	☐	☐
50	I find it very easy to play games with children that involve pretending.	☐	☐	☐	☐

THANK YOU

WE APPRECIATE THE TIME THAT YOU HAVE SPENT COMPLETING THIS QUESTIONNAIRE

ID ☐☐☐☐☐
